# Mono- and Bimetallic [Cp*Rh] Complexes of the 4,6-Bis(pyridyl)pyrimidine Ligand

**DOI:** 10.1021/acs.organomet.6c00293

**Published:** 2026-10-16

**Authors:** Jiayi Yu, Charlie O. Griebel, Thalia R. O’Neill, Robert D. Pike, Davide Lionetti

**Affiliations:** † Department of Chemistry, 4400 Franklin & Marshall College, P.O. Box 3003, Lancaster, Pennsylvania 17604, United States; ‡ Department of Chemistry, 8604 College of William & Mary, P.O. Box 8795, Williamsburg, Virginia 23187, United States

## Abstract

Multinucleating polyheterocyclic platforms are intriguing ligands for half-sandwich rhodium complexes, as they could engender various modes of cooperation in electron- and proton-transfer reactions. Herein, we report the synthesis and spectroscopic, structural, and electrochemical characterization of mono- and bimetallic pentamethylcyclopentadienyl (Cp*) rhodium complexes of the 4,6-bis­(pyridyl)­pyrimidine (bppm) ligand. These complexes undergo two-electron reduction of each [Cp*Rh] site at potentials that depend on complex nuclearity ([Rh_1_] vs [Rh_2_]) and on the monodentate ligand at Rh (Cl^–^ vs NCCH_3_). Spectroscopic and structural analysis of a chemically prepared monorhodium­(I) complex confirmed significant delocalization of electron density onto the bppm platform. While [Rh-Cl] bppm complexes displayed enhanced stability vs literature compounds supported by related polyheterocyclic scaffolds, various modes of bidentate ligand exchange were observed in their solvento analogues. Electrochemical analyses revealed that dissociation of a bidentate motif from Rh could be induced by electron transfer (ET), a transformation that parallels the well-established ET-induced dissociation of monodentate ligands in these species. Investigation of complexes of the literature 2,2’-bipyrimidine (bpym) ligand demonstrated analogous bidentate ligand exchange reactivity in this system, suggesting that these processes may be more generally operative in [Cp*Rh] complexes supported by binucleating platforms.

## Graphical abstract

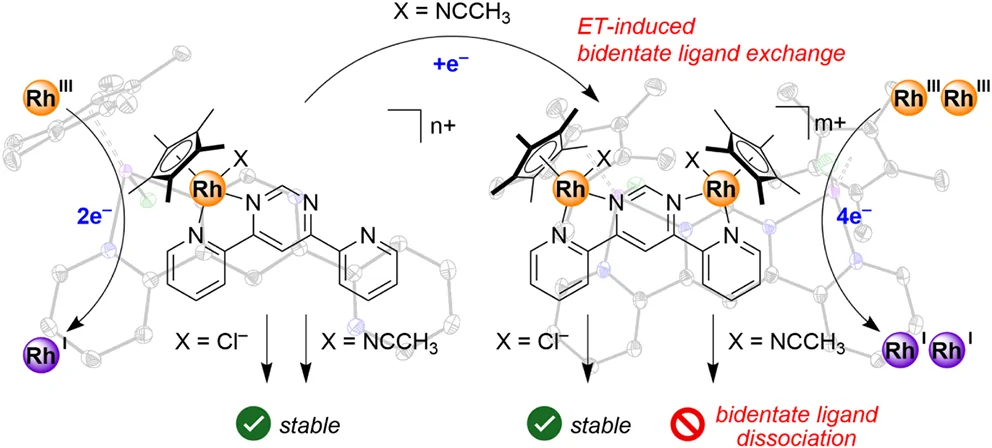

## Introduction

Rhodium complexes containing cyclopentadienyl-type ligands (e.g., pentamethylcyclopentadienyl, Cp*) and polypyridyl platforms such as 2,2’-bipyridine (bpy) catalyze the electrochemical conversion of electrons (e^–^) and protons (H^+^) into gaseous hydrogen (H_2_),^1–7^ as well as various related hydride transfer reactions.^8–15^ In this reactivity, management of e^–^ and H^+^ equivalents is achieved via complementary participation of different ligands: reduction of Rh­(III) complexes to Rh­(I) is facilitated by delocalization of electron density onto the redox-noninnocent bpy frameworks,^16,17^ while protonation of these reduced species can result in transfer of the incoming H^+^ moiety to the Cp* platform, which acts as a pendant base in this context.^13,18–20^ Intriguingly, the ability to access cyclopentadi*ene* ([(Cp*H)­Rh]) intermediates is correlated with competence in H_2_ evolution electrocatalysis, as [Cp*Rh] complexes supported by phosphine-containing ligands, which form traditional rhodium–hydride complexes, are not electrocatalytically active under the same conditions that lead to H_2_ evolution from complexes containing bpy ligands.^7,21–23^ [(Cp*H)­Rh] species have also been identified following hydride (H^–^) transfer to Rh­(III) precursors,^13^ suggesting a broader relevance for these intermediates in catalytic transformations.^20,24^

Polyheterocyclic platforms with multiple metal-binding motifs represent an intriguing class of ligands for [Cp*Rh] complexes, as they could enable additional modes of metal–metal and metal–ligand cooperation. For example, incorporation of multiple [Cp*Rh] fragments on the same ligand scaffold could facilitate storage of additional reducing equivalents at mild potentials.^25,26^ Conversely, even in the absence of a second [Cp*Rh] site, a more extensive aromatic ligand could facilitate ligand participation in ET; the presence of proton-responsive motifs (such as uncoordinated heterocyclic moieties) in the vicinity of the single Rh center could also complement management of H^+^ equivalents by Cp*.^27–29^ Studies of the properties engendered by these ligands could thus enable new strategies for tuning reactivity in [Cp*Rh] complexes.

Several [Cp*Rh] compounds supported by polyheterocyclic frameworks with two distinct bidentate (N,N) donor motifs have appeared in the literature. Monorhodium complexes supported by the 3,6-bis­(2-pyridyl)-4-phenylpyridazine (bppd, Chart [Fig chart1]) and 3,6-bis­(pyrazolyl)­pyridazine (bpzpd) frameworks were reported by Rao and coworkers.^30,31^ Dirhodium complexes supported by the 2,5-bis­(phenyliminoethyl)­pyrazine (bpip) and 3,6-bis­(pyridyl)-1,2,4,5-tetrazine (bptz) platforms were described by Kaim and coworkers,^25,26^ while the group of Koizumi reported the [Cp*Rh]_2_ complex of the anionic 3,5-bis­(pyridyl)­pyrazolate (bpp) framework.^32^ Only two ligands have been leveraged to support both mono- and dirhodium complexes of this type—the 2,2’-bipyrimidine (bpym) scaffold, reported independently by the groups of Kaim and Štěpnička and Süss-Fink,^33–35^ and the 2,3-bis­(pyridyl)­pyrazine (bppz) ligand, investigated by Kaim,^36^ Rao,^37^ and Pandey.^38–40^

**1 chart1:**
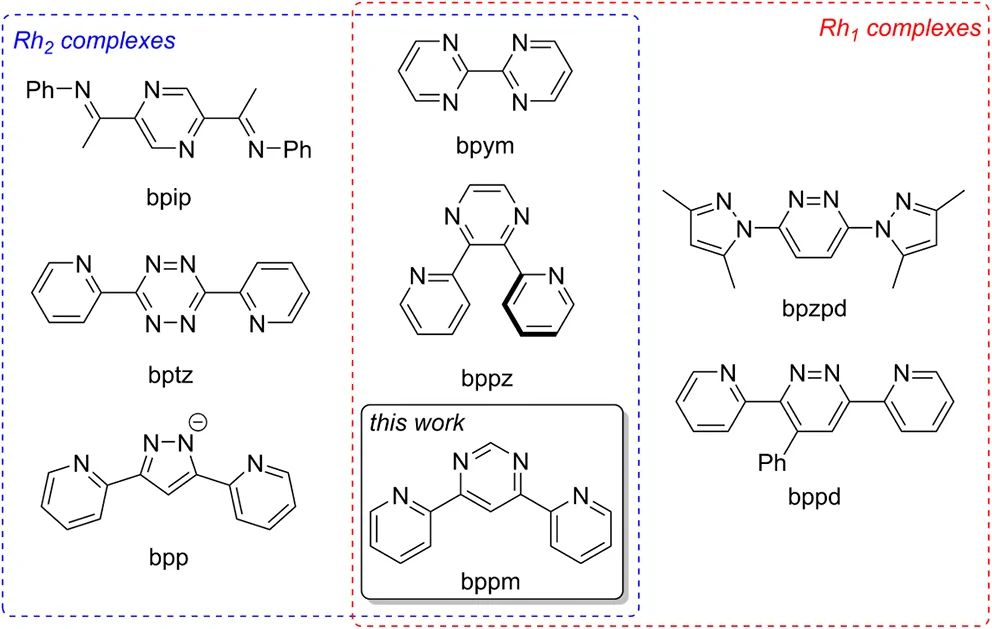
Binucleating Polyheterocyclic Ligands used to Support [Cp*Rh] and [Cp*Rh]_2_ Complexes

While electrochemical analyses have been reported for some of these complexes, systematic ET studies involving mono- and dirhodium complexes supported by the same multinucleating platform have been less frequent,^34,36^ and no reports have appeared in the literature involving compounds of this type in electrocatalytic H_2_ evolution. Furthermore, the platforms for which both [Rh_1_] and [Rh_2_] compounds have been investigated (bpym and bppz) impose significant steric or electronic limitations on their [Rh_2_] complexes: in bpym complexes, both Rh centers share donors from both ligand heterocycles, weakening binding of the second [Cp*Rh] fragment and leading to diminished stability in solution.^33^ Conversely, the steric clash resulting from the *ortho* position of the two pyridyl groups in the bppz framework leads to deviation of this scaffold from planarity and likely influences its participation in ET.^36,38^

Here, we report the [Cp*Rh] complexes of the 4,6-bis­(pyridyl)­pyrimidine framework (bppm, Chart [Fig chart1]). The reduced steric demands enforced by this ligand and the presence of a single shared pyrimidine donor enhance the stabilization of both mono- and bis-[Cp*Rh] complexes featuring Cl^–^ or CH_3_CN ligands vs literature polyheterocyclic scaffolds, enabling direct comparison of spectroscopic and structural features as a function of complex nuclearity and coordination environment at Rh. Electrochemical analyses revealed rich ET profiles indicating 2e^–^ reduction of each Rh site at potentials that depend on both complex nuclearity and the nature of the monodentate ligand at Rh. Analysis of a monorhodium complex in the Rh­(I) oxidation state confirmed participation of the bppm framework in the stabilization of reduced species. During these investigations, evidence was obtained for dissociation of bidentate moieties following reduction to Rh­(II), resulting in interconversion between mono- and dirhodium complexes under electrochemical conditions—a hitherto understudied reactivity manifold in compounds of this type. The preparation of mono- and dirhodium complexes of bppm, their spectroscopic and structural characterization, and the electrochemical analyses of these complexes, including in the context of H_2_ evolution catalysis, are presented. The reactivity of complexes supported by the literature bpym framework under chemical and electrochemical conditions is also revisited.

## Experimental Section

### General Considerations

Manipulations involving air-stable compounds were carried out on the benchtop without exclusion of air or water except where otherwise noted. Air-sensitive compounds were prepared and manipulated in an N_2_-filled glovebox (MBraun) or under N_2_ using standard Schlenk techniques. All solvents were of commercial grade; all chemicals were obtained from major commercial suppliers and used as received unless otherwise noted. THF, CH_3_CN, and toluene were dried over activated alumina using a solvent purification system (JC Meyer) prior to use and were stored over molecular sieves. Et_2_O was dried over sodium/benzophenone and stored over molecular sieves. RhCl_3_·xH_2_O was obtained from Pressure Chemical. Organic reagents were obtained from Ambeed, TCI, or Sigma-Aldrich and used as received. Silver salts were obtained from Strem. [Cp*RhCl_2_]_2_,^41^ [Cp*Rh­(NCCH_3_)_3_]­(PF_6_)_2_,^42^ and bpym^43^ were prepared as previously described. Deuterated NMR solvents were purchased from TCI, Oakwood, Sigma-Aldrich, or Cambridge Isotope Laboratories. CD_3_CN was dried over CaH_2_, and C_6_D_6_ was dried over sodium/benzophenone. ^1^H, ^13^C, ^19^F, and ^31^P NMR spectra were collected on a 500 MHz Agilent spectrometer or on a 400 MHz Bruker NeoAvance spectrometer and referenced to the residual protio solvent signal in the case of ^1^H and ^13^C.^44^ ^31^P NMR spectra were referenced to 85% aqueous H_3_PO_4_ and ^19^F NMR spectra were referenced to CFCl_3_ following the recommended scale based on ratios of absolute frequencies (Ξ).^45,46^ ^15^N NMR chemical shifts were determined via ^1^H–^15^N heteronuclear multiple-bond correlation (HMBC) spectroscopy (^15^N: 41 MHz); a 100 ms delay was used for evolution of long-range couplings. The number of data points was 1024 (*f*_2_ = ^1^H) × 128 (*f*_1_ = ^15^N); this matrix was zero filled to 4096 × 512 prior to FT.^47^ A capillary containing a 0.4 M solution of CH_3_NO_2_ in the same deuterated solvent as the sample was used for referencing ^15^N data to CH_3_NO_2_. Chemical shifts (δ) are reported in units of ppm, and coupling constants (*J*) are reported in Hz. Elemental analyses were performed by Midwest Microlab (Indianapolis, IN). No uncommon hazards are noted.

### X-ray Crystallography

Crystals were mounted on MiTeGen MicroMounts using paratone oil. All measurements were made at 100 K using Mo Kα radiation on a Bruker-AXS D8 Venture four-circle diffractometer, equipped with a microfocus tube and a Photon 3 CPAD detector. Initial space-group determination was based on a fast scan with 180 frames. The data were reduced using SAINT+,^48,49^ and empirical absorption correction applied using SADABS.^50^ Structures were solved using intrinsic phasing (SHELXT).^51^ Least-squares refinement for all structures was carried out on *F* ^2^. The non-hydrogen atoms were refined anisotropically. Hydrogen atoms were placed in riding positions and refined isotropically. Structure refinement and calculation of derived results were performed using Olex software equipped with the SHELX package of computer programs.^52,53^

### Electrochemistry

Electrochemical experiments were carried out under N_2_ or Ar. 0.1 M tetra­(*n*-butylammonium) hexafluorophosphate (electrochemical grade, Sigma-Aldrich) in acetonitrile served as the supporting electrolyte. Measurements were made with a Pine Research WaveDriver 40 potentiostat/galvanostat using a standard three-electrode configuration. The working electrode was the basal plane of highly oriented pyrolytic graphite (HOPG; GraphiteStore.com, Buffalo Grove, IL; surface area: 0.090 cm^2^) or a circular glassy carbon electrode with a PTFE shroud (Pine Research; surface area: 0.0707 cm^2^). The counter electrode was a platinum wire (Kurt J. Lesker, Jefferson Hills, PA; 99.99%, 0.5 mm diameter) and a silver wire immersed in CH_3_CN electrolyte solution and separated from the working solution by a Vycor frit (CH Instruments, Sigma-Aldrich) served as a pseudoreference electrode. Ferrocene was added to the electrolyte solution at the conclusion of each experiment (∼1 mM); the midpoint potential of the ferrocenium/ferrocene couple (denoted as Fc^+/0^) served as an external standard for comparison of the recorded potentials. Concentrations of the analyte for cyclic voltammetry were typically 2 mM.

### Synthesis of 4,6-Bis­(pyridyl)­pyrimidine (bppm)

4,6-Bis­(pyridyl)­pyrimidine was prepared via modification of a literature procedure for 2,5-bis­(pyridyl)­pyrazine.^54^ 2-Bromopyridine (7.298 g, 46.19 mmol, 2.2 equiv) was dissolved in dry THF. This solution was cooled to −78 °C and ^*n*^BuLi (18.0 mL, 46.2 mmol, 2.2 equiv) was added to the solution dropwise. The mixture was stirred for 30 min. In a separate Schlenk flask, ZnCl_2_ (6.297 g, 46.19 mmol, 2.2 equiv) was dissolved in dry THF. The ZnCl_2_ solution was transferred to the 2-bromopyridine/^*n*^BuLi mixture via a cannula. This mixture was stirred for 2 h at room temperature. 4,6-Dibromopyrimidine (4.995 g, 20.99 mmol, 1.0 equiv) and Pd­(PPh_3_)_4_ (2.425 g, 2.099 mmol, 10 mol %) were dissolved in dry THF in a Schlenk flask, and the reaction mixture was transferred to this vessel. The mixture was stirred at 65 °C overnight. A 0.5 M EDTA solution (620 mL, pH ≈8) and saturated aqueous NaHCO_3_ (230 mL) were added to the mixture, and the dark brown solution was stirred for 15 min. The solution was extracted with DCM (3 × 100 mL); the organic layers were combined and washed with brine (70 mL) and dried with magnesium sulfate. The dark solution was filtered, and the solvent was removed *in vacuo*. The resulting dark solid was initially purified by sublimation (to remove PPh_3_). The light brown solid was further purified by eluting through silica gel with DCM (300 mL) and then 15% MeOH/DCM (200 mL). Solvent from the 15% MeOH/DCM fraction was removed *in vacuo*, giving the title product as a white solid. Additional crops of pure material can be obtained by recrystallization of the impure fractions from hot methanol (total yield: 1.207 g, 24.5%). Spectroscopic data was consistent with previous literature reports.^55^ ^15^N NMR (41 MHz, CD_3_CN): δ −70.31 (*N*_pyridine_), −97.26 (*N*_pyrimidine_) ppm.

### Synthesis of (bppm)­Rh-Cl

Complex **(bppm)­Rh-Cl** was synthesized via modification of a procedure outlined by Rao and coworkers for the preparation of the monorhodium complex of the bppd ligand.^31^ [Cp*RhCl_2_]_2_ (0.211 g, 0.341 mmol, 1 equiv), bppm (0.206 g, 0.879 mmol, 2.57 equiv), and NH_4_PF_6_ (0.140 g, 0.859 mmol, 2.5 equiv) were stirred in methanol (74 mL) at 70 °C for 4 h. After cooling the solution overnight, the product was collected via filtration through a fritted glass funnel, washed with cold methanol and diethyl ether, and air-dried. Yellow powder, 0.316 g (70.9%). Single crystals suitable for X-ray diffraction were obtained via vapor diffusion of Et_2_O into a solution of the title compound in CH_3_CN. The labeling scheme for spectroscopic assignments is shown in Figure 1. ^1^H NMR (500 MHz, CD_3_CN) δ 9.48 (d, ^5^ *J*_H,H_ = 1.2 Hz, 1 H, C9–*H*), 9.25 (d, ^5^ *J*_H,H_ = 1.2 Hz, 1 H, C7–*H*), 8.97 (ddd, ^3^ *J*_H,H_ = 5.4 Hz, ^4^ *J*_H,H_ = 1.5 Hz, ^5^ *J*_H,H_ = 0.9 Hz, 1H, C1–*H*), 8.84 (ddd, ^3^ *J*_H,H_ = 4.7 Hz, ^4^ *J*_H,H_ = 1.7 Hz, ^5^ *J*_H,H_ = 0.9 Hz, 1H, C14–*H*), 8.68 (ddd, ^3^ *J*_H,H_ = 8.1 Hz, ^4^ *J*_H,H_ = 1.3 Hz, ^5^ *J*_H,H_ = 0.9 Hz, 1H, C4–*H*), 8.60 (ddd, ^3^ *J*_H,H_ = 7.9 Hz, ^4^ *J*_H,H_ = 1.1 Hz, ^5^ *J*_H,H_ = 0.9 Hz, 1H, C11–*H*), 8.31 (ddd, ^3^ *J*_H,H_ = 8.1 Hz, ^3^ *J*_H,H_ = 7.7 Hz, ^4^ *J*_H,H_ = 1.5 Hz, 1H, C3–*H*), 8.07 (ddd, ^3^ *J*_H,H_ = 7.9 Hz, ^3^ *J*_H,H_ = 7.5 Hz, ^4^ *J*_H,H_ = 1.7 Hz, 1H, C12–*H*), 7.94 (ddd, ^3^ *J*_H,H_ = 7.7 Hz, ^3^ *J*_H,H_ = 5.4 Hz, ^4^ *J*_H,H_ = 1.3 Hz, 1H, C2–*H*), 7.64 (ddd, ^3^ *J*_H,H_ = 7.5 Hz, ^3^ *J*_H,H_ = 4.7 Hz, ^4^ *J*_H,H_ = 1.1 Hz, 1H, C13–*H*), 1.73 (s, 15 H, C16–*H*) ppm. ^13^C­{^1^H} NMR (125 MHz, CD_3_CN) δ 167.10 (C6 or C8), 162.54 (C8 or C6), 160.08 (C9), 153.52 (C5), 153.50 (C1), 152.53 (C10), 151.32 (C14), 141.57 (C3), 139.01 (C12), 131.25 (C2), 128.32 (C13), 126.67 (C4), 123.70 (C11), 115.84 (C7), 98.80 (d, ^1^ *J*_C,Rh_ = 8.1 Hz, C15), 9.28 (C16) ppm. ^31^P­{^1^H} NMR (202 MHz, CD_3_CN) δ −145.48 (hept, ^1^ *J*_P,F_ = 706.4 Hz, *P*F_6_^–^) ppm. ^19^F NMR (469 MHz, CD_3_CN) δ −73.76 (d, ^1^ *J*_F,P_ = 706.6 Hz, P*F*_6_^–^) ppm. ^15^N NMR (41 MHz, CD_3_CN): δ −69.14 (*N*_D_), −89.06 (*N*_C_), −131.25 (*N*_A_), −147.65 (*N*_B_) ppm. Elemental analysis: calculated for C_24_H_25_ClF_6_N_4_PRh (%): C 44.16, H 3.86, N 8.58; found: C 44.13, H 3.96, N 8.36.

**Figure 1:**
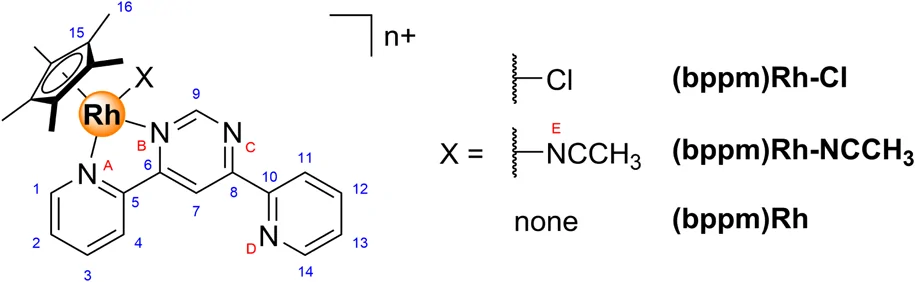
Labeling scheme for spectroscopic assignments for mononuclear bppm complexes.

### Synthesis of (bppm)­(Rh-Cl)_2_

Complex **(bppm)­(Rh-Cl)**_**2**_ was synthesized via modification of a procedure outlined by Kaim and coworkers for the preparation of the dirhodium complex of the bpym ligand.^33^ [Cp*RhCl_2_]_2_ (0.335 g, 0.542 mmol, 1.26 equiv) and AgPF_6_ (0.274 g, 1.08 mmol, 2.52 equiv) were stirred in acetone (67 mL) for 30 min. The colorless precipitate was separated from the orange solution via filtration through a fine-porosity fritted-glass funnel. A solution of bppm (0.101 g, 0.431 mmol, 1 equiv) in acetone was added to the orange filtrate, and the mixture was stirred overnight. The solution was concentrated *in vacuo* to 30 mL, and pentane was added to induce precipitation. The product was collected via filtration on a fritted-glass funnel, washed with diethyl ether, and air-dried. Yellow powder, 0.445 g (96.3%). Single crystals suitable for X-ray diffraction were obtained via vapor diffusion of Et_2_O into a solution of the title compound in CH_3_CN. The labeling scheme for spectroscopic assignments is shown in Figure 2. ^1^H NMR (500 MHz, CD_3_CN) δ 9.37 (s, 1H, C8–*H*), 9.22 (s, 1H, C7–*H*), 9.04 (dd, ^3^ *J*_H,H_ = 5.4 Hz, ^4^ *J*_H,H_ = 1.4 Hz, 2H, C1–*H*), 8.87 (d, ^3^ *J*_H,H_ = 8.0 Hz, 2H, C4–*H*), 8.45 (ddd, ^3^ *J*_H,H_ = 7.9 Hz, ^3^ *J*_H,H_ = 7.9 Hz, ^4^ *J*_H,H_ = 1.5 Hz, 2H, C3–*H*), 8.06 (dd, ^3^ *J*_H,H_ = 7.4 Hz, ^3^ *J*_H,H_ = 5.8 Hz, 2H, C2–*H*), 1.72 (s, 15H, C10–*H*) ppm. ^13^C­{^1^H} NMR (125 MHz, CD_3_CN) δ 164.11 (C8), 160.05, 154.31 (C1), 151.41, 142.20 (C3), 132.73 (C2), 132.65, 128.27 (C4), 99.56 (d, ^1^ *J*_C,Rh_ = 8.2 Hz, C9), 9.53 (C10) ppm. ^31^P­{^1^H} NMR (202 MHz, CD_3_CN) δ −145.49 (hept, ^1^ *J*_P,F_ = 706.3 Hz, *P*F_6_^–^) ppm. ^19^F NMR (469 MHz, CD_3_CN) δ −73.74 (d, ^1^ *J*_F,P_ = 706.5 Hz, P*F*_6_^–^) ppm. ^15^N NMR (41 MHz, CD_3_CN): δ −130.07 (*N*_A_), −138.28 (*N*_B_) ppm. Elemental analysis: calculated for C_38_H_50_Cl_2_F_12_N_4_OP_2_Rh_2_ (**(bppm)­(Rh-Cl)**_**2**_ **·Et**_**2**_ **O**, consistent with XRD characterization) (%): C 39.84, H 4.40, N 4.89; found: C 39.46, H 4.01, N 5.18.

**Figure 2:**
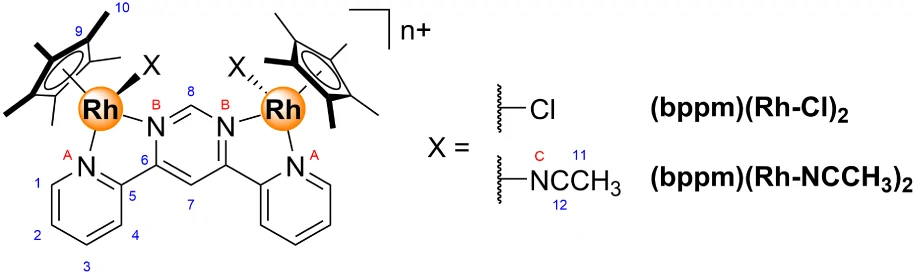
Labeling scheme for spectroscopic assignments for dinuclear bppm complexes.

### Synthesis of (bppm)­Rh-NCCH_3_

A solution of bppm (0.0253 g, 0.108 mmol, 1.5 equiv) in CH_3_CN was added to a stirring solution of [Cp*Rh­(NCCH_3_)_3_]­(PF_6_)_2_ (0.0464 g, 0.0713 mmol, 1 equiv) in CH_3_CN. The solution became darker orange immediately after mixing. After 30 min, the volume of the solution was reduced *in vacuo* and diethyl ether added to induce precipitation. The product was collected via filtration on a fritted-glass funnel and dried *in vacuo*. Yellow powder, 0.0443 g (77.4%). Single crystals suitable for X-ray diffraction were obtained by layering a solution of the title compound in CH_3_CN with Et_2_O. The labeling scheme for spectroscopic assignments is shown in Figure 1. ^1^H NMR (500 MHz, CD_3_CN) δ 9.54 (d, ^5^ *J*_H,H_ = 1.1 Hz, 1 H, C9–*H*), 9.34 (d, ^5^ *J*_H,H_ = 1.1 Hz, 1 H, C7–*H*), 9.03 (ddd, ^3^ *J*_H,H_ = 5.5 Hz, ^4^ *J*_H,H_ = 1.6 Hz, ^5^ *J*_H,H_ = 0.6 Hz, 1H, C1–*H*), 8.88 (ddd, ^3^ *J*_H,H_ = 4.7 Hz, ^4^ *J*_H,H_ = 1.8 Hz, ^5^ *J*_H,H_ = 0.9 Hz, 1H, C14–*H*), 8.78 (ddd, ^3^ *J*_H,H_ = 8.0 Hz, ^4^ *J*_H,H_ = 1.6 Hz, ^5^ *J*_H,H_ = 0.6 Hz, 1H, C4–*H*), 8.64 (ddd, ^3^ *J*_H,H_ = 7.9 Hz, ^4^ *J*_H,H_ = 1.3 Hz, ^5^ *J*_H,H_ = 0.9 Hz, 1H, C11–*H*), 8.43 (ddd, ^3^ *J*_H,H_ = 8.0 Hz, ^3^ *J*_H,H_ = 7.9 Hz, ^4^ *J*_H,H_ = 1.6 Hz, 1H, C3–*H*), 8.11 (ddd, ^3^ *J*_H,H_ = 7.9 Hz, ^3^ *J*_H,H_ = 7.8 Hz, ^4^ *J*_H,H_ = 1.8 Hz, 1H, C12–*H*), 8.05 (ddd, ^3^ *J*_H,H_ = 7.9 Hz, ^3^ *J*_H,H_ = 5.5 Hz, ^4^ *J*_H,H_ = 1.6 Hz, 1H, C2–*H*), 7.68 (ddd, ^3^ *J*_H,H_ = 7.8 Hz, ^3^ *J*_H,H_ = 4.7 Hz, ^4^ *J*_H,H_ = 1.3 Hz, 1H, C13–*H*), 1.76 (s, 15 H, C16–*H*) ppm; no signal corresponding to the Rh-bound NCCH_3_ ligand was observed in ^1^H NMR data. ^13^C­{^1^H} NMR (125 MHz, CD_3_CN) δ 168.14 (C8), 163.32 (C6), 160.18 (C9), 154.29 (C5), 153.92 (C1), 152.21 (C10), 151.53 (C14), 142.78 (C3), 139.22 (C12), 132.04 (C2), 128.68 (C13), 127.37 (C4), 123.94 (C11), 116.53 (C9), 101.70 (d, ^1^ *J*_C,Rh_ = 8.2 Hz, C15), 9.42 (C16) ppm. ^31^P­{^1^H} NMR (202 MHz, CD_3_CN) δ −145.50 (hept, ^1^ *J*_P,F_ = 706.4 Hz, *P*F_6_^–^) ppm. ^19^F NMR (469 MHz, CD_3_CN) δ −73.71 (d, ^1^ *J*_F,P_ = 706.7 Hz, P*F*_6_^–^) ppm. ^15^N NMR (41 MHz, CD_3_CN): δ −69.14 (*N*_D_), −87.89 (*N*_C_), −140.62 (*N*_A_), −157.03 (*N*_B_), −205.07 (*N*_E_) ppm. Elemental analysis: calculated for C_26_H_28_F_12_N_5_P_2_Rh (%): C 38.87, H 3.51, N 8.72; found: C 37.43, H 3.24, N 8.49; although these results are outside the range establishing analytical purity, they represent the best values obtained to date. Analysis by ^1^H NMR using 1,3,5-methoxybenzene as an internal standard indicated ≥ 95% purity (Figure S21).

### Synthesis of (bppm)­(Rh-NCCH_3_)_2_

A solution of bppm (0.0176 g, 0.0750 mmol, 1 equiv) in CH_3_CN was added to a stirring solution of [Cp*Rh­(NCCH_3_)_3_]­(PF_6_)_2_ (0.0977 g, 0.150 mmol, 2 equiv) in CH_3_CN. The solution became lighter yellow immediately after mixing. After 30 min, diethyl ether was added to induce precipitation. The product was collected via filtration on a fritted-glass funnel, washed with diethyl ether and heptane, and dried *in vacuo*. Yellow powder, 0.0889 g (86.4%). The labeling scheme for spectroscopic assignments is shown in Figure 2; two isomers (**a** and **b**) are obtained in a ∼2:1 **a**/**b** ratio. Isomer **a**: ^1^H NMR (400 MHz, CD_3_NO_2_) δ 9.69 (s, 1H, C8–*H*), 9.34–9.26 (m, 3H, C7–*H* and C1–*H*), 8.99–8.91 (m, 2H, C4–*H*), 8.63–8.55 (m, 2H, C3–*H*), 8.29–8.23 (m, 2H, C2–*H*), 2.17 (s, 6H, C11–*H*), 1.92 (s, 30H, C10–*H*) ppm. ^13^C­{^1^H} NMR (101 MHz, CD_3_NO_2_) δ 166.78 (C6), 159.41 (C8), 154.68 (C1), 152.13 (C5), 143.72 (C3), 134.30 (C2), 129.77 (C4), 128.24 (d, ^2^ *J*_C,Rh_ = 6.4 Hz, C12), 119.96 (C7), 103.23 (d, ^1^ *J*_C,Rh_ = 8.4 Hz, C9), 9.64 (C10), 3.46 (C11) ppm. Isomer **b**: 9.89 (s, 1H C8–*H*), 9.34–9.26 (m, 3H, C7–*H* and C1–*H*), 8.99–8.91 (m, 2H, C4–*H*), 8.63–8.55 (m, 2H, C3–*H*), 8.29–8.23 (m, 2H, C2–*H*), 2.27 (s, 6H, C11–*H*), 1.97 (s, 30H, C10–*H*) ppm. ^13^C­{^1^H} NMR (101 MHz, CD_3_NO_2_) δ 166.63 (C6), 160.43 (C8), 154.49 (C1), 152.46 (C5), 143.63 (C3), 134.30 (C2), 129.57 (C4), 127.95 (d, ^2^ *J*_C,Rh_ = 6.5 Hz, C12), 120.04 (C7), 103.10 (d, ^1^ *J*_C,Rh_ = 8.4 Hz, C9), 9.89 (C10), 3.46 (C11) ppm. ^31^P­{^1^H} NMR (162 MHz, CD_3_CN) δ −144.84 (hept, ^1^ *J*_P,F_ = 707.9 Hz, *P*F_6_^–^) ppm. ^19^F NMR (376 MHz, CD_3_CN) δ −73.25 (d, ^1^ *J*_F,P_ = 707.5 Hz, P*F*_6_^–^) ppm. ^15^N NMR (41 MHz, CD_3_CN) δ −145.31 (*N*_B_), −139.45 (*N*_A_), −207.90 (*N*_Ca_), −208.59 (*N*_Cb_) ppm. Elemental analysis: calculated for C_38_H_46_F_24_N_6_P_4_Rh_2_ (%): C 33.25, H 3.38, N 6.12; found: C 33.37, H 3.24, N 6.11.

### Synthesis of (bppm)­Rh

A solution of Cp_2_Co (0.107 g, 0.566 mmol, 2 equiv) in THF was added to a suspension of **(bppm)­Rh-Cl** (0.187 g, 0.285 mmol, 1 equiv) in THF. Over time, the yellow heterogeneous solution darkened in color and became more homogeneous. After 1.5 h, the solvent was removed *in vacuo*. The resulting residue was washed with hexanes and diethyl ether to remove any leftover Cp_2_Co; the residue was then extracted with toluene until the washings were colorless. Volatiles from the toluene fraction were removed *in vacuo* to give the product as a dark brown solid (0.0917 g, 68.1%). Single crystals were obtained by cooling a solution of the title compound in Et_2_O to −35 °C. The labeling scheme for spectroscopic assignments is shown in Figure 1. ^1^H NMR (500 MHz, CD_3_CN) δ 9.86 (d, ^5^ *J*_H,H_ = 1.3 Hz, 1 H, C9–*H*), 9.05 (d, ^5^ *J*_H,H_ = 1.3 Hz, 1 H, C7–*H*), 8.96 (d, ^1^ *J*_H,H_ = 6.4 Hz, 1H, C1–*H*), 8.90 (ddd, ^3^ *J*_H,H_ = 4.7 Hz, ^4^ *J*_H,H_ = 1.1 Hz, ^5^ *J*_H,H_ = 1.1 Hz, 1H, C14–*H*), 8.56 (ddd, ^3^ *J*_H,H_ = 4.6 Hz, ^4^ *J*_H,H_ = 1.8 Hz, ^5^ *J*_H,H_ = 0.9 Hz, 1H, C11–*H*), 7.59 (ddd, ^3^ *J*_H,H_ = 8.6 Hz, ^4^ *J*_H,H_ = 1.3 Hz, ^5^ *J*_H,H_ = 1.3 Hz, 1H, C4–*H*), 7.28 (ddd, ^3^ *J*_H,H_ = 8.0 Hz, ^3^ *J*_H,H_ = 7.4 Hz, ^4^ *J*_H,H_ = 2.0 Hz, 1H, C13–*H*), 6.79 (ddd, ^3^ *J*_H,H_ = 7.4 Hz, ^3^ *J*_H,H_ = 4.7 Hz, ^4^ *J*_H,H_ = 1.2 Hz, 1H, C12–*H*), 6.66 (ddd, ^3^ *J*_H,H_ = 8.5 Hz, ^3^ *J*_H,H_ = 6.6 Hz, ^4^ *J*_H,H_ = 1.4 Hz, 1H, C3–*H*), 7.38 (m, 1H, C2–*H*), 1.77 (s, 15 H, C16–*H*) ppm. ^13^C­{^1^H} NMR (125 MHz, CD_3_CN) δ 159.81, 157.25, 150.21 (C1), 149.61 (C11), 143.21, 142.87, 140.41, 139.69 (C13), 123.52 (C4), 123.44 (C3), 121.41 (C12), 119.30 (C14), 115.82 (C2), 114.96 (C7), 89.20 (d, ^1^ *J*_C,Rh_ = 7.2 Hz, C15), 9.56 (C16) ppm. ^15^N NMR (41 MHz, CD_3_CN): δ −135.35 (*N*_A_), −152.63 (*N*_B_) ppm; signals corresponding to *N*_C_ and *N*_D_ could not be observed. No satisfactory elemental analysis results could be obtained due to extreme sensitivity of the title compound to O_2_.

### Synthesis of (bpy)­Rh-Cl

Complex **(bpy)­Rh-Cl** was synthesized via modification of a literature procedure for related bipyridine complexes.^7^ To a suspension of [Cp*RhCl_2_]_2_ (0.100 g, 0.162 mmol, 1 equiv) in CH_3_CN was added a solution of AgPF_6_ (0.0827 g, 0.324 mmol, 2 equiv) in CH_3_CN. After 30 min, the solution was filtered through Celite to remove AgCl and a solution of bpy (0.0536 g, 0.343 mmol, 2.1 equiv) was added. After 30 min, the volume of the solution was reduced *in vacuo* and diethyl ether added to induce precipitation. The product was collected via filtration on a fritted-glass funnel and dried *in vacuo*. Yellow powder, 0.172 g (92.5%). Spectroscopic characterization was in agreement with previously published data.^12^ ^15^N NMR (41 MHz, CD_3_CN): δ −133.59 (Rh–*N*_pyridine_) ppm.

### Synthesis of (bpy)­Rh-NCCH_3_

Complex **(bpy)­Rh-NCCH**_**3**_ was synthesized via a literature procedure.^56^ Spectroscopic characterization was in agreement with previously published data. ^15^N NMR (41 MHz, CD_3_CN): δ −142.96 (Rh–*N*_pyridine_), −193.35 (Rh–*N*CCH_3_) ppm.

### Synthesis of (bpy)­Rh

Complex **(bpy)­Rh** was synthesized via a literature procedure.^57^ Spectroscopic characterization was in agreement with previously published data. ^15^N NMR (41 MHz, C_6_D_6_): δ −135.93 (Rh–*N*_pyridine_) ppm.

### Synthesis of (bpym)­Rh-Cl

Complex **(bpym)­Rh-Cl** was synthesized via a modification of the procedure reported by Štěpnička and Süss-Fink.^34^ [Cp*RhCl_2_]_2_ (0.151 g, 0.244 mmol, 1 equiv), bpym (0.0994 g, 0.628 mmol, 2.57 equiv), and NH_4_PF_6_ (0.100 g, 0.613 mmol, 2.5 equiv) were stirred in methanol (50 mL) at 70 °C for 4 h, during which time the mixture remained heterogeneous. After cooling, the solution was filtered through a fritted-glass funnel. The resulting yellow solid was washed with cold methanol and diethyl ether and dried *in vacuo*. Yellow powder, 0.187 g (67.5%). Spectroscopic characterization was in agreement with previously published data.^34^ Single crystals suitable for X-ray diffraction were obtained via vapor diffusion of Et_2_O into a solution of the title compound in CH_3_CN. ^15^N NMR (41 MHz, CD_3_CN): δ −79.69 (*N*_pyrimidine_), −141.79 (Rh–*N*_pyrimidine_) ppm.

### Synthesis of (bpym)­(Rh-Cl)_2_

Complex **(bpym)­(Rh-Cl)**_**2**_ was synthesized via a modification of the procedure reported by Kaim.^33^ [Cp*RhCl_2_]_2_ (0.167 g, 0.270 mmol, 1.4 equiv) was treated with AgPF_6_ (0.137 g, 0.542 mmol, 2.8 equiv) in acetone (30 mL) for 30 min, during which time a colorless precipitate formed. The precipitate was removed via filtration through a fine-porosity fritted glass funnel, and an acetone solution of bpym (0.0305 g, 0.193 mmol, 1 equiv) was added to the filtrate (total volume ∼100 mL). The mixture was stirred in the dark for 14 h. The volume of the solution was reduced to ∼10 mL *in vacuo*, which induced precipitation of a yellow solid. This solid was collected via filtration on a fritted-glass funnel, washed with cold acetone, and dried *in vacuo*. Yellow powder, 0.136 g (70.8%). Spectroscopic characterization was in agreement with previously published data; a diastereomeric ratio of ∼3:2 was typically observed.^33^ Single crystals suitable for X-ray diffraction were obtained via vapor diffusion of Et_2_O into a solution of the title compound in CH_3_NO_2_/CH_3_CN (3:1 v/v). ^15^N NMR (41 MHz, CD_3_CN): δ −133.59 (Rh–*N*_pyrimidine_, major isomer), −134.76 (Rh–*N*_pyrimidine_, minor isomer) ppm.

### Synthesis of (bpym)­Rh-NCCH_3_

A solution of bpym (0.0193 g, 0.122 mmol, 1.5 equiv) in CH_3_CN was added to a stirring solution of [Cp*Rh­(NCCH_3_)_3_]­(PF_6_)_2_ (0.0530 g, 0.0814 mmol, 1 equiv) in CH_3_CN. The solution became slightly darker orange immediately after mixing. After 30 min, the volume of the solution was removed *in vacuo* and diethyl ether added to induce precipitation. The product was collected via filtration on a fritted-glass funnel and dried *in vacuo*. Yellow powder, 0.0539 g (91.0%). Single crystals suitable for X-ray diffraction were obtained via vapor diffusion of Et_2_O into a solution of the title compound in CH_3_CN. The labeling scheme for spectroscopic assignments is shown in Figure 3. ^1^H NMR (400 MHz, CD_3_CN): δ 9.35 (dd, ^3^ *J*_H,H_ = 4.8 Hz, ^4^ *J*_H,H_ = 2.0 Hz, 2H C3–*H*), 9.19 (dd, ^3^ *J*_H,H_ = 5.7 Hz, ^4^ *J*_H,H_ = 2.0 Hz, 2H C1–*H*), 8.04 (dd, ^3^ *J*_H,H_ = 5.7 Hz, ^3^ *J*_H,H_ = 4.8 Hz, 2H C2–*H*), 1.73 (s, 15H, C6–*H*) ppm; no signal corresponding to the Rh-bound NCCH_3_ ligand was observed in ^1^H NMR data. ^13^C­{^1^H} NMR (101 MHz, CD_3_CN) δ 162.76 (C3), 161.20 (C4), 160.37 (C1), 127.03 (C2), 101.92 (d, ^1^ *J*_C,Rh_ = 8.5 Hz, C5), 9.38 (C6) ppm. ^31^P­{^1^H} NMR (162 MHz, CD_3_CN) δ −145.49 (hept, ^1^ *J*_P,F_ = 706.7 Hz, *P*F_6_^–^) ppm. ^19^F NMR (376 MHz, CD_3_CN) δ −73.69 (d, ^1^ *J*_F,P_ = 706.7 Hz, P*F*_6_^–^) ppm. ^15^N NMR (41 MHz, CD_3_CN): δ −78.51 (*N*_B_), −151.17 (*N*_A_), −206.24 (*N*_C_) ppm. Elemental analysis: calculated for C_20_H_24_F_12_N_5_P_2_Rh (%): C 33.03, H 3.33, N 9.63; found: C 33.08, H 3.36, N 9.49.

**Figure 3:**
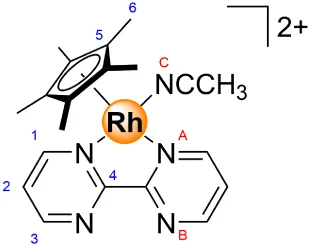
Numbering scheme for spectroscopic assignment for **(bpym)­Rh-NCCH_3_**.

## Results and Discussion

### Synthesis and Spectroscopic Characterization of Mono- and Dirhodium bppm Complexes

On the basis of literature precedent for [Cp*Rh] complexes supported by polyheterocyclic frameworks, we hypothesized that the 4,6-bis­(pyridyl)­pyrimidine platform (bppm, Scheme 1) would be well-suited to studies of both mono- and dirhodium complexes. The placement of the two metal-binding motifs was expected to prevent excessive steric clash (as in the bppz ligand), and the presence of a single shared heterocycle donor (vs two in the bpym framework) should also minimize destabilization of dirhodium species. Monometallic bppm complexes of Ru, Ni, Mo,^55^ and Cu^55,58^ as well as bimetallic Ru, Mo, Pd,^59^ Cu,^55,58,60^ Mn, Co, and Ni^60^ compounds have been reported, supporting the feasibility of this platform for studies of complexes with varying nuclearity.

**Scheme 1:**
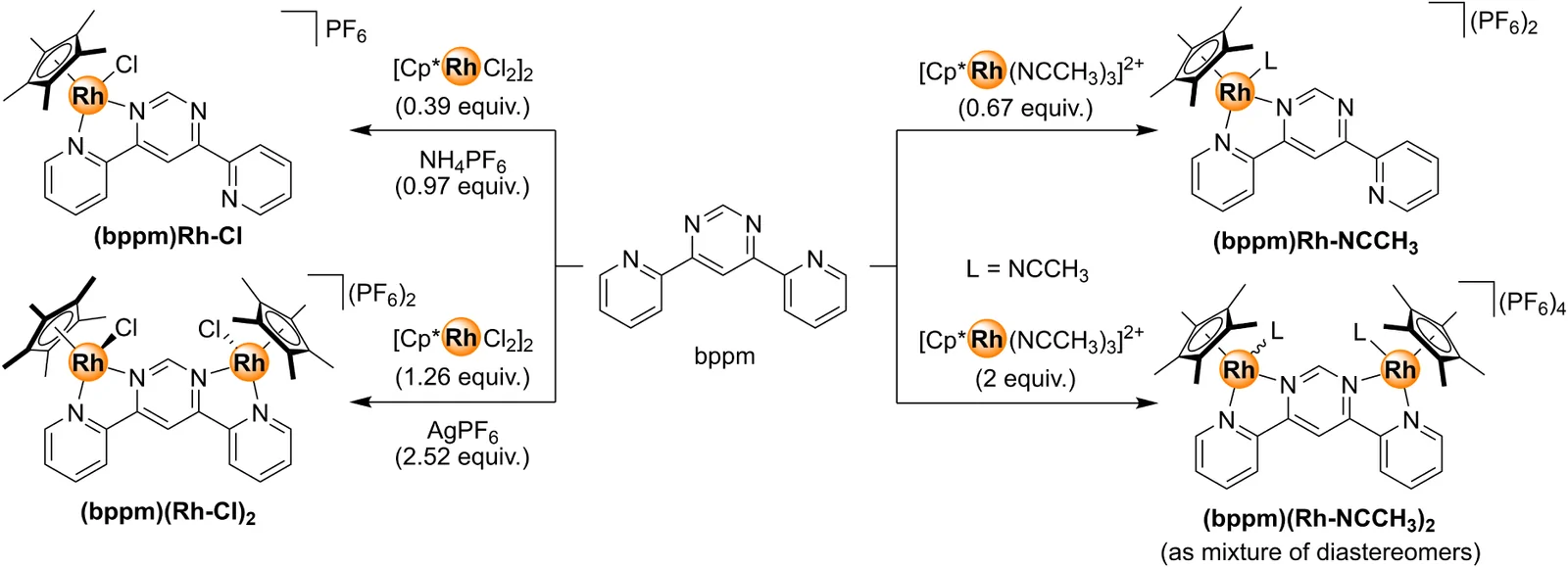
Synthesis of (bppm)­Rh_n_ Complexes with Cl^–^ and NCCH_3_ (n = 1, 2)

Mono- and dirhodium bppm complexes were synthesized from the well-known [Cp*RhCl_2_]_2_ precursor.^41^ As separation of mono- and dirhodium compounds proved challenging, synthetic procedures targeting mono- and dirhodium compounds were independently optimized to avoid the generation of complexes of both nuclearities in the same mixture. Reaction of [Cp*RhCl_2_]_2_ with NH_4_PF_6_ in the presence of an excess of bppm (∼1.3 equiv. per Rh) provided access to [Rh_1_] complex **(bppm)­Rh-Cl** (Scheme 1).^31^ A procedure similar to the synthesis of the dirhodium complex of bpym,^33^ utilizing a substoichiometric amount of ligand (0.40 equiv. bppm per Rh), instead provided the dirhodium complex **(bppm)­(Rh-Cl)**_**2**_.

As a free ligand, bppm possesses *C*_2v_ symmetry in solution, displaying 6 signals in its proton nuclear magnetic resonance (^1^H NMR) spectrum (Figure 4a). Addition of a [Cp*RhCl] moiety desymmetrizes the bppm framework, resulting in 10 aromatic resonances being observed (7–10 ppm, Figure 4b), in addition to the signal corresponding to the Cp* hydrogens (1.73 ppm). Addition of a second [Cp*RhCl] fragment results in resymmetrization of the bppm ligand; as in free bppm, only 6 resonances appear in the aromatic region of the ^1^H NMR spectrum for **(bppm)­(Rh-Cl)**_**2**_ (Figure 4c), with the resonance for the two equivalent Cp* groups being observed at 1.72 ppm.

**Figure 4:**
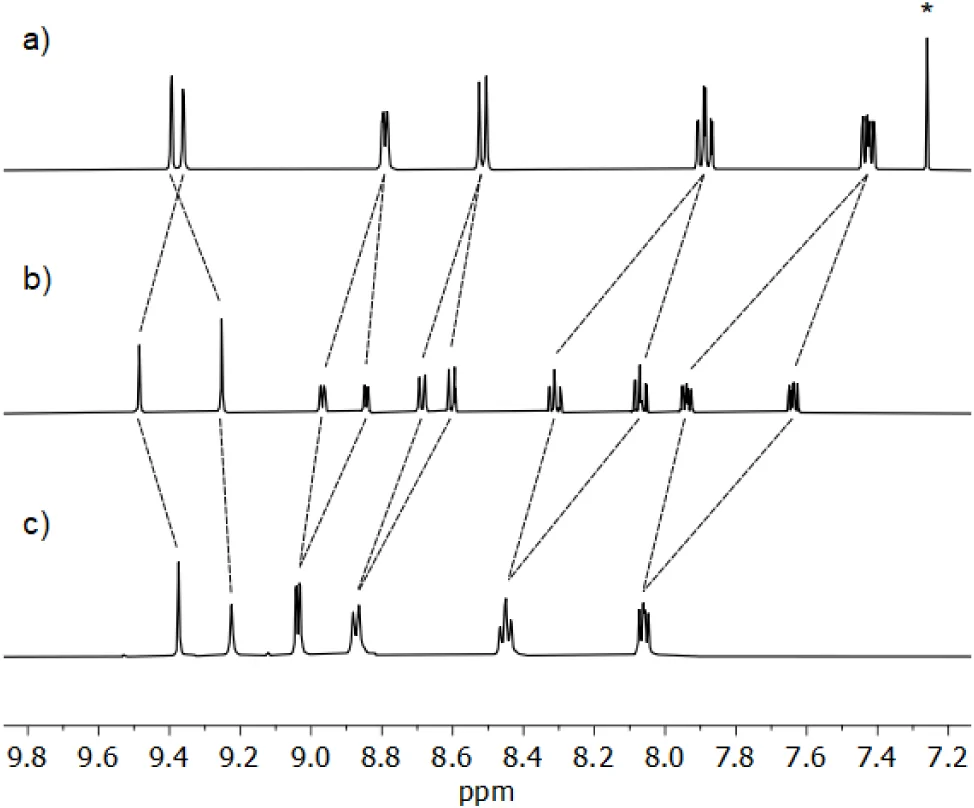
Partial ^1^H NMR spectra of (a) bppm (400 MHz, CDCl_3_), (b) **(bppm)­Rh-Cl** (500 MHz, CD_3_CN), and (c) **(bppm)­(Rh-Cl)_2_** (500 MHz, CD_3_CN) highlighting the changes in scaffold symmetry upon complexation. The peak marked with (*) corresponds to the solvent residual peak in CDCl_3_.

Two diastereomers are possible for **(bppm)­(Rh-Cl)**_**2**_, differing in the *syn* vs *anti* orientation of the Cp*/Cl^–^ ligands. Literature [Rh_2_] complexes supported by neutral polyheterocyclic ligands typically form both isomers in ratios between 1:1 and ∼3:1,^25,26,34,60^ while the anionic bpp ligand generates the *anti* diastereomer exclusively.^32^ NMR data for **(bppm)­(Rh-Cl)**_**2**_ suggests a significantly larger diastereomer ratio (>10:1) vs complexes of other neutral ligands (Figure S11). Neither variable-temperature (VT) NMR of **(bppm)­(Rh-Cl)**_**2**_ in CD_3_CN up to 70 °C nor prolonged heating in CD_3_CN at 70 °C resulted in substantial isomerization (Figures S65–S66), suggesting that the minor isomer is not readily accessible from the major diastereomer.

Chloride-free complexes were also targeted for comparative studies. While acetonitrile-bound complex **(bppm)­Rh-NCCH**_**3**_ could not be accessed via abstraction of chloride from **(bppm)­Rh-Cl**,^22,61,62^ metalation of free bppm with the tris­(acetonitrile) complex [Cp*Rh­(NCCH_3_)_3_]­(PF_6_)_2_ under conditions of excess ligand (1.5:1 bppm/Rh) yielded **(bppm)­Rh-NCCH**_**3**_ (Scheme 1).^56,63^ **(bppm)­Rh-NCCH**_**3**_ displays similar ^1^H NMR signatures to **(bppm)­Rh-Cl**, indicating asymmetry of the bppm framework consistent with the expected [Rh_1_] nuclearity. Attempts to access the corresponding dirhodium solvento complex **(bppm)­(Rh-NCCH**_**3**_)_**2**_ under similar metalation conditions resulted in mixtures of various species, including **(bppm)­Rh-NCCH**_**3**_ and [Cp*Rh­(NCCH_3_)_3_]^2+^.^42^ However, NMR data collected for this material in the less-coordinating solvent CD_3_NO_2_ did not indicate the presence of **(bppm)­Rh-NCCH**_**3**_ or [Cp*Rh­(NCCH_3_)_3_]^2+^ (Figure S54); instead, NMR features were consistent with the presence of the two diastereomers—*syn* and *anti*—of the putative **(bppm)­(Rh-NCCH**_**3**_)_**2**_ complex in a ∼2:1 ratio. Elemental analysis data for this material was consistent with the expected composition of **(bppm)­(Rh-NCCH**_**3**_)_**2**_. Addition of CD_3_CN (∼25% v/v) to CD_3_NO_2_ solutions of this material results in partial conversion to multiple species over the course of several hours (Figure S67), suggesting that reaction with CH_3_CN causes at least partial dissociation of the bppm scaffold. While addition of chloride ion (as [^n^Bu_4_N]­Cl) to **(bppm)­Rh-NCCH**_**3**_ results in conversion to **(bppm)­Rh-Cl** (Figure S68), addition of Cl^–^ to **(bppm)­(Rh-NCCH**_**3**_)_**2**_ does not cleanly generate **(bppm)­(Rh-Cl)**_**2**_ (Figure S69), though reactivity of **(bppm)­(Rh-Cl)**_**2**_ itself with excess Cl^–^ (Figure S70) complicates the analysis of these observations. Conversely, assignment of (**bppm)­(Rh-NCCH**_**3**_)_**2**_ was corroborated by the addition of [Cp*Rh­(NCCH_3_)_3_]^2+^ (3 × 0.33 equiv. aliquots) to isolated **(bppm)­Rh-NCCH**_**3**_, which results in the same NMR features as those observed for the isolated material (Figure S71).

### Structural Analysis of bppm Complexes

Characterization of **(bppm)­Rh-Cl** in the solid state via single-crystal X-ray diffraction (XRD) revealed the expected κ^2^ coordination mode of the bppm framework, together with the η^5^-Cp* ligand (Figure 5a,b). The coordination sphere of Rh was completed by a chloride ligand, with an outer-sphere PF_6_^–^ anion for charge balance. The free pyridyl group in **(bppm)­Rh-Cl** preferentially adopts a conformation that places the two uncoordinated N atoms (N3 and N4, Figure 5a) in a *trans* arrangement, as in free bpy,^64^ likely to relieve steric strain. Most structural metrics for the Rh site in **(bppm)­Rh-Cl** are in close alignment with those reported for Rh complexes featuring the bpy ligand (Table 1; Chart [Fig chart2]) and for monorhodium complexes supported by the multinucleating bppd and bppz platforms (Table S6, Chart S2). This observation indicates that the nature of the bound N-heterocycles and the presence of additional, uncoordinated pyridyl groups have minimal structural effects at Rh.

**2 chart2:**
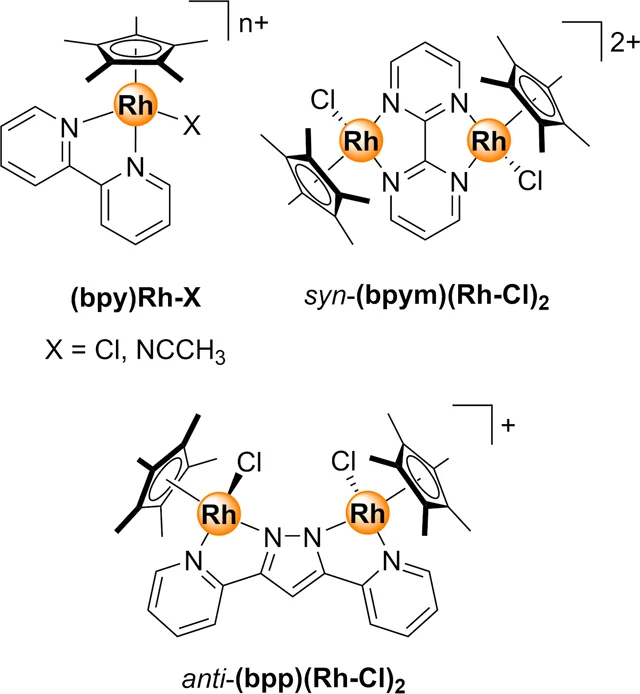
Reference Complexes for Analyses of bppm Compounds

**Table 1:** Selected Structural Metrics for Monorhodium bppm Complexes and Related bpy Compounds

Complex	(bppm)Rh-Cl	(bpy)Rh-Cl^a^			(bppm)Rh-NCCH_3_	(bpy)Rh-NCCH_3_ ^b^
CCDC #	2574181	1420047	675527	1303106	2574180	995532
bond distances (Å)						
**Rh–N1**	2.1164(14)	2.105(3)	2.1040(7)	2.100(5)	2.1145(17)	2.097(2), 2.1123(18)
**Rh–N2**	2.1167(14)	2.105(3)	2.1040(7)	2.100(5)	2.1184(17)	2.1035(17), 2.1090(18)
**Rh–X** ^c^	2.4050(4)	2.3972(14)	2.379(3)	2.385(3)	2.0781(18)	2.074(2), 2.066(2)
Rh–Cp* ^d^	1.7802(7)	1.784(3)	1.774(6)	1.776(4)	1.7813(9)	1.7804(12), 1.7832(10)
**d_C–C_(Rh)** ^e^	1.475(2)	1.461(7)	1.490(17)	1.481	1.481(3)	1.467(3), 1.466(3)
**d_C–C_(non-Rh)** ^f^	1.480(2)	-	-	-	1.480(3)	-
bond and plane angles (°)						
**N–Rh–N**	76.46(5)	76.68(16)	75.3(4)	76.6(3)	76.50(7)	77.09(7), 76.74(7)
∠(Cp*-[RhN_2_]) ^g^	57.59(6)	57.7(2)	59.0(5)	57.5(3)	53.59(7)	57.92(9), 55.75(9)
**∠(Rh–N≡C)**	-	-	-	-	177.02(17)	173.2(3), 176.94(17)
Reference	this work	^65^	^66^	^67^	this work	^56^

a Metrics for three complexes containing different counteranions (PF_6_^–^, ^–^OTf, and ClO_4_^–^, respectively).

b Metrics for two independent molecules in the asymmetric unit cell.

c Length of the bond between Rh and the monodentate ligand (Cl^–^ or NCCH_3_).

d Distance between Rh and the calculated centroid of the Cp* ligand.

e Length of the inter-ring C–C bond between Rh-bound heterocycles.

f Length of the inter-ring bond between the central heterocycle and the non-Rh-bound pyridyl fragment (absent in bpy complexes).

g Angle between the plane of the Cp* ligand and the calculated mean plane for the [RhN_2_] fragment.

**Figure 5:**
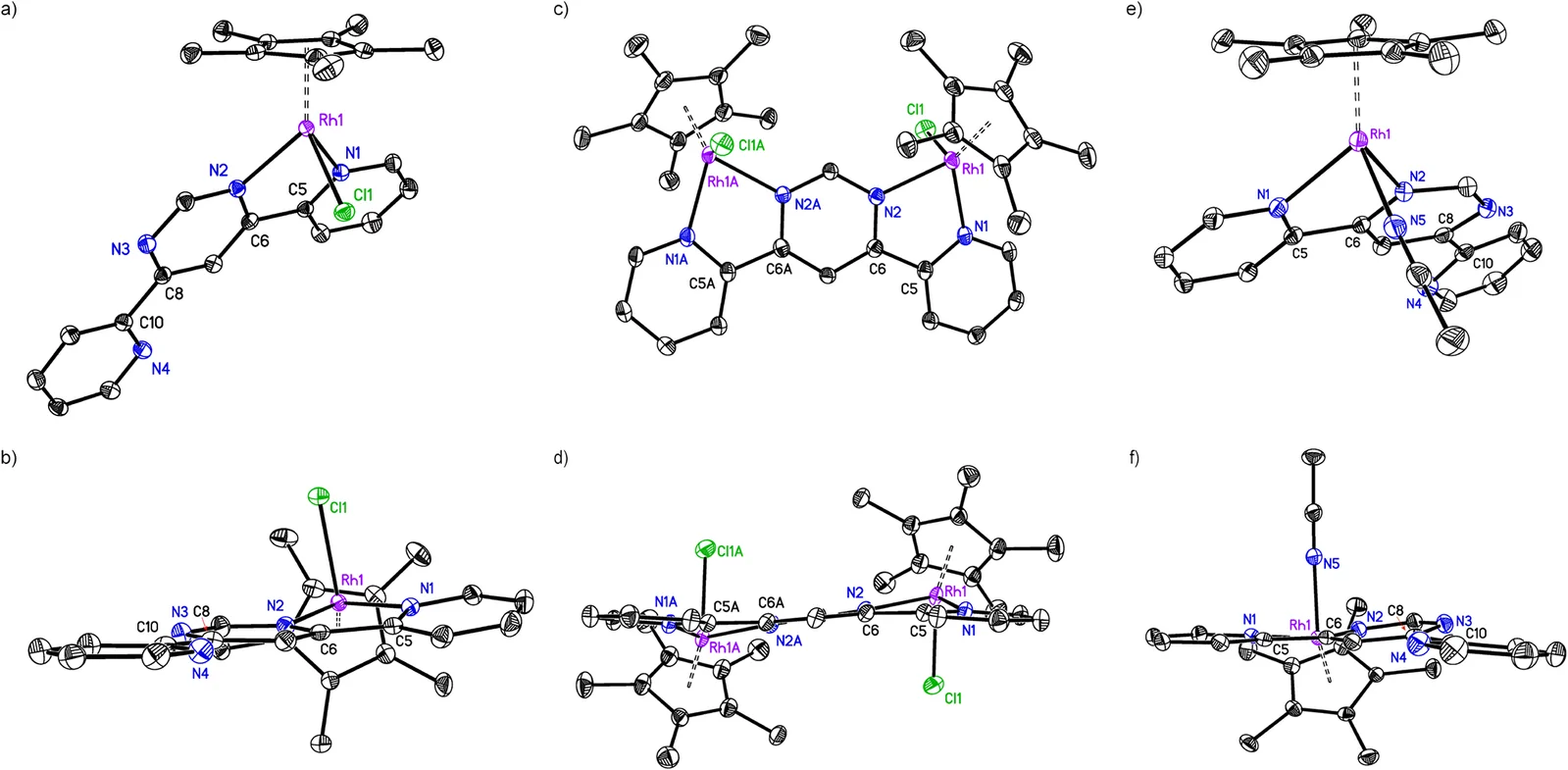
Solid-state structures (XRD) of **(bppm)­Rh-Cl** (a,b), **(bppm)­(Rh-Cl)_2_** (c,d), and **(bppm)­Rh-NCCH_3_** (e,f). H atoms and outer-sphere counteranions omitted for clarity. Displacement ellipsoids shown at the 50% probability level. Data collected at 100 K.

The solid-state structure of **(bppm)­Rh-NCCH**_**3**_ was also determined from XRD data (Figure 5e,f). **(bppm)­Rh-NCCH**_**3**_ adopts a nearly identical structure to its chloride analogue; the similarity in structural metrics indicates that exchange of Cl^–^ for a solvent ligand also has minimal effects on the geometry at Rh. Similarly, minor differences were observed between **(bpy)­Rh-Cl** and its solvento analogue, **(bpy)­Rh-NCCH**_**3**_ (Table 1).^56^ The NCCH_3_ ligand in **(bppm)­Rh-NCCH**_**3**_ is nearly linear (∠(Rh–N–C) = 177.02(17)°), similar to what was observed for **(bpy)­Rh-NCCH**_**3**_ (∼173°–177°; Table 1).

XRD analysis of **(bppm)­(Rh-Cl)**_**2**_ revealed Rh centers bound to both bidentate sites in the bppm framework, with Cp* and chloride ligands completing the coordination sphere at each Rh (Figure 5c,d). Critically, the Cp* and chloride ligands are located on opposite sides of the bppm scaffold in this structure, consistent with steric demands from the bulky Cp* groups favoring formation of the *anti* isomer. The structural metrics at the Rh sites (e.g., Rh–ligand distances, interligand angles) in **(bppm)­(Rh-Cl)**_**2**_ are similar to those of the other two complexes supported by polyheterocyclic ligands similar to bppm (bpym and bpp; Table 2).^32,34^ The bond and angle metrics in **(bppm)­(Rh-Cl)**_**2**_ are also in broad agreement with those of related monorhodium compounds (Table 1). While the Rh site metrics in bppm complexes are generally not significantly different from those in compounds of other mono- or dinucleating ligands, intriguing trends can be discerned in the extent of deformation of the polyheterocyclic frameworks as a function of their structural features, in the distortion of the 5-membered metallocycles, and in the correlation between these deformations and the observation of possible π–π interactions between ligand platforms (see the XRD section of the Supporting Information for further discussion).

**Table 2:** Selected Structural Metrics for (bppm)­(Rh-Cl)_2_ and Reference Dirhodium Complexes

Complex	(bppm)(Rh-Cl)_2_^a^	(bpym)(Rh-Cl)_2_ ^a^,^b^		(bpp)(Rh-Cl)_2_
CCDC#	2574175	635001	2574176	1401649
diastereomer	*anti*	*syn*	*syn*	*anti*
bond distances (Å)				
**Rh–N1**	2.121(2)	2.135(5)	2.147(4)	2.108(7), 2.162(7)
**Rh–N2**	2.091(2)	2.159(5)	2.139(4)	2.167(7), 2.134(8)
**Rh–Cl**	2.3805(7)	2.4053(16)	2.3991(14)	2.374(3), 2.399(3)
Rh–Cp* ^c^	1.7728(13)	1.781(3)	1.786(2)	1.779(4), 1.783(5)
**d_C–C_** ^d^	1.465(4)	1.47(1)	1.466(9)	1.438(13), 1.442(13)
bond and plane angles (°)				
**N–Rh–N**	76.20(8)	76.53(17)	77.10(15)	76.9(3), 77.1(3)
∠(Cp*-[RhN_2_]) ^e^	54.59(10)	59.9(2)	59.75(16)	51.1(3), 53.8(4)
Reference	this work	^34^	this work	^32^

a Complex on a crystallographic symmetry element, leading to identical metrics for the two halves of the complex.

b Metrics for two reported solventomorphs.

c Distance between Rh and the calculated centroid of the Cp­(*) ligand.

d Length of the inter-ring C–C bond between Rh-bound heterocycles.

e Angle between the plane of the Cp* ligand and the calculated mean plane for the [RhN_2_] fragment.

### Electron Transfer Studies of (bppm)­Rh-Cl and (bppm)­(Rh-Cl)_2_

Cyclic voltammetry (CV) studies of **(bppm)­Rh-Cl** in CH_3_CN revealed a two-electron reduction event at *E*_1/2_ = −1.05 V vs the ferrocenium/ferrocene couple (hereafter denoted as Fc^+/0^), corresponding to the (formal) Rh­(III/I) couple (Figure 6; Table 3). This potential is more positive than that for reduction of **(bpy)­Rh-Cl** (*E*_1/2_ = −1.21 V),^17^ suggesting that the additional N center in the pyrimidine ring and of the uncoordinated wingtip pyridyl group slightly lowers the electron density at Rh. An additional 1e^–^ process at −2.25 V vs Fc^+/0^ likely indicates reduction of the bppm framework to the bppm^•–^ radical anion. This reduction occurs at a more positive potential than in **(bpy)­Rh-Cl** (*E*_1/2_ = −2.61 V),^17^ consistent with the more extensive aromatic system in bppm resulting in lower-lying ligand π* orbitals which can be reduced at a more positive potential. The 2e^–^ Rh­(III/I) couples observed for these complexes are the result of an ECE’ electron-transfer mechanism: initial reduction of **(bppm)­Rh-Cl** (E) generates a substitutionally labile 19e^–^ Rh­(II) species, from which the monodentate ligand can rapidly dissociate (C). This process yields a 17e^–^ Rh­(II) intermediate whose reduction potential is more positive than that for the initial Rh­(III) complex (a process termed potential inversion); under voltammetric conditions, this complex is therefore immediately reduced to Rh­(I) (E’), giving rise to the observed 2e^–^ events in CV experiments.^17,68^

**Table 3:** Reduction Potentials^a^ for Select Monorhodium Complexes Supported by Bidentate Frameworks

	*E*_1/2_(Rh(III/I))		
Complex	X = Cl^–^	X = NCCH_3_	Δ*E*_1/2_ ^b^
**(bppm)Rh-X**	–1.05	–0.89	0.16
**(bpy)Rh-X**	–1.21	–1.04	0.17
**(bppz)Rh-X**	–1.18^c^	-	-
**(bpym)Rh-X**	–1.08	–0.91	0.17

a In V vs Fc^+/0^.

b Shift in midpoint potential between the chloride and solvento complex .

c From ref 36.

**Figure 6:**
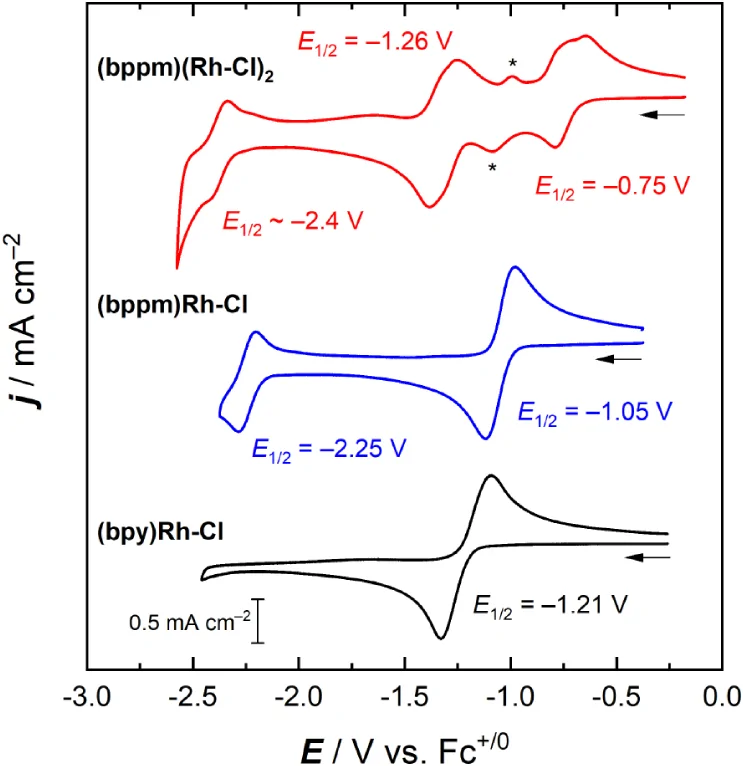
CV data (0.1 M [^n^Bu_4_N]­[PF_6_] in CH_3_CN, 100 mV/s, [Rh complex] = 2 mM) for complexes **(bpy)­Rh-Cl** (lower trace, black), **(bppm)­Rh-Cl** (middle trace, blue), and **(bppm)­(Rh-Cl)_2_** (upper trace, red). Events corresponding to **(bppm)­Rh-Cl** in the data for **(bppm)­(Rh-Cl)_2_** are marked with (*).

In CV data for **(bppm)­(Rh-Cl)**_**2**_, the most prominent features are two events at *E*_1/2_= −0.75 V and −1.26 V vs Fc^+/0^, respectively, and a smaller wave at ∼ −2.4 V (Figure 6; Table 4). An additional event at −1.04 V (similar to the potential for the Rh­(III/I) couple in **(bppm)­Rh-Cl**) is also consistently observed. Based on spectroelectrochemical data, reduction of [Rh_2_] complexes supported by the bptz and bpip ligands (Chart [Fig chart1]) was proposed by Kaim and coworkers to proceed via sequential reduction of each Rh center, with the first 2e^–^ process involving initial reduction of the highly electron-poor ligand framework, followed by Rh-based reduction and Cl^–^ dissociation to generate a formally Rh­(I) center via an EEC mechanism; the second Rh site then undergoes reduction via the typical ECE process.^25,26^ The events at −0.75 V and −1.26 V in CV data for **(bppm)­(Rh-Cl)_2_** are consistent with a similar reductive sequence and can be assigned as the formal Rh­(III,III)/Rh­(III,I) and Rh­(III,I)/Rh­(I,I) couples, respectively. The separation between the two reductions at each Rh center in **(bppm)­(Rh-Cl)_2_** is significantly smaller than in the complexes reported by Kaim,^25,26^ with only partial resolution of the distinct 1e^–^ processes in **(bppm)­(Rh-Cl)**_**2**_. Thus, while the possibility of a net EEC mechanism in the reduction of the first Rh site in **(bppm)­(Rh-Cl)_2_** cannot be ruled out, the data is also consistent with an ECE mechanism for reduction of each [Cp*Rh] unit similar to mononuclear analogues.

**Table 4:** Reduction Potentials^a^ for Select (L–L)­(Rh-Cl)_2_ Complexes Supported by Binucleating Ligands

Complex	*E*_1/2_, Rh(III,III)/Rh(III,I)	*E*_1/2_, Rh(III,I)/Rh(I,I)	*E*_1/2_, L/L^•–^	ref.
**(bppm)(Rh-Cl)**_**2**_	–0.75	–1.26	–2.4	this work
**(bppz)(Rh-Cl)**_**2**_	–0.85	–1.20	–2.29	^36^
**(bpip)(Rh-Cl)**_**2**_ ^b^,^c^	*E*_pc_ = −0.50, −0.76	–1.14, −1.36	–2.41	^26^
	*E*_pa_ = −0.43			
**(bptz)(Rh-Cl)**_**2**_ ^d^	*E*_pc_ = −0.03, −1.06	–1.20	–1.98	^25^
	*E*_pa_ = +0.04, −0.46			
**(bpym)(Rh-Cl)**_**2**_ ^e^	–0.63	*E*_pc_ = −1.27	-	^34^
**(bpym)(Rh-Cl)**_**2**_	–0.74	–1.26	–2.6	^33^
**(bpym)(Rh-Cl)**_**2**_	–0.72	–1.27	-	this work
**(bpp)(Rh-Cl)**_**2**_	–1.29	–1.77	–2.07	^32^

a In V vs Fc^+/0^ Based on data collected in CH_3_CN.

b Distinct cathodic peaks but a single anodic peak observed for the Rh­(III,III)/Rh­(III,I) couple.

c Distinct 1e^–^ events observed for the Rh­(III,I)/Rh­(I,I) couple.

d Distinct cathodic and anodic peaks observed for the Rh­(III,III)/Rh­(III,I) couple (initial reduction is ligand-based).

e In *N*,*N*-dimethylformamide (DMF).

### Chemical Reduction of (bppm)­Rh-Cl

To confirm assignment of the reduction of **(bppm)­Rh-Cl** as producing the corresponding Rh­(I) complex **(bppm)­Rh**, the synthesis of this putative Rh­(I) compound was attempted. In place of sodium amalgam (Na­(Hg)),^7,57,69^ whose reduction potential (*E* = −2.36 V)^70^ is incompatible with the positively shifted ligand-based reduction in **(bppm)­Rh-Cl**, this compound was treated with 2 equiv of the milder reductant cobaltocene (Cp_2_Co, *E*_1/2_ = −1.31 V; Scheme 2)^70^ in THF, resulting in a dramatic change from a yellow heterogeneous mixture to a nearly homogeneous, dark-blue solution. The dark solid obtained following workup was more soluble in nonpolar solvents than its precursor, consistent with a neutrally charged species. ^1^H NMR analysis of this material in C_6_D_6_ revealed an analogous pattern to that observed for **(bppm)­Rh-Cl**, with a desymmetrized bppm framework accompanied by a signal at 1.77 ppm corresponding to the Cp* protons, supporting retention of the single [Cp*Rh] fragment.

**Scheme 2:**
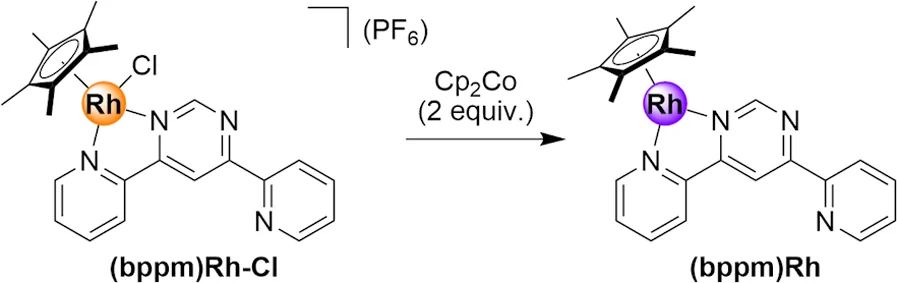
Chemical Reduction of Complex (bppm)­Rh-Cl

XRD data collected for a single crystal of this material confirmed its identification as the desired Rh­(I) complex. **(bppm)­Rh** crystallizes in the *Pna2*_*1*_ space group, with two independent molecules in the asymmetric unit cell; the Rh center is supported by two ligands—the η^5^-Cp* ligand and the κ^2^-bound bppm framework. The geometry at Rh is *pseudo*-trigonal planar, with a near-orthogonal orientation of the bppm and Cp* ligands (∼82°), similar to what has been observed for the corresponding bpy complex, **(bpy)­Rh**, and its cyclopentadienyl analogue, **CpRh­(bpy)** (Table 5). Likewise, the Rh–N and Rh–Cp* distances and the N–Rh–N angle are similar across these three complexes.

**Table 5:** Selected Structural Metrics for (bppm)­Rh and Reference Rh­(I) Complexes

Complex	(bppm)Rh^a^	(bpy)Rh^b^		CpRh(bpy)
CCDC#	2574177	959935	995550	1532782
bond distances (Å)				
**Rh–N1**	1.987(4), 2.004(4)	2.003(3)	2.000(3)	1.993(2)
**Rh–N2**	2.030(4), 2.024(4)	1.997(3)	2.005(2)	1.990(3)
Rh–Cp(*) ^c^	1.836(3), 1.836(3)	1.8355(15)	1.8472(12)	1.868(2)
**d_C–C_(Rh)** ^d^	1.424(7), 1.417(7)	1.424(5)	1.422(4)	1.441(3)
**d_C–C_(non-Rh)** ^e^	1.481(7), 1.472(7)	-	-	-
bond and plane angles (°)				
**N–Rh–N**	77.93(17), 77.81(18)	78.21(11)	78.16(10)	78.76(8)
∠(Cp(*)-[RhN_2_]) ^f^	82.316(5), 81.639(5)	84.86(11)	86.32(9)	83.24(10)
Reference	this work	^57^	^56^	^69^

a Values for two independent molecules in the asymmetric unit cell shown.

b Metrics for two distinct reported structures.

c Distance between Rh and the calculated centroid of the Cp­(*) ligand.

d Length of the inter-ring C–C bond between Rh-bound heterocycles.

e Length of the inter-ring bond between the central heterocycle and the non-Rh-bound pyridyl fragment (absent in bpy complexes).

f Angle between the plane of the Cp­(*) ligand and the calculated mean plane for the [RhN_2_] fragment.

The Rh–N bond lengths in **(bppm)­Rh** are contracted by ∼0.1 Å vs in **(bppm)­Rh-Cl**; this is consistent with a stronger interaction resulting from π-backdonation by the reduced Rh center into the bppm π* MOs.^17^ Electron delocalization onto the polypyridyl framework also results in contraction of the bond between the two Rh-bound heterocycles. The inter-ring C–C bond in the Rh-bound side of the bppm ligand in **(bppm)­Rh** (C5–C6, Figure 7) is significantly shorter (1.424(7), 1.417(7) Å in the two independent molecules) than the corresponding bond on the non-Rh-bound side of the molecule (C8–C10; 1.481(7), 1.472(7) Å) as well as the inter-ring C–C bonds in **(bppm)­Rh-Cl** (1.475(2) and 1.480(2) Å) and **(bppm)­(Rh-Cl)**_**2**_ (1.465(4) Å). Similar changes are observed for **(bpy)­Rh** and **CpRh­(bpy)** vs their Rh­(III) analogues (Tables 5 and 1).^56,57,69^ Contraction of the inter-ring bond in the bidentate fragment is consistent with delocalization of electron density into the LUMO of the polypyridyl framework, which typically possesses bonding character across this C–C bond, and has thus been used to assess the extent of ligand participation in stabilization of reduced metal centers via π-backdonation in bpy complexes.^17,69^ In **(bppm)­Rh**, the absence of contraction of the inter-ring bond between the central pyrimidine ring and the uncoordinated pyridyl group (C8–C10, Figure 7) suggests that delocalization of e^–^ density may be limited to the portion of the bppm framework that is directly interacting with Rh, rather than extending to the entire platform.

**Figure 7:**
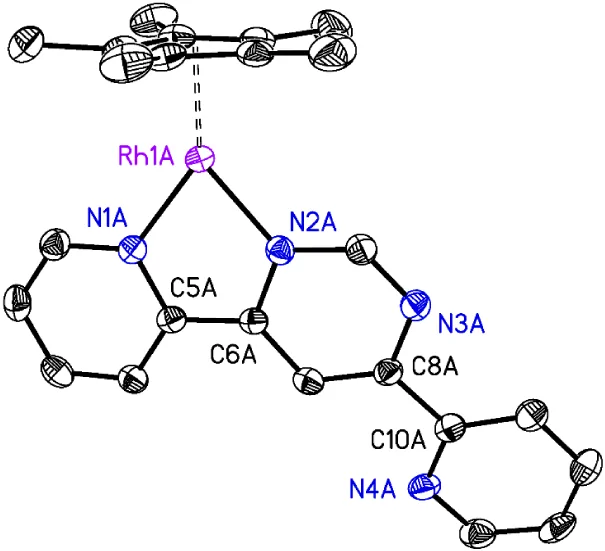
Solid-state structure (XRD) of complex **(bppm)­Rh**. Hydrogen atoms omitted for clarity. Displacement ellipsoids shown at the 50% probability level. One of two independent molecules in the asymmetric unit cell shown for clarity. Data collected at 100 K.

In the electronic absorption spectrum of **(bppm)­Rh** (Figure 8), the most intense band in the visible region, centered at 644 nm (ε_644_ = 1.6 × 10^4^ M^–1^ cm^–1^), is red-shifted vs the corresponding feature in **(bpy)­Rh** (516 nm).^56,69^ This shift results in the observed difference in apparent solution color (blue for **(bppm)­Rh** vs purple for **(bpy)­Rh**) due to the increased absorption of **(bppm)­Rh** in the red region of the visible spectrum. This band has been assigned to a π → π* transition between orbitals that are delocalized between the metal center and the ligand;^17,35^ the substantial red shift in this feature between **(bppm)­Rh** and **(bpy)­Rh** (>100 nm) is somewhat surprising given the lack of evidence for delocalization of electron density in the portion of the bppm platform that is not bound to Rh inferred from structural data. However, similar red shifts have been reported by Kaim for the analogous feature in the mixed-valence [Rh­(I)­Rh­(III)] complexes supported by the bptz and bpip frameworks.^25,26^

**Figure 8:**
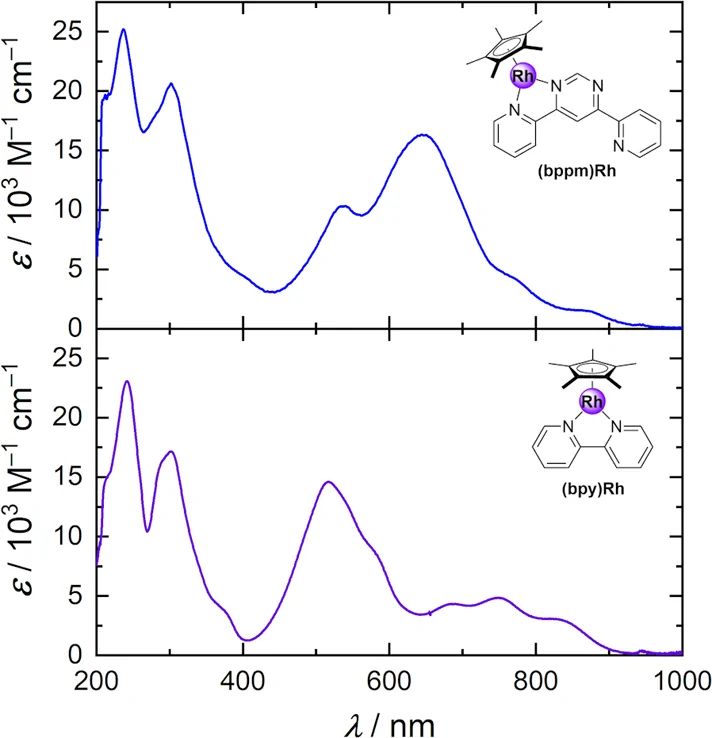
Electronic absorption spectra in THF of **(bppm)­Rh** (upper panel, blue) and **(bpy)­Rh** (lower panel, purple).

### Electrocatalytic Studies with bppm Complexes

Having established the reductive profile of **(bppm)­Rh-Cl** and **(bppm)­(Rh-Cl)**_**2**_, we sought to determine the ability of these complexes to participate in H_2_ evolution catalysis and evaluate the possible synergy between a Rh center and proton-responsive motif in its close proximity (as in **(bppm)­Rh-Cl**) or between adjacent Rh centers (**(bppm)­(Rh-Cl)**_**2**_) in catalysis. CV experiments were carried out under typical catalytic conditions for analogous bpy complexes using anilinium triflate ([C_6_H_5_NH_3_]­[OTf], p*K*_a_ = 10.6 in CH_3_CN^71^) as a proton source (1:1 [C_6_H_5_NH_3_]^+^/[C_6_H_5_NH_2_], 0–5 equiv.; [Rh] = 2.0 mM, *P*(H_2_) = 1 atm; *E*°(H^+^/H_2_) = −0.66 V vs Fc^+/0^).^72^ Under these conditions, **(bpy)­Rh-Cl** displays catalytic current enhancement at the potential corresponding to the Rh­(III/I) couple, consistent with H_2_ evolution (Figure 9, upper panel).^7^ For **(bppm)­Rh-Cl**, however, only a minimal current increase was observed at the corresponding potential (Figure 9, middle panel), suggesting that **(bppm)­Rh-Cl** is not a competent H_2_ evolution catalyst under these conditions. For dirhodium complex **(bppm)­(Rh-Cl)**_**2**_, no catalytic current was observed at the first Rh­(III/I) couple (−0.75 V; Figure 9, lower panel), consistent with the minimal overpotential (η = 0.09 V) for H_2_ evolution at this potential.^72^ More substantial current enhancement was observed near the second Rh­(III/I) couple (*E* = −1.26 V), though still significantly lower than in the parent bpy complex. Overall, therefore, neither bppm-supported complex was a particularly competent electrocatalyst compared to literature bpy compounds; crucially, the observation of potentially complicating ligand exchange processes (vide infra) may plausibly explain the limited catalytic competence of **(bppm)­Rh-Cl** and **(bppm)­(Rh-Cl)**_**2**_.

**Figure 9:**
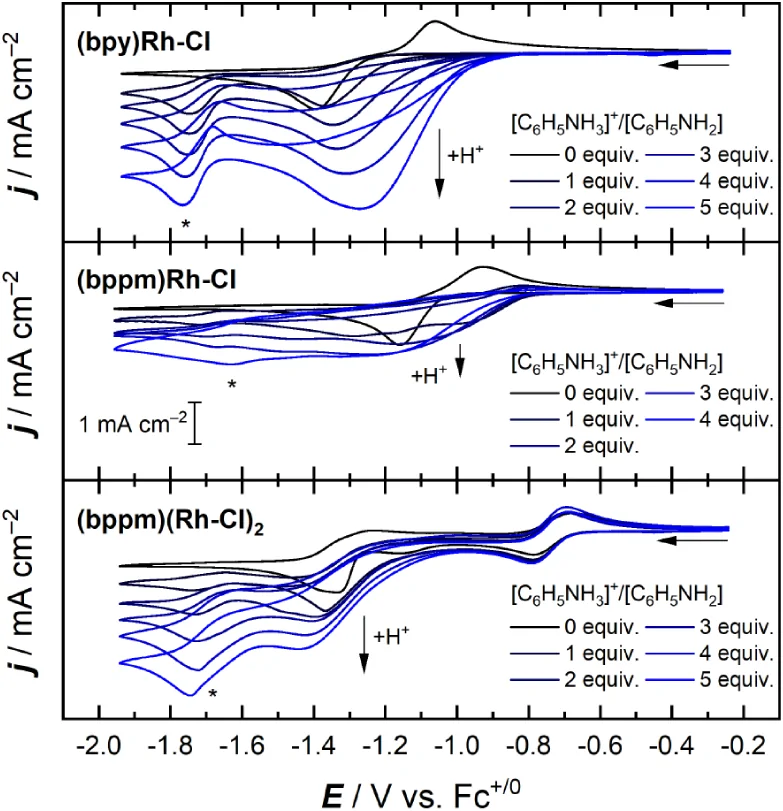
CV data (0.1 M [^n^Bu_4_N]­[PF_6_] in CH_3_CN, 100 mV/s, [Rh complex] = 2 mM, *P*(H_2_) = 1 atm) for **(bpy)­Rh-Cl**, **(bppm)­Rh-Cl**, and **(bppm)­(Rh-Cl)_2_** in the presence of added anilinium acid and conjugate base. Peaks marked with (*) correspond to possible [Cp*H] species generated under catalytic conditions.

Under catalytic conditions, new redox events were observed for **(bppm)­Rh-Cl**, **(bppm)­(Rh-Cl)**_**2**_, and **(bpy)­Rh-Cl** at potentials below −1.6 V vs Fc^+/0^ (marked with (*) in Figure 9), more negative than the corresponding Rh­(III/I) couples. In the data for **(bpy)­Rh-Cl**, this event is observed at a nearly identical potential to that reported for the isolated (Cp*H)­Rh­(bpy)Br complex (*E*_1/2_ = −1.73 V),^19^ suggesting formation of a similar protonated intermediate under electrocatalytic conditions. Analogous behavior was reported for the complex supported by the electron-deficient 4,4’-bis­(trifluoromethyl)­bipyridine (^CF3^bpy) ligand in the presence of the weaker acid triethylammonium triflate.^7^ The occurrence of reduction events at potentials more negative than the respective Rh­(III/I) couples in **(bppm)­Rh-Cl** and **(bppm)­(Rh-Cl)**_**2**_ in the presence of acid suggests possible participation of [Cp*H]-type complexes in their reactivity, though the formation of other electroactive fragments cannot be ruled out at this stage.

### Electron-Transfer Studies of Solvento Complexes

CV data for **(bppm)­Rh-NCCH**_**3**_ revealed a 2e^–^ reduction analogous to that observed for **(bppm)­Rh-Cl** at a potential of −0.89 V vs Fc^+/0^ (Figure 10). This potential is ∼0.15 V more positive than that for the chloride complex, likely due to the dicationic nature of **(bppm)­Rh-NCCH**_**3**_ vs monocationic **(bppm)­Rh-Cl**. Similar shifts (0.15–0.20 V) have previously been observed for rhodium solvento vs chloride complexes of mononucleating N-/P-donor bidentate ligands.^22,73,74^

**Figure 10:**
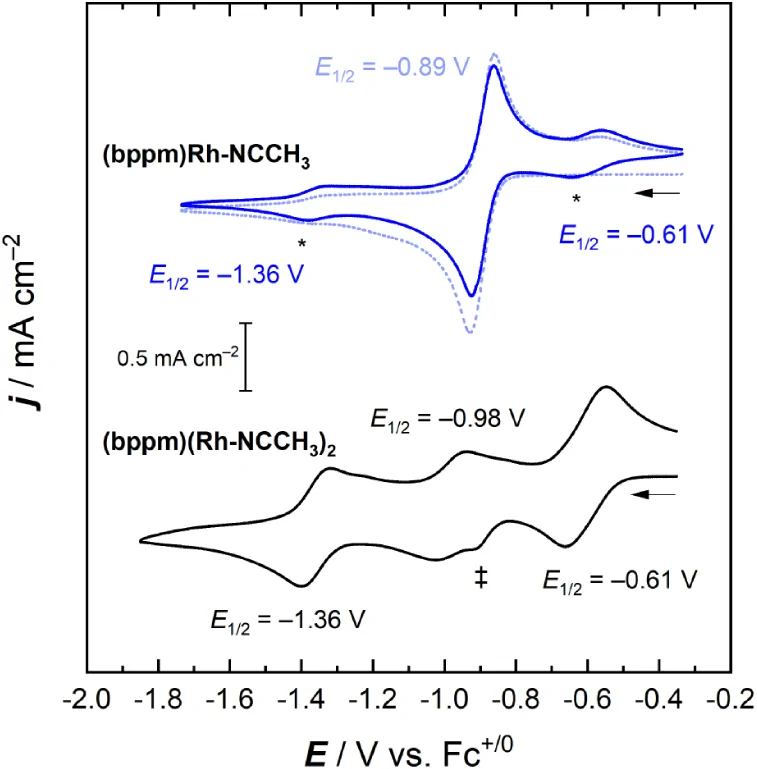
CV data (0.1 M [^n^Bu_4_N]­[PF_6_] in CH_3_CN, 100 mV/s, [Rh complex] = 2 mM) for complexes **(bppm)­(Rh-NCCH_3_**)**_2_** (lower trace, black) and **(bppm)­Rh-NCCH_3_** (upper trace, blue). Events corresponding to **(bppm)­(Rh-NCCH_3_**)**_2_** formed by ET-induced ligand exchange in **(bppm)­Rh-NCCH_3_** are marked with (*). The cathodic event suggesting the presence of **(bppm)­Rh-NCCH_3_** in the data for **(bppm)­(Rh-NCCH_3_**)**_2_** is marked with (‡).

Further CV scans revealed several additional ET events. During the first cathodic sweep, a smaller event was observed at a more negative potential (*E*_1/2_ = −1.36 V). In the second voltammetric cycle, a new redox event was observed at *E*_1/2_ = −0.55 V—more positive than the reduction of **(bppm)­Rh-NCCH**_**3**_—which was absent in the first cathodic scan. The changes in the CV profile in sequential scans indicate the generation of a new electroactive byproduct. The potentials for the new ET events (more positive and more negative, respectively, than the Rh­(III/I) couple for **(bppm)­Rh-NCCH**_**3**_) and the separation between these events (∼0.7 V, similar to the separation between the Rh­(III,III/III,I) and Rh­(III,I/I,I) couples in **(bppm)­(Rh-Cl)**_**2**_) suggest the formation of a dirhodium complex from the reduction of **(bppm)­Rh-NCCH**_**3**_.

To validate this hypothesis, we investigated the ET profile of **(bppm)­(Rh-NCCH**_**3**_)_**2**_. Due to incompatibility of CH_3_NO_2_ or CH_2_Cl_2_ with CV studies of compounds in this family due to their reactivity with Rh­(I) species,^69^ and to the insolubility of this complex in THF or 1,2-difluorobenzene (DFB), CV data was collected on freshly prepared solutions of **(bppm)­(Rh-NCCH**_**3**_)_**2**_ in CH_3_CN. These analyses revealed several reduction events in a similar potential window as that investigated for **(bppm)­Rh-NCCH**_**3**_. Two of these events occurred at potentials that are virtually identical to those for the byproduct generated following ET in **(bppm)­Rh-NCCH**_**3**_ (*E*_1/2_ = −0.61 and −1.36 V, respectively; Figure 10), supporting the generation of **(bppm)­(Rh-NCCH**_**3**_)_**2**_ from its monorhodium analogue. A broad wave is also observed at *E*_1/2_ = −0.98 V; this is not consistent with either **(bppm)­Rh-NCCH**_**3**_ or with reduction of [Cp*Rh­(NCCH_3_)_3_]^2+^ (*E*_1/2_ = −0.86 V, Figure S89). While this event may arise from another unidentified species, the possibility that the reduction of the second Rh center in **(bppm)­(Rh-NCCH**_**3**_)_**2**_ occurs via sequential 1e^–^ steps rather than the typical 2e^–^ ECE mechanism cannot be ruled out.^25,26^ A small peak near −0.9 V is, however, consistent with formation of at least some **(bppm)­Rh-NCCH**_**3**_ (cf. *E*_pc_(Rh­(III/I) = −0.93 V; Figure 10), consistent with spectroscopic data.

The observation of the two events that arise from ET-induced reactivity of **(bppm)­Rh-NCCH**_**3**_ in CV data for **(bppm)­(Rh-NCCH**_**3**_)_**2**_ supports the generation of the dirhodium complex from the reduction of **(bppm)­Rh-NCCH**_**3**_. Based on the known labilization of monodentate ligands in these systems upon reduction,^1,3,17^ it can be hypothesized that reduction of **(bppm)­Rh-NCCH**_**3**_ to Rh­(II) can result in dissociation of the κ^2^-N_2_ ligand from the [Cp*Rh] moiety, likely concurrently with binding of at least one additional CH_3_CN molecule to this Rh center (Scheme 3, red pathway). Under CV conditions, this [Cp*Rh] fragment may bind to the [N_2_] donor motif of a *different* bppm framework that already contains a [Cp*Rh] unit, resulting in the generation of a dirhodium species that can thus be observed in subsequent voltammetric scans. The role of the solvent in facilitating this reactivity was supported by CV analysis of **(bppm)­Rh-NCCH**_**3**_ in the noncoordinating solvent DFB. While an irreversible reduction was observed under these conditions, no new ET events at more negative potentials than the initial reduction were observed (Figure S86), suggesting that **(bppm)­(Rh-NCCH**_**3**_)_**2**_ is not generated in DFB, with the observed irreversibility instead likely due to reaction with the fluorinated solvent.^57^

**Scheme 3:**
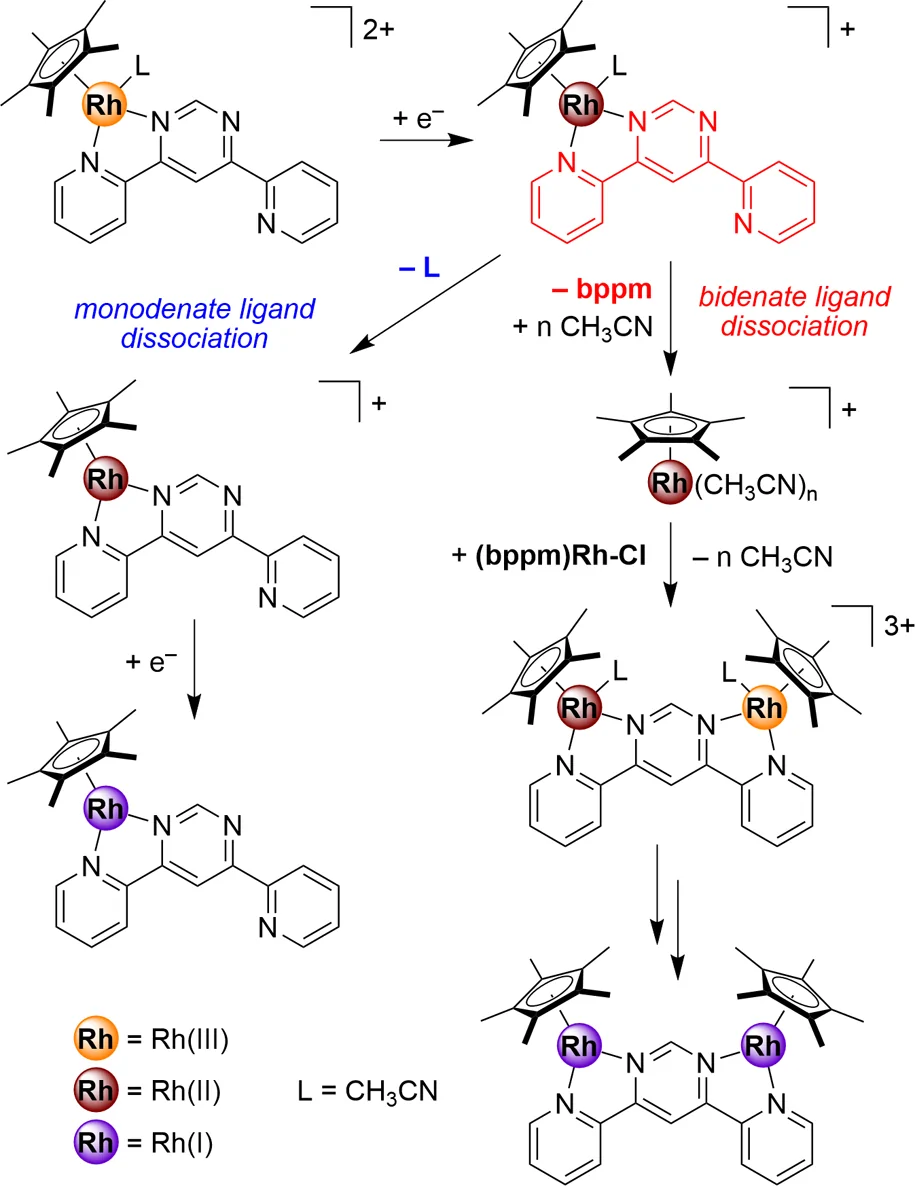
Proposed Ligand Dissociation Pathways from (bppm)­Rh-NCCH_3_ upon Reduction

Dissociation of bppm in **(bppm)­Rh-NCCH**_**3**_ appears to be competitive with that of the monodentate ligand (Scheme 3, blue pathway) even in the presence of a very large excess of CH_3_CN solvent ([CH_3_CN] ≈ 19.5 M vs [Rh] = 2 mM in CV experiments)—despite the limited lifetime of the Rh­(II) intermediate generated under CV conditions, substantial bidentate ligand dissociation is observed on the CV time scale. Attempts to engender similar bidentate ligand dissociation reactions in bppm complexes at the more substitutionally inert Rh­(III) state revealed no reactivity even under thermolysis or photolysis conditions (Figures S72–S75; see the SI for further discussion), underscoring the role of ET in promoting ligand exchange in **(bppm)­Rh-NCCH**_**3**_.

In light of these findings, the CV data for **(bppm)­(Rh-Cl)**_**2**_ were reevaluated. An event near the potential for the Rh­(III/I) couple in **(bppm)­Rh-Cl** is commonly observed in voltammetric analyses of **(bppm)­(Rh-Cl)**_**2**_ (Figure 6, peaks marked with asterisks). As **(bppm)­(Rh-Cl)**_**2**_ is stable in CH_3_CN, this suggests that the monorhodium complex may be generated upon reduction of the first Rh center in **(bppm)­(Rh-Cl)**_**2**_. Crucially, the extent of ET-induced reactivity in dirhodium complexes **(bppm)­(Rh-Cl)**_**2**_ and **(bppm)­(Rh-NCCH**_**3**_)_**2**_ may, however, be masked by the relative potentials of mono- vs dirhodium species. As the Rh­(III/I) couples in these monorhodium complexes appear at more negative potentials than the Rh­(III,III/III,I) couples of the corresponding dirhodium compounds, no conclusive evidence for a change in solution composition *as a result of ET* can be inferred from the CV data for the dirhodium complexes, since any monorhodium byproducts would appear in the first cathodic scan regardless of the role of ET in their formation. Nonetheless, the stability of **(bppm)­(Rh-Cl)**_**2**_ in solution over a time frame commensurate with CV data collection procedures does provide at least circumstantial evidence for ET-induced reactivity in this complex.

The dissociation of the bidentate ligand in **(bppm)­Rh-NCCH**_**3**_ represents an unusual paradigm in the ligand-exchange profile of the 19e^–^ complexes that result from reduction of half-sandwich Rh­(III) complexes. While dissociation of the monodentate ligand at the Rh­(II) state is well-established,^17^ the possible dissociation of bidentate ligands has received more modest attention. ET-induced interconversion between mono- and dirhodium complexes supported by the bpym ligand was postulated by Štěpnička, Süss-Fink, and coworkers,^34^ though reports by Kaim and coworkers on the same system indicated reactivity at the Rh­(III) state, complicating analysis of ET-induced exchange.^33^ Possible evidence for partial conversion to the corresponding monorhodium complex was also observed in CV data for the dirhodium compound supported by the bppz ligand, also described by Kaim and coworkers, though the original report did not include a discussion of this possibility.^36^ More recently, Blakemore and coworkers observed dissociation of dipyridylmethane- or diazafluorene-type ligands upon reduction of their Rh­(III) complexes, possibly driven by formation of the Rh­(II) [Cp*RhCl]_2_ dimer.^62,73,74^ Importantly, bppm monorhodium compounds are uniquely primed to facilitate detection of bidentate ligand dissociation, as the second binding site on the same bppm platform enables capture of the nascent “free” [Cp*Rh] moiety by the open binding site on a second equivalent of a monorhodium complex. The product of this reactivity is itself an electroactive compound with a substantially different ET profile than its precursor, thereby facilitating its observation.

### Reinvestigation of 2,2’-Bipyrimidine Complexes

Observation of ET-induced ligand exchange in bppm complexes encouraged us to revisit the literature bipyrimidine complexes **(bpym)­Rh-Cl** and **(bpym)­(Rh-Cl)**_**2**_, for which dissociation of a bidentate motif from [Cp*Rh] had been described. The groups of Kaim^33,35^ and Štěpnička and Süss-Fink^34^ reported the synthesis and spectroscopic and electrochemical characterization of the mono- and dirhodium complexes of **(bpym)­Rh-Cl** and **(bpym)­(Rh-Cl)**_**2**_ (Scheme 4). Evidence for electrochemical interconversion between mono- and dirhodium complexes was presented in both reports, with Kaim and coworkers further describing bidentate ligand dissociation in complex **(bpym)­(Rh-Cl)**_**2**_ at the Rh­(III) state. Despite these observations, ET-induced ligand exchange reactivity was not investigated further in either study. Furthermore, several inconsistencies were noted between these reports on the conditions required to synthesize bpym complexes and their stability in various organic solvents (see the SI for further discussion).

**Scheme 4:**
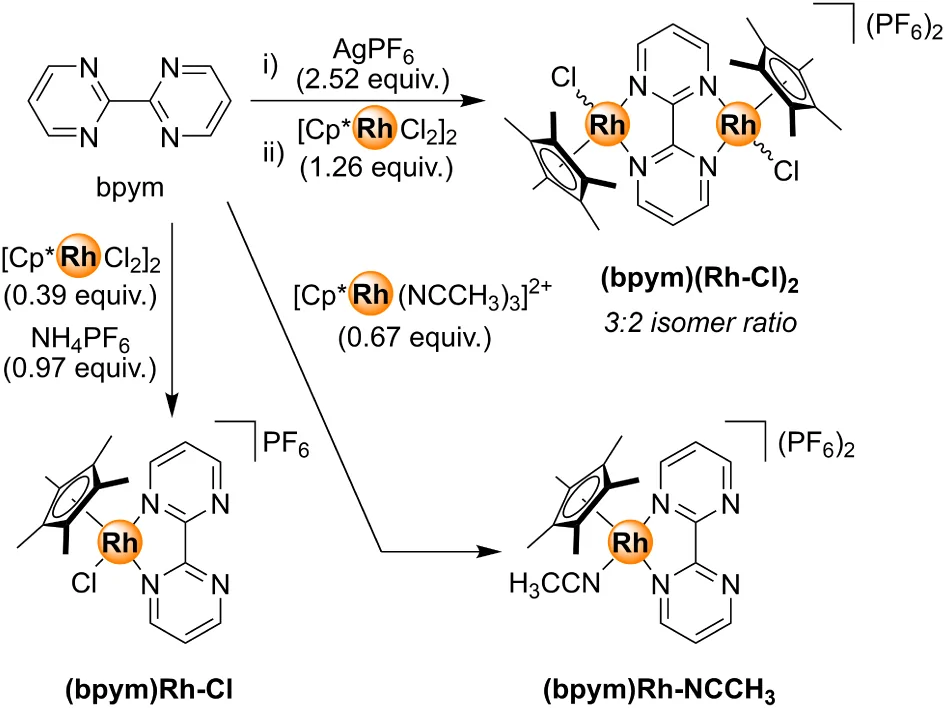
Synthesis of (bpym)­(Rh-X)_n_ Complexes

Complexes **(bpym)­Rh-Cl** and **(bpym)­(Rh-Cl)**_**2**_ were prepared via optimized procedures derived from the original reports (Scheme 4; see the SI for further discussion).^33,34^ In the course of our work, we obtained new X-ray diffraction data for several bpym complexes. Two solid-state structures of monorhodium complex **(bpym)­Rh-Cl** were obtained, one as a CH_3_CN solvate and one without cocrystallized solvents (Figure 11a,b). These structures revealed the expected κ^2^ binding of the bpym framework; the bond and angle metrics were consistent with those of the analogous bipyridine complexes (Tables 6 and 1). Crystals grown from solutions of **(bpym)­(Rh-Cl)**_**2**_ in the presence of CH_3_CN (obtained prior to recognizing the extent of its instability to this solvent) provided a new solventomorph of the *syn* isomer of **(bpym)­(Rh-Cl)**_**2**_ (as a CH_3_CN solvate; Figure 11d), a nonsolvated structure of which had previously been reported.^34^ Despite the observed instability of this complex, the Rh–N bond lengths in **(bpym)­(Rh-Cl)**_**2**_ are only marginally elongated with respect to **(bpym)­Rh-Cl** (Table 6). Attempts to obtain X-ray-quality crystals of **(bpym)­(Rh-Cl)**_**2**_ from noncoordinating solvents have been hampered by its very limited solubility (**(bpym)­(Rh-Cl)**_**2**_ is effectively insoluble in CHCl_3_, CH_2_Cl_2_, and acetone), and crystallization from CH_3_NO_2_ has likewise proved unsuccessful.

**Table 6:** Selected Structural Metrics for bpym Complexes

Complex	(bpym)Rh-Cl·CH_3_CN	(bpym)Rh-Cl^a^	(bpym)(Rh-Cl)_2_ ^a^		(bpym)Rh-NCCH_3_
CCDC#	2574179	2574174	2574176	635001	2574178
bond distances (Å)					
Rh–N1	2.1072(19)	2.118(2)	2.147(4)	2.135(5)	2.129(3)
Rh–N2	2.1254(19)	2.118(2)	2.139(4)	2.159(5)	2.116(3)
Rh–X^b^	2.3923(8)	2.3800(8)	2.3991(14)	2.4053(16)	2.067(3)
Rh–Cp*^c^	1.7821(11)	1.7810(10)	1.786(2)	1.781(3)	1.7812(14)
d_C–C_ ^d^	1.482(3)	1.488(5)	1.466(9)	1.47(1)	1.488(4)
bond and plane angles (°)					
N–Rh–N	76.28(8)	76.18(11)	77.10(15)	76.53(17)	76.21(10)
∠(Cp*-[RhN_2_])^e^	58.66(8)	59.20(11)	59.75(16)	59.9(2)	58.95(11)
Reference	this work	this work	this work	^34^	this work

a Complex on a crystallographic symmetry element.

b Length of the bond between Rh and the monodentate ligand (Cl^–^ or NCCH_3_).

c Distance between Rh and the calculated centroid of the Cp­(*) ligand.

d Length of the inter-ring C–C bond between pyrimidine heterocycles.

e Angle between the plane of the Cp* ligand and the calculated mean plane for the 5-membered Rh metallocycle.

**Figure 11:**
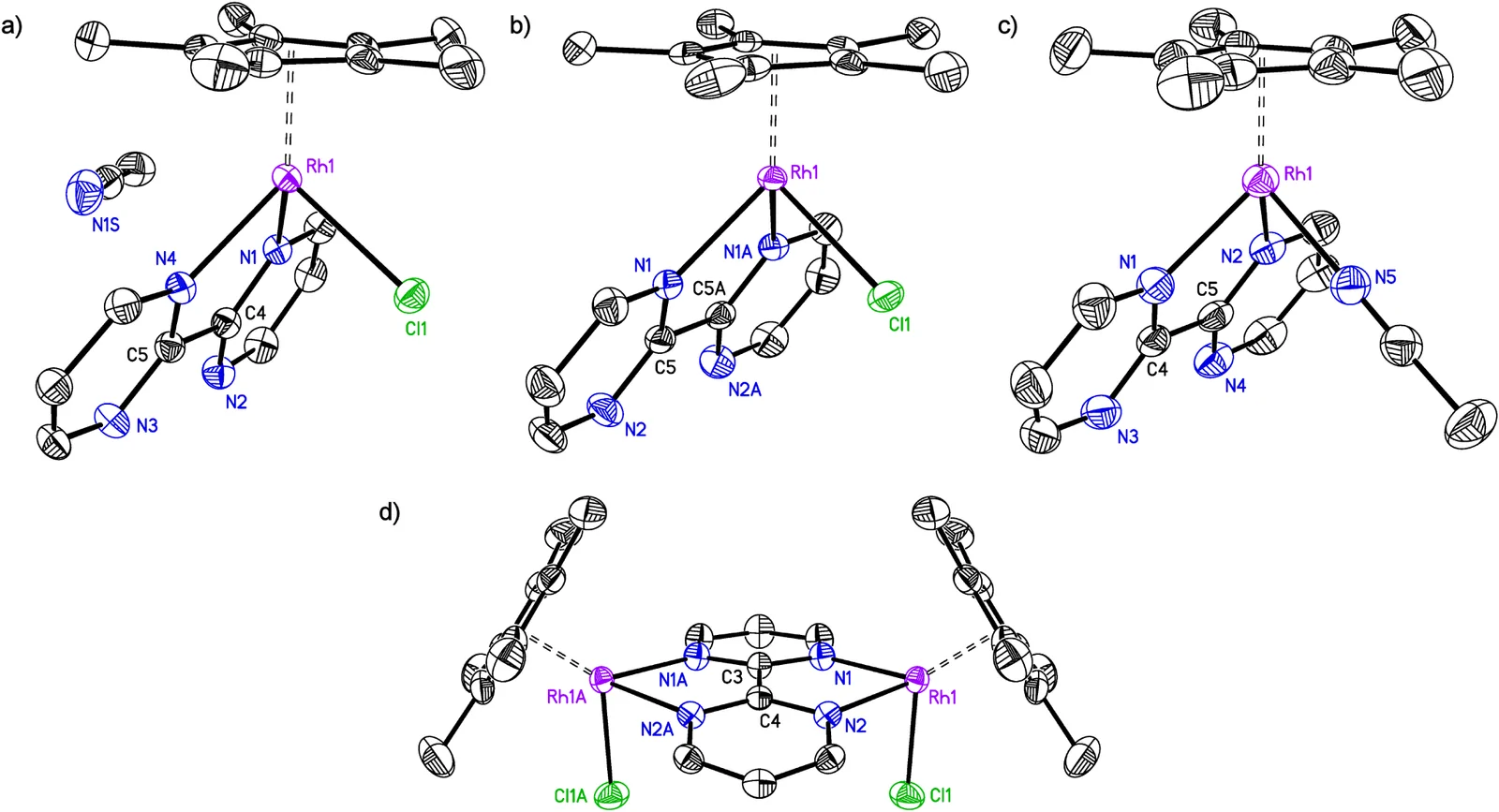
Solid-state structures (XRD) of (a) **(bpym)**Rh-C**l•CH**_3_ **CN**, (b) **(bpym)­Rh-Cl**, (c) **(bpym)­Rh-NCCH_3_**, and (d) **(bpym)­(Rh-Cl)_2_**. H atoms and outer-sphere counteranions omitted for clarity. Displacement ellipsoids shown at the 50% probability level. Data collected at 100 K.

As reported by Štěpnička and Süss-Fink, **(bpym)­(Rh-Cl)**_**2**_ displayed several irreversible reduction processes in N,N-dimethylformamide (DMF) between −0.8 and −1.4 V vs Fc^+/0^, including events consistent with conversion to complex **(bpym)­Rh-Cl**, though this reactivity was not investigated further.^34^ CV data obtained by Kaim in CD_3_CN, conversely suggested near-complete conversion to **(bpym)­Rh-Cl** prior to the initial reduction event—i.e., via ligand exchange at Rh­(III).^33^ In our hands, CV data collected for **(bpym)­Rh-Cl** in CH_3_CN displayed a 2e^–^ process centered at −1.08 V vs Fc^+/0^ (Figure 12, upper trace), consistent with an earlier report by Kaim^35^ and ∼100 mV more negative than the potential determined in DMF (*E*_1/2_ = −0.60 V vs SCE, −0.98 V vs Fc^+/0^). Analysis of **(bpym)­(Rh-Cl)**_**2**_, conversely, indicated substantial but incomplete conversion to **(bpym)­Rh-Cl** upon dissolution: events consistent with **(bpym)­Rh-Cl** were observed in the first CV scan (Figure 12, lower trace), yet a cathodic peak consistent with reduction of **(bpym)­(Rh-Cl)**_**2**_ was also observed (*E*_pc_ = −0.80 V). These observations are consistent with our NMR studies on the stability of **(bpym)­(Rh-Cl)**_**2**_, indicating facile but not immediately complete decomposition in the presence of CH_3_CN.

**Figure 12:**
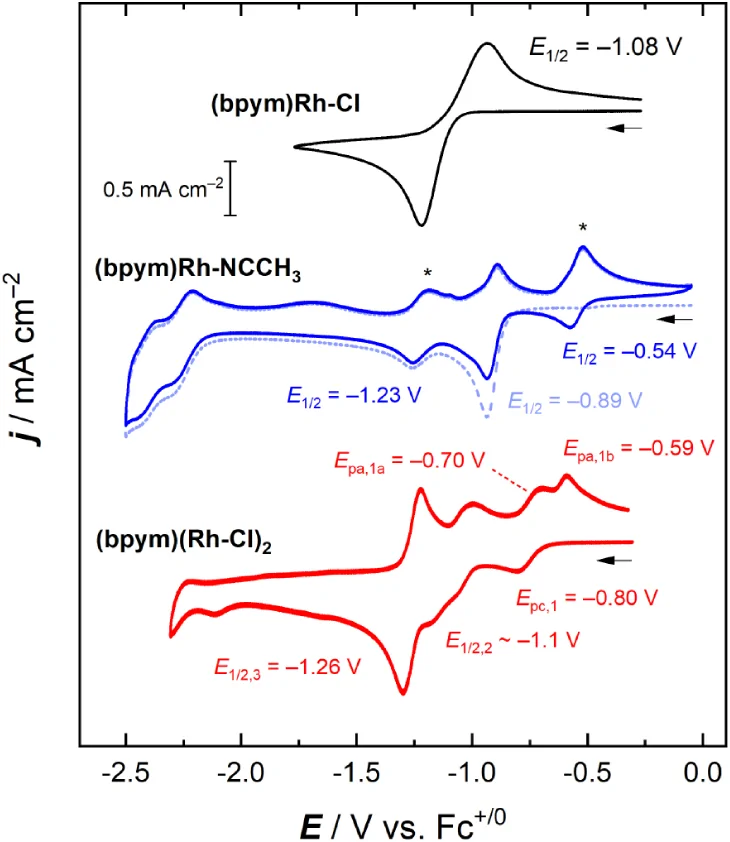
CV data (0.1 M [^n^Bu_4_N]­[PF_6_] in CH_3_CN, 100 mV/s, [Rh complex] = 2 mM) for **(bpym)­Rh-Cl** (upper trace, black), **(bpym)­Rh-NCCH_3_** (middle traces; light blue: first CV scan; dark blue: second CV scan), and **(bpym)­(Rh-Cl)_2_** (lower trace, red). Peaks marked with (*) correspond to the putative dirhodium solvento complex generated following reduction of **(bpym)­Rh-NCCH_3_**.

As ET-induced bppm exchange was influenced by the nature of the monodentate ligand at Rh, we hypothesized that similar effects may be observed in bpym compounds. Solvento complex **(bpym)­Rh-NCCH**_**3**_ was prepared via metalation of the bpym ligand with [Cp*Rh­(NCCH_3_)_3_]^2+^. This complex displayed analogous ^1^H NMR signatures to those for the corresponding chloride complex; as in **(bppm)­Rh-NCCH**_**3**_, no signal corresponding to the solvent ligand was observed in ^1^H NMR data, indicating fast exchange in CD_3_CN. Solid-state characterization of **(bpym)­Rh-NCCH**_**3**_ via XRD revealed the expected ligand set at Rh, with a CH_3_CN molecule having replaced the Cl^–^ ligand in **(bpym)­Rh-NCCH**_**3**_ (Figure 11c). The structural metrics for **(bpym)­Rh-NCCH**_**3**_ are nearly indistinguishable from those of **(bpym)­Rh-Cl**, suggesting minimal structural effects of the replacement of Cl^–^ by CH_3_CN.

CV data for **(bpym)­Rh-NCCH**_**3**_ in CH_3_CN revealed clear evidence for ET-induced bidentate ligand exchange. In the initial cathodic scan, the first ET event is observed at *E*_pc_ = −0.94 V (Figure 12, middle trace); this potential is consistent with reduction of **(bpym)­Rh-NCCH**_**3**_, with a 0.28 V positive shift from the potential required for reduction of **(bpym)­Rh-Cl**. A second reduction peak is observed at −1.26 V, and additional events are observed below −2 V. In the anodic return scan and in subsequent voltammetric cycles, however, a new event is also clearly observed at −0.54 V that was absent in the initial cathodic sweep, consistent with a change in solution composition following reduction. While we have been unable to generate dirhodium solvento complex **(bpym)­(Rh-NCCH**_**3**_)_**2**_, and the identity of the species produced during reduction of **(bpym)­Rh-NCCH**_**3**_ could not be independently confirmed, the similarity of its CV features (*E*_1/2_ = −0.54 and −1.23 V) to those of dirhodium complex **(bpym)­(Rh-Cl)**_**2**_ (*E*_1/2_ = ∼−0.7 and −1.26 V) strongly suggests formation of the corresponding dirhodium species. The relative reactivity of **(bpym)­Rh-NCCH**_**3**_ vs **(bpym)­Rh-Cl** is consistent with the bppm complexes—the presence of the neutral acetonitrile ligand appears to favor dissociation of the bidentate framework.

These results indicate that the properties of mono- and dirhodium bpym complexes are closely correlated to those of bppm compounds. Dirhodium complexes supported by the bpym ligand are significantly more sensitive to CH_3_CN even at the less substitutionally labile Rh­(III) state, likely due to the shared nature of the two heterocycle donors in the bpym platform, which appears to diminish the strength of its binding to a second [Cp*Rh] fragment. Similar to bppm complexes, solvento bpym compounds are generally more prone to bidentate ligand exchange; the additional instability inherent to the bpym framework, furthermore, prevents the chemical preparation of a dirhodium solvento complex, while the corresponding bppm complex can be accessed under noncoordinating conditions. These results emphasize the demands imposed by a binucleating ligand framework in which both metal centers share donors from the same two heterocycles, which results in more extensive reactivity even at the typically more inert Rh­(III) state.

## Conclusions

Mono- and bis-[Cp*Rh] complexes of 4,6-bis­(pyridyl)­pyrimidine were synthesized and characterized via spectroscopic, structural, and electrochemical methods. The stability profile, structural features, and ET properties of these complexes depend intimately on the nuclearity of the compound ([Rh] vs [Rh_2_]) and on the identity of the monodentate ligand that completes the coordination sphere at Rh (Cl^–^ vs NCCH_3_). Monorhodium chloride complex **(bppm)­Rh-Cl** undergoes a net 2e^–^ reduction at a potential comparable to that of related bipyridine complexes. Spectroscopic and structural analyses of the chemically prepared reduced complex **(bppm)­Rh** confirmed participation of the bppm framework in stabilization of the formally Rh­(I) center, with crystallographic data indicating delocalization of electron density primarily onto the fragment of the bppm ligand directly bound to Rh.

Complex **(bppm)­(Rh-Cl)**_**2**_ undergoes two distinct 2e^–^ reductions at more positive and more negative potentials, respectively, than the Rh­(III/I) couple in **(bppm)­Rh-Cl**. Signals corresponding to the cathodic and anodic events for **(bppm)­Rh-Cl** are also routinely observed in CV data for **(bppm)­(Rh-Cl)**_**2**_, hinting at partial dissociation of the bppm framework from Rh following ET. Nonetheless, thermolysis and photolysis experiments revealed **(bppm)­(Rh-Cl)**_**2**_ to be more stable (at the Rh­(III) state) than other dirhodium complexes in this family, especially in the presence of coordinating solvent (e.g., CH_3_CN), consistent with a less demanding binucleating platform. **(bppm)­Rh-Cl** and **(bppm)­(Rh-Cl)**_**2**_ were found to not be particularly competent H_2_ evolution electrocatalysts vs literature bpy complexes, indicating that neither the ability to store additional reducing equivalents (**(bppm)­(Rh-Cl)**_**2**_) nor the presence of additional proton-responsive motifs (**(bppm)­Rh-Cl**) result in substantial improvement in catalytic performance in this context.

The exchange of Cl^–^ for NCCH_3_ reveals new reactivity manifolds in these complexes. Evidence for ET-induced bidentate ligand exchange was observed in the electrochemical analyses of monorhodium solvento complex **(bppm)­Rh-NCCH**_**3**_. These observations implicate participation of bidentate frameworks in reactivity that parallels the well-established dissociation of the monodentate ligand (Cl^–^ or solvent) upon reduction under electrochemical conditions. While dissociation of bidentate donors from [Cp*Rh] had been reported for select ligand platforms, typically as a result of steric or electronic constraints enforced by these frameworks, the reactivity of **(bppm)­Rh-NCCH**_**3**_ represents the clearest example of bidentate ligand dissociation reactivity induced by reduction to the Rh­(II) state. The labilization of the Rh–bppm interactions indicated by these observations is further showcased by the instability of dirhodium complex **(bppm)­(Rh-NCCH**_**3**_)_**2**_ to coordinating compounds even at the more substitutionally inert Rh­(III) state.

Based on our findings on bppm complexes, the mono- and dirhodium complexes supported by the 2,2’-bipyrimidine ligand, which had appeared in the literature but whose ET-induced reactivity had not been fully elucidated, were reinvestigated. While the bpym platform supports both mono- and dirhodium complexes, these are substantially more prone to dissociation of the bidentate motifs from Rh than their bppm analogues, potentially as a result of the sharing of both heterocycle donors by both Rh centers in dirhodium species. Thus, evidence for generation of monorhodium complex **(bpym)­Rh-Cl** was observed in both spectroscopic and electrochemical analyses of **(bpym)­(Rh-Cl)**_**2**_, implicating bidentate ligand dissociation at Rh­(III), with possible further exchange at Rh­(II) being masked by reactivity in the more oxidized precursor. As in the case of bppm, exchange of Cl^–^ for NCCH_3_ results in increased lability of the bidentate ligand, leading to ET-induced dissociation of bpym from Rh in **(bpym)­Rh-NCCH**_**3**_ and to **(bpym)­(Rh-NCCH**_**3**_)_**2**_ being chemically inaccessible. These findings highlight the role of the structural features of binucleating polyheterocyclic scaffolds in regulating the stability of [Cp*Rh] complexes, emphasizing the importance of rigorously evaluating the binding profile of candidate frameworks for applications in catalysis.

## Supplementary Material



## Notes

NMR (^1^H, ^13^C, ^31^P, ^19^F, and ^15^N) spectra for characterization; discussion of ^15^N NMR data; spectroscopic data for reactivity of **(bppm)­Rh-Cl**, **(bppm)­(Rh-Cl)**_**2**_, **(bppm)­Rh-NCCH**_**3**_, **(bppm)­(Rh-NCCH**_**3**_)_**2**_, and **(bpym)­(Rh-Cl)**_**2**_; additional discussion of the chemical reactivity of bppm complexes; discussion of the synthesis and chemical stability of bpym complexes; additional CV data, including CV data for [Cp*Rh­(NCCH_3_)_3_]^2+^; additional X-ray diffraction data and refinement details for all XRD structures; and additional discussion of XRD analyses (PDF)
