# Supplementary material for: Mono- and Bimetallic [Cp*Rh] Complexes of the 4,6-Bis(pyridyl)pyrimidine Ligand
Source: Organometallics. 2026 Oct 16;45(21):2592–608. doi: 10.1021/acs.organomet.6c00293 (PMC13753723; doi:10.1021/acs.organomet.6c00293)
Supplement: Supplementary file 1 [file om6c00293_si_001.pdf]

Supporting information for

Mono- and Bimetallic [Cp*Rh] Complexes of the 4,6-bis(Pyridyl)Pyrimidine Ligand

*Jiayi Yu,[a,‡] Charlie O. Griebel,[a ‡] Thalia R. O'Neill,[a] Robert D. Pike,[b] Davide Lionetti[a,] **

[a] Department of Chemistry, Franklin & Marshall College, P.O. Box 3003, Lancaster, PA 17604, USA
[b] Department of Chemistry, College of William & Mary, P.O. Box 8795, Williamsburg, VA 23187
‡ these authors contributed equally; * corresponding author: dlionett@fandm.edu;

Contents **Page**

NMR data S4
Characterization of new compounds........ S4
Figure S1. ^{1}H NMR spectrum of **(bppm)Rh-Cl**........ S4
Figure S2. ^{13}C{^{1}H} NMR spectrum of **(bppm)Rh-Cl**........ S4
Figure S3. ^{31}P{^{1}H} NMR spectrum of **(bppm)Rh-Cl** S5
Figure S4. ^{19}F NMR spectrum of **(bppm)Rh-Cl** S5
Figure S5. COSY spectrum of **(bppm)Rh-Cl**........ S6
Figure S6. ^{1}H-^{13}C HSQC spectrum of **(bppm)Rh-Cl**........ S6
Figure S7. ^{1}H-^{13}C HMBC spectrum of **(bppm)Rh-Cl**........ S7
Figure S8. NOESY spectrum of **(bppm)Rh-Cl**........ S7
Figure S9. ^{1}H-^{15}N HMBC spectrum for **(bppm)Rh-Cl** S8
Figure S10. ^{1}H NMR spectrum of **(bppm)(Rh-Cl)₂**........ S9
Figure S11. ^{1}H NMR of **(bppm)(Rh-Cl)₂** highlighting the minor diastereomer S9
Figure S12. ^{13}C{^{1}H} NMR spectrum of **(bppm)(Rh-Cl)₂**........ S10
Figure S13. ^{31}P{^{1}H} NMR spectrum of **(bppm)(Rh-Cl)₂** S10
Figure S14. ^{19}F NMR spectrum of **(bppm)(Rh-Cl)₂** S11
Figure S15. COSY spectrum of **(bppm)(Rh-Cl)₂**........ S11
Figure S16. ^{1}H-^{13}C HSQC spectrum of **(bppm)(Rh-Cl)₂**........ S12
Figure S17. ^{1}H-^{13}C HMBC spectrum of **(bppm)(Rh-Cl)₂**........ S12
Figure S18. NOESY spectrum of **(bppm)(Rh-Cl)₂** S13
Figure S19. ^{1}H-^{15}N HMBC spectrum for **(bppm)(Rh-Cl)₂** S13
Figure S20. ^{1}H NMR spectrum of **(bppm)Rh-NCCH₃**........ S14
Figure S21. ^{1}H NMR spectrum for purity determination of **(bppm)Rh-NCCH₃**........ S14
Figure S22. ^{13}C{^{1}H} NMR spectrum of **(bppm)Rh-NCCH₃**........ S15
Figure S23. ^{31}P{^{1}H} NMR spectrum of **(bppm)Rh-NCCH₃** S15
Figure S24. ^{19}F NMR spectrum of **(bppm)Rh-NCCH₃** S16
Figure S25. COSY spectrum of **(bppm)Rh-NCCH₃**........ S16
Figure S26. ^{1}H-^{13}C HSQC spectrum of **(bppm)Rh-NCCH₃**........ S17
Figure S27. ^{1}H-^{13}C HMBC spectrum of **(bppm)Rh-NCCH₃**........ S17
Figure S28. NOESY spectrum of **(bppm)Rh-NCCH₃** S18
Figure S29. ^{1}H-^{15}N HMBC spectrum for **(bppm)Rh-NCCH₃** S18
Figure S30. ^{1}H NMR spectrum of **(bppm)(Rh-NCCH₃)₂**........ S19
Figure S31. ^{1}H NMR spectrum of **(bppm)(Rh-NCCH₃)₂** with isomer labels........ S19
Figure S32. ^{13}C{^{1}H} NMR spectrum of **(bppm)(Rh-NCCH₃)₂**........ S20
Figure S33. ^{31}P{^{1}H} NMR spectrum of **(bppm)(Rh-NCCH₃)₂** S20
Figure S34. ^{19}F NMR spectrum of **(bppm)(Rh-NCCH₃)₂** S21
Figure S35. COSY spectrum of **(bppm)(Rh-NCCH₃)₂**........ S21
Figure S36. ^{1}H-^{13}C HSQC spectrum of **(bppm)(Rh-NCCH₃)₂**........ S22
Figure S37. ^{1}H-^{13}C HSQC spectrum of **(bppm)(Rh-NCCH₃)₂**........ S22

Figure S38. ^1H-^{13}C HMBC spectrum of **(bppm)(Rh-NCCH$_3$)$_2$**......S23
Figure S39. ^1H-^{13}C HMBC spectrum of **(bppm)(Rh-NCCH$_3$)$_2$**......S23
Figure S40. NOESY spectrum of **(bppm)(Rh-NCCH$_3$)$_2$**......S24
Figure S41. ^1H-^{15}N HMBC spectrum for **(bppm)(Rh-NCCH$_3$)$_2$**......S24
Figure S42. ^1H NMR spectrum of **(bppm)Rh**......S25
Figure S43. $^{13}C\{^1H\}$cvvv NMR spectrum of **(bppm)Rh**......S25
Figure S44. COSY spectrum of **(bppm)Rh**......S26
Figure S45. ^1H-^{13}C HSQC spectrum of **(bppm)Rh**......S26
Figure S46. NOESY spectrum of **(bppm)Rh**......S27
Figure S47. ^1H-^{15}N HMBC spectrum for **(bppm)Rh**......S27
Figure S48. ^1H NMR spectrum of **(bpym)Rh-NCCH$_3$**......S28
Figure S49. $^{13}C\{^1H\}$ NMR spectrum of **(bpym)Rh-NCCH$_3$**......S28
Figure S50. $^{31}P\{^1H\}$ NMR spectrum of **(bpym)Rh-NCCH$_3$**......S29
Figure S51. ^{19}F NMR spectrum of **(bpym)Rh-NCCH$_3$**......S29
Figure S52. COSY spectrum of **(bpym)Rh-NCCH$_3$**......S30
Figure S53. ^1H-^{13}C HSQC spectrum of **(bpym)Rh-NCCH$_3$**......S30
Figure S54. ^1H-^{13}C HMBC spectrum of **(bpym)Rh-NCCH$_3$**......S31
Figure S55. NOESY spectrum of **(bpym)Rh-NCCH$_3$**......S31
Figure S56. ^1H-^{15}N HMBC spectrum for **(bpym)Rh-NCCH$_3$**......S32
^{15}N NMR data for literature compounds......S33
Figure S57. ^1H-^{15}N HMBC spectrum for **bppm**......S33
Figure S58. ^1H-^{15}N HMBC spectrum for **bpy**......S33
Figure S59. ^1H-^{15}N HMBC spectrum for **(bpy)Rh-Cl**......S34
Figure S60. ^1H-^{15}N HMBC spectrum for **(bpy)Rh-NCCH$_3$**......S34
Figure S61. ^1H-^{15}N HMBC spectrum for **(bpy)Rh**......S35
Figure S62. ^1H-^{15}N HMBC spectrum for **bpym**......S35
Figure S63. ^1H-^{15}N HMBC spectrum for **(bpym)Rh-Cl**......S36
Figure S64. ^1H-^{15}N HMBC spectrum for **(bpym)(Rh-Cl)$_2$**......S36
Table S1. Summary of ^{15}N data for compounds containing polyheterocyclic platforms......S37
Discussion of ^{15}N NMR data......S37
Other NMR data......S39
Figure S65. VT ^1H NMR spectra of **(bppm)(Rh-Cl)$_2$**......S39
Figure S66. ^1H NMR spectra for **(bppm)(Rh-Cl)$_2$** after heating......S40
Figure S67. ^1H NMR monitoring of reaction of **(bppm)(Rh-NCCH$_3$)$_2$** with CD_3CN......S41
Figure S68. ^1H NMR monitoring of reaction of **(bppm)Rh-NCCH$_3$** with $^nBu_4NCl$......S42
Figure S69. ^1H NMR monitoring of reaction of **(bppm)(Rh-NCCH$_3$)$_2$** with $^nBu_4NCl$......S43
Figure S70. ^1H NMR monitoring of reaction of **(bppm)(Rh-Cl)$_2$** with $^nBu_4NCl$......S44
Figure S71. ^1H NMR monitoring of reaction of **(bppm)Rh-NCCH$_3$** with $[Cp^*Rh(NCCH_3)_3]^{2+}$......S45
Figure S72. ^1H NMR data for **(bppm)Rh-Cl** under heating......S46
Figure S73. ^1H NMR data for **(bppm)Rh-Cl** under photolysis......S47
Figure S74. ^1H NMR data for **(bppm)Rh-NCCH$_3$** under heating......S48
Figure S75. ^1H NMR data for **(bppm)Rh-NCCH$_3$** under photolysis......S49
Discussion of studies of bidentate ligand exchange at the Rh(III) state.......S49
Discussion of synthesis and stability of mono- and dirhodium bpym complexes......S50
Figure S76. ^1H NMR spectra for **(bpym)(Rh-Cl)$_2$** (three synthetic replicates)......S51
Figure S77. ^1H NMR spectrum for the synthesis of **(bpym)(Rh-Cl)$_2$** at higher concentration......S51
Figure S78. VT ^1H NMR spectra for **(bpym)(Rh-Cl)$_2$**......S52
Figure S79. ^1H NMR spectra for **(bpym)(Rh-Cl)$_2$** after heating......S53
Figure S80. ^1H NMR spectra for addition of CD_3CN to **(bpym)(Rh-Cl)$_2$**......S54

Electrochemistry S55
Figure S81. Scan rate dependence studies for **(bppm)Rh-Cl** S55
Figure S82. Scan rate dependence studies for **(bppm)(Rh-Cl)$_2$** S56
Figure S83. CV data for **(bppm)(Rh-Cl)$_2$** at high scan rates S57
Figure S84. Scan rate dependence for **(bppm)Rh-NCCH$_3$** S58
Figure S85. CV data for **(bppm)Rh-NCCH$_3$** at high scan rates S59
Figure S86. CV data for (bppm)Rh-NCCH$_3$ in 1,2-difluorobenzene S60
Figure S87. Scan rate dependence studies for **(bpym)Rh-NCCH$_3$** S61
Figure S88. CV data for **(bpym)Rh-NCCH$_3$** at high scan rates S62
Figure S89. CV data for [Cp*Rh(NCCH$_3$)$_3$](PF$_6$)$_2$. S63

X-Ray Crystallography S64
Table S2. Crystal and refinement data S64
Table S3. Crystal and refinement data (cont'd) S65
Table S4. Crystal and refinement data (cont'd) S66
Table S5. Comparison of metrics for **(bppm)Rh-Cl** with additional monorhodium complexes S67
Additional analysis of structural data: distortions in polyheterocyclic ligands S68
Chart S1. Interplane angles in polyheterocyclic ligand frameworks S68
Chart S2. Mono- and dirhodium complexes for structural analysis. S68
Table S6. Ligand structural metrics for the complexes investigated in the present study S69
Table S7. Structural metrics relevant to π-π interactions S72
Analysis of metallocycle planarity vs. polyheterocyclic ligand twist angle S73
Table S8. Metallocycle structural metrics S74
Figure S90. Plot of the normal Rh-metallocycle distance vs. metallocycle RMSD S75
Refinement details for (bppm)Rh-Cl S76
Figure S91. Full solid-state structure for **(bppm)Rh-Cl** S76
Refinement details for (bppm)(Rh-Cl)$_2$ S77
Figure S92. Full solid-state structure for **(bppm)(Rh-Cl)$_2$** S77
Refinement details for (bppm)Rh-NCCH$_3$ S78
Figure S93. Full solid-state structure for **(bppm)Rh-NCCH$_3$** S78
Refinement details for (bppm)Rh S79
Figure S94. Full solid-state structure for **(bppm)Rh** S79
Refinement details for (bpym)Rh-Cl S80
Figure S95. Full solid-state structure for **(bpym)Rh-Cl** S80
Refinement details for (bpym)Rh-Cl•CH$_3$CN S81
Figure S96. Full solid-state structure for **(bpym)Rh-Cl•CH$_3$CN** S81
Refinement details for (bpym)(Rh-Cl)$_2$ S82
Figure S97. Full solid-state structure for **(bpym)(Rh-Cl)$_2$** S82
Refinement details for (bpym)Rh-NCCH$_3$ S83
Figure S98. Full solid-state structure for **(bpym)Rh-NCCH$_3$** S83

References S84

NMR data

Characterization of new compounds

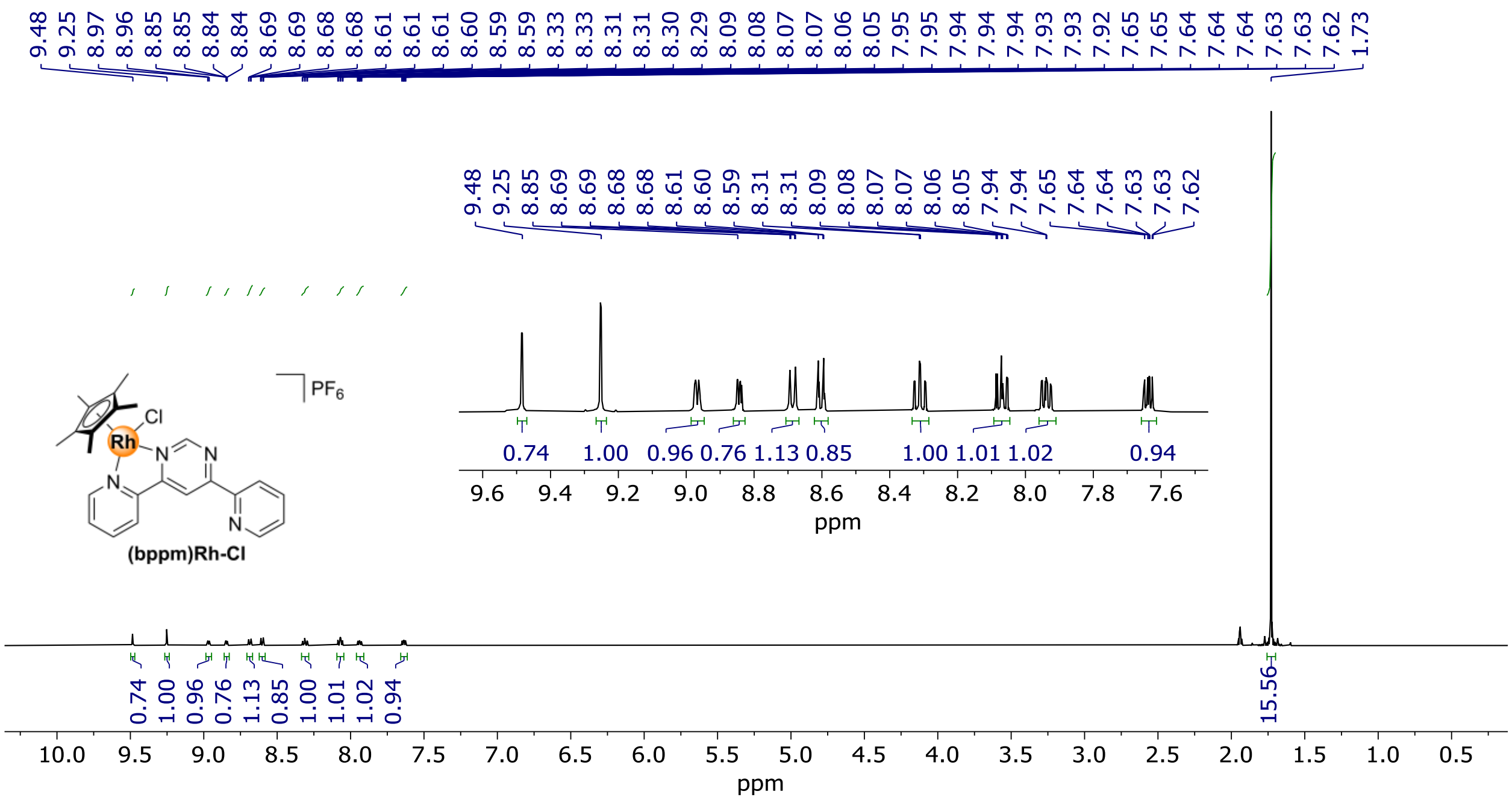

Figure S1. ^{1}H NMR (500 MHz, CD_3CN) of **(bppm)Rh-Cl**. Inset: enlarged view of aromatic region.

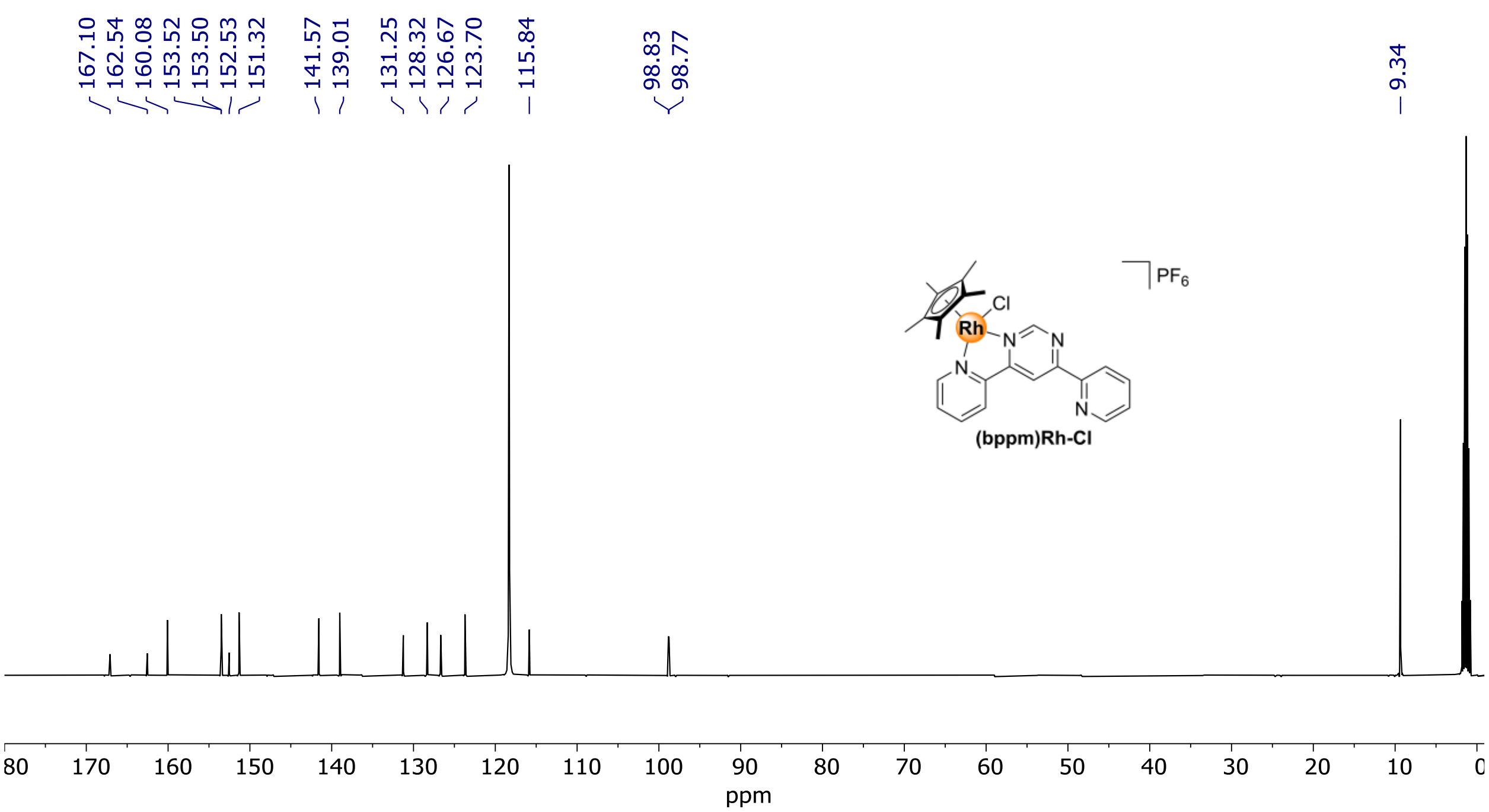

Figure S2. $^{13}C\{^1H\}$ NMR spectrum (125 MHz, CD_3CN) of **(bppm)Rh-Cl**.

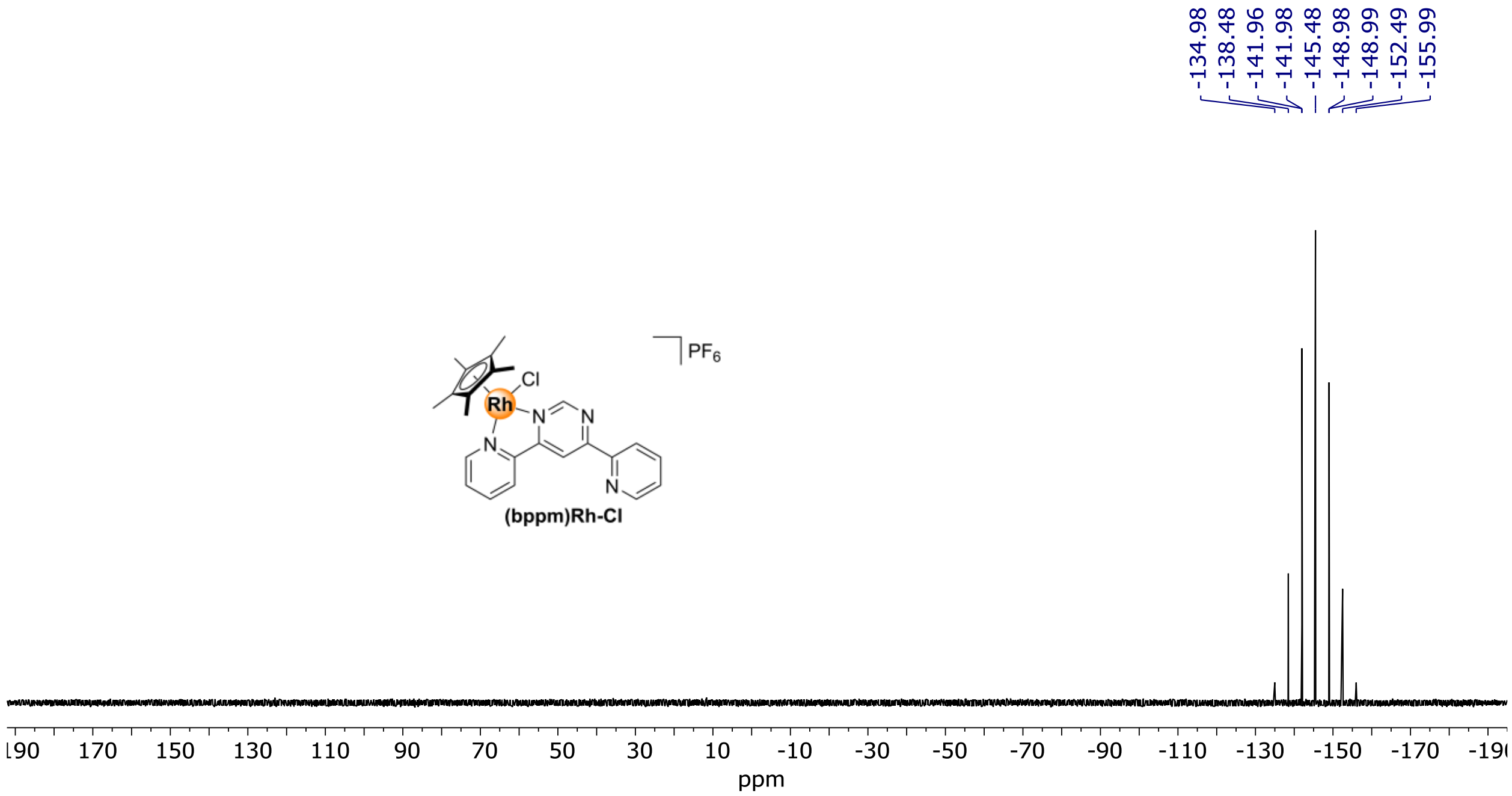

Figure S3. $^{31}P\{^{1}H\}$ NMR spectrum (202 MHz, CD_3CN) of **(bppm)Rh-Cl**.

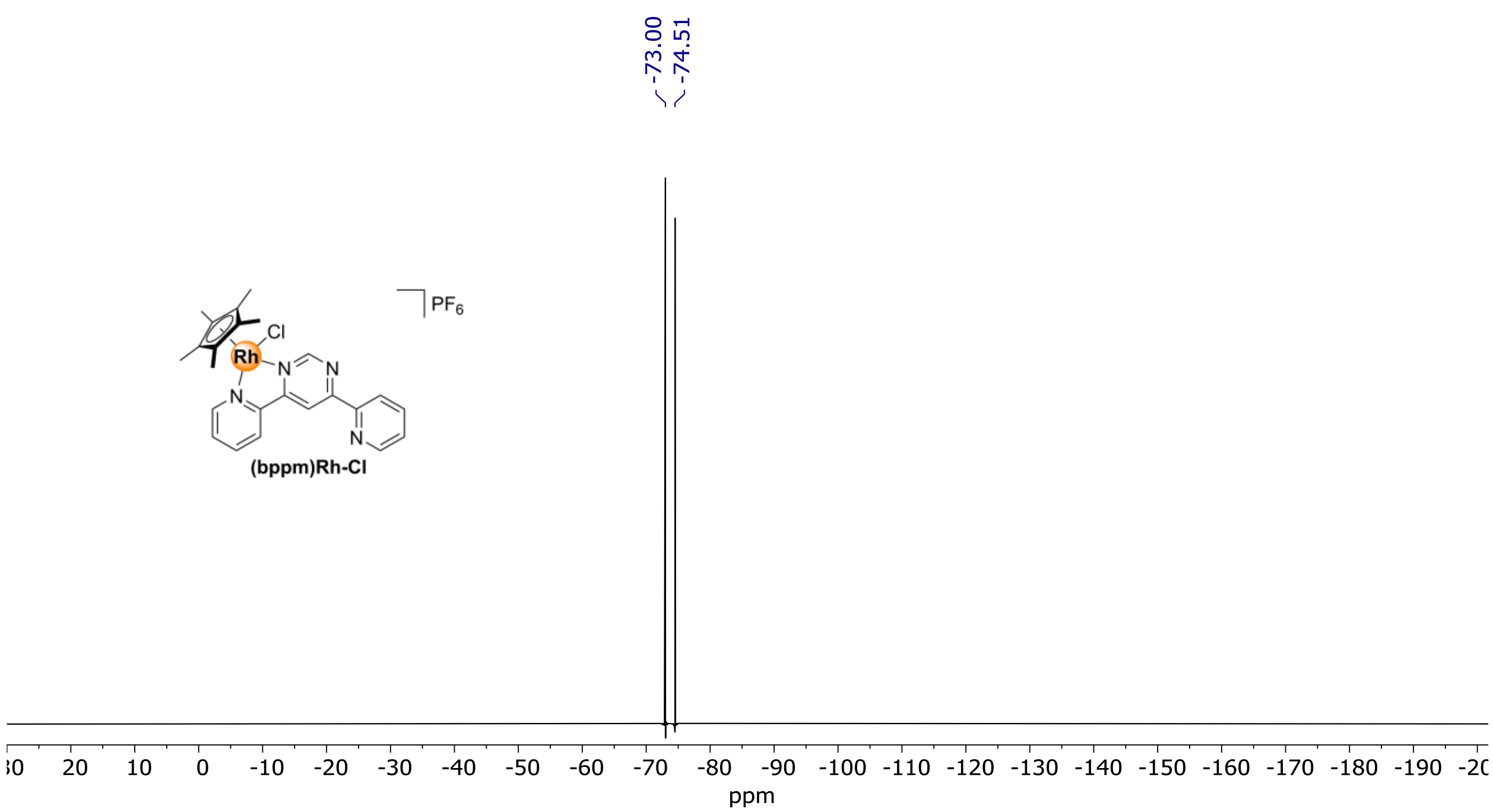

Figure S4. ^{19}F NMR spectrum (469 MHz, CD_3CN) of **(bppm)Rh-Cl**.

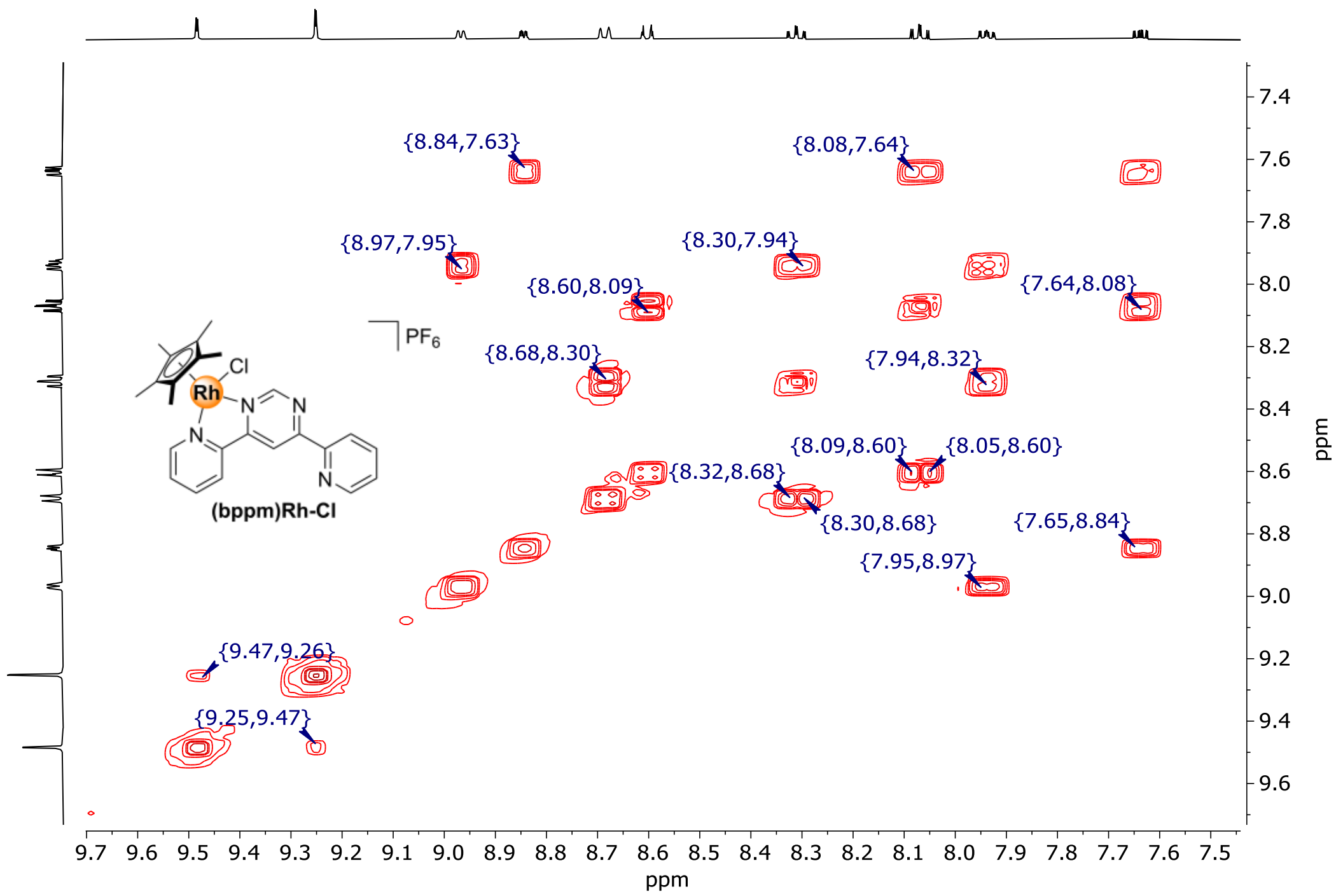

Figure S5. Partial COSY spectrum (500 MHz, CD_3CN) of **(bppm)Rh-Cl**.

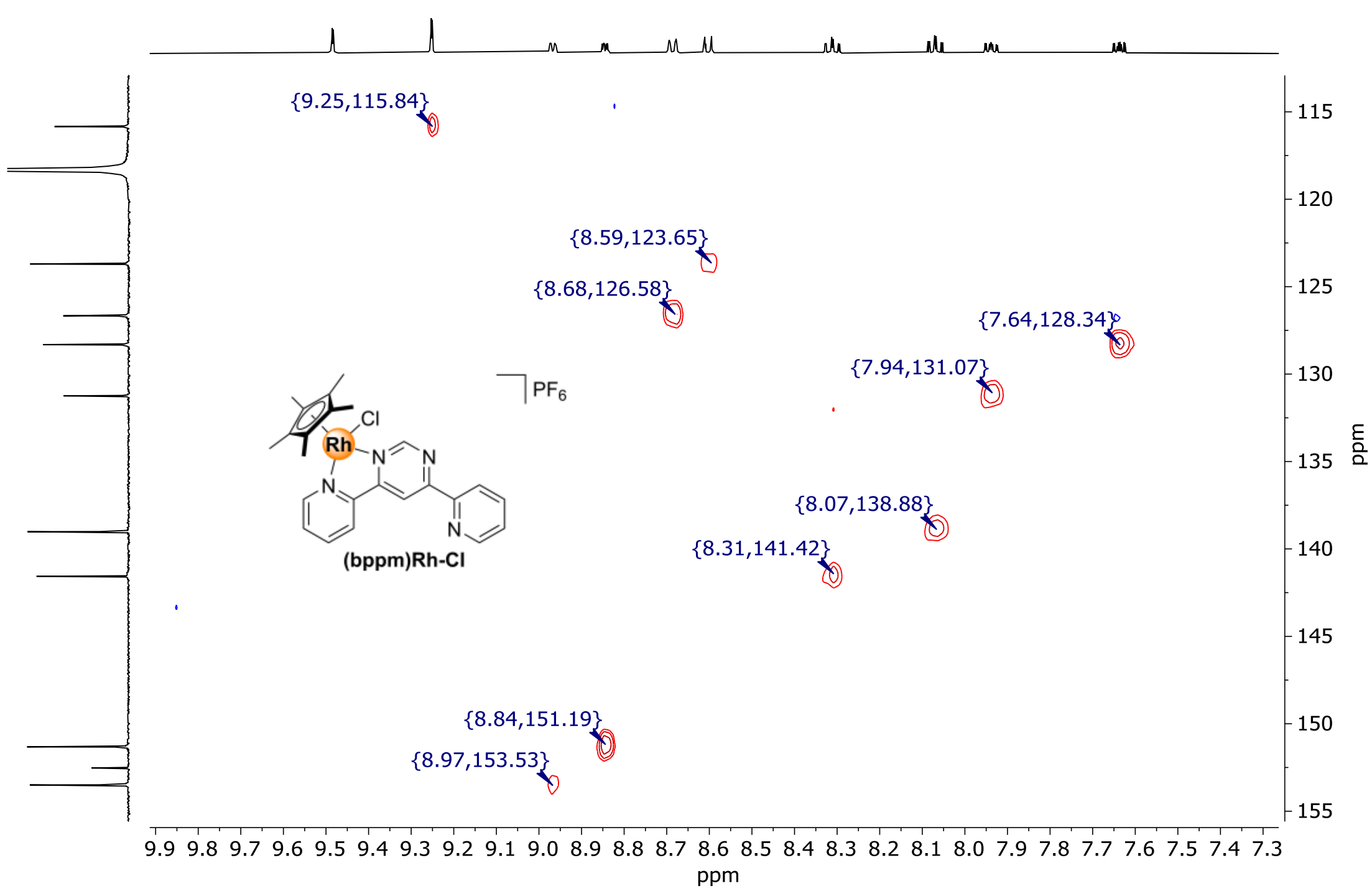

Figure S6. Partial ^{1}H-^{13}C HSQC spectrum (^{1}H: 500 MHz, ^{13}C: 125 MHz, CD_3CN) of **(bppm)Rh-Cl**.

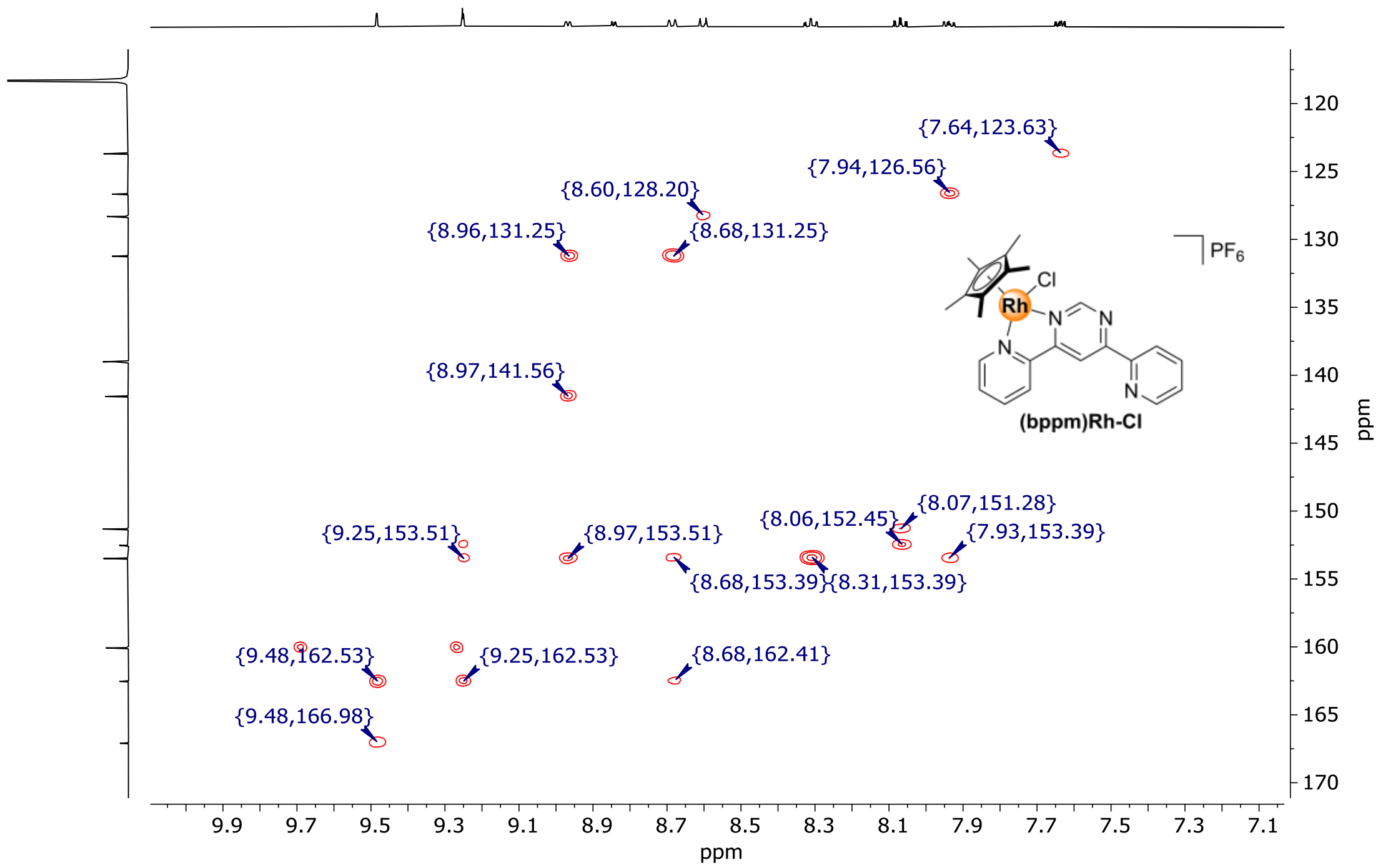

Figure S7. Partial ^{1}H-^{13}C HMBC spectrum (^{1}H: 500 MHz, ^{13}C: 125 MHz, CD_3CN) of **(bppm)Rh-Cl**.

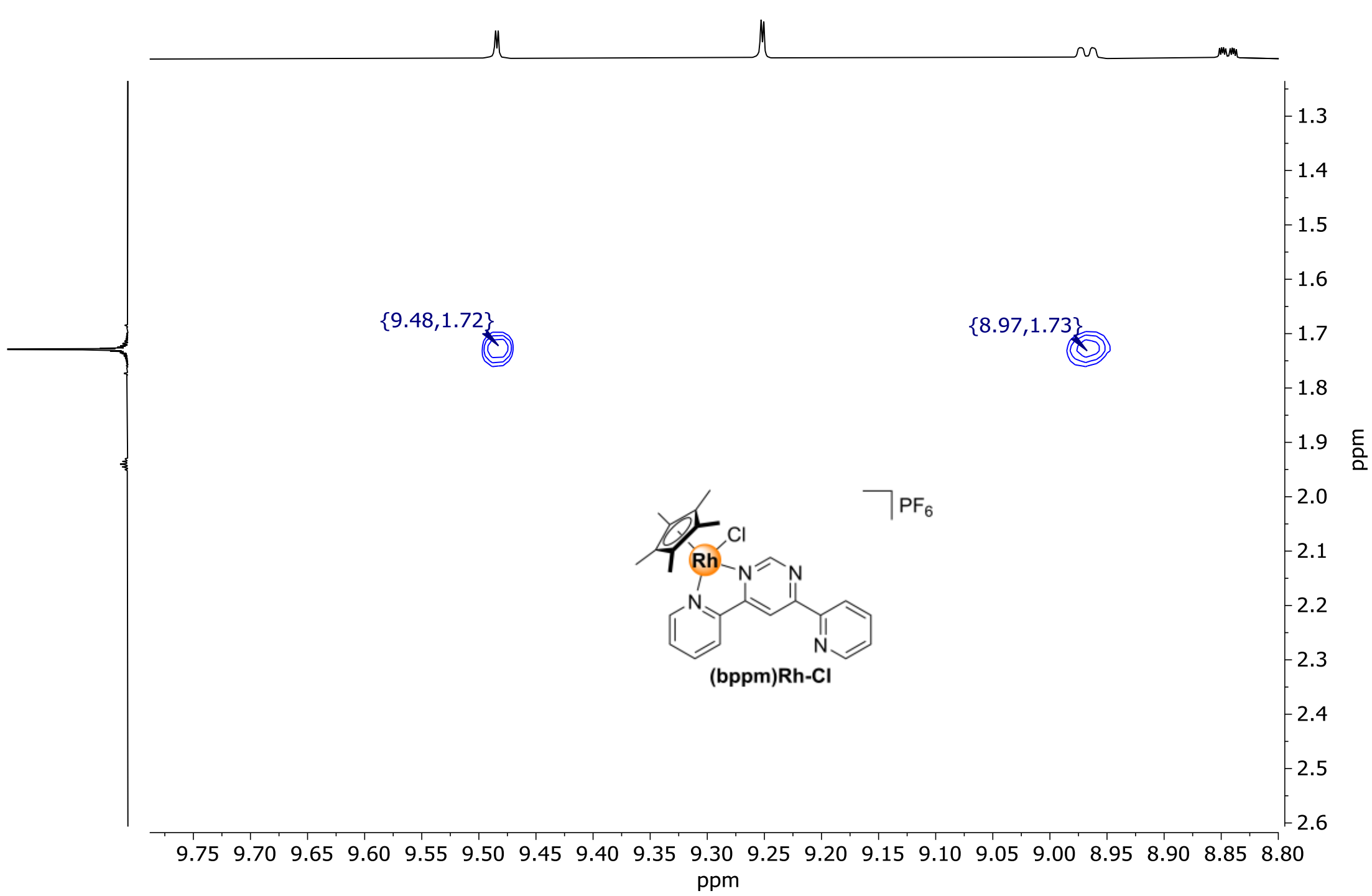

Figure S8. Partial NOESY spectrum (500MHz, CD_3CN) of **(bppm)Rh-Cl**.

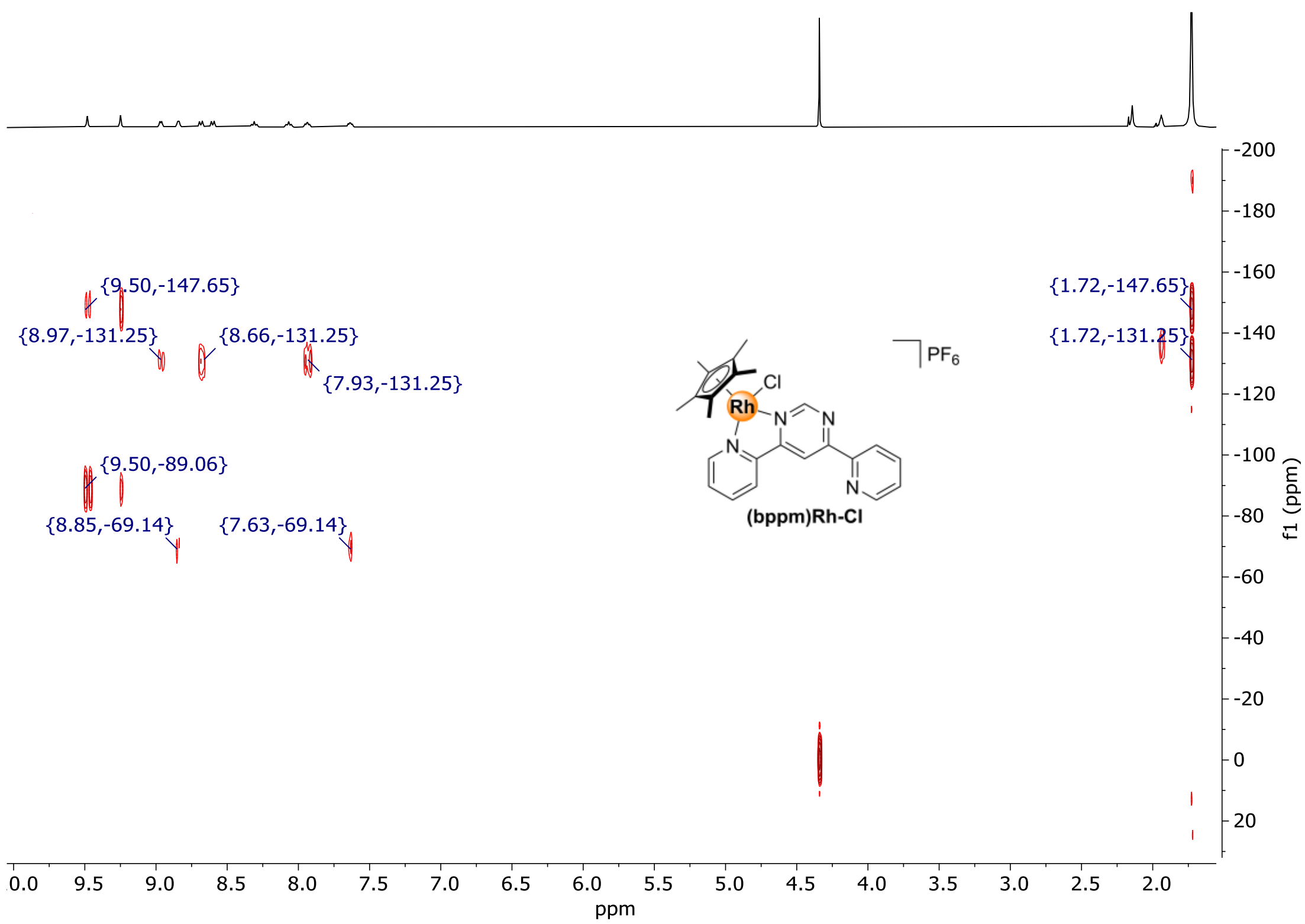

Figure S9. ^{1}H-^{15}N HMBC spectrum (^{1}H: 400 MHz, ^{15}N: 41 MHz; CD_3CN) for **(bppm)Rh-Cl**.

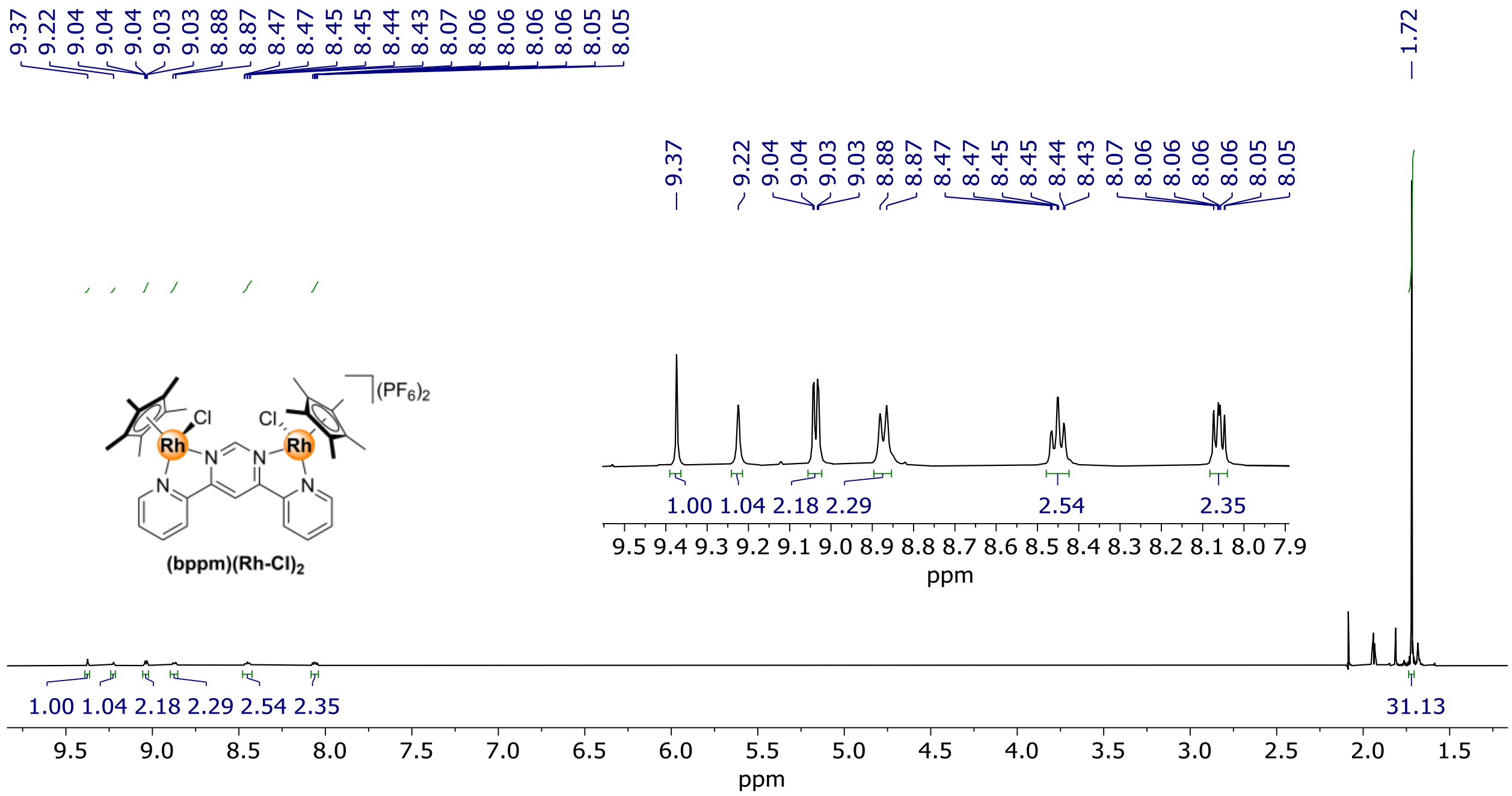

Figure S10. ^{1}H NMR (500 MHz, CD_3CN) of **(bppm)(Rh-Cl)$_2$**. Inset: enlarged view of aromatic region.

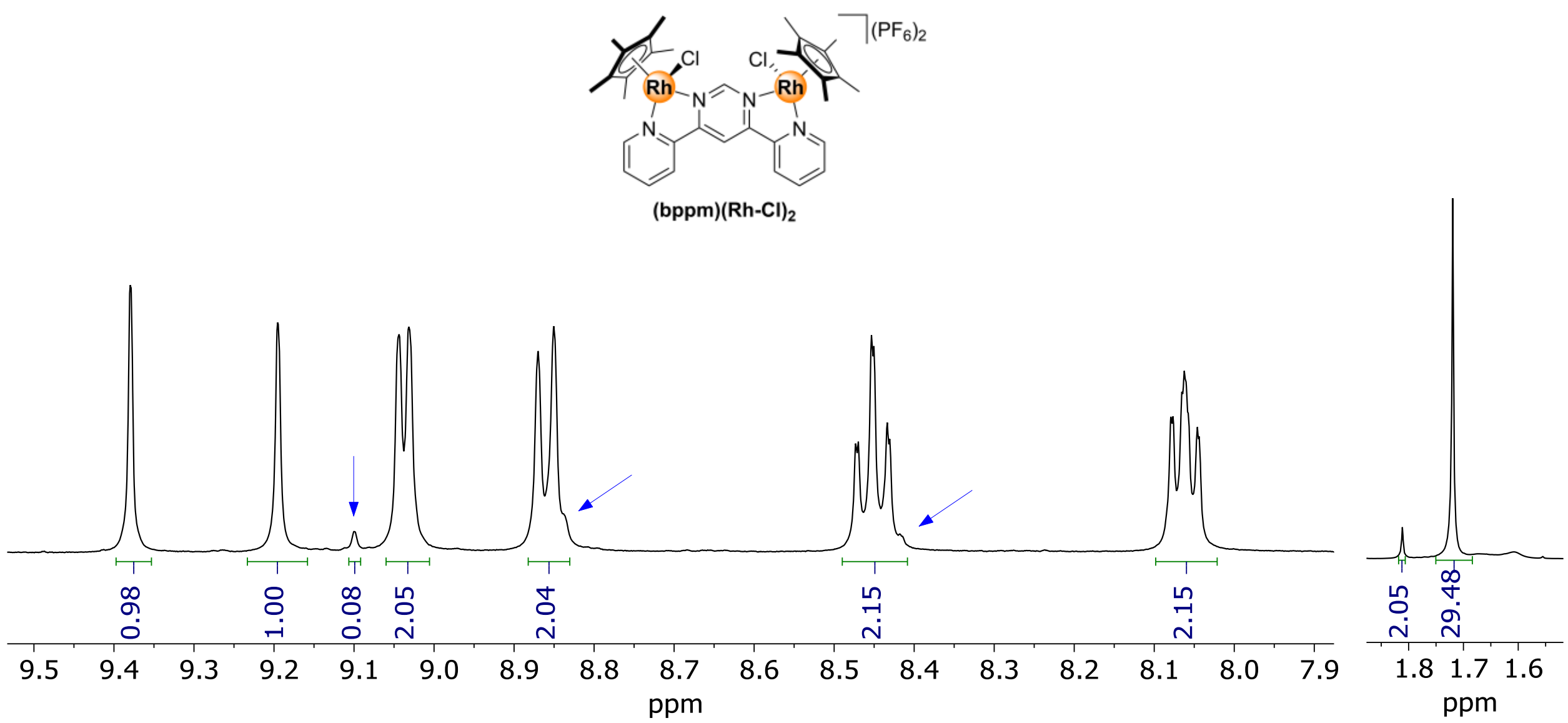

Figure S11. ^{1}H NMR (500 MHz, CD_3CN) of **(bppm)(Rh-Cl)$_2$** highlighting the signals corresponding to the minor diastereomer (blue arrows). The diasteromeric ratio is ~15:1.

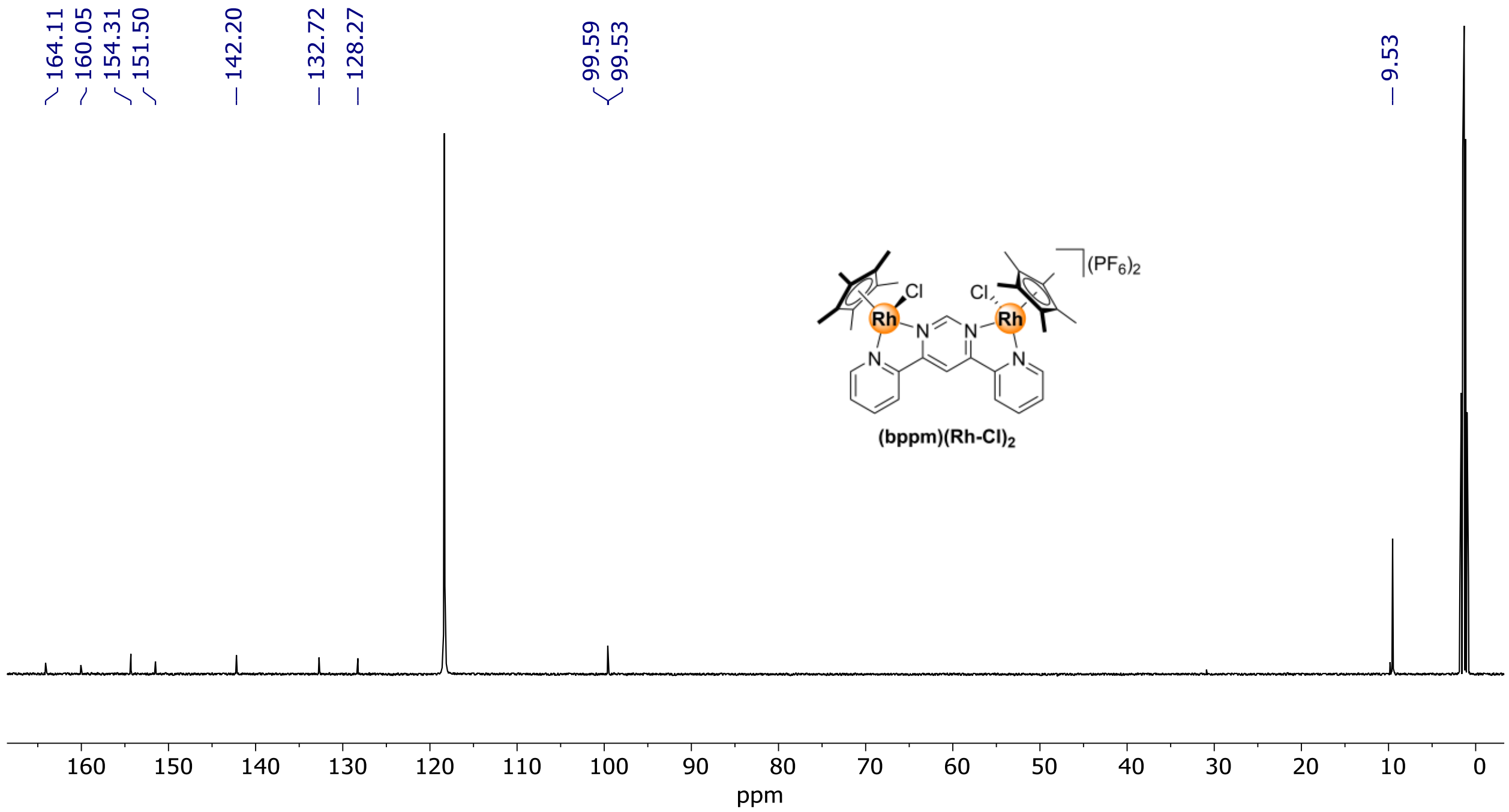

Figure S12. $^{13}C\{^{1}H\}$ NMR spectrum (125 MHz, CD_3CN) of **(bppm)(Rh-Cl)₂**.

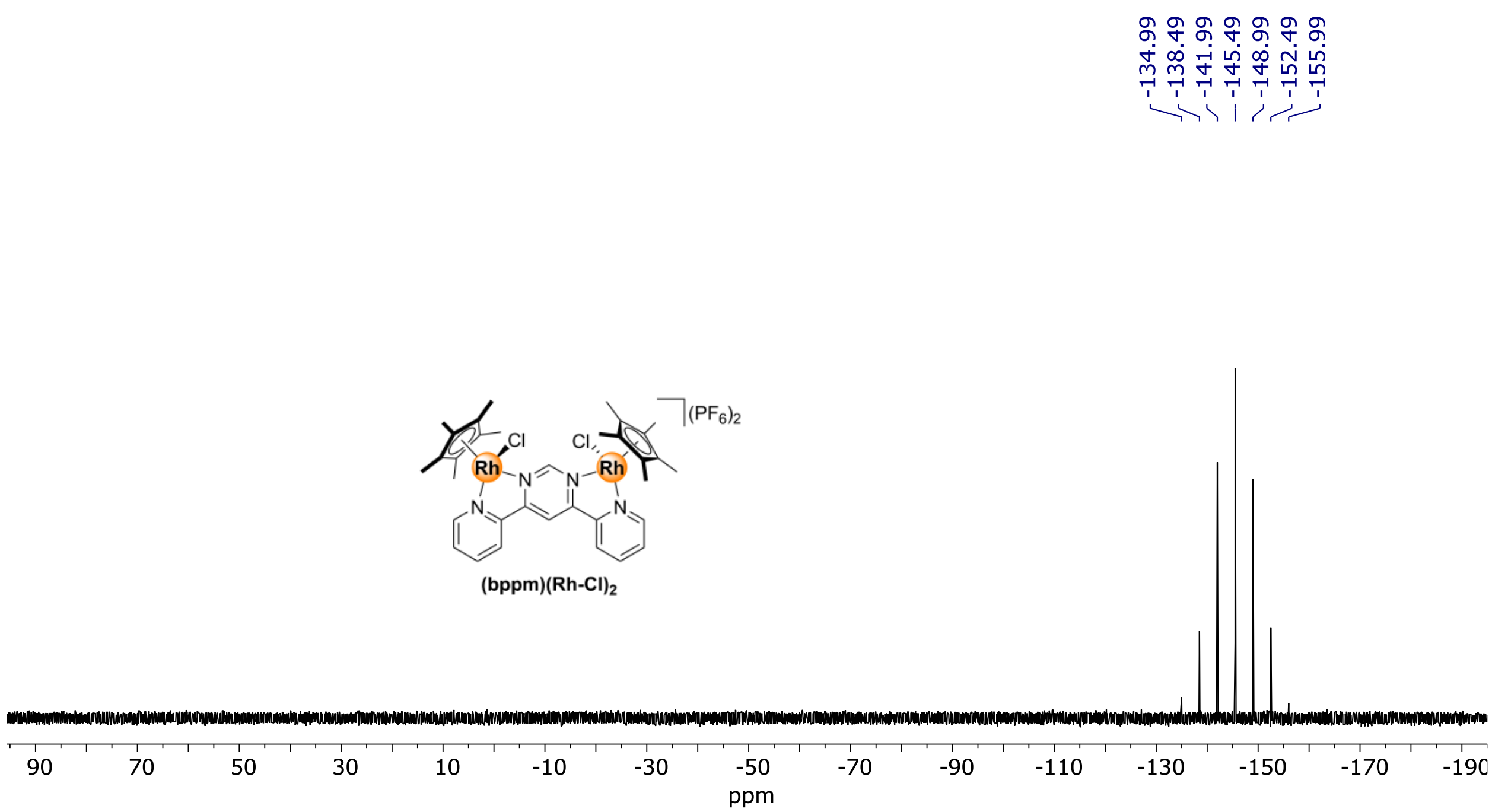

Figure S13. $^{31}P\{^{1}H\}$ NMR spectrum (202 MHz, CD_3CN) of **(bppm)(Rh-Cl)₂**.

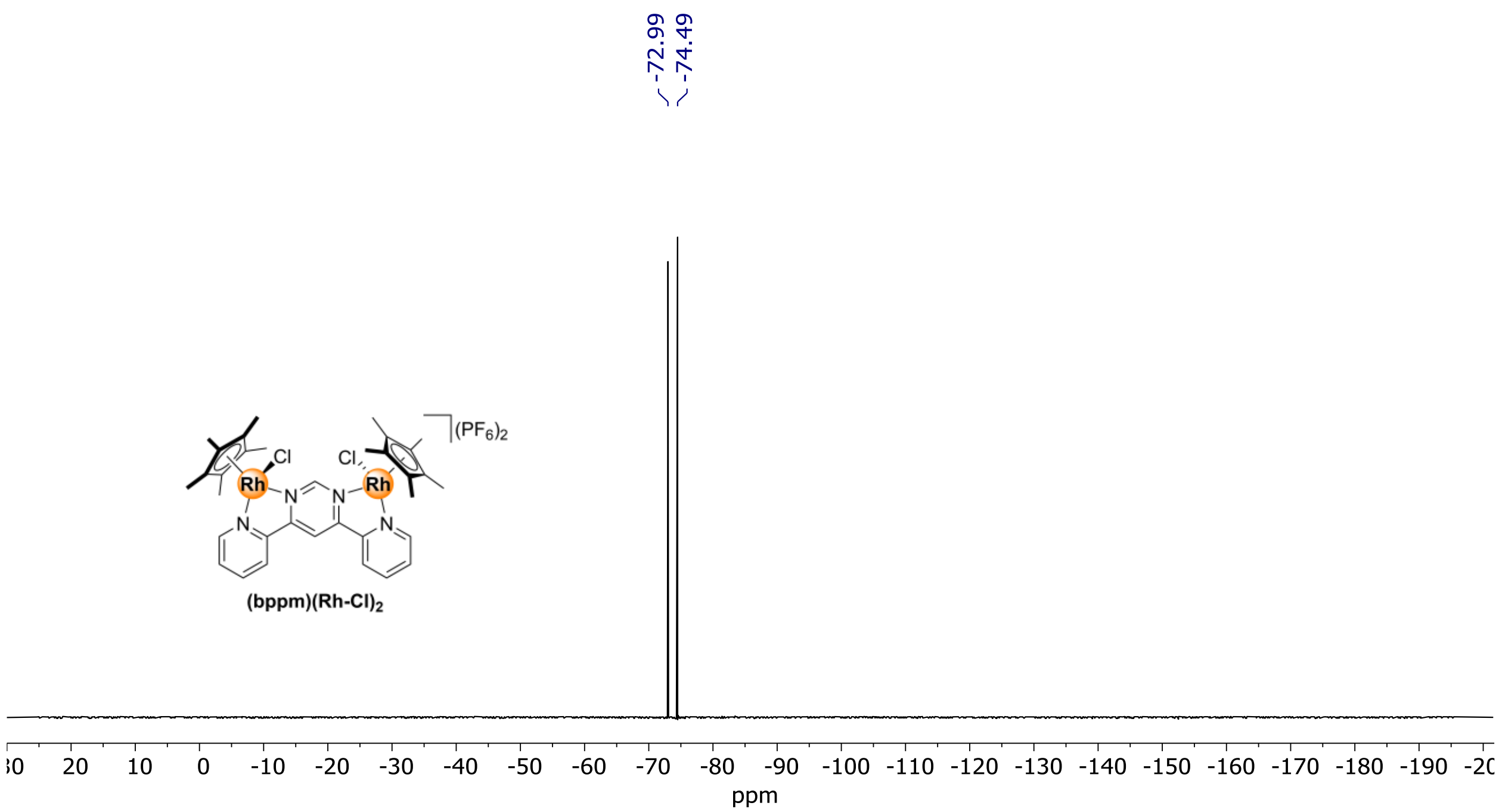

Figure S14. ^{19}F NMR spectrum (469 MHz, CD_3CN) of **(bppm)(Rh-Cl)$_2$**.

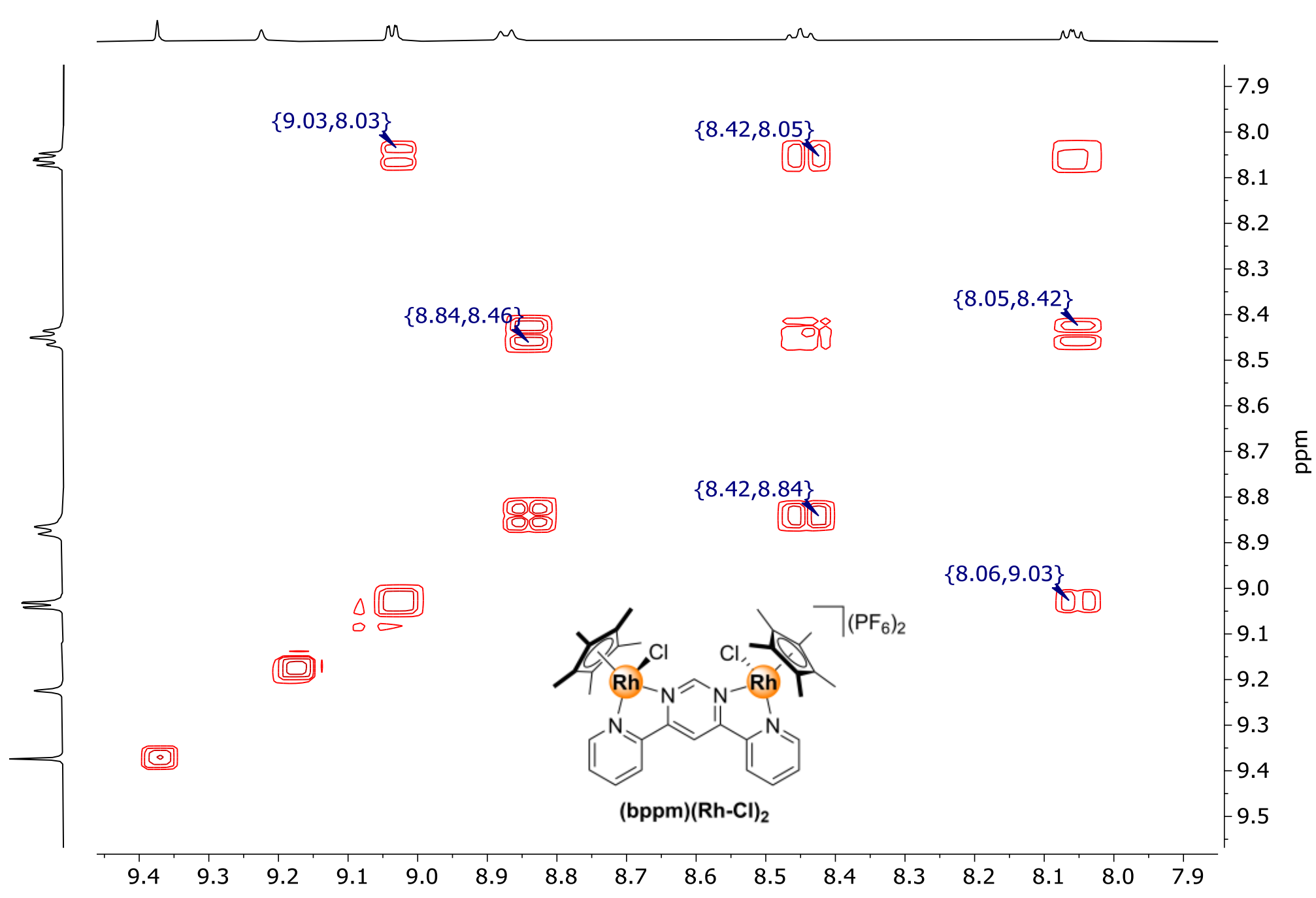

Figure S15. Partial COSY spectrum (500 MHz, CD_3CN) of **(bppm)(Rh-Cl)$_2$**.

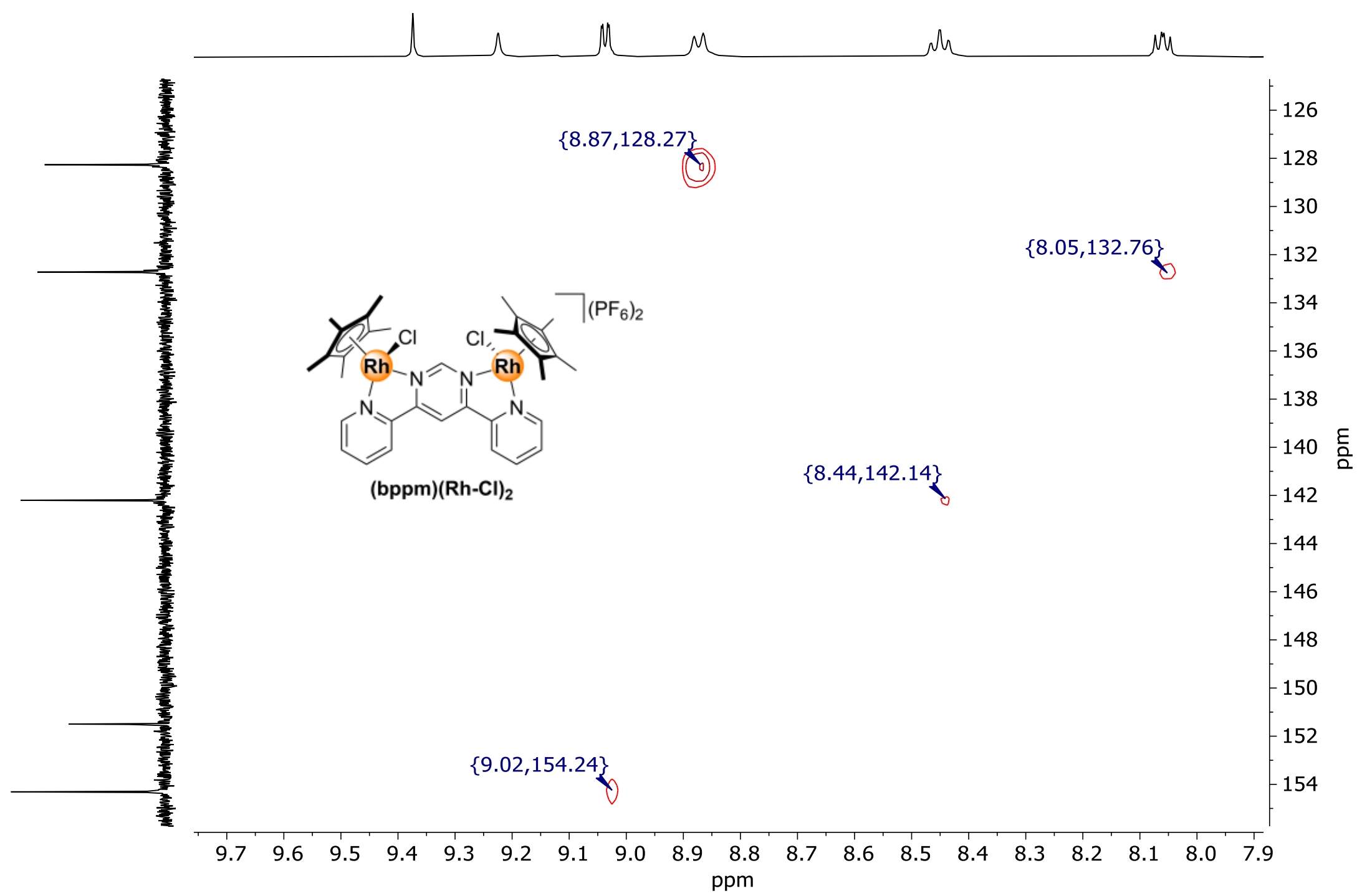

Figure S16. Partial ^{1}H-^{13}C HSQC spectrum (^{1}H: 500 MHz, ^{13}C: 125 MHz, CD_3CN) of **(bppm)(Rh-Cl)$_2$**.

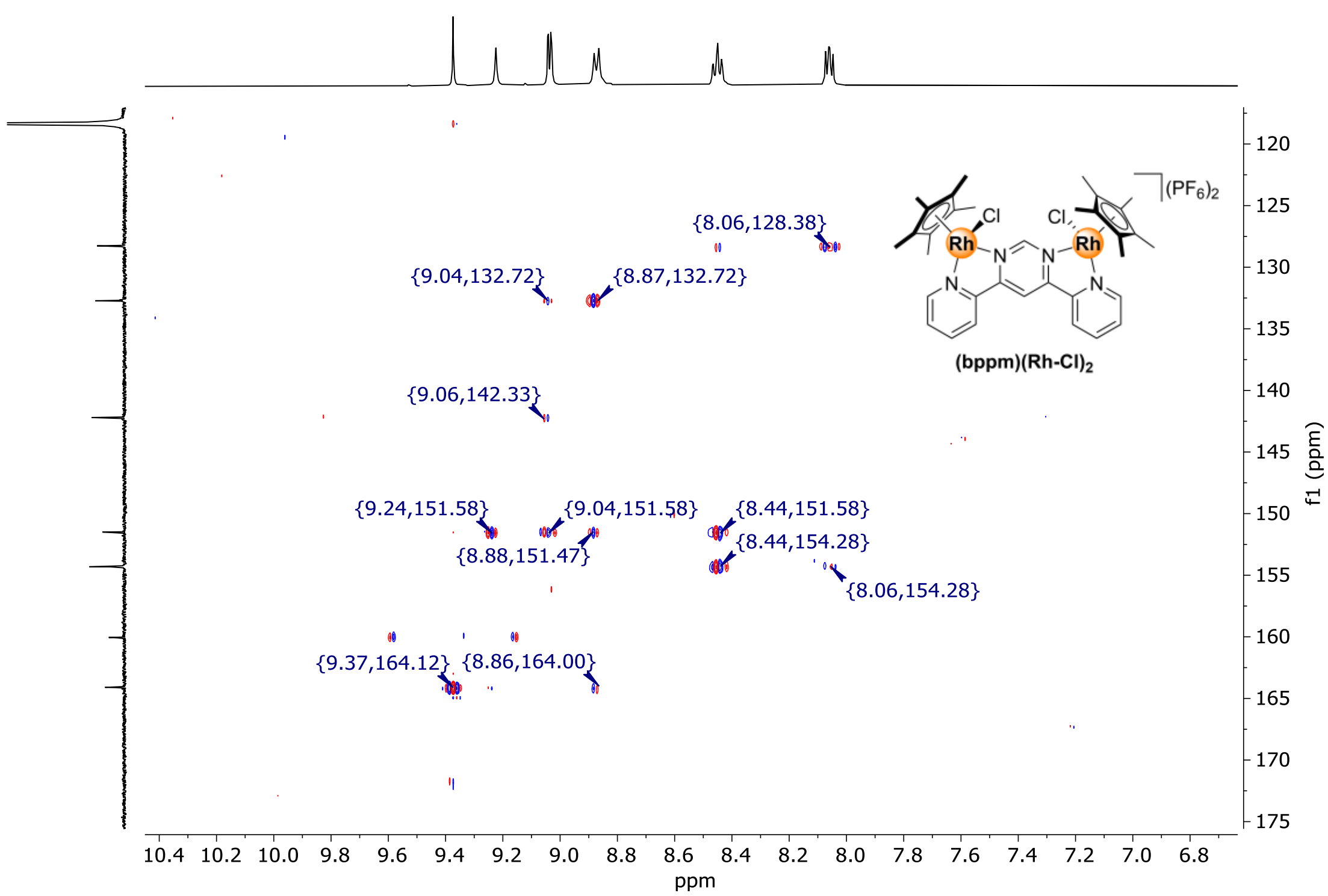

Figure S17. Partial ^{1}H-^{13}C HMBC spectrum (^{1}H: 500 MHz, ^{13}C: 125 MHz, CD_3CN) of **(bppm)(Rh-Cl)$_2$**.

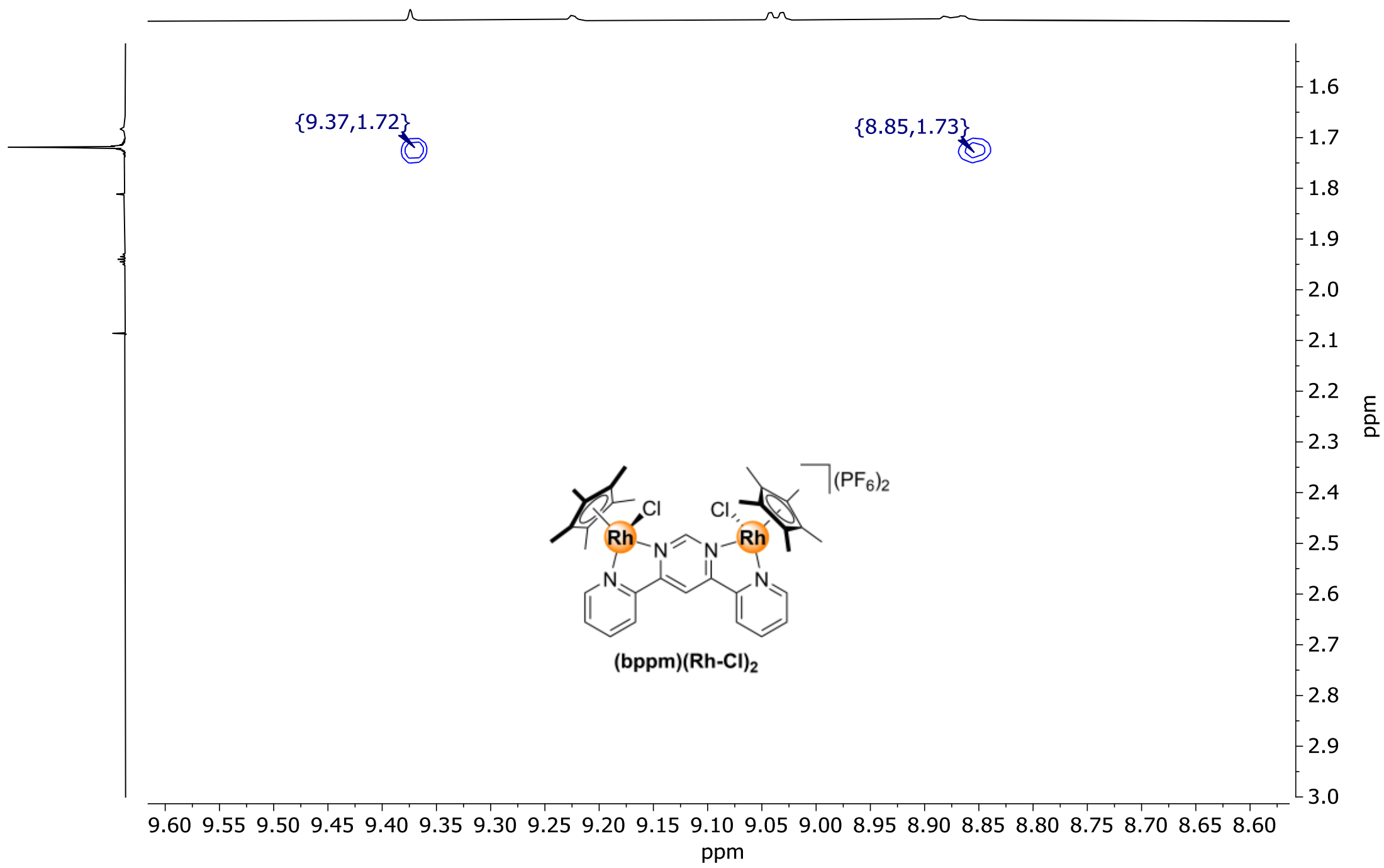

Figure S18. Partial NOESY spectrum (500MHz, CD_3CN) of **(bppm)(Rh-Cl)$_2$**.

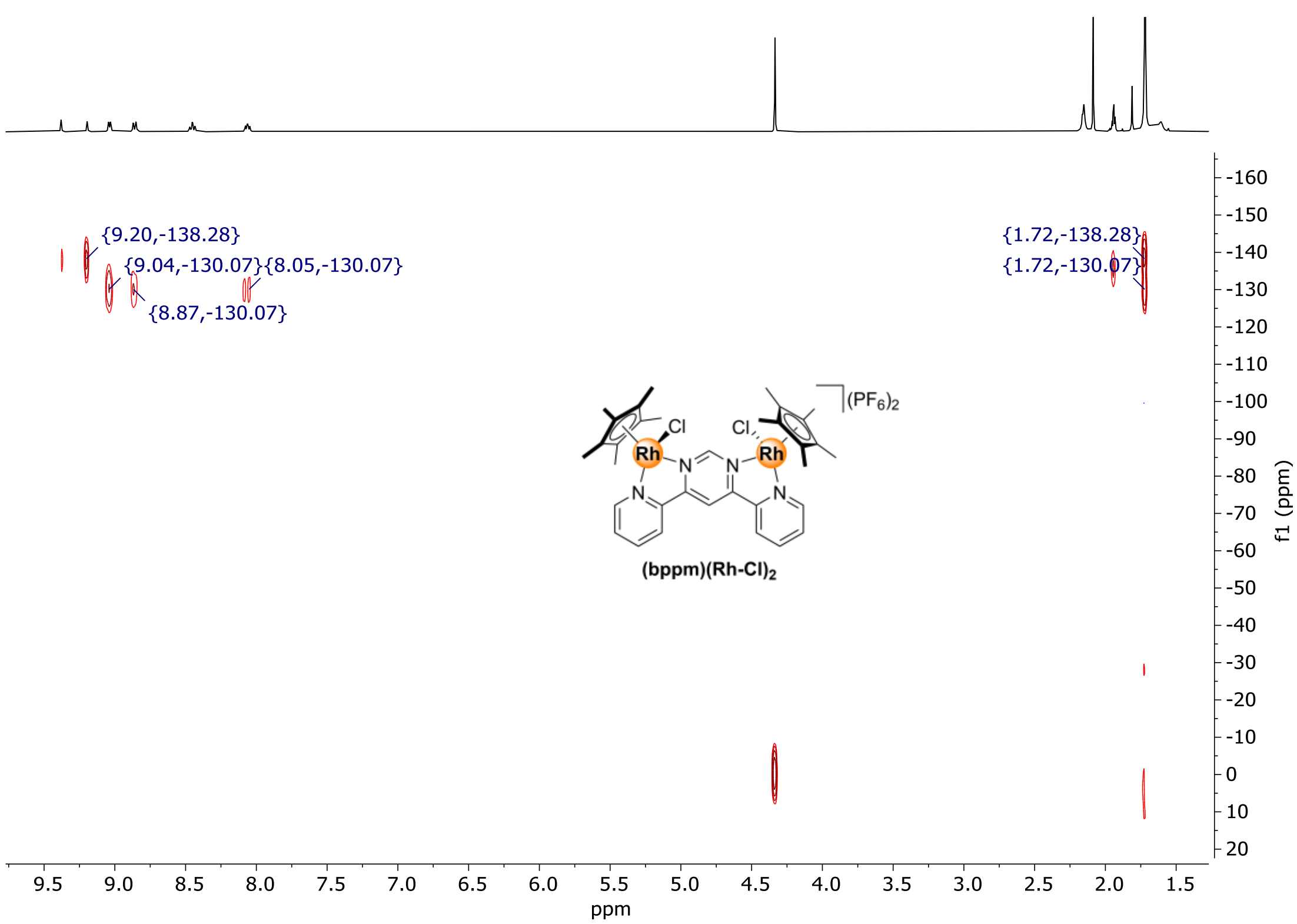

Figure S19. ^1H-^{15}N HMBC spectrum (^1H: 400 MHz, ^{15}N: 41 MHz; CD_3CN) for **(bppm)(Rh-Cl)$_2$**.

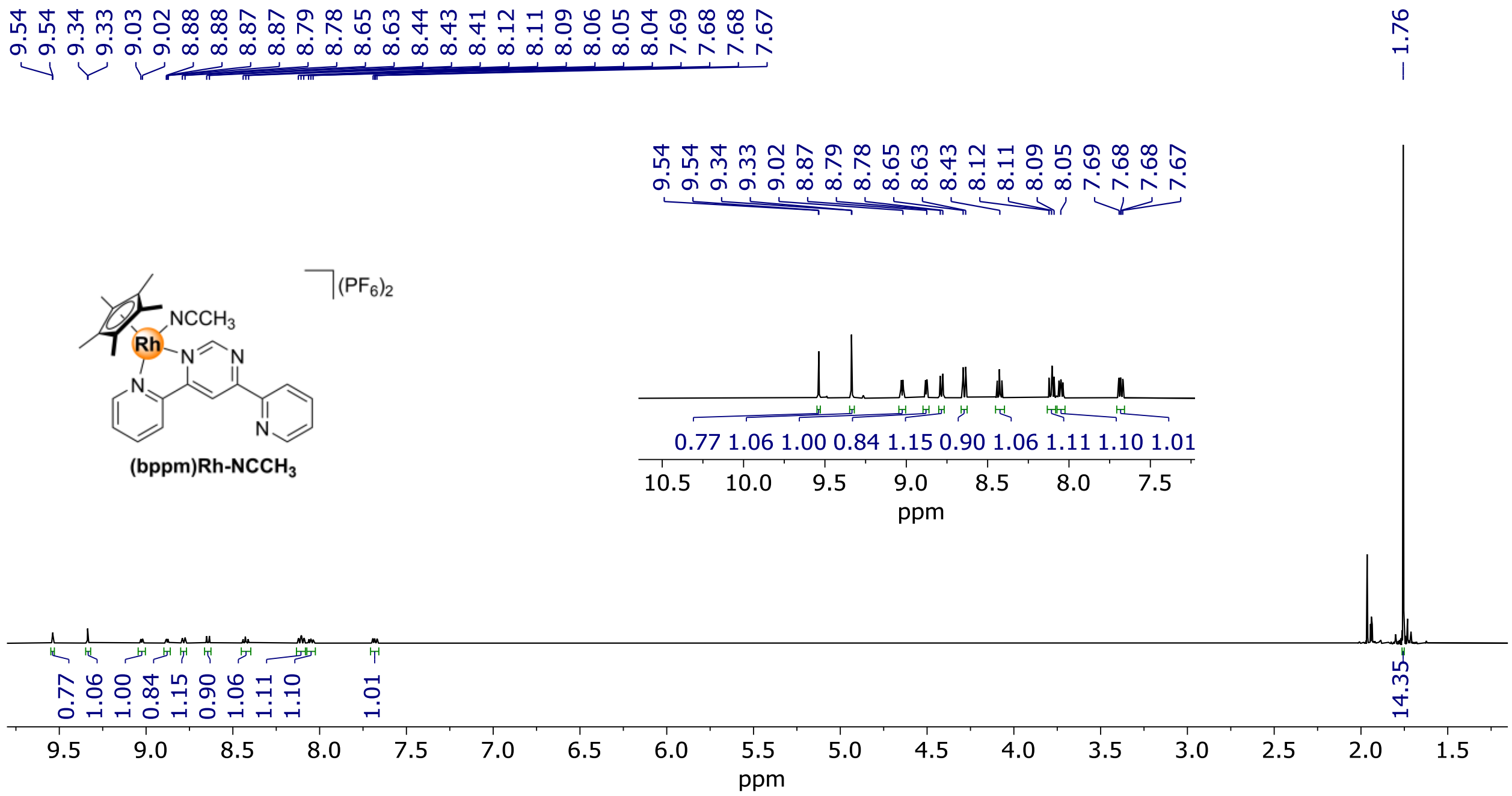

Figure S20. ^{1}H NMR (500 MHz, CD_3CN) of **(bppm)Rh-NCCH$_3$**. Inset: enlarged view of aromatic region.

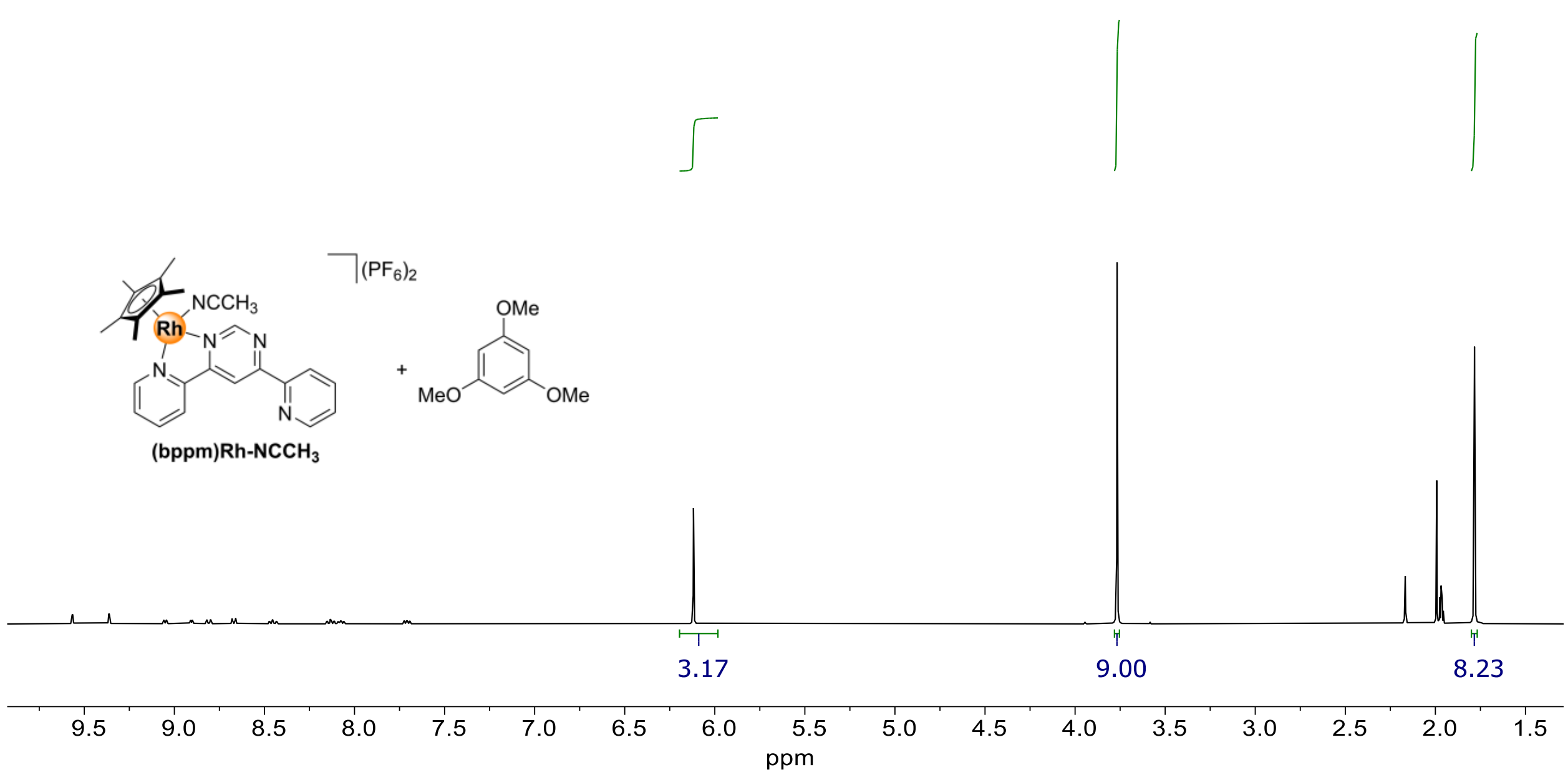

Figure S21. ^{1}H NMR (400 MHz, CD_3CN) of **(bppm)Rh-NCCH$_3$** (21.6 m*M*) in the presence of 1,3,5-trimethoxybenzene (38.4 m*M*). The integration values indicate >95% purity.

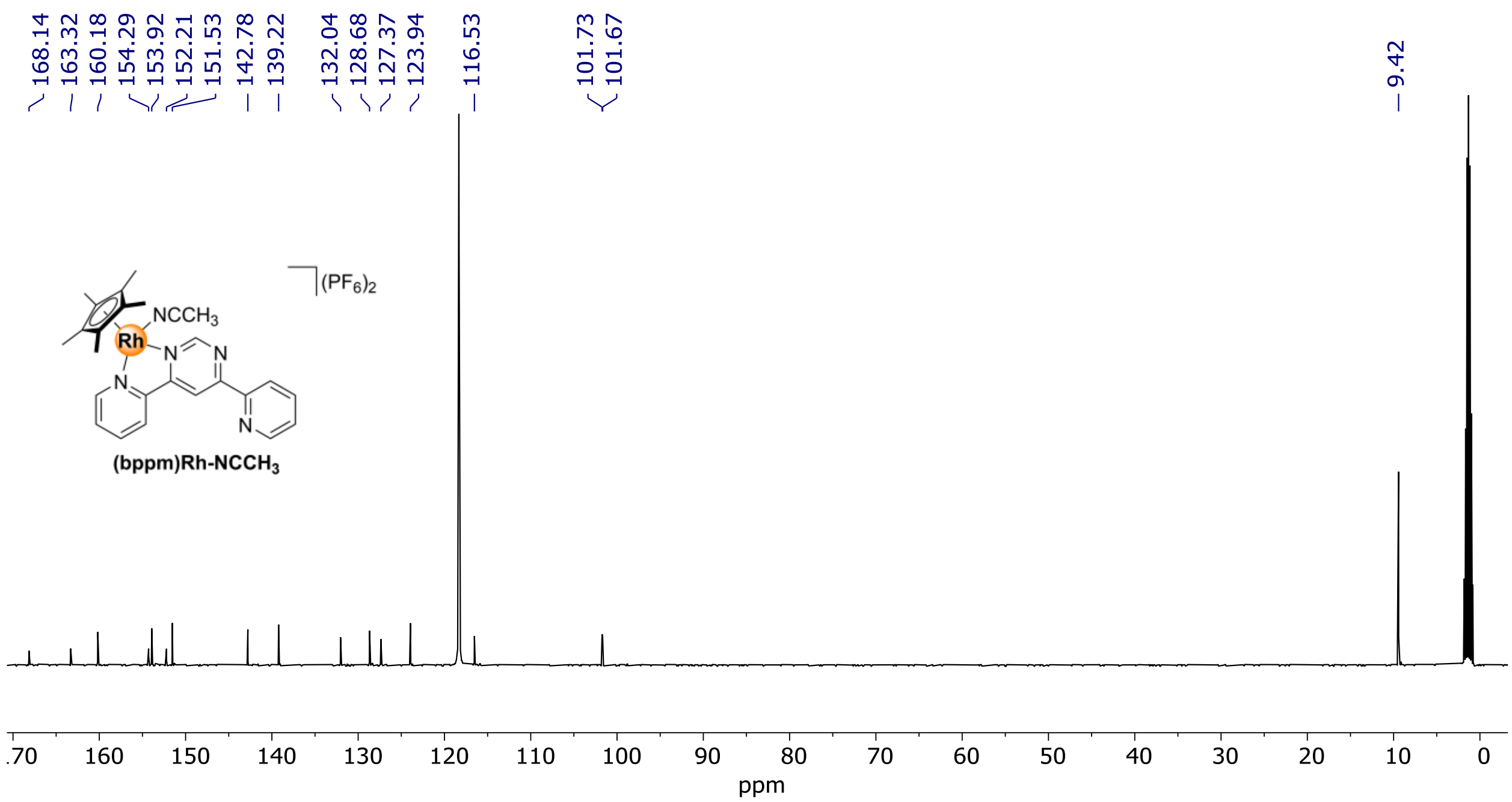

Figure S22. $^{13}C\{^{1}H\}$ NMR spectrum (125 MHz, CD_3CN) of **(bppm)Rh-NCCH$_3$**.

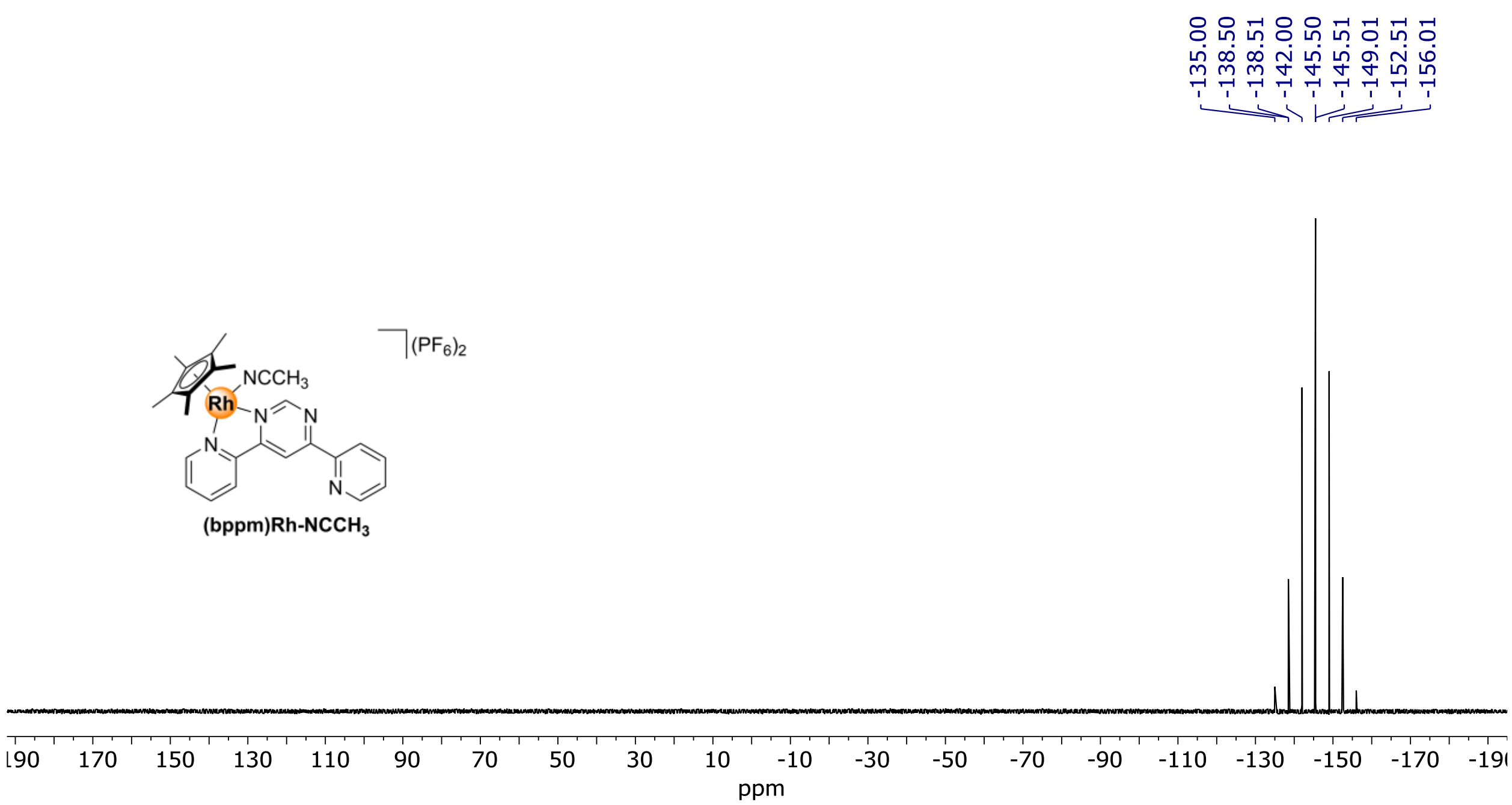

Figure S23. $^{31}P\{^{1}H\}$ NMR spectrum (202 MHz, CD_3CN) of **(bppm)Rh-NCCH$_3$**.

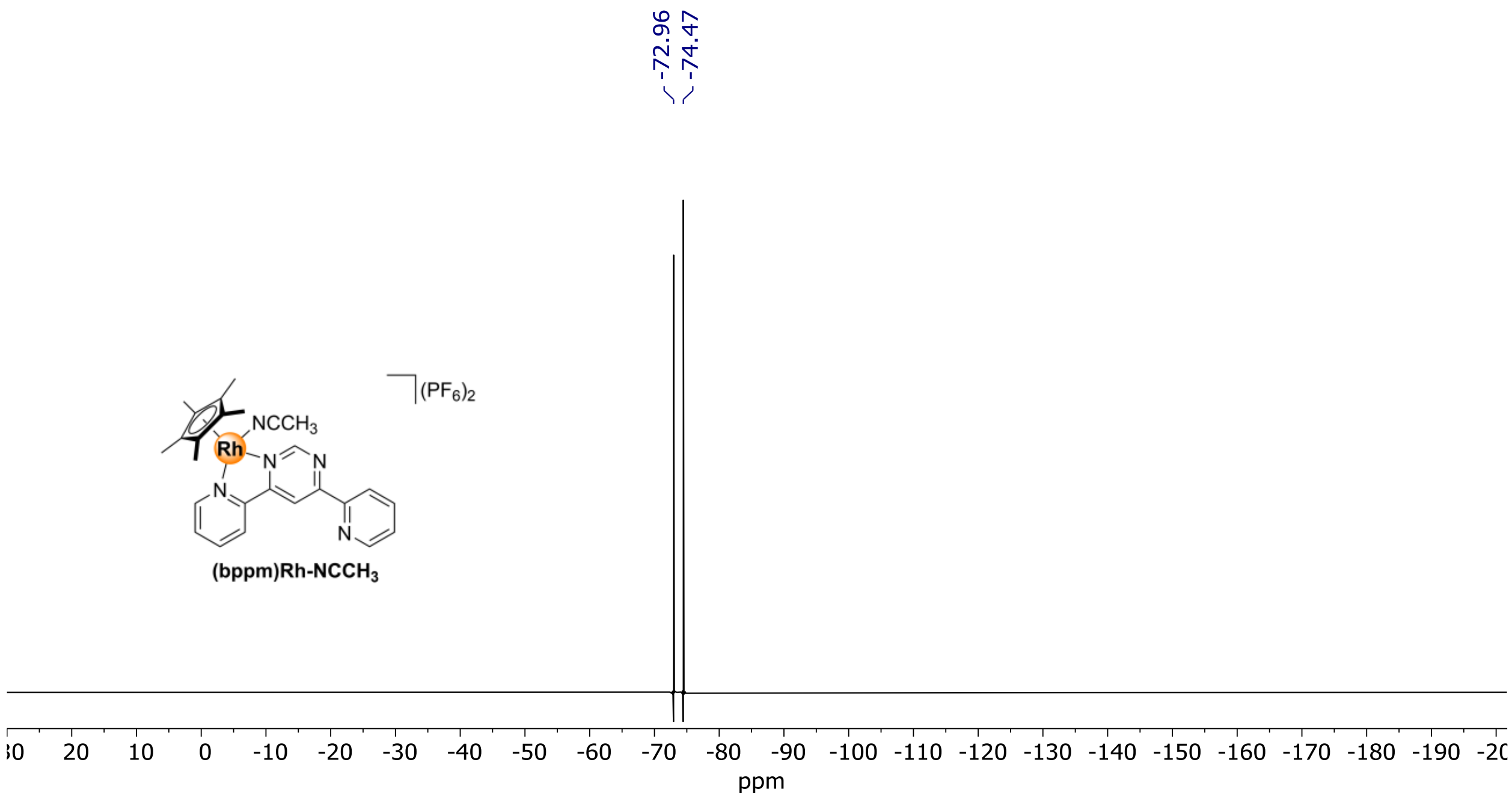

Figure S24. ^{19}F NMR spectrum (469 MHz, CD_3CN) of **(bppm)Rh-NCCH$_3$**.

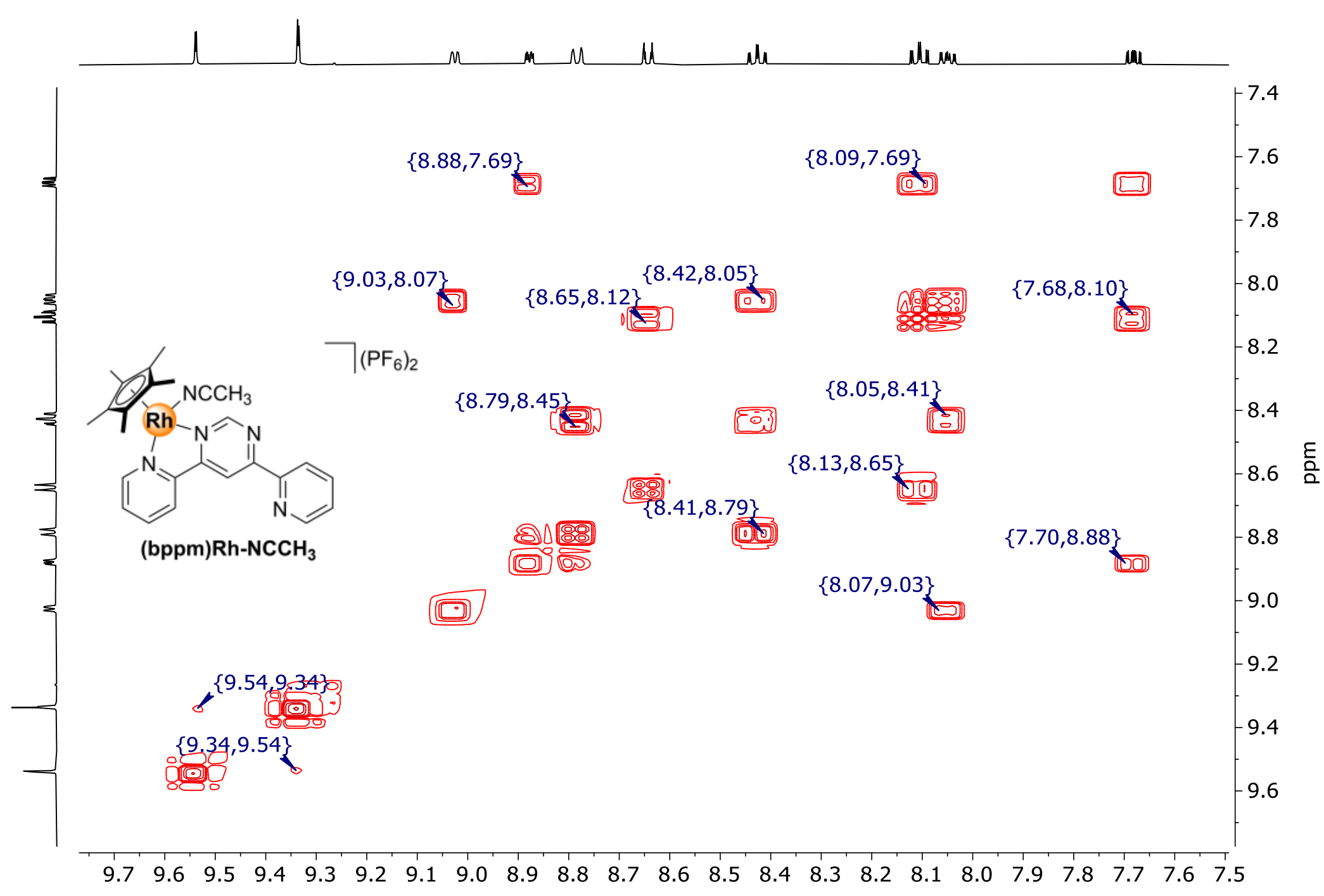

Figure S25. Partial COSY spectrum (500 MHz, CD_3CN) of **(bppm)Rh-NCCH$_3$**.

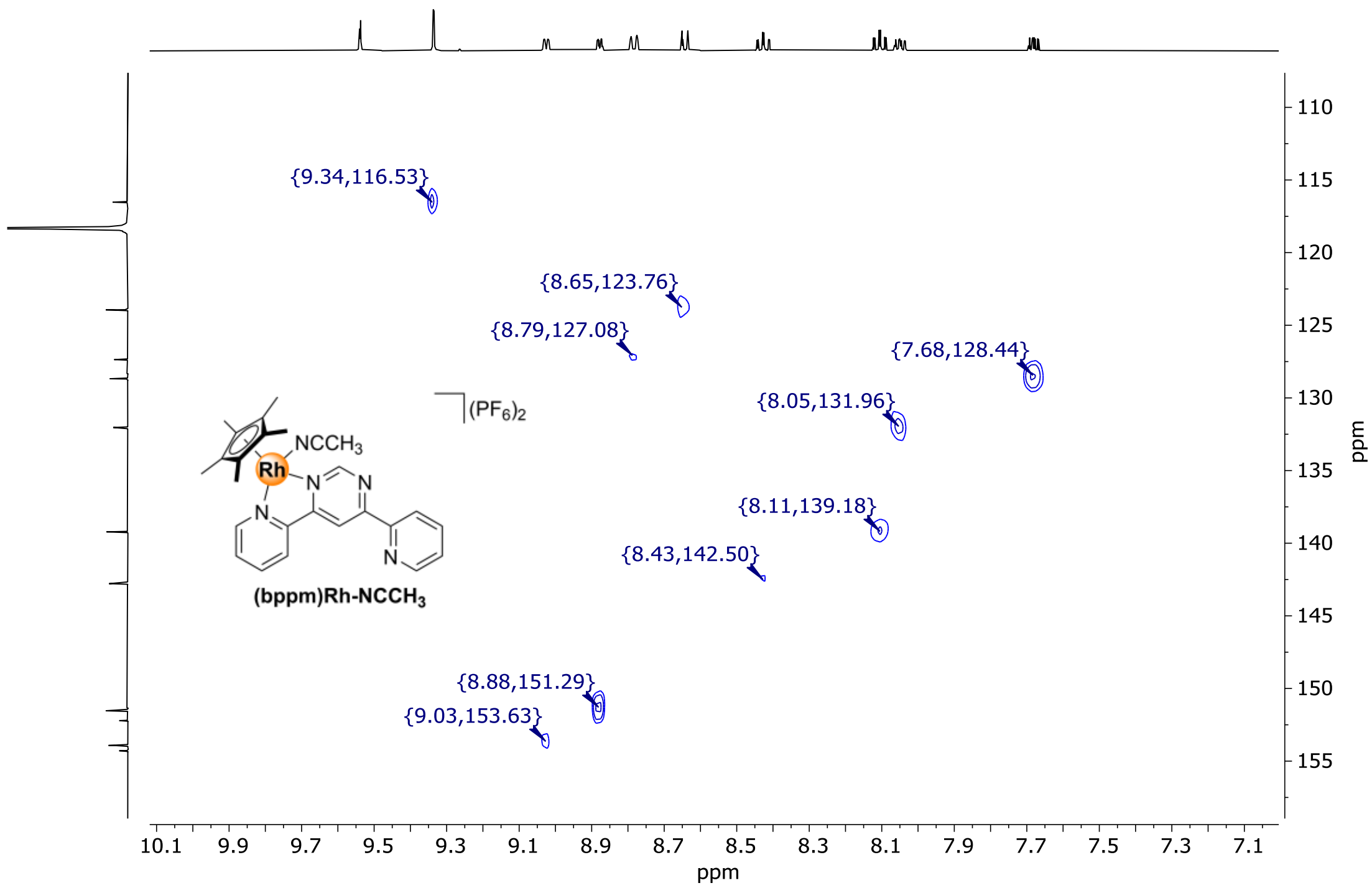

Figure S26. Partial ^{1}H-^{13}C HSQC spectrum (^{1}H: 500 MHz, ^{13}C: 125 MHz, CD_3CN) of **(bppm)Rh-NCCH$_3$**.

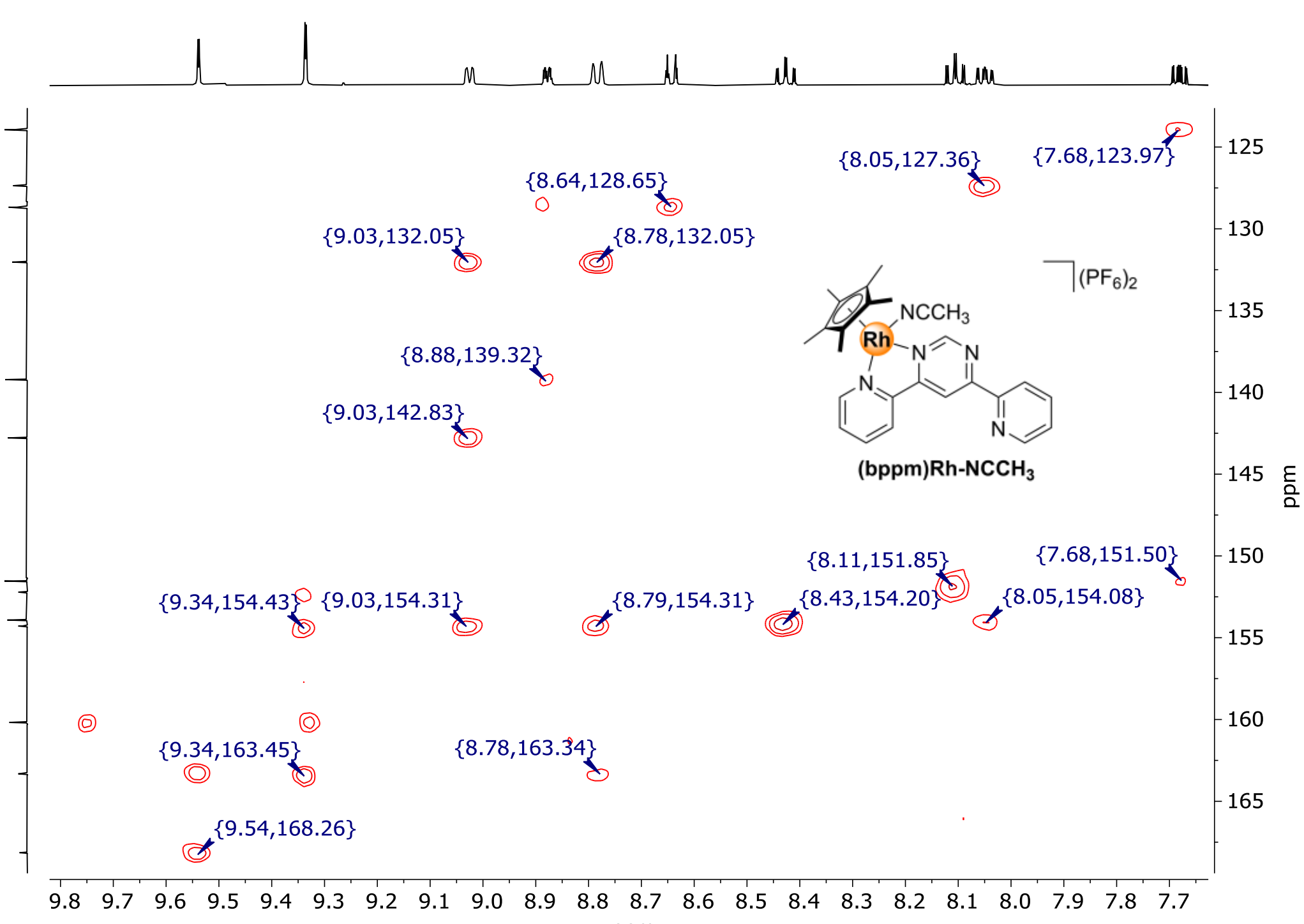

Figure S27. Partial ^{1}H-^{13}C HMBC spectrum (^{1}H: 500 MHz, ^{13}C: 125 MHz, CD_3CN) of **(bppm)Rh-NCCH$_3$**.

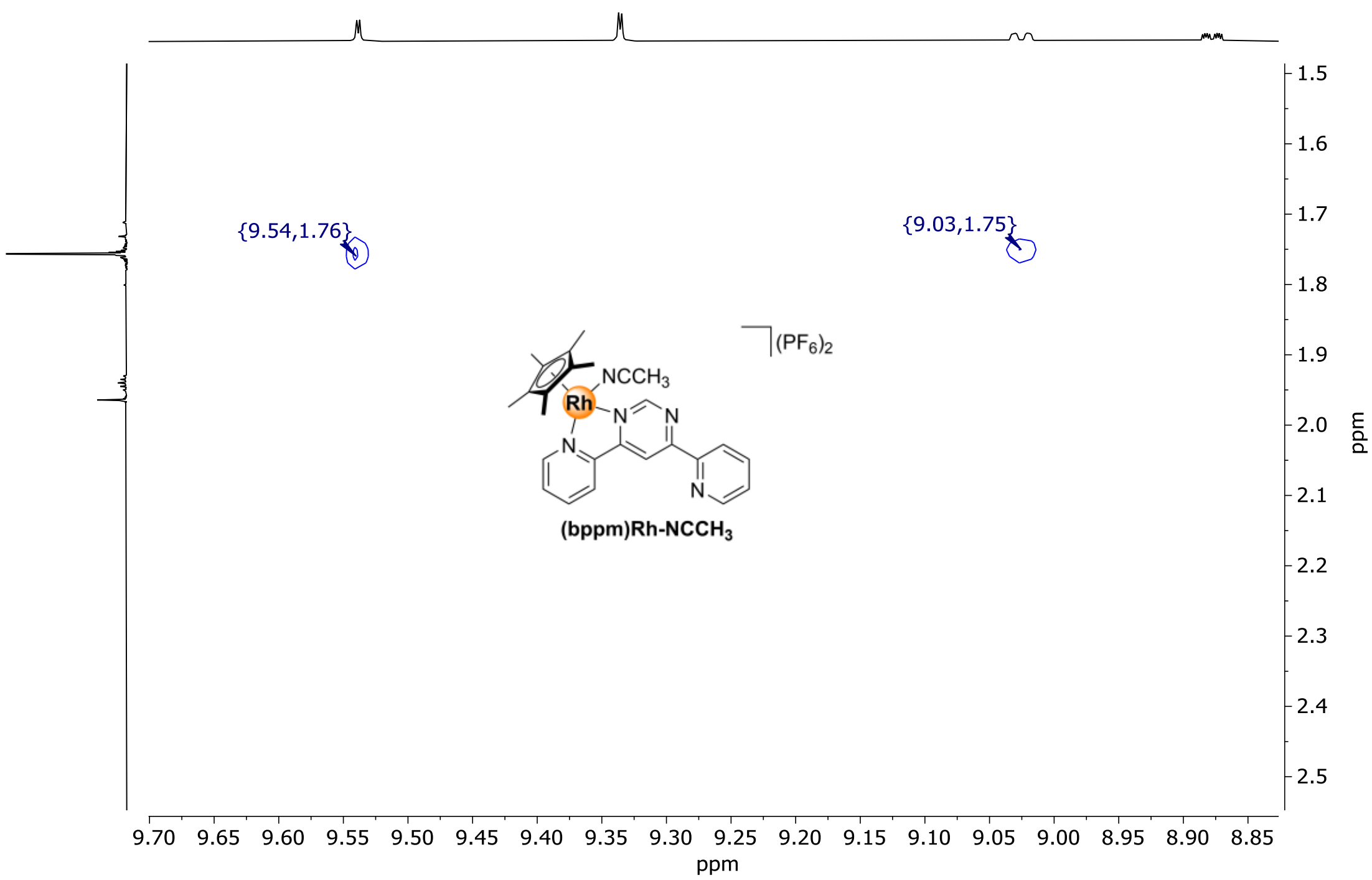

Figure S28. Partial NOESY spectrum (500MHz, CD_3CN) of **(bppm)Rh-NCCH$_3$**.

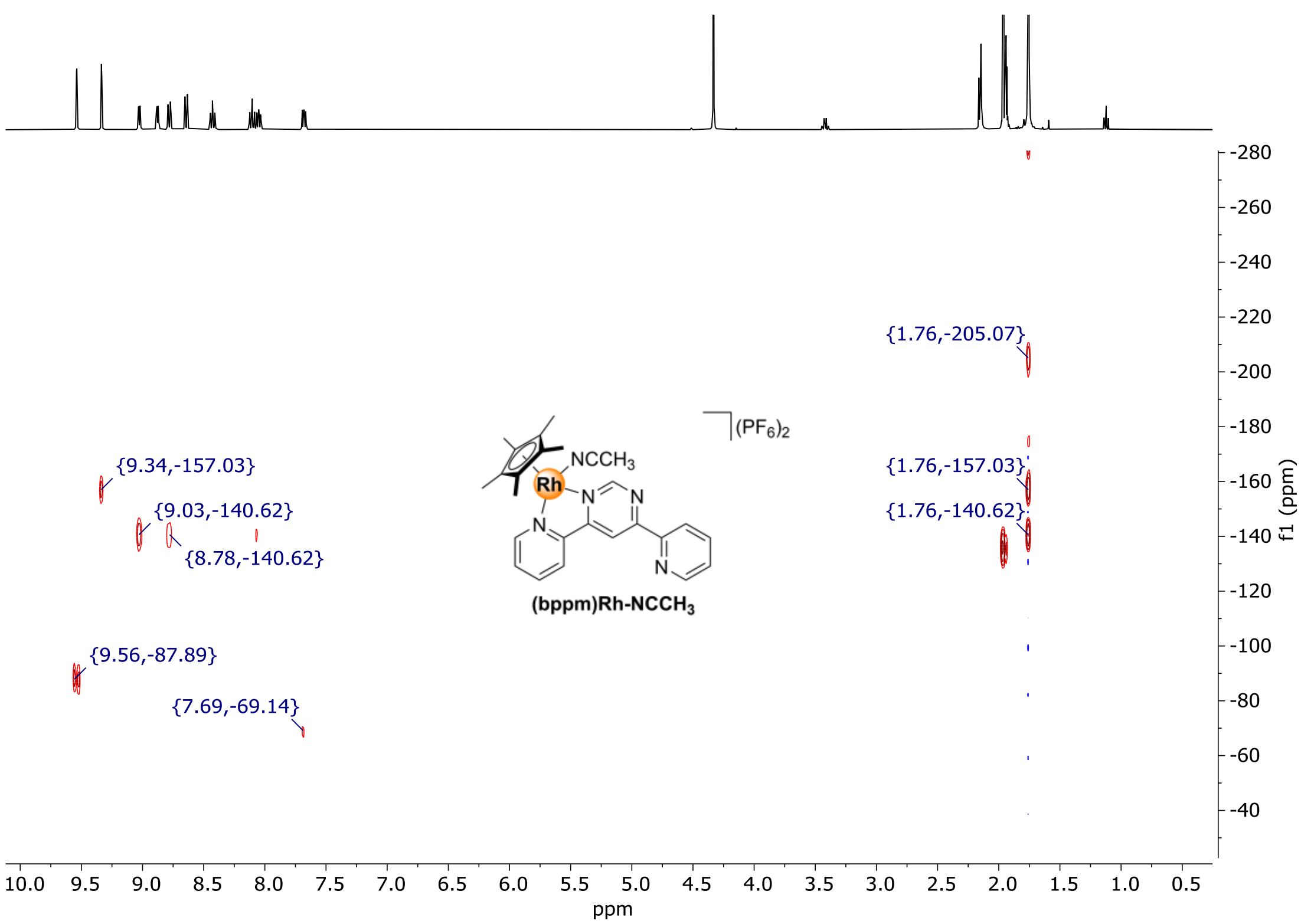

Figure S29. ^1H-^{15}N HMBC spectrum (^1H: 400 MHz, ^{15}N: 41 MHz; CD_3CN) for **(bppm)Rh-NCCH$_3$**.

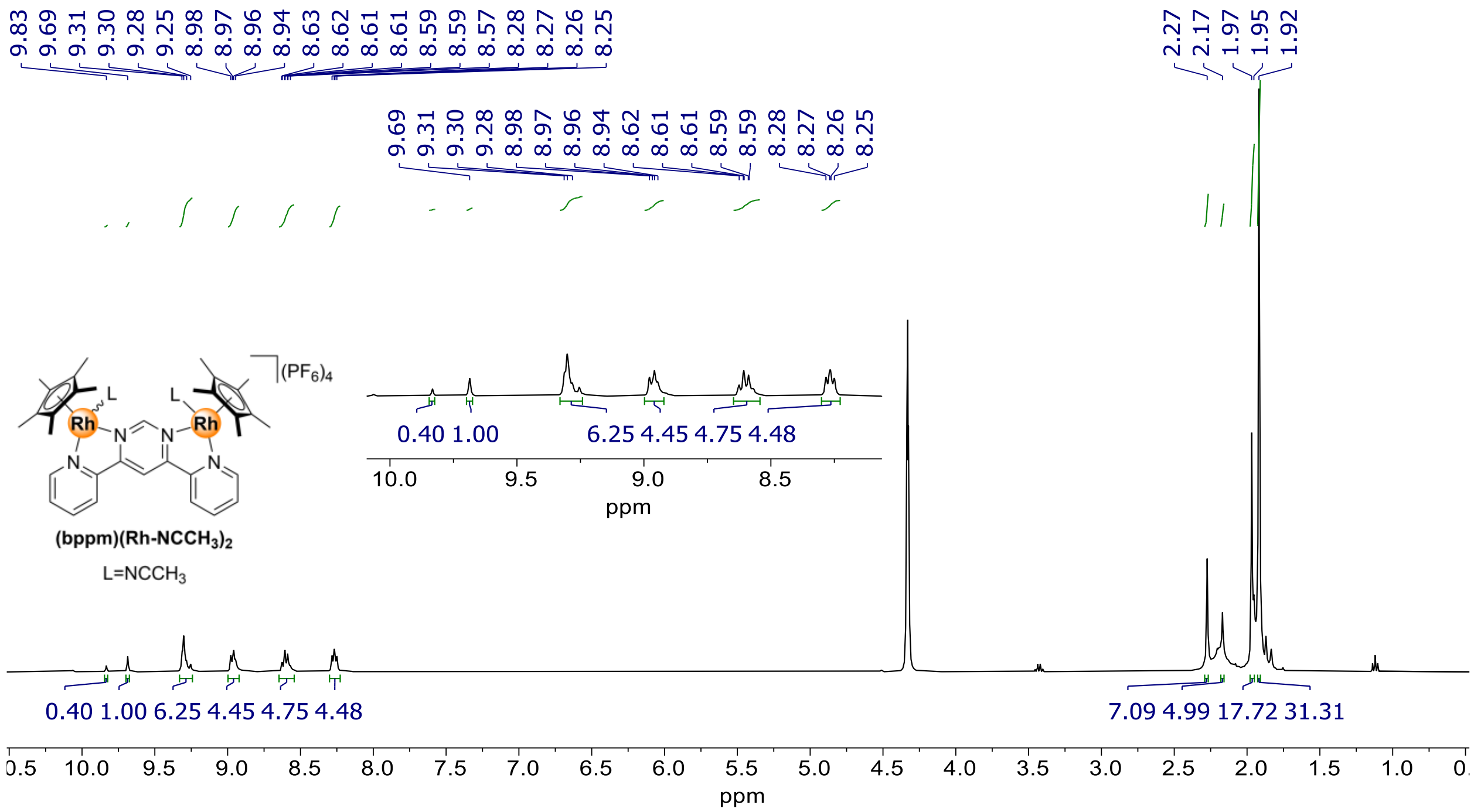

Figure S30. ^{1}H NMR (400 MHz, CD_3NO_2) of **(bppm)(Rh-NCCH$_3$)$_2$**. Inset: enlarged view of aromatic region.

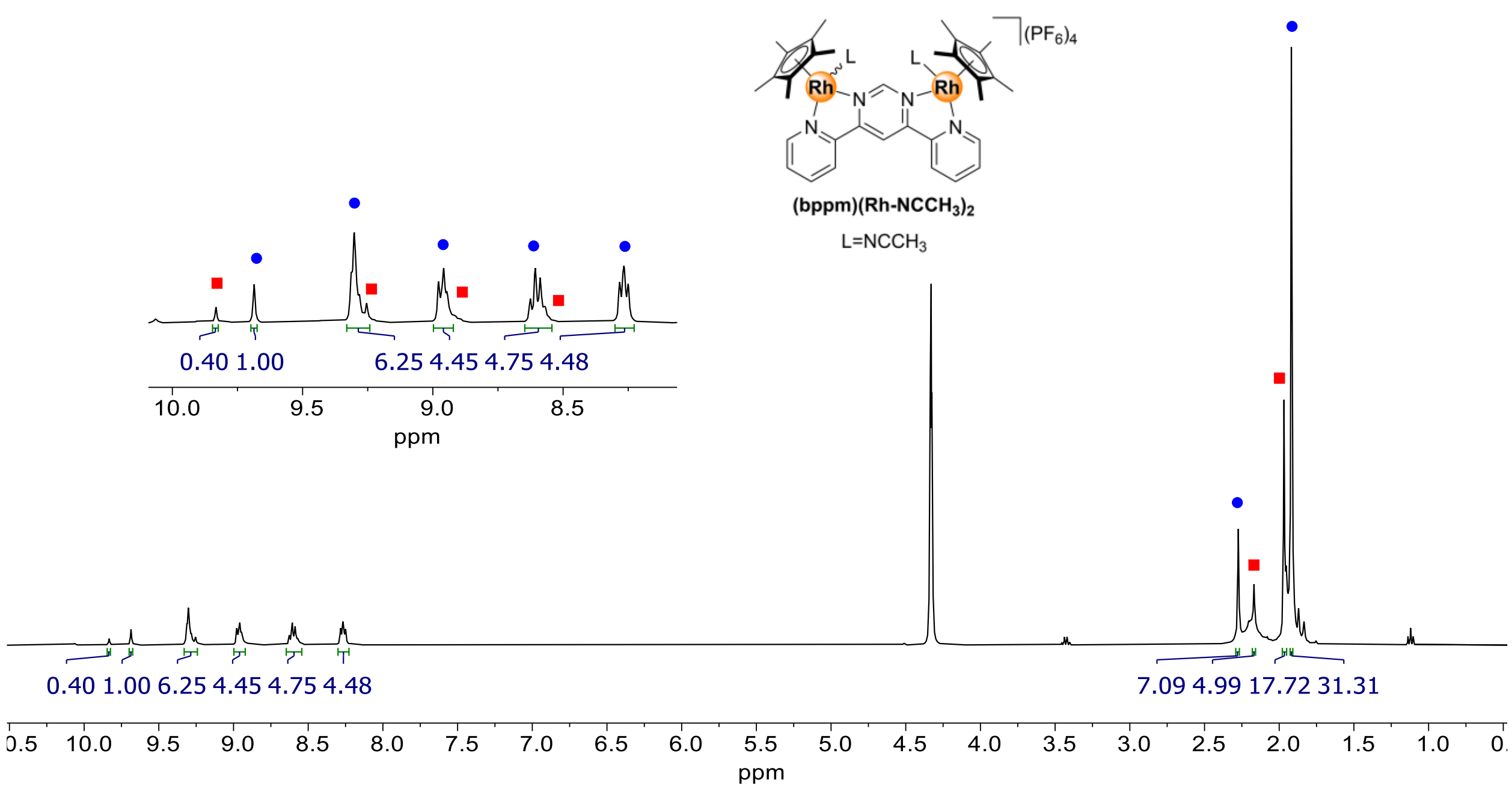

Figure S31. ^{1}H NMR spectrum (400 MHz, CD_3NO_2) of **(bppm)(Rh-NCCH$_3$)$_2$**; yhe peaks labeled with blue circles and red squares are consistent with two species with the general formula $[\{Cp^*Rh(NCCH_3)\}(bppm)]^{4+}$ in a ~2:1 ratio.

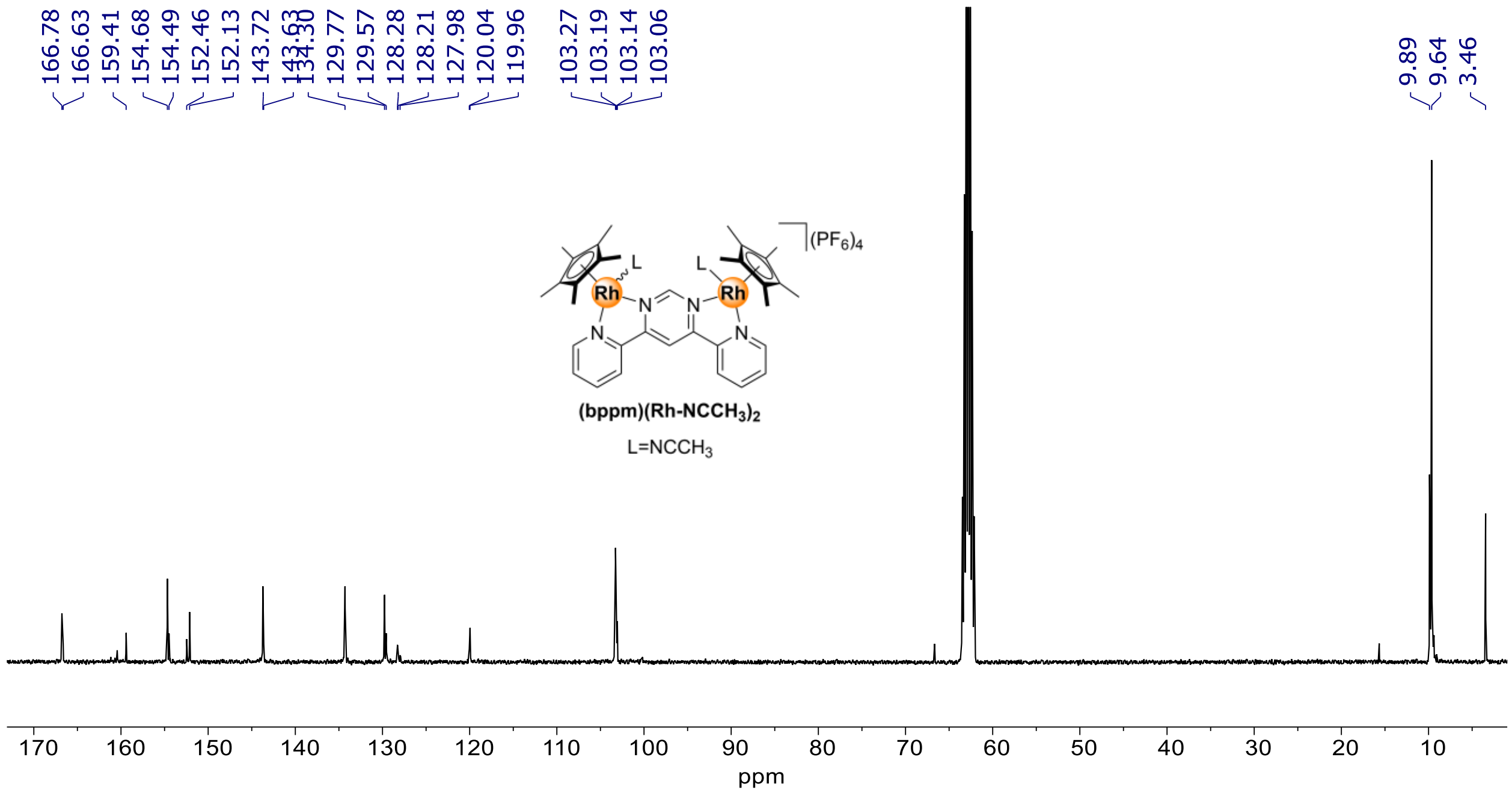

Figure S32. $^{13}C\{^1H\}$ NMR spectrum (101 MHz, CD_3NO_2) of **(bppm)(Rh-NCCH$_3$)$_2$**.

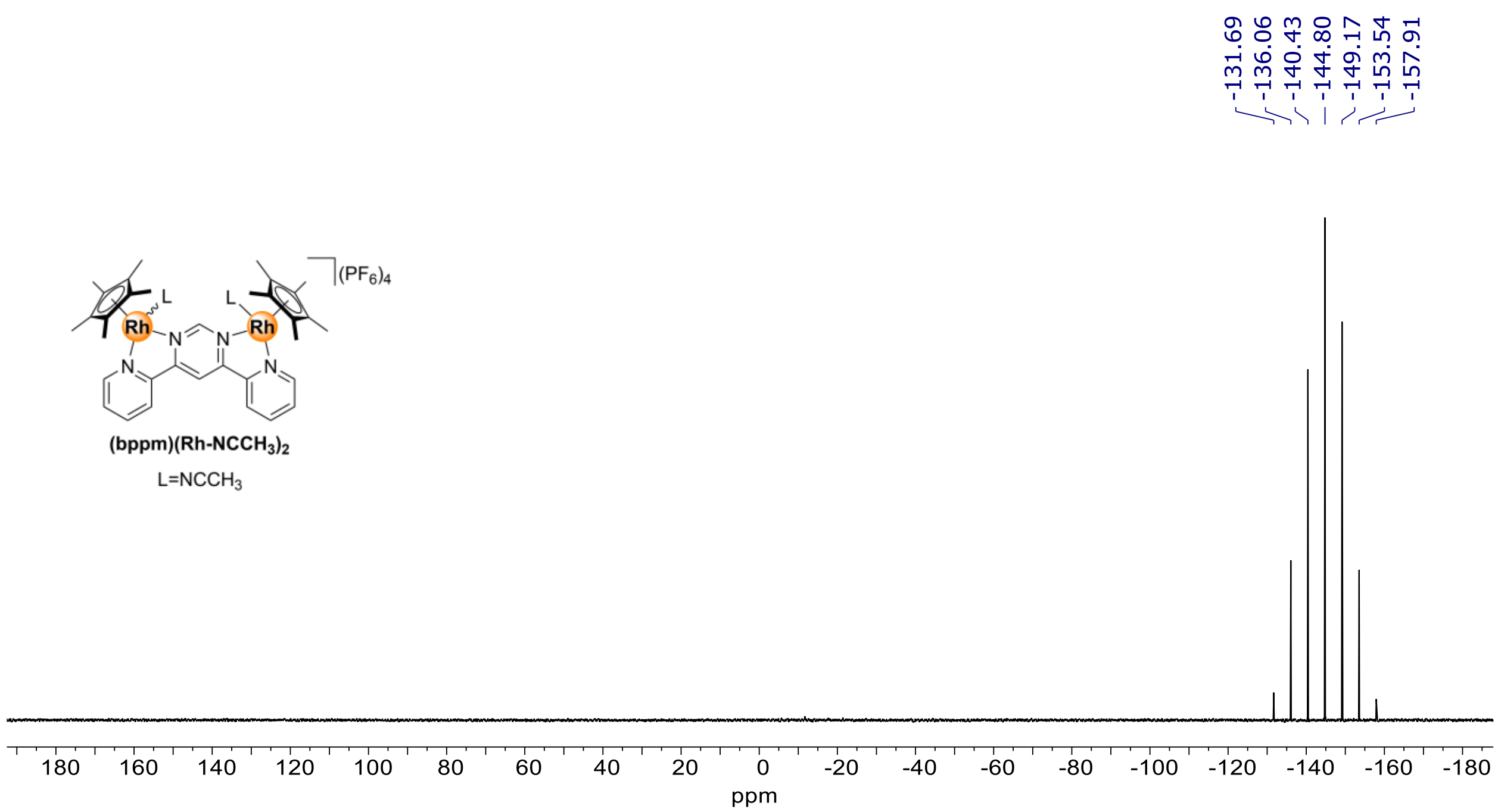

Figure S33. $^{31}P\{^1H\}$ NMR spectrum (162 MHz, CD_3NO_2) of **(bppm)(Rh-NCCH$_3$)$_2$**.

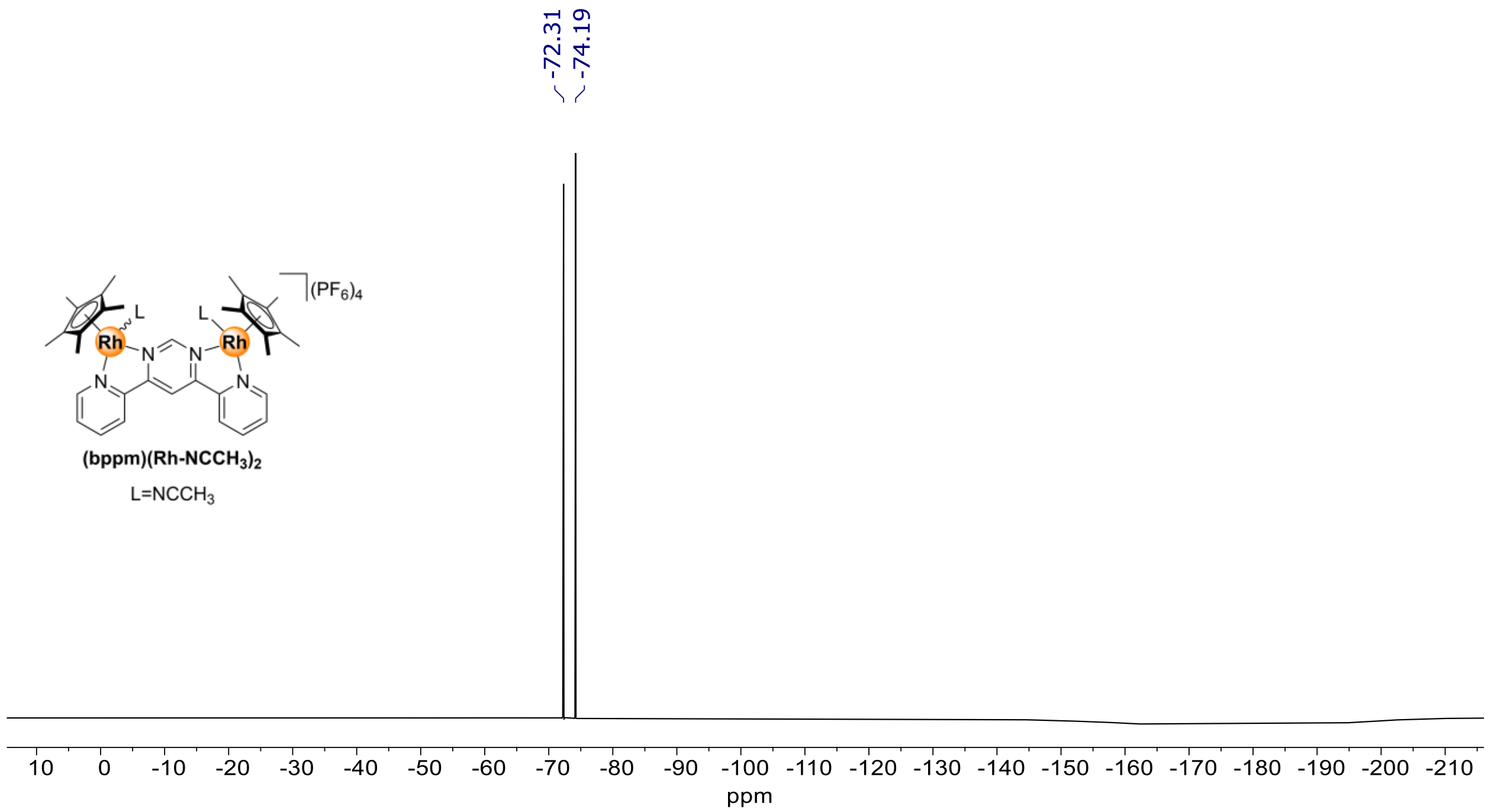

Figure S34. ^{19}F NMR spectrum (376 MHz, CD_3NO_3) of **(bppm)(Rh-NCCH$_3$)$_2$**.

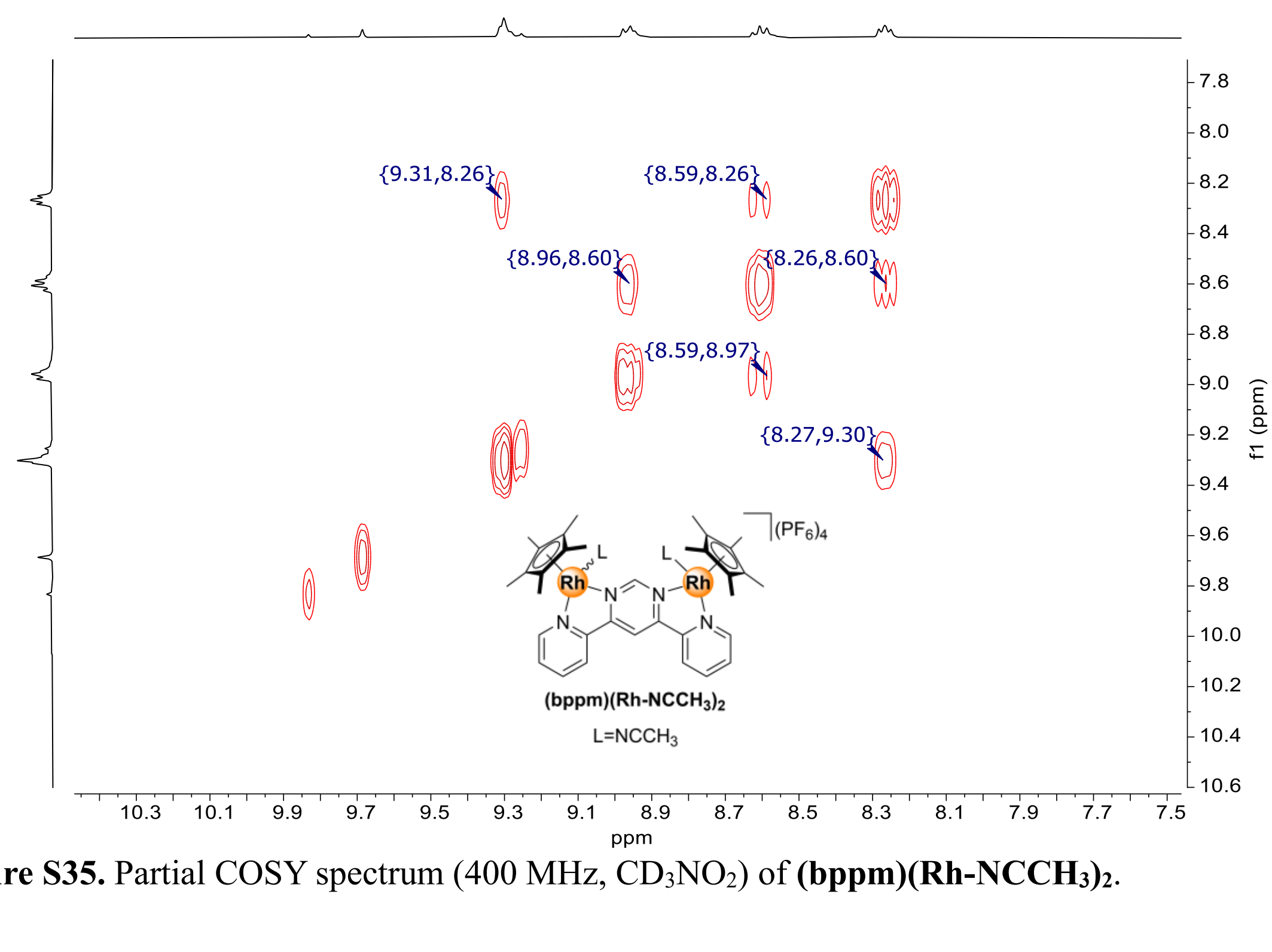

Figure S35. Partial COSY spectrum (400 MHz, CD_3NO_2) of **(bppm)(Rh-NCCH$_3$)$_2$**.

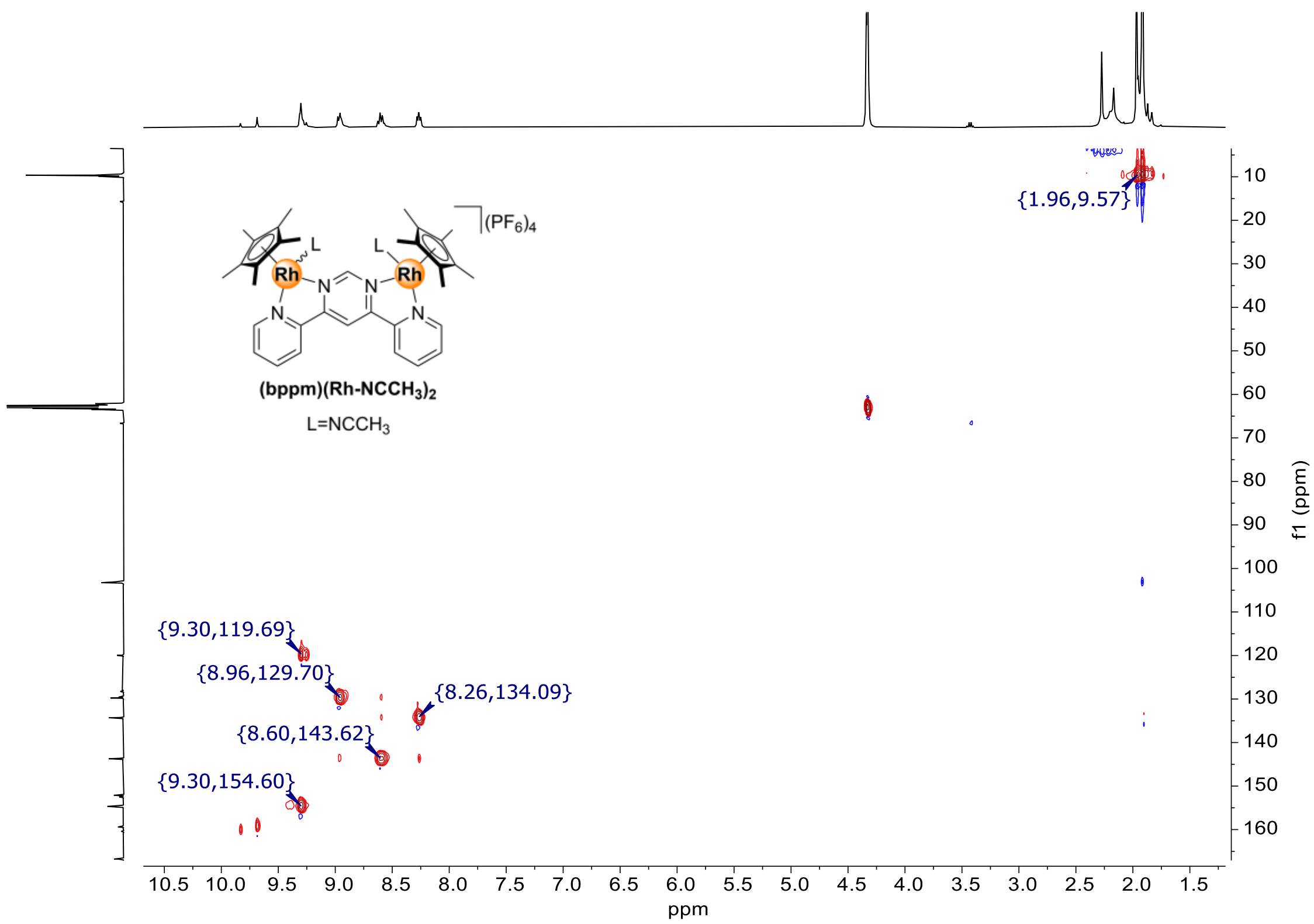

Figure S36. Partial ^{1}H-^{13}C HSQC spectrum (^{1}H: 400 MHz, ^{13}C: 101 MHz, CD_3NO_2) of **(bppm)(Rh-NCCH$_3$)$_2$**.

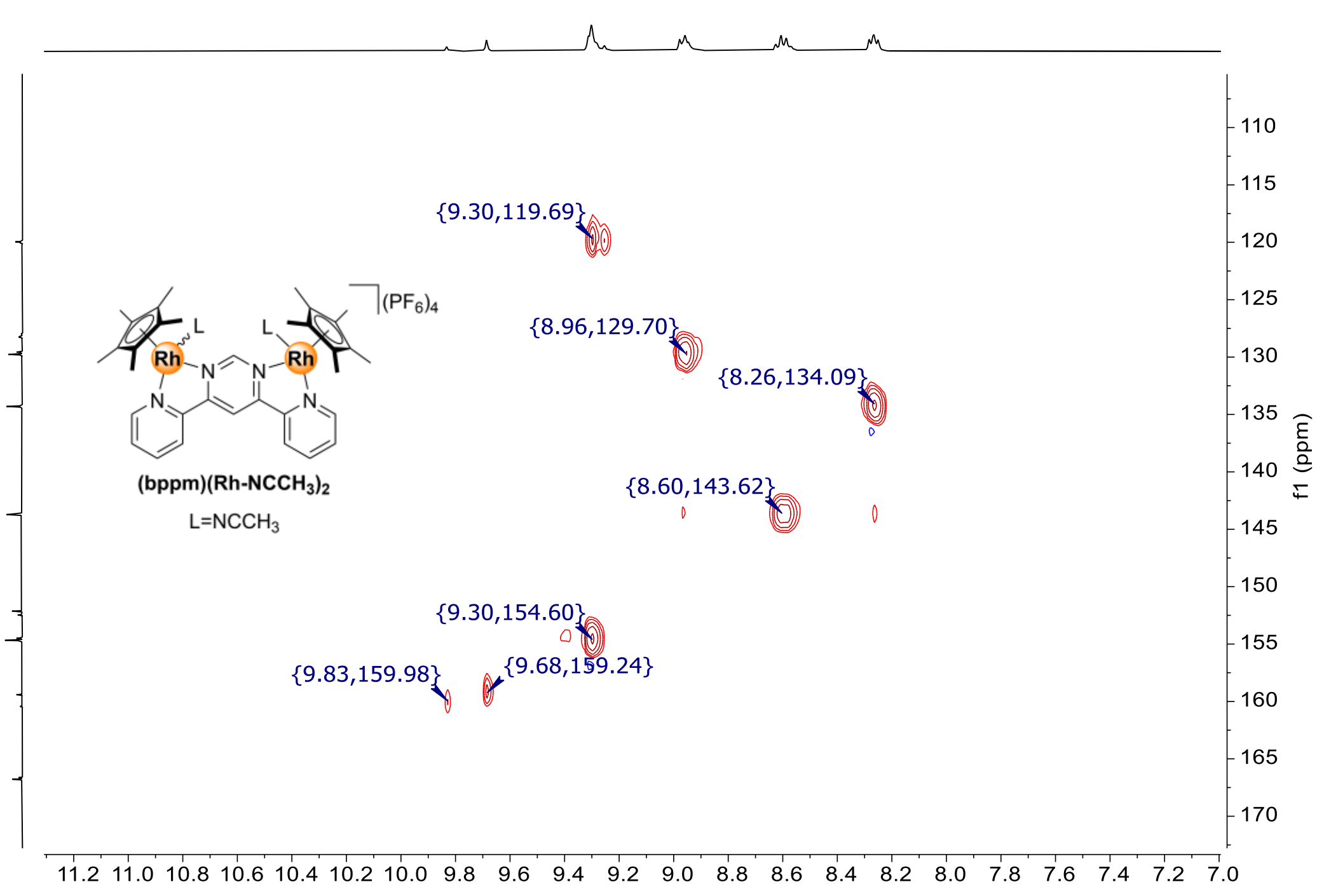

Figure S37. Partial ^{1}H-^{13}C HSQC spectrum (^{1}H: 400 MHz, ^{13}C: 101 MHz, CD_3NO_2) of **(bppm)(Rh-NCCH$_3$)$_2$**.

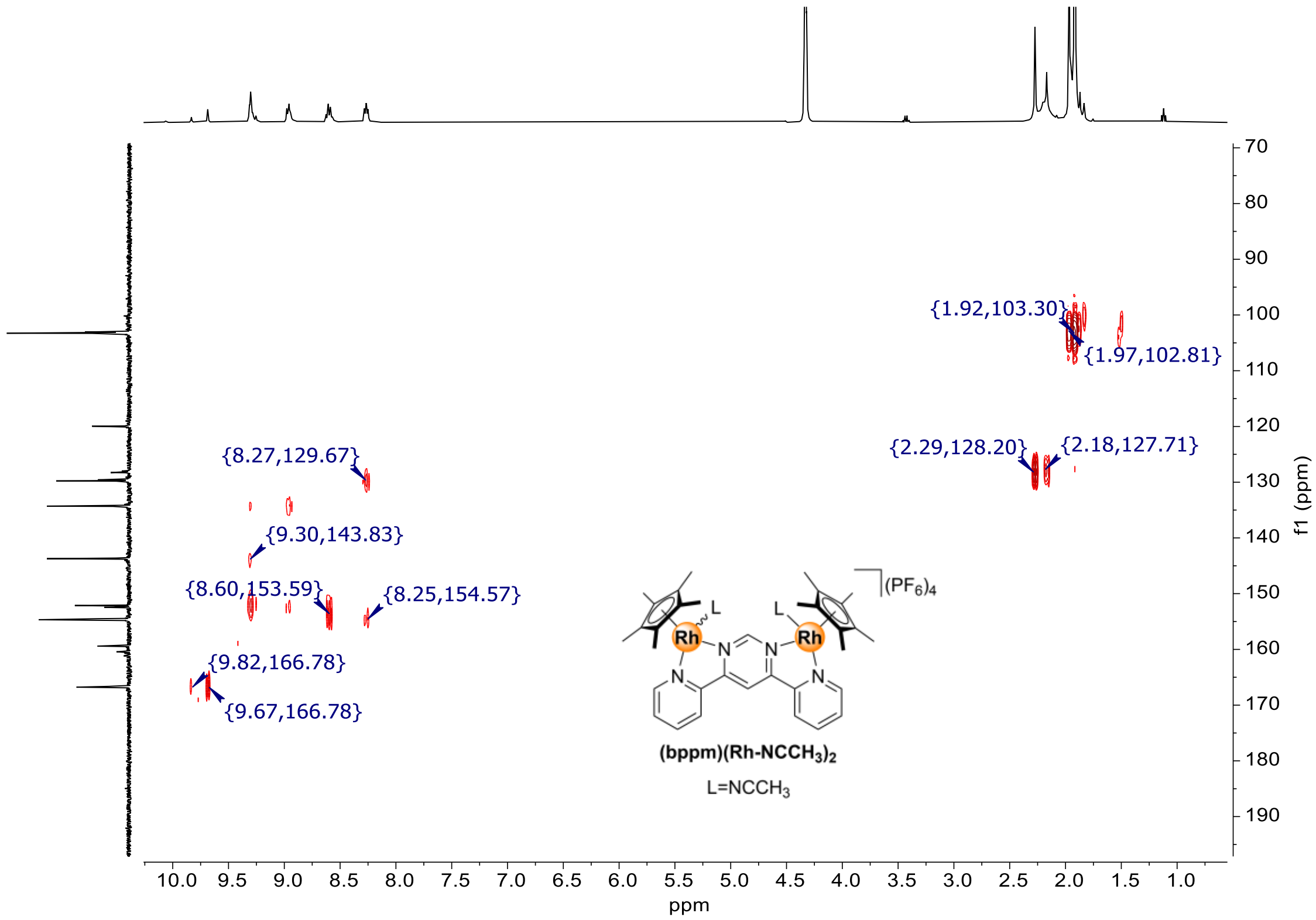

Figure S38. Partial ^{1}H-^{13}C HMBC spectrum (^{1}H: 400 MHz, ^{13}C: 101 MHz, CD_3NO_2) of **(bppm)(Rh-NCCH$_3$)$_2$**.

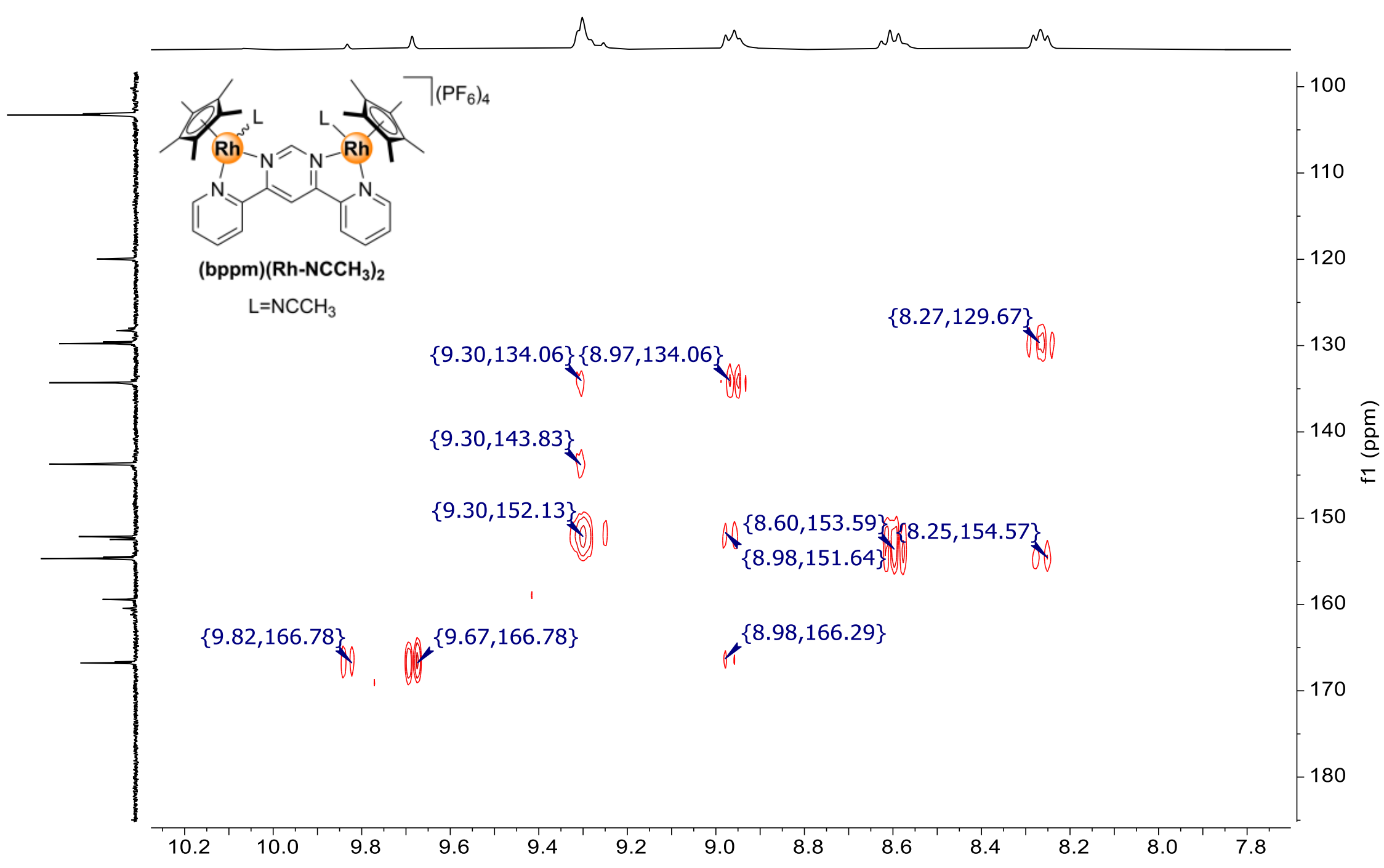

Figure S39. Partial ^{1}H-^{13}C HMBC spectrum (^{1}H: 400 MHz, ^{13}C: 101 MHz, CD_3NO_2) of **(bppm)(Rh-NCCH$_3$)$_2$**.

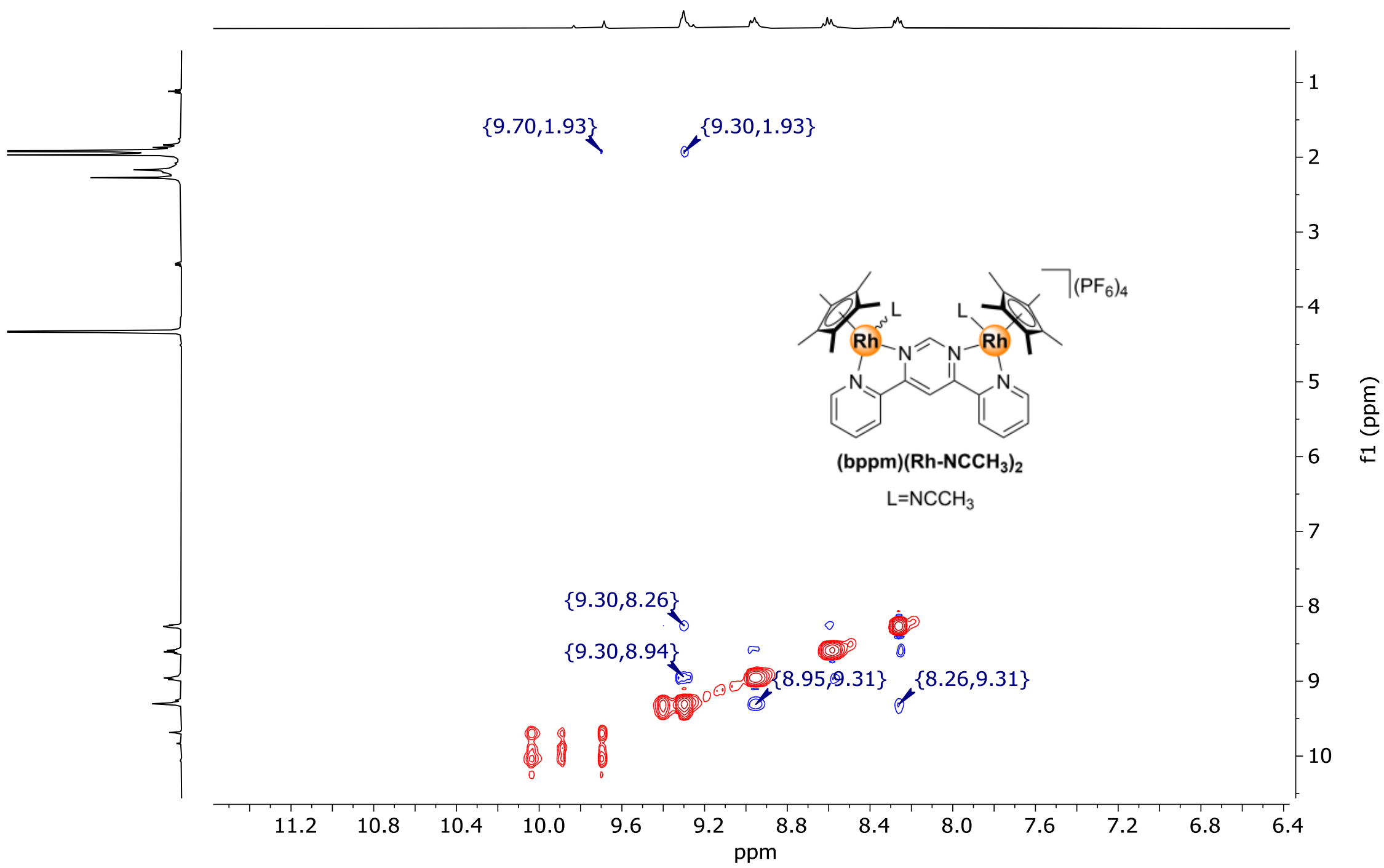

Figure S40. Partial NOESY spectrum (400 MHz, CD_3NO_2) of **(bppm)(Rh-NCCH$_3$)$_2$**.

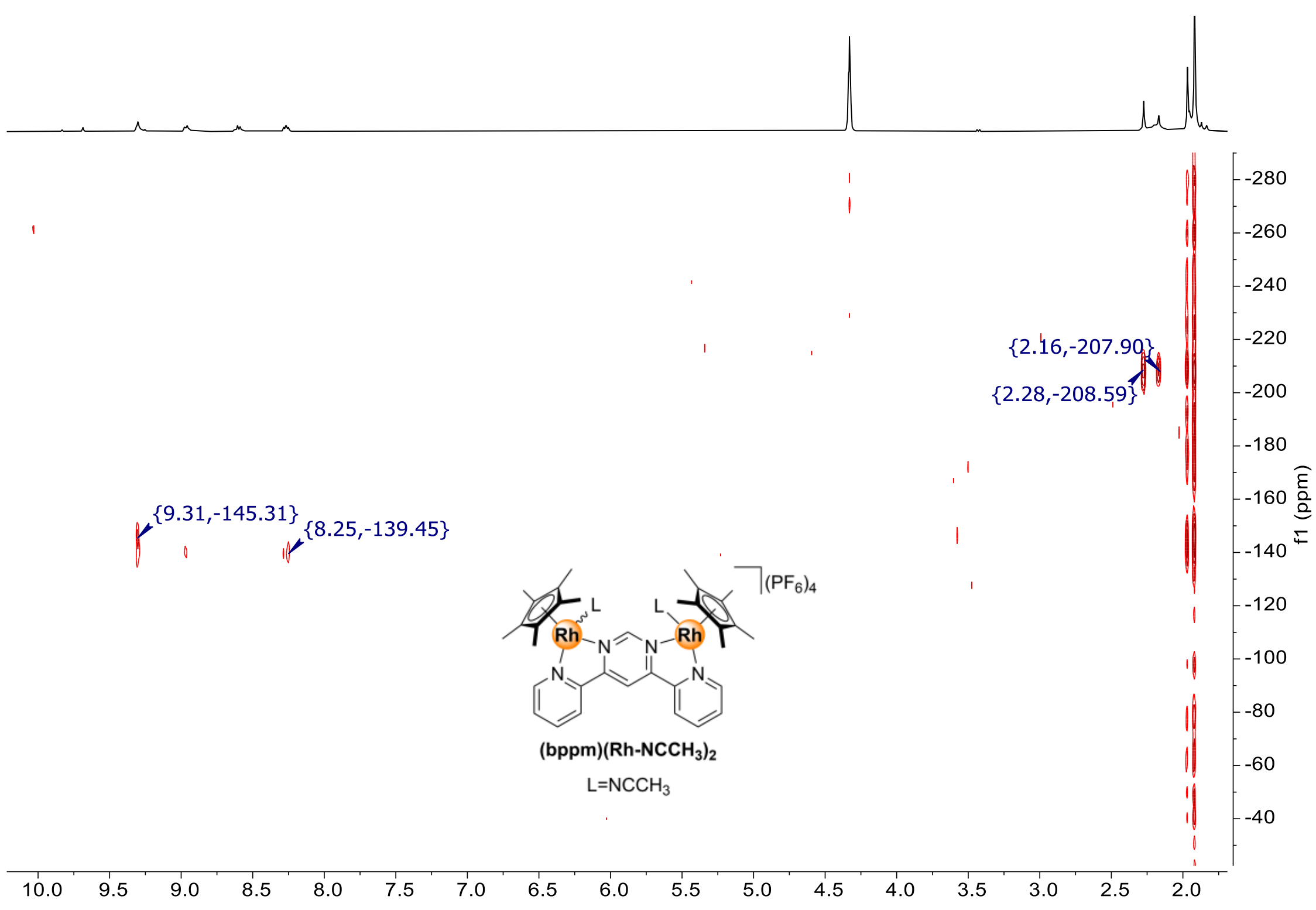

Figure S41. Partial ^1H-^{15}N HMBC spectrum (^1H: 400 MHz, ^{15}N: 41 MHz, CD_3NO_2) of **(bppm)(Rh-NCCH$_3$)$_2$**.

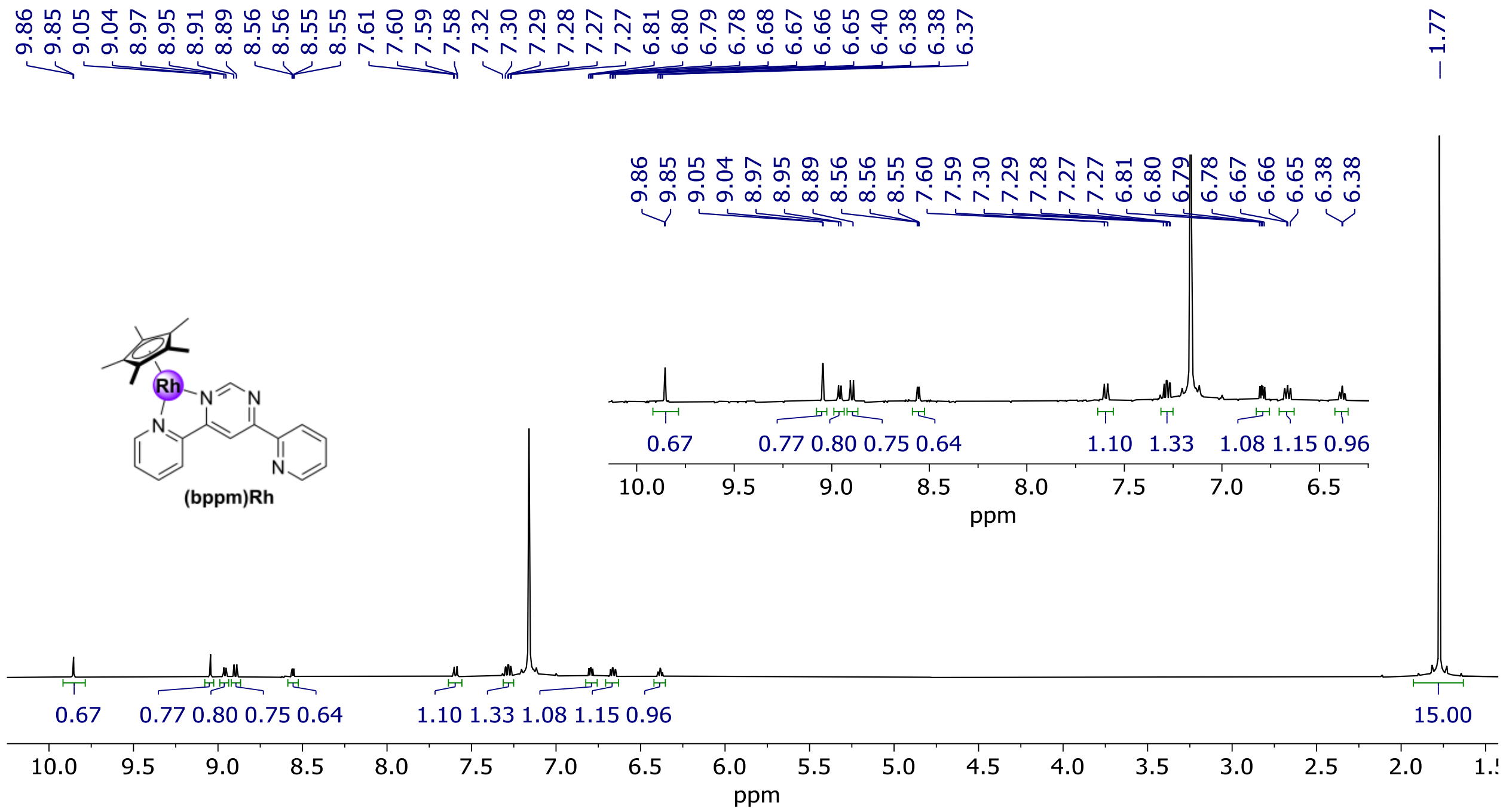

Figure S42. ^{1}H NMR (500 MHz, CD_3CN) of **(bppm)Rh**. Inset: enlarged view of aromatic region.

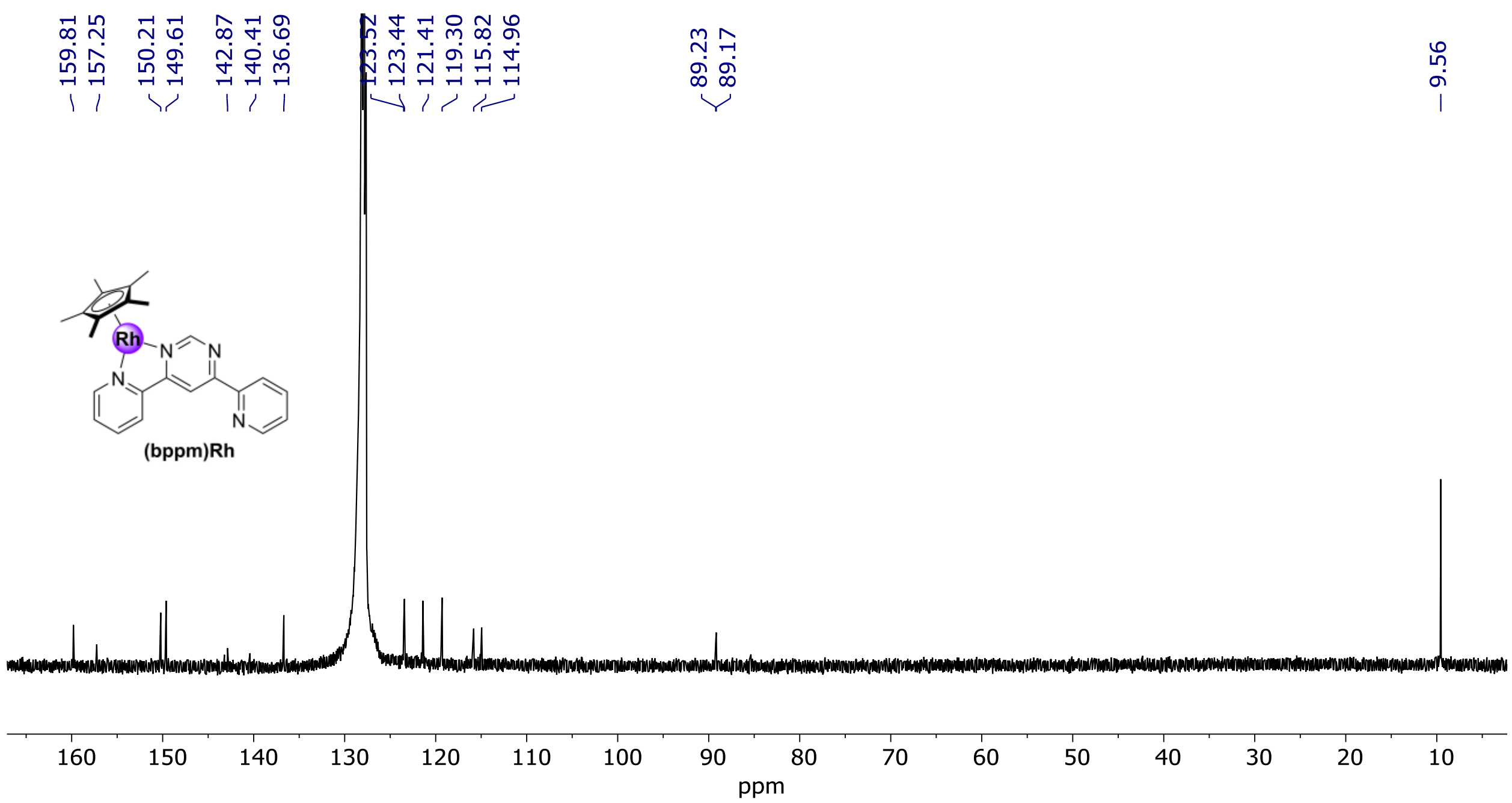

Figure S43. $^{13}C\{^{1}H\}$ NMR spectrum (125 MHz, CD_3CN) of **(bppm)Rh**

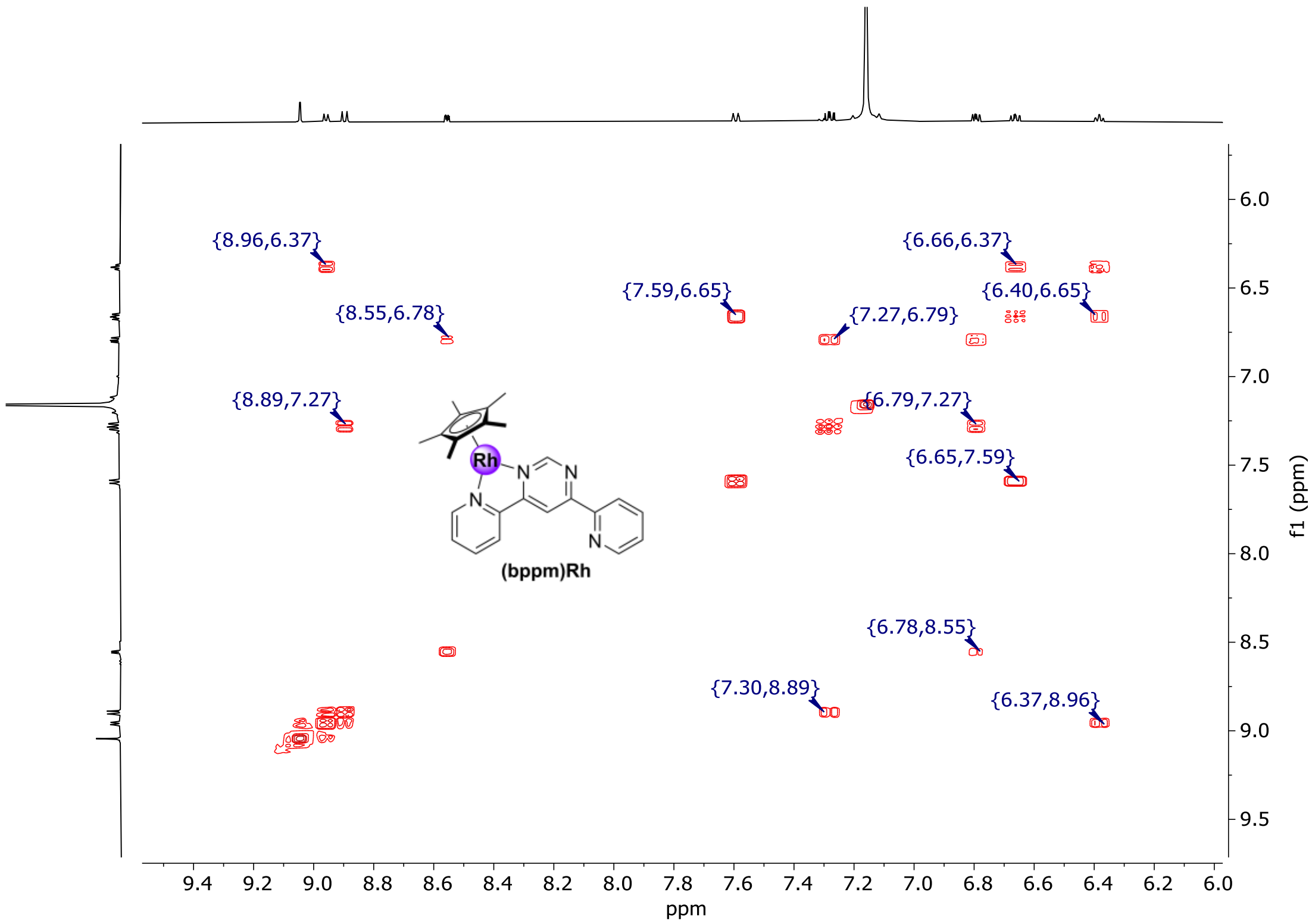

Figure S44. Partial COSY spectrum (500 MHz, CD_3CN) of **(bppm)Rh**.

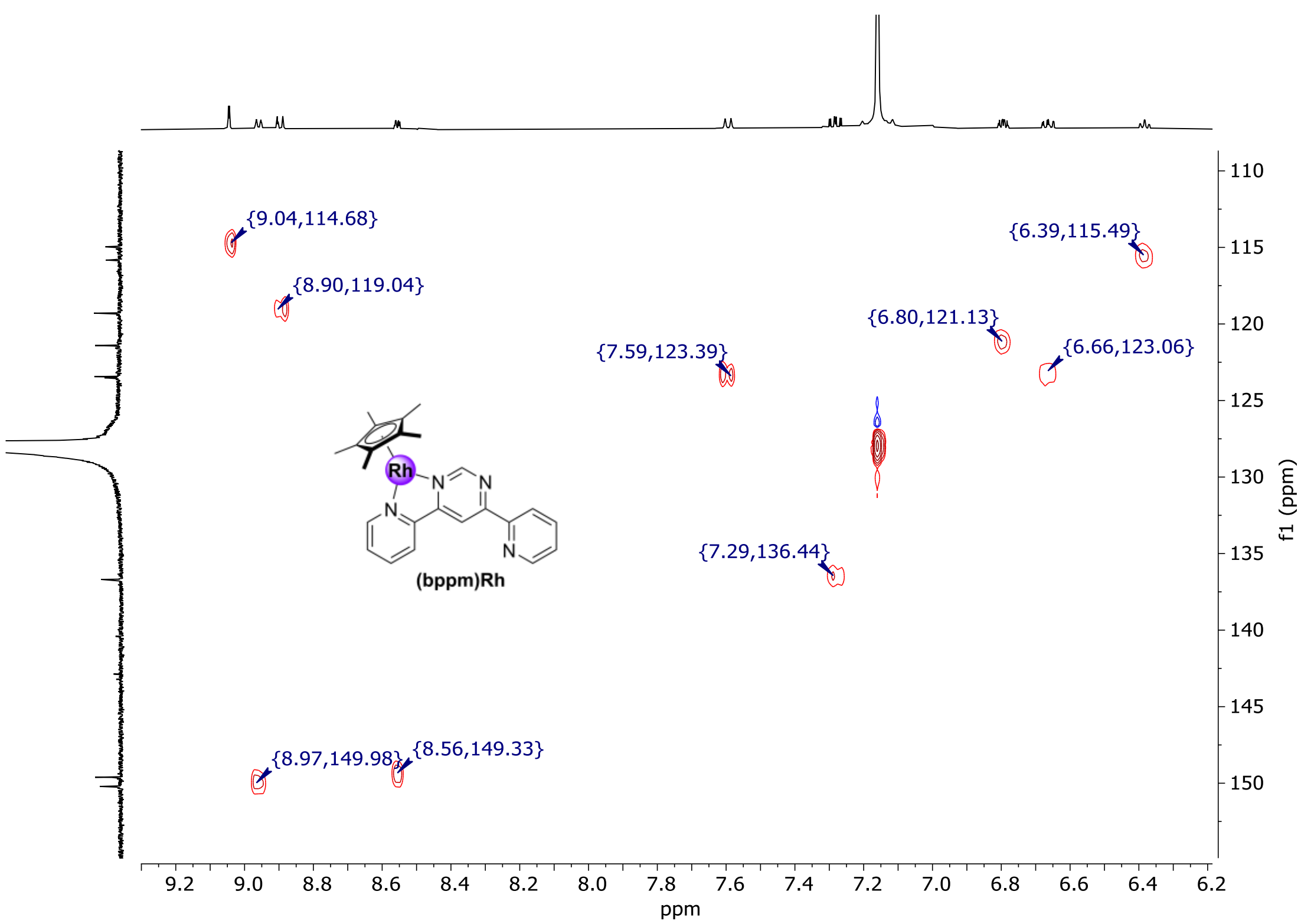

Figure S45. Partial ^{1}H-^{13}C HSQC spectrum (^{1}H: 500 MHz, ^{13}C: 125 MHz, CD_3CN) of **(bppm)Rh**.

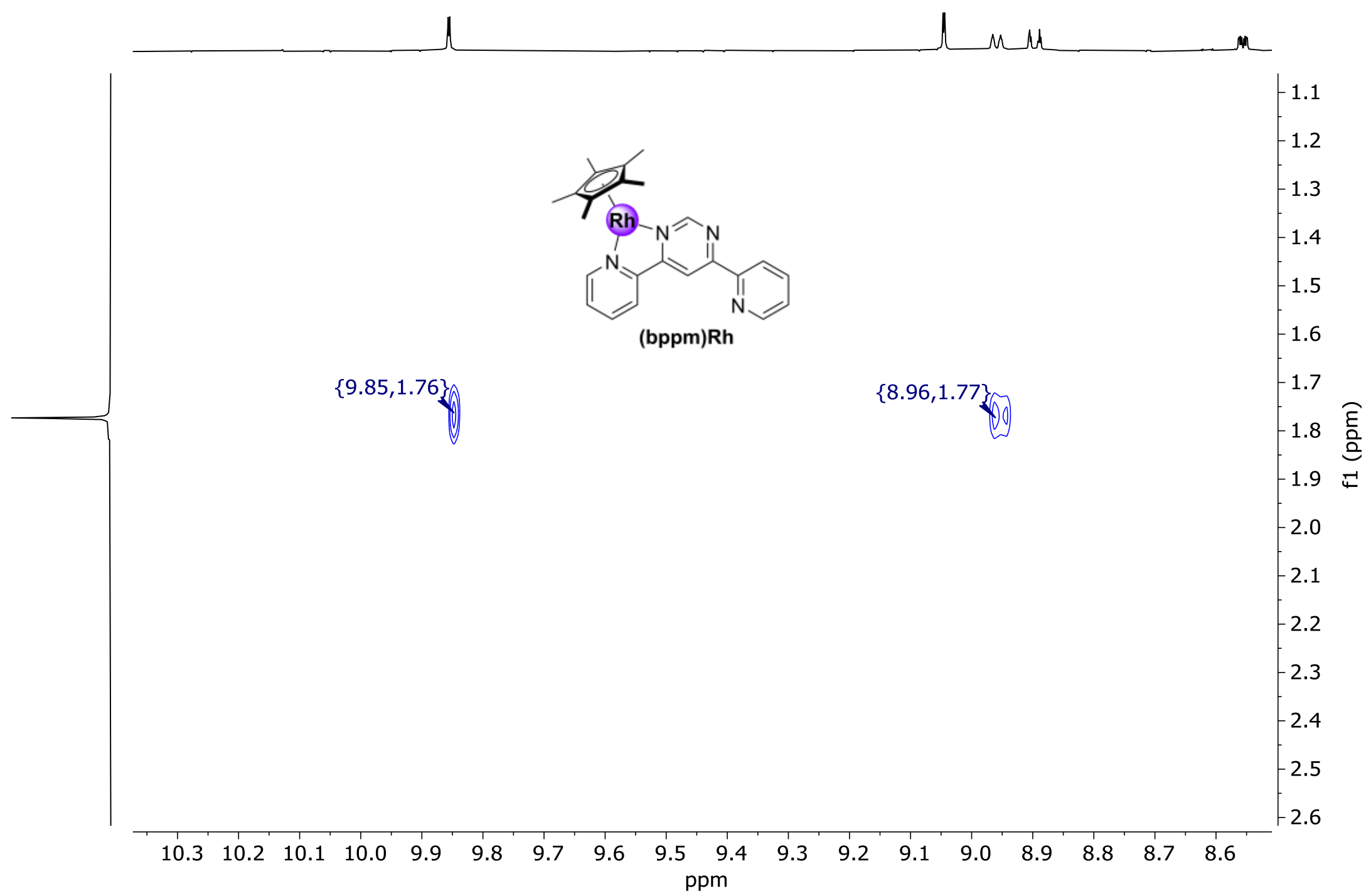

Figure S46. Partial NOESY spectrum (500MHz, CD_3CN) of **(bppm)Rh**.

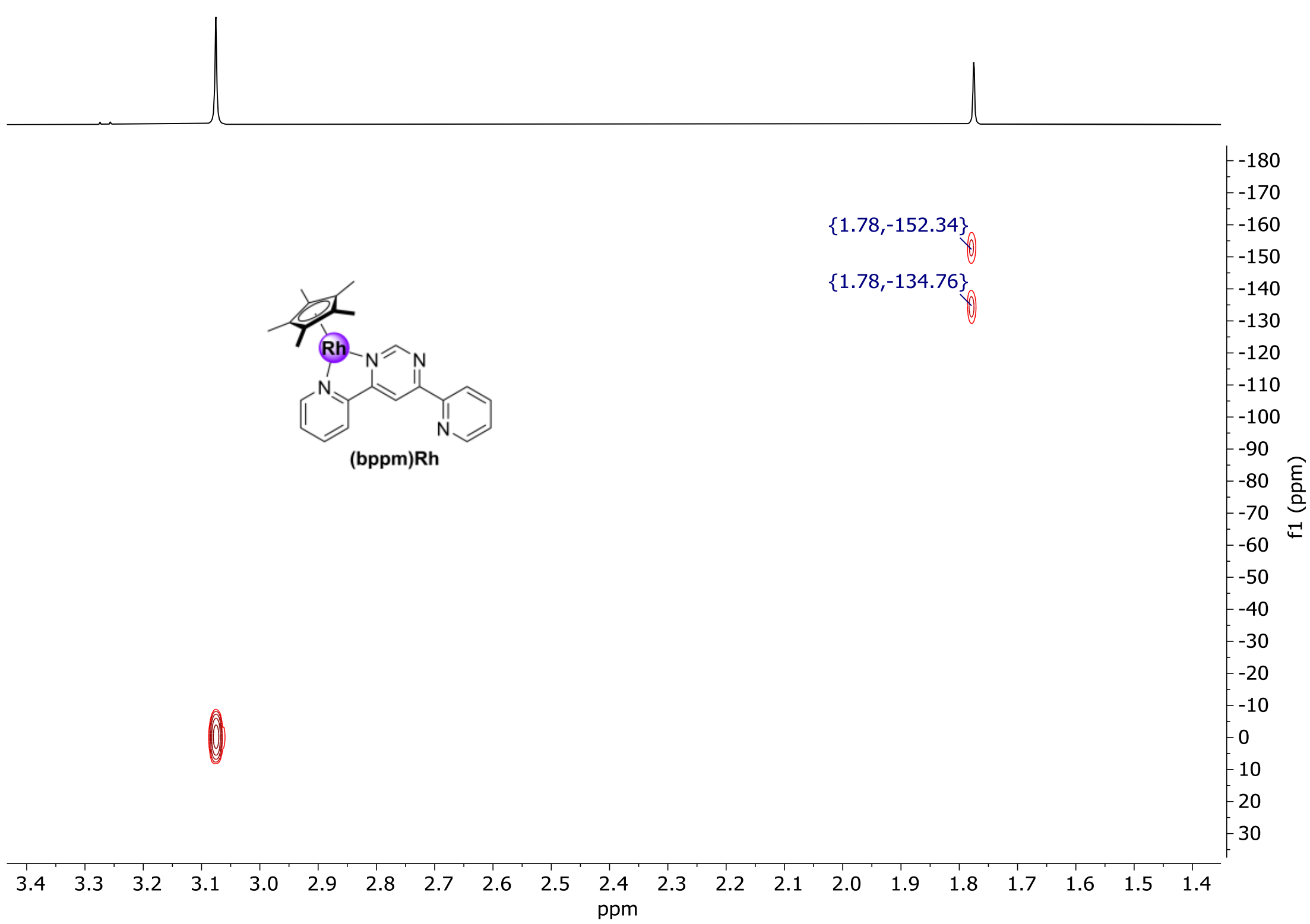

Figure S47. Partial ^1H-^{15}N HMBC spectrum (^1H: 400 MHz, ^{15}N: 41 MHz; CD_3CN) for **(bppm)Rh**.

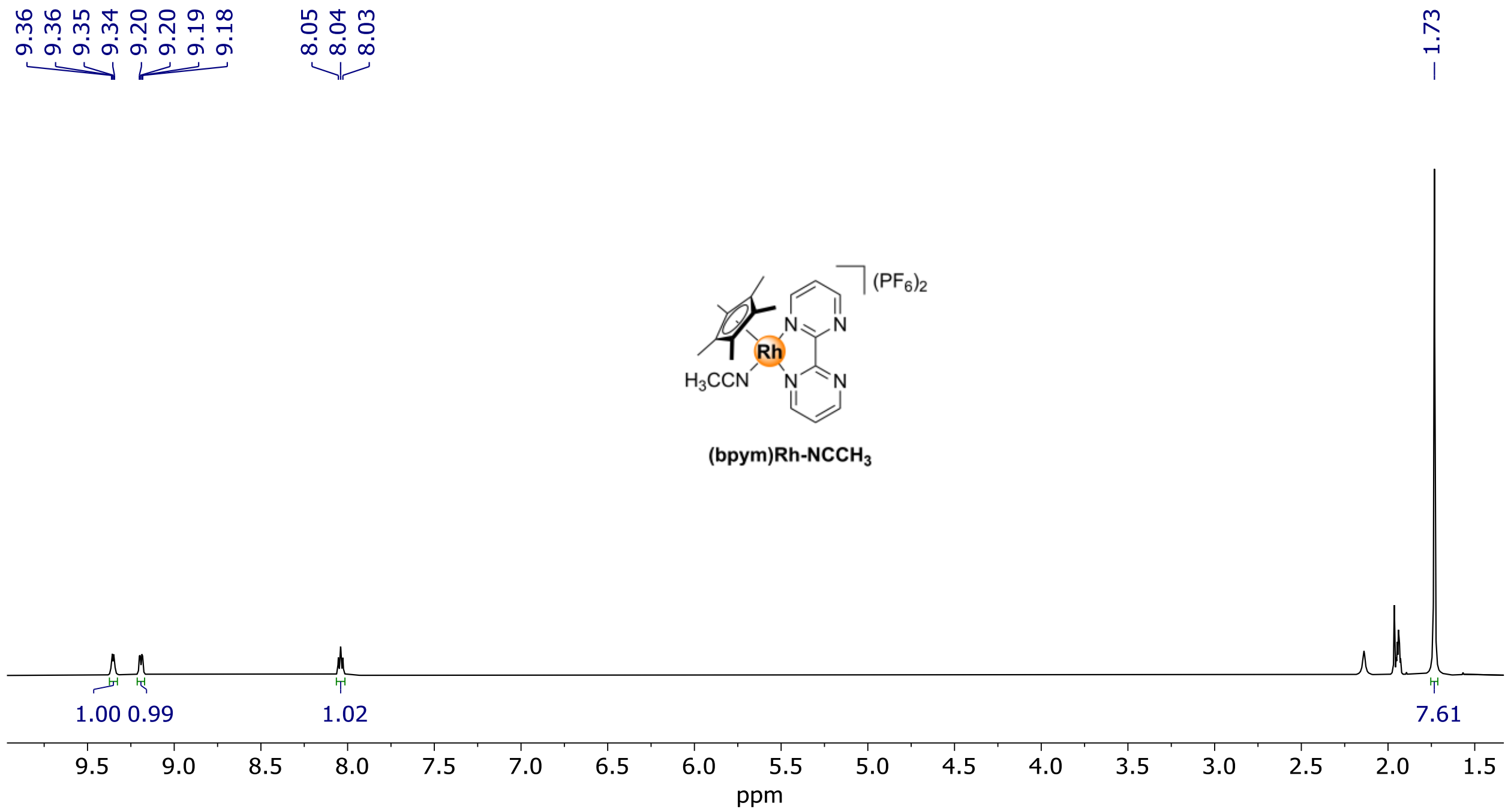

Figure S48. ^{1}H NMR (500 MHz, CD_3CN) of **(bpym)Rh-NCCH₃**.

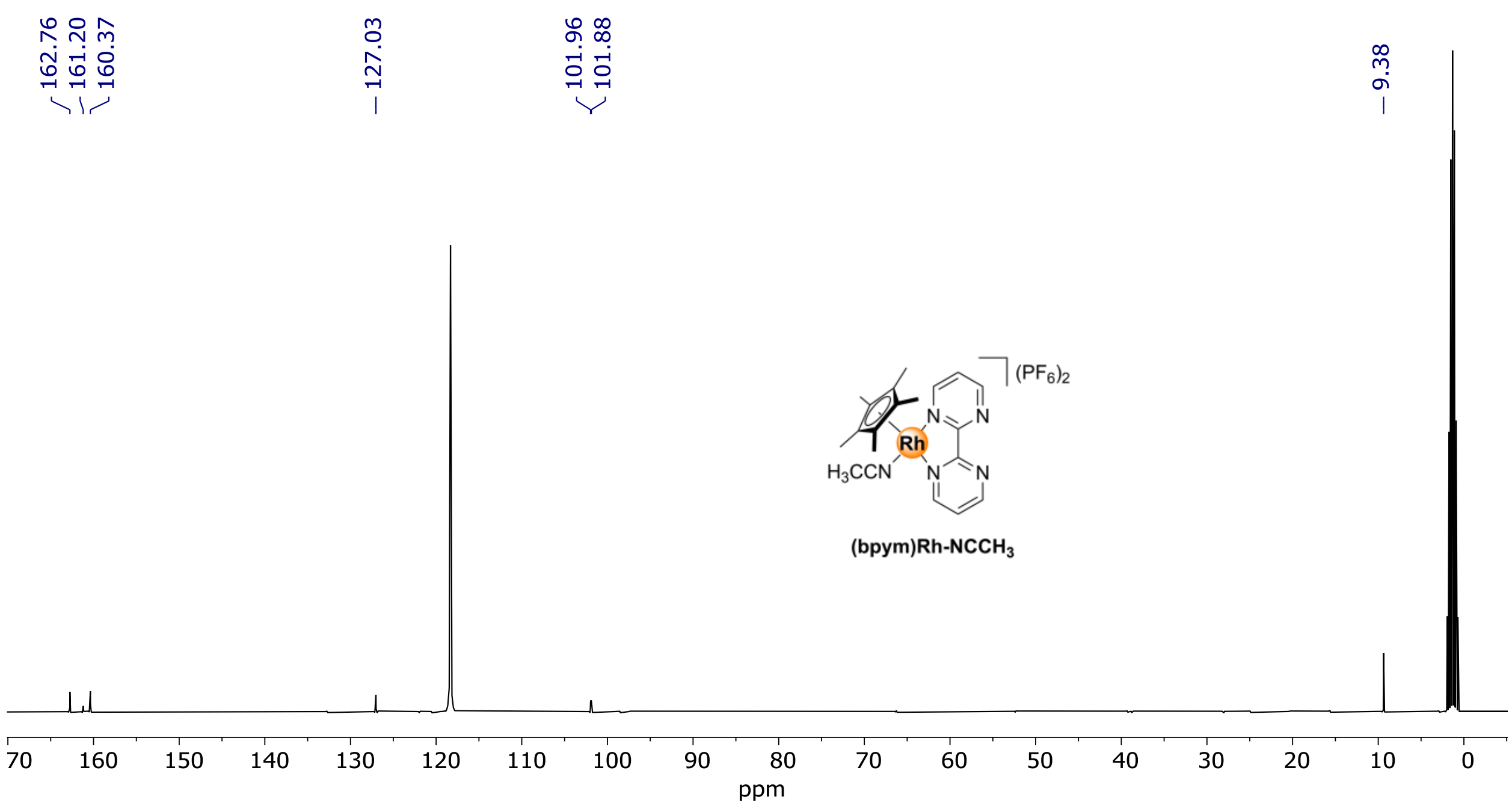

Figure S49. $^{13}C\{^{1}H\}$ NMR spectrum (125 MHz, CD_3CN) of **(bpym)Rh-NCCH$_3$**.

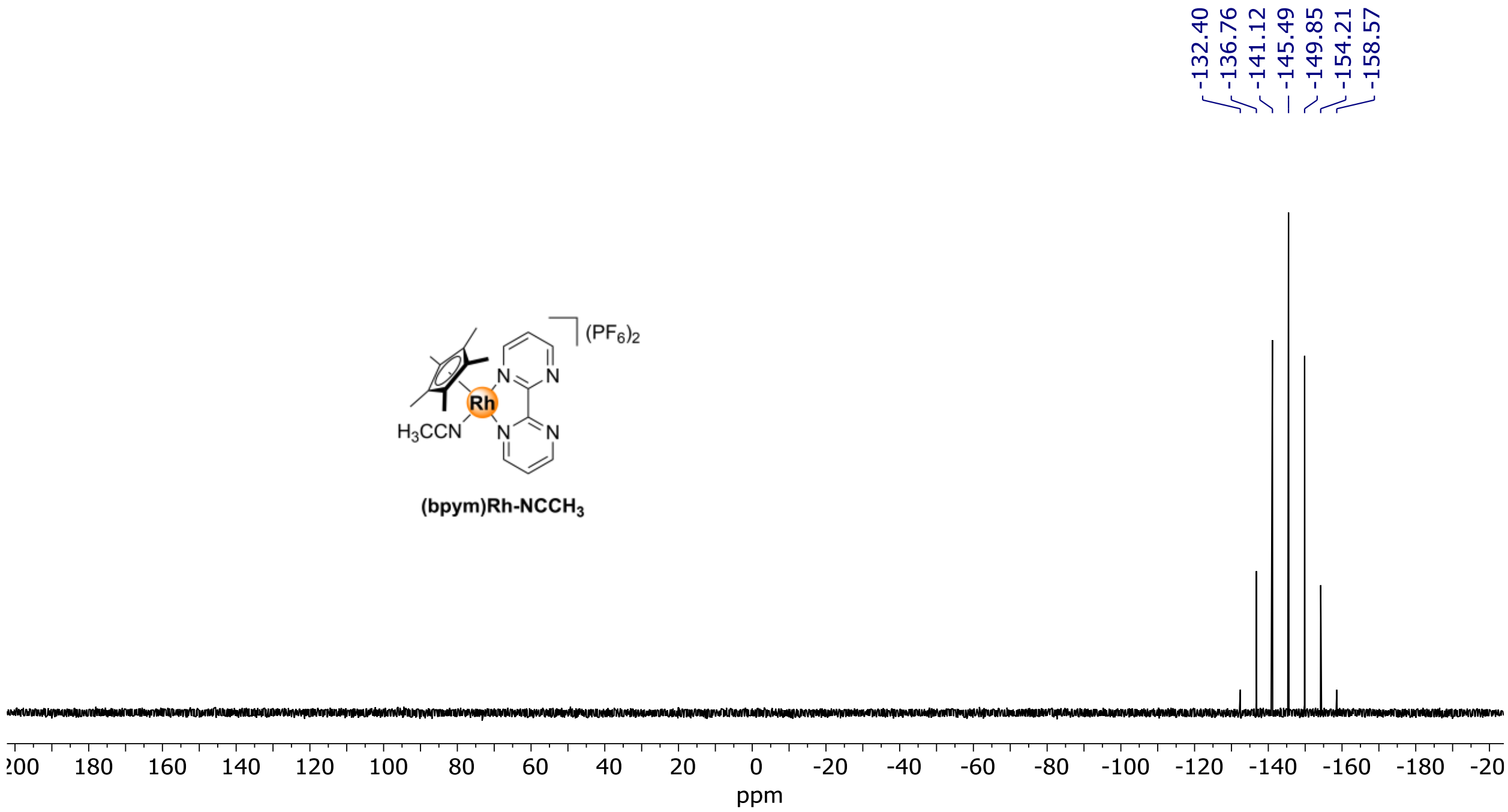

Figure S50. $^{31}P\{^{1}H\}$ NMR spectrum (162 MHz, CD_3CN) of **(bpym)Rh-NCCH$_3$**.

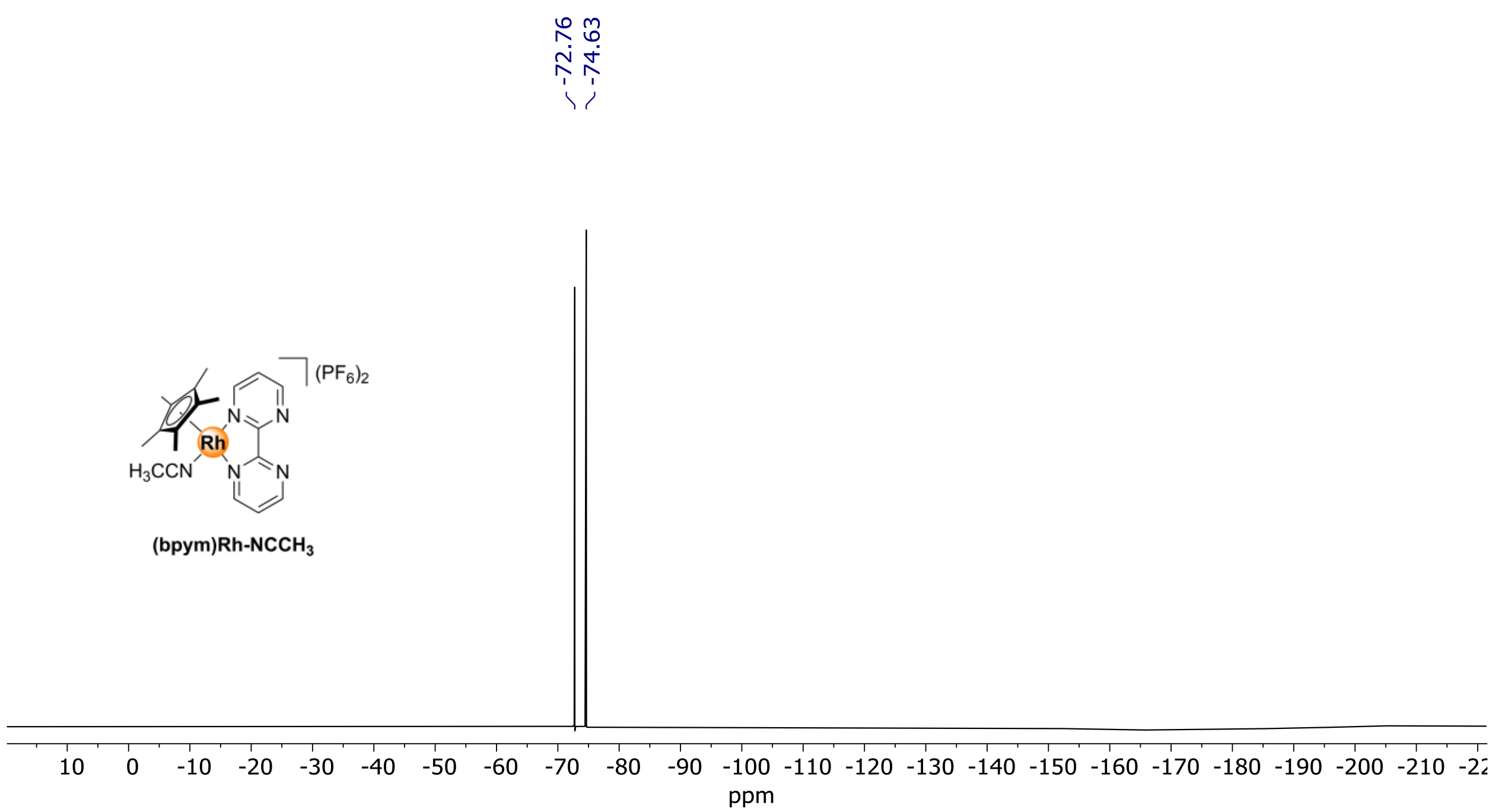

Figure S51. ^{19}F NMR spectrum (376 MHz, CD_3CN) of **(bpym)Rh-NCCH$_3$**.

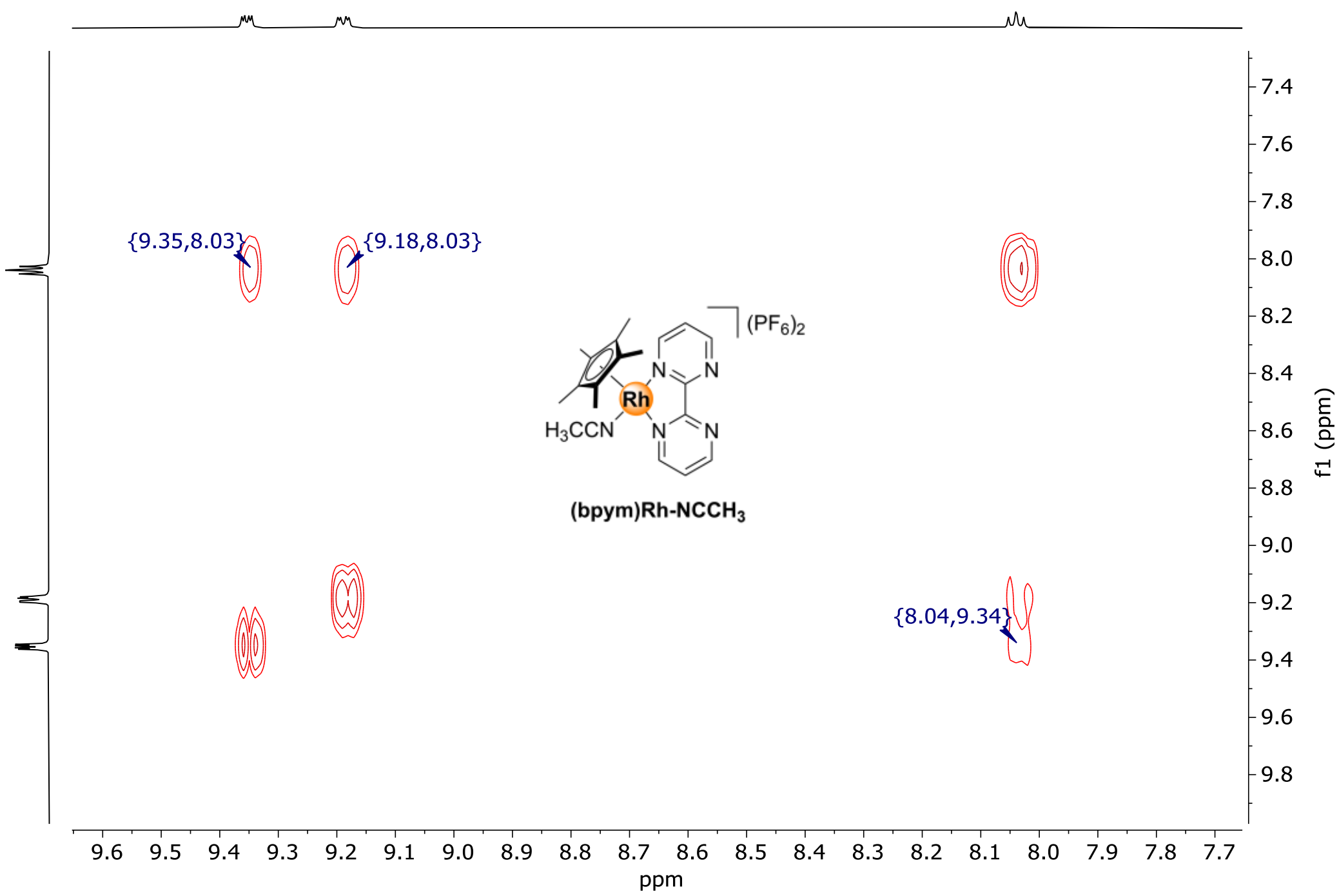

Figure S52. Partial COSY spectrum (400 MHz, CD_3CN) of **(bpym)Rh-NCCH$_3$**.

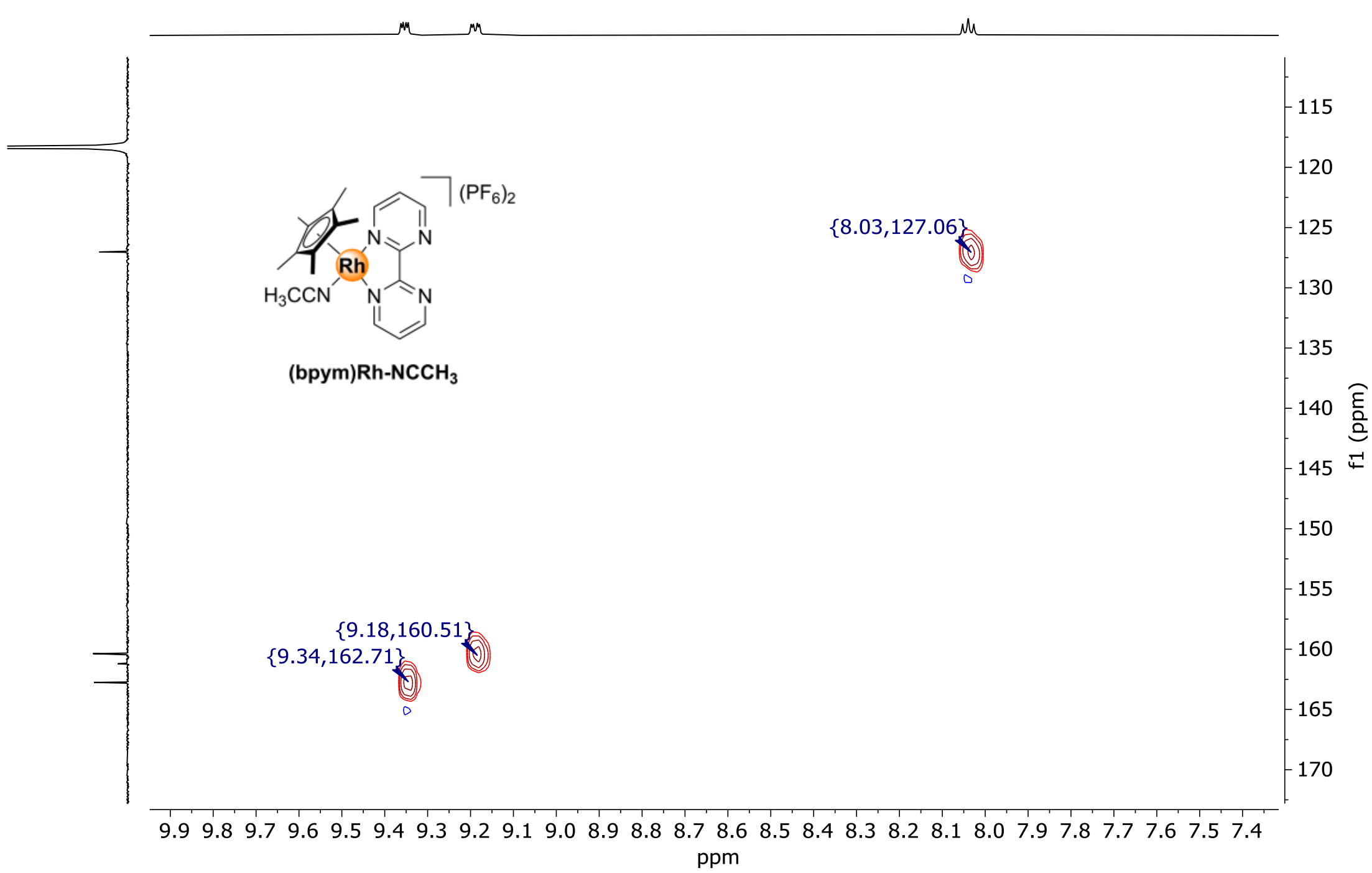

Figure S53. Partial ^1H-^{13}C HSQC spectrum (^1H: 400 MHz, ^{13}C: 101 MHz, CD_3CN) of **(bpym)Rh-NCCH$_3$**.

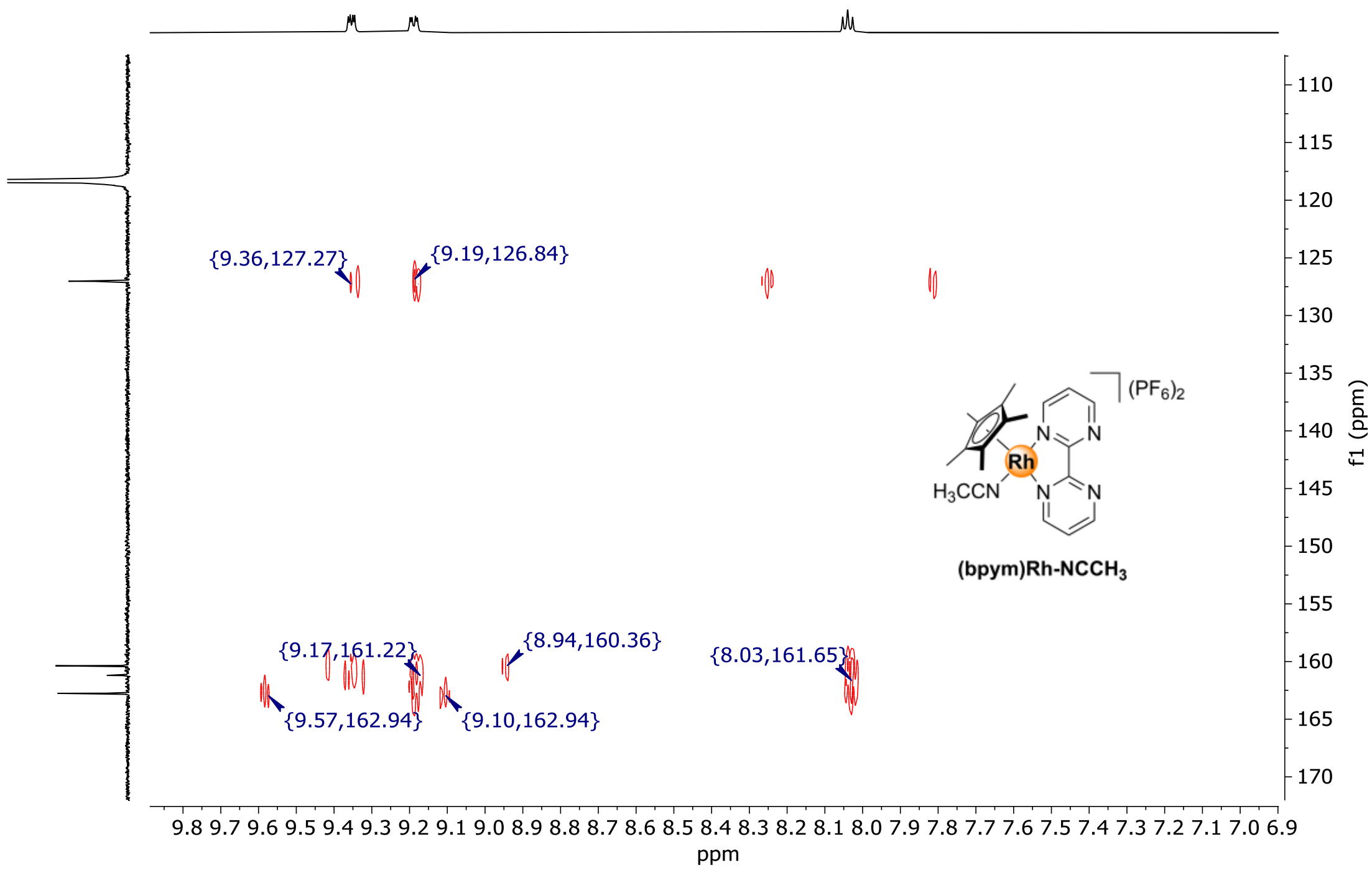

Figure S54. Partial ^{1}H-^{13}C HMBC spectrum (^{1}H: 400 MHz, ^{13}C: 101 MHz, CD_3CN) of **(bpym)Rh-NCCH$_3$**.

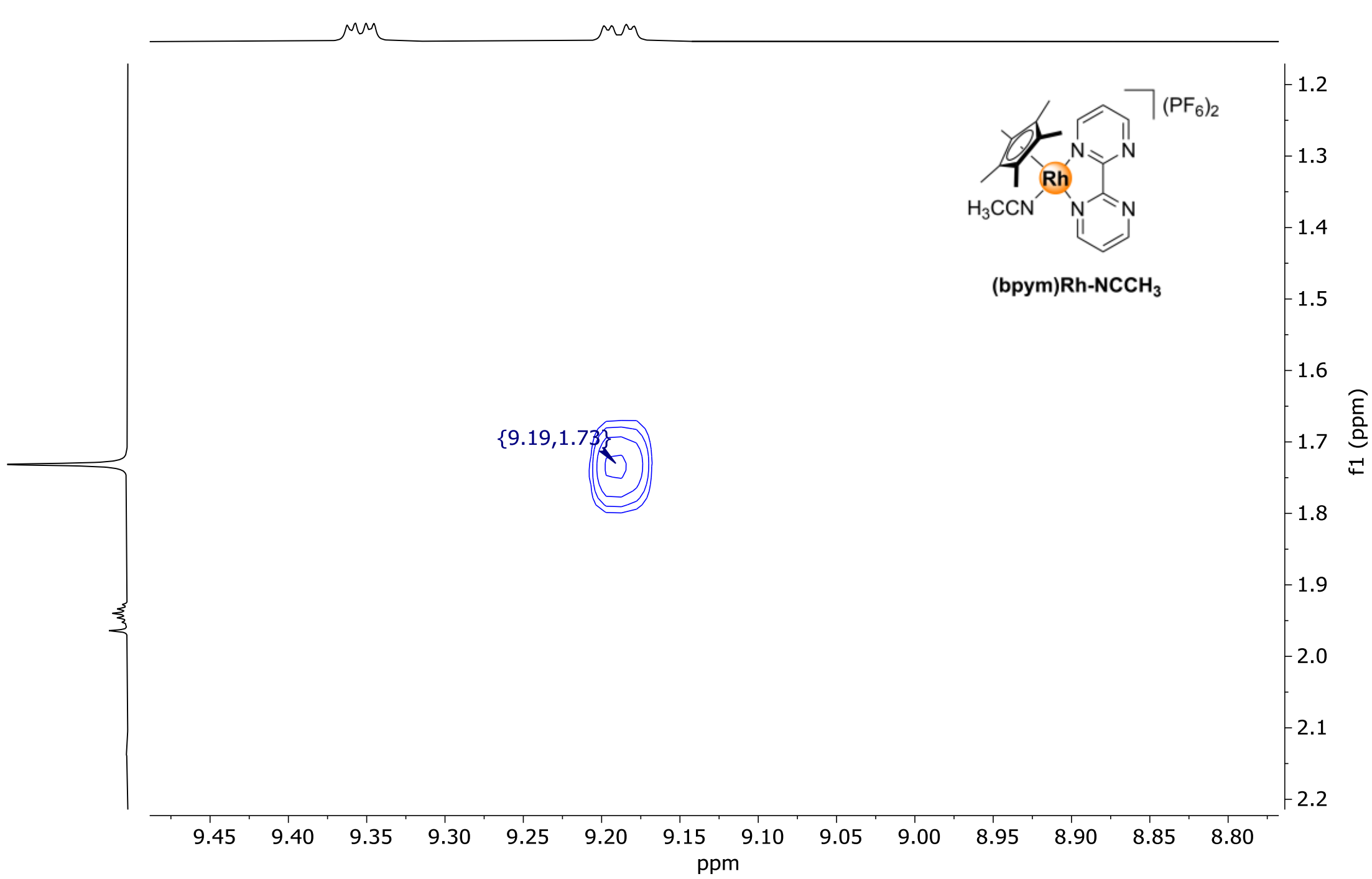

Figure S55. Partial NOESY spectrum (400MHz, CD_3CN) of **(bpym)Rh-NCCH$_3$**.

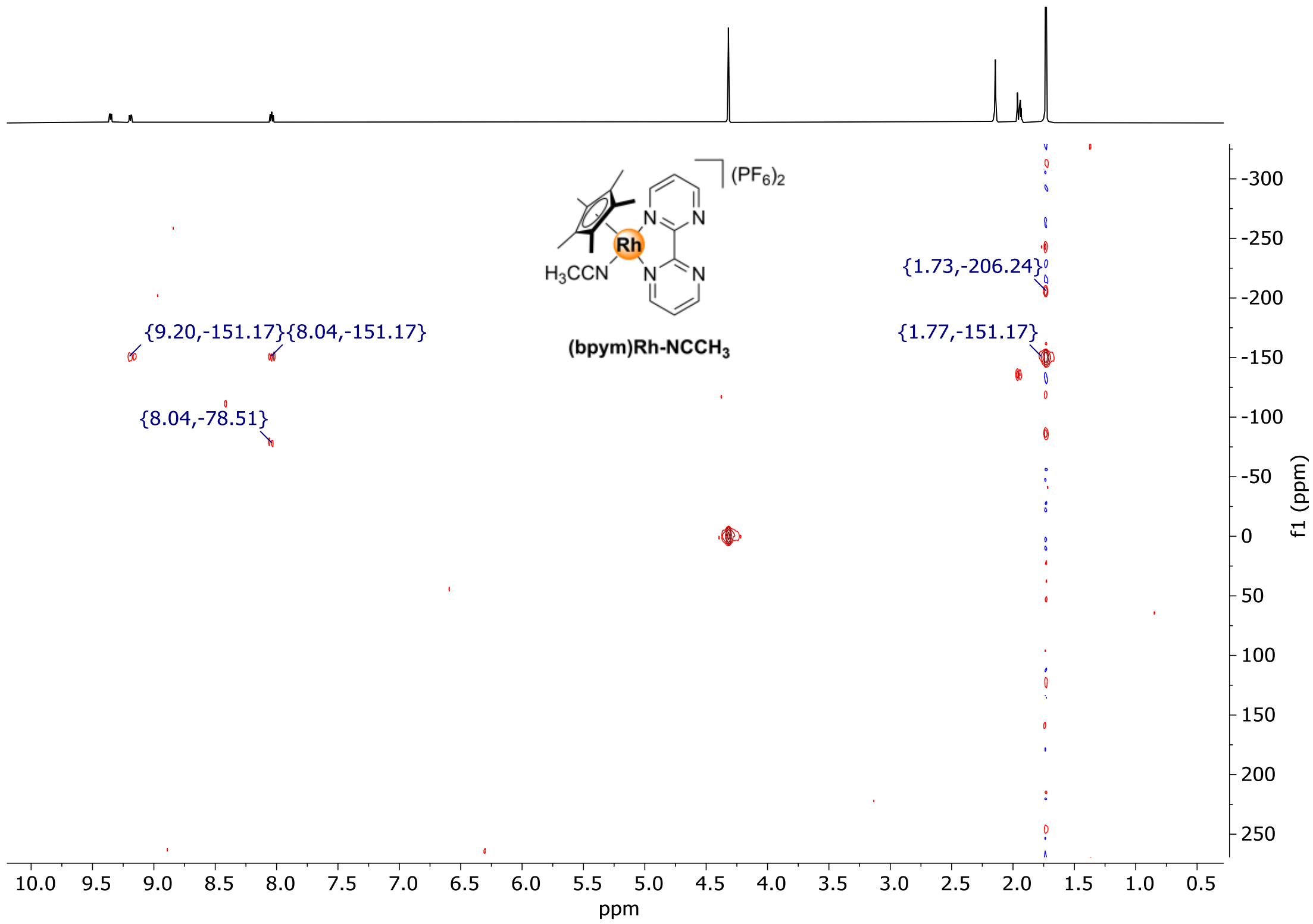

Figure S56. ^{1}H-^{15}N HMBC spectrum (^{1}H: 400 MHz, ^{15}N: 41 MHz; CD_3CN) for **(bpym)Rh-NCCH$_3$**.

^{15}N NMR data for literature complexes

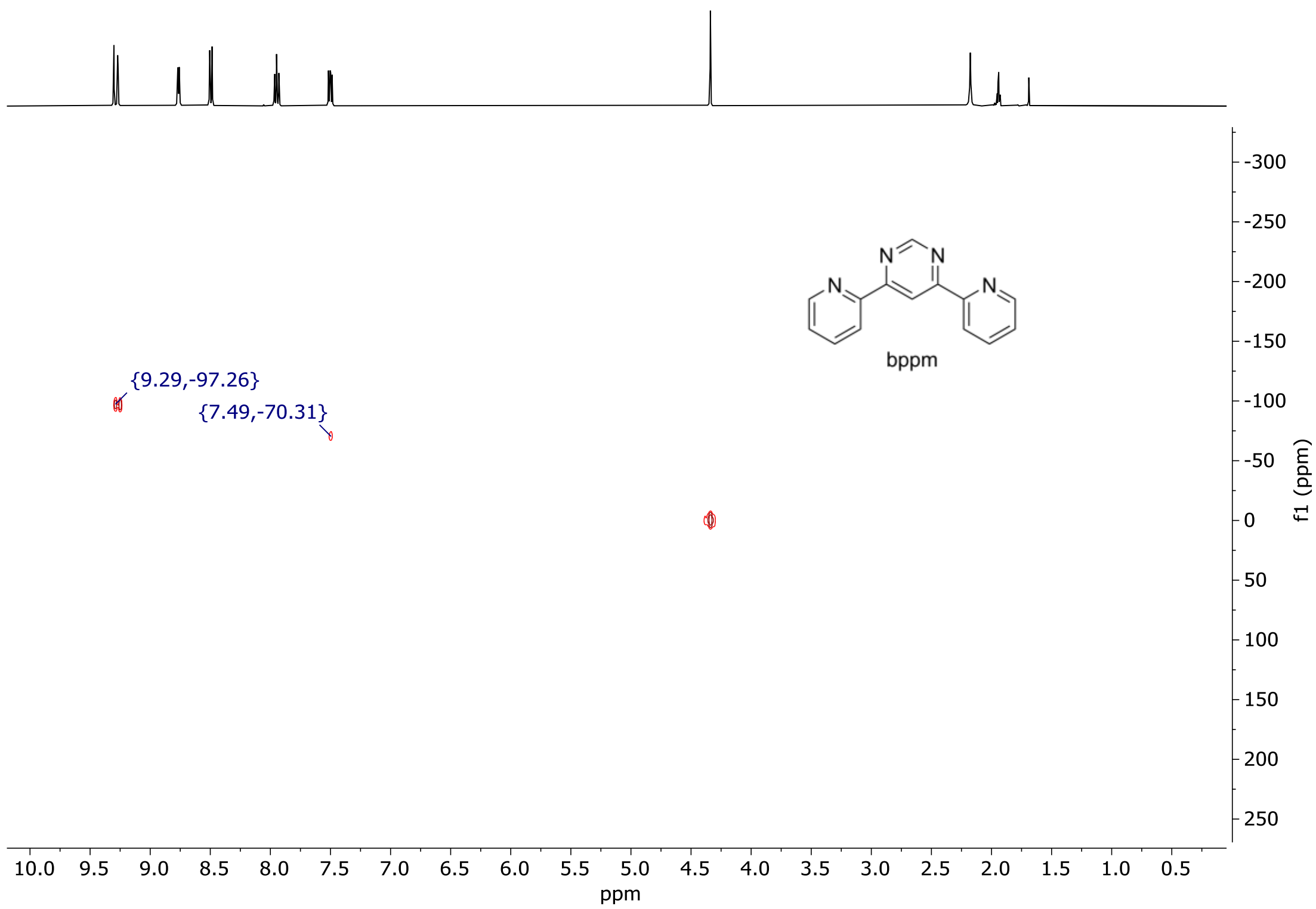

Figure S57. ^{1}H-^{15}N HMBC spectrum (^{1}H: 400 MHz, ^{15}N: 41 MHz; CD_3CN) for **bppm**.

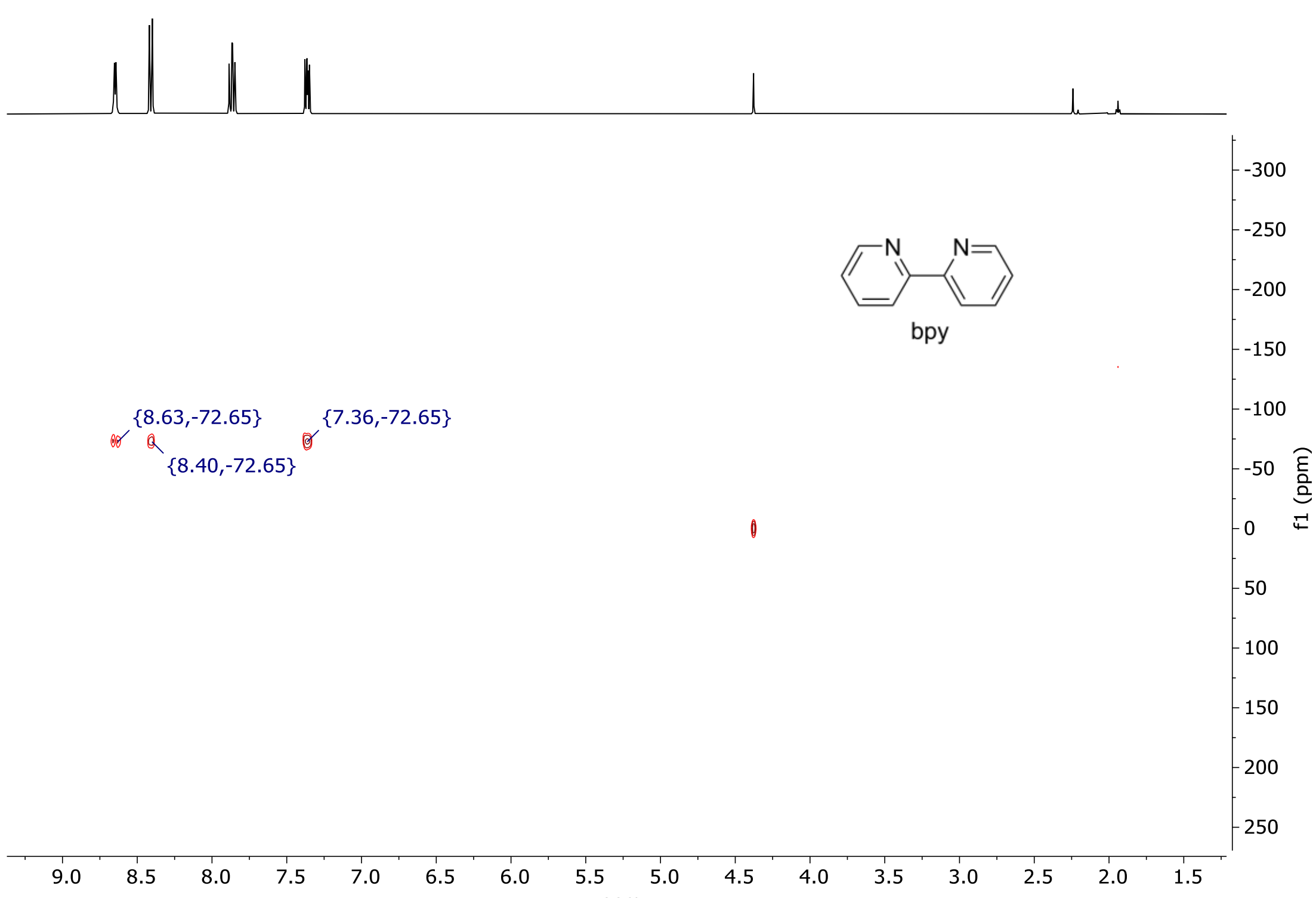

Figure S58. ^{1}H-^{15}N HMBC spectrum (^{1}H: 400 MHz, ^{15}N: 41 MHz; CD_3CN) for **bpy**.

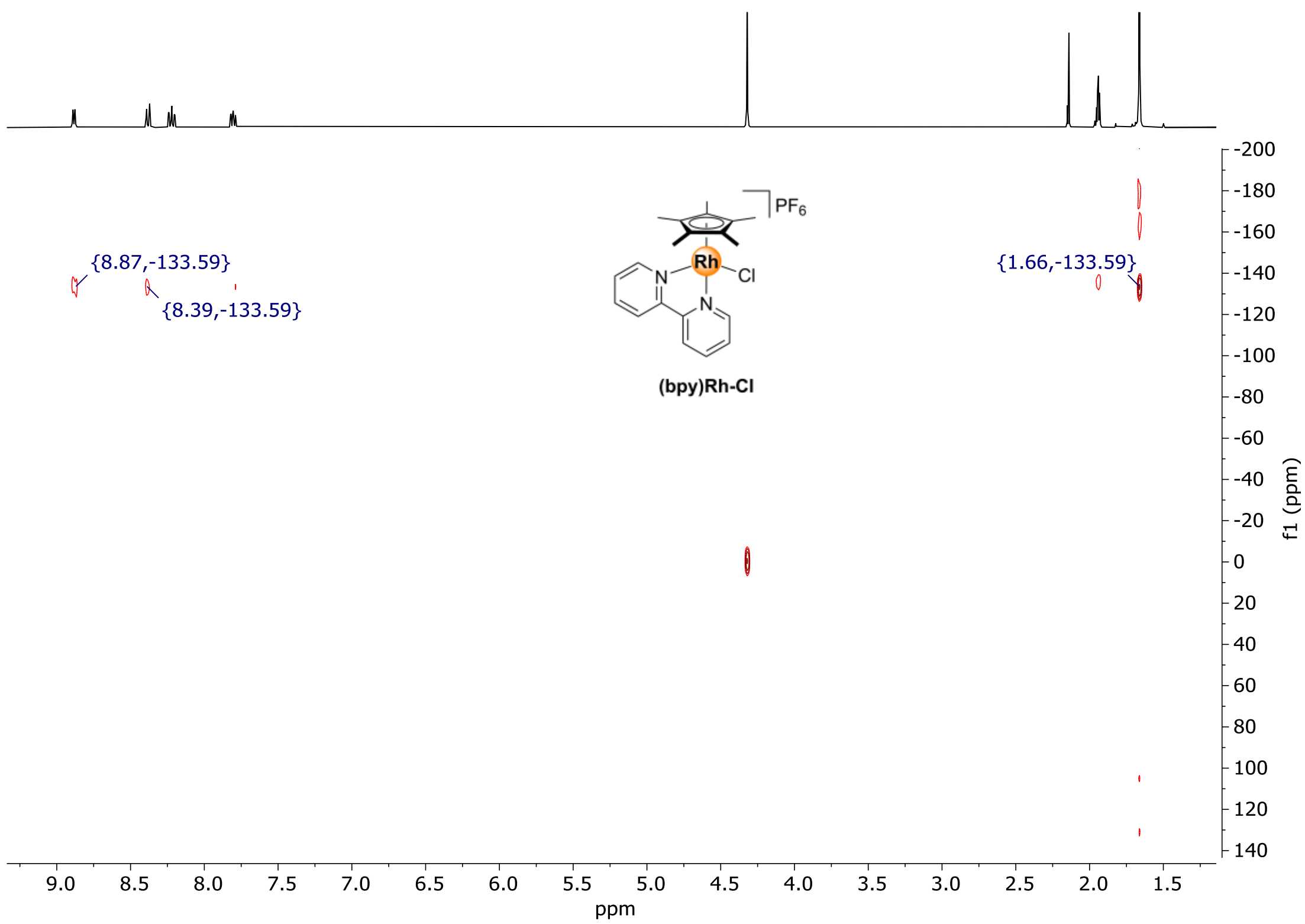

Figure S59. ^{1}H-^{15}N HMBC spectrum (^{1}H: 400 MHz, ^{15}N: 41 MHz; CD_3CN) for **(bpy)Rh-Cl**.

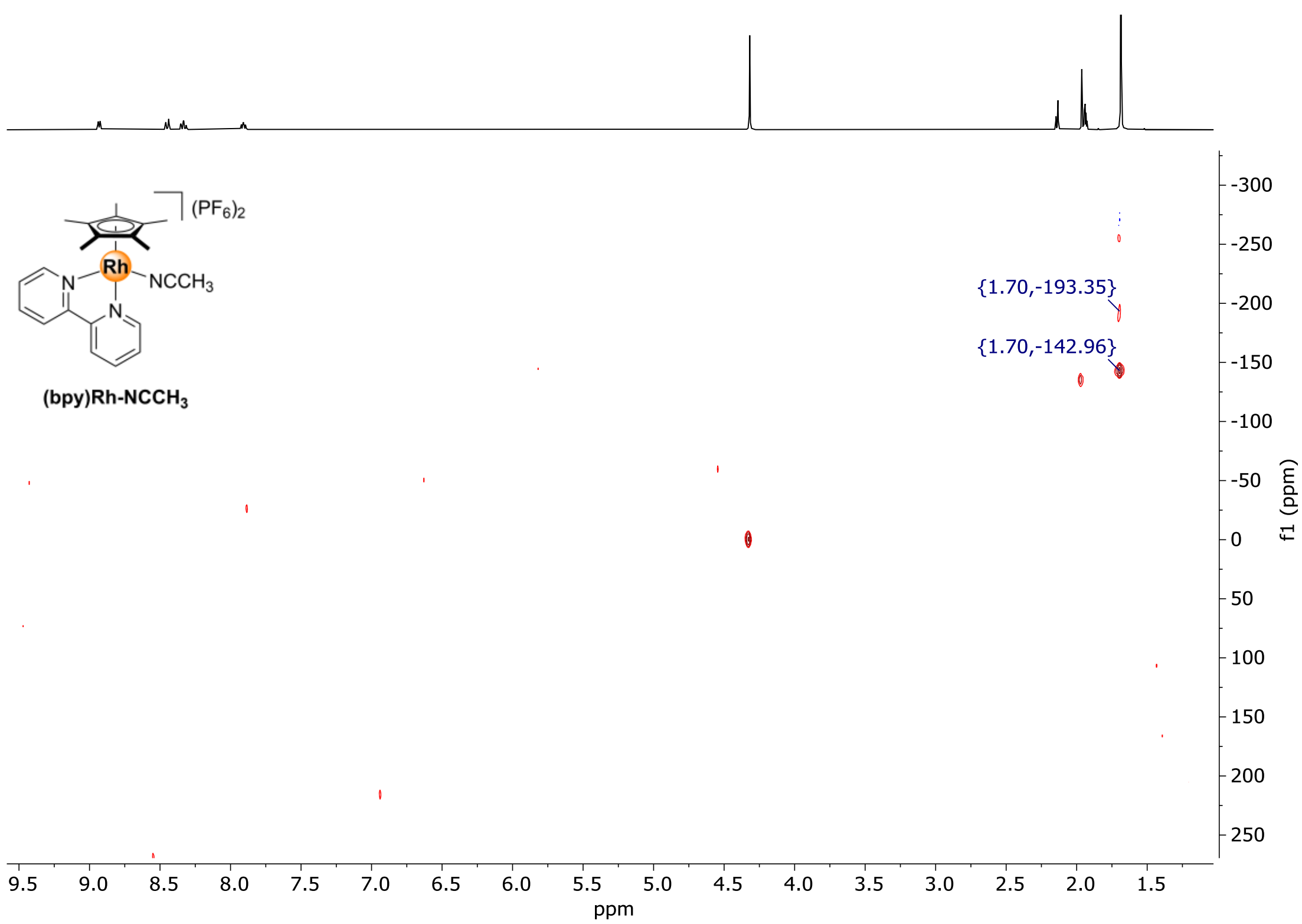

Figure S60. ^{1}H-^{15}N HMBC spectrum (^{1}H: 400 MHz, ^{15}N: 41 MHz; CD_3CN) for **(bpy)Rh-NCCH$_3$**.

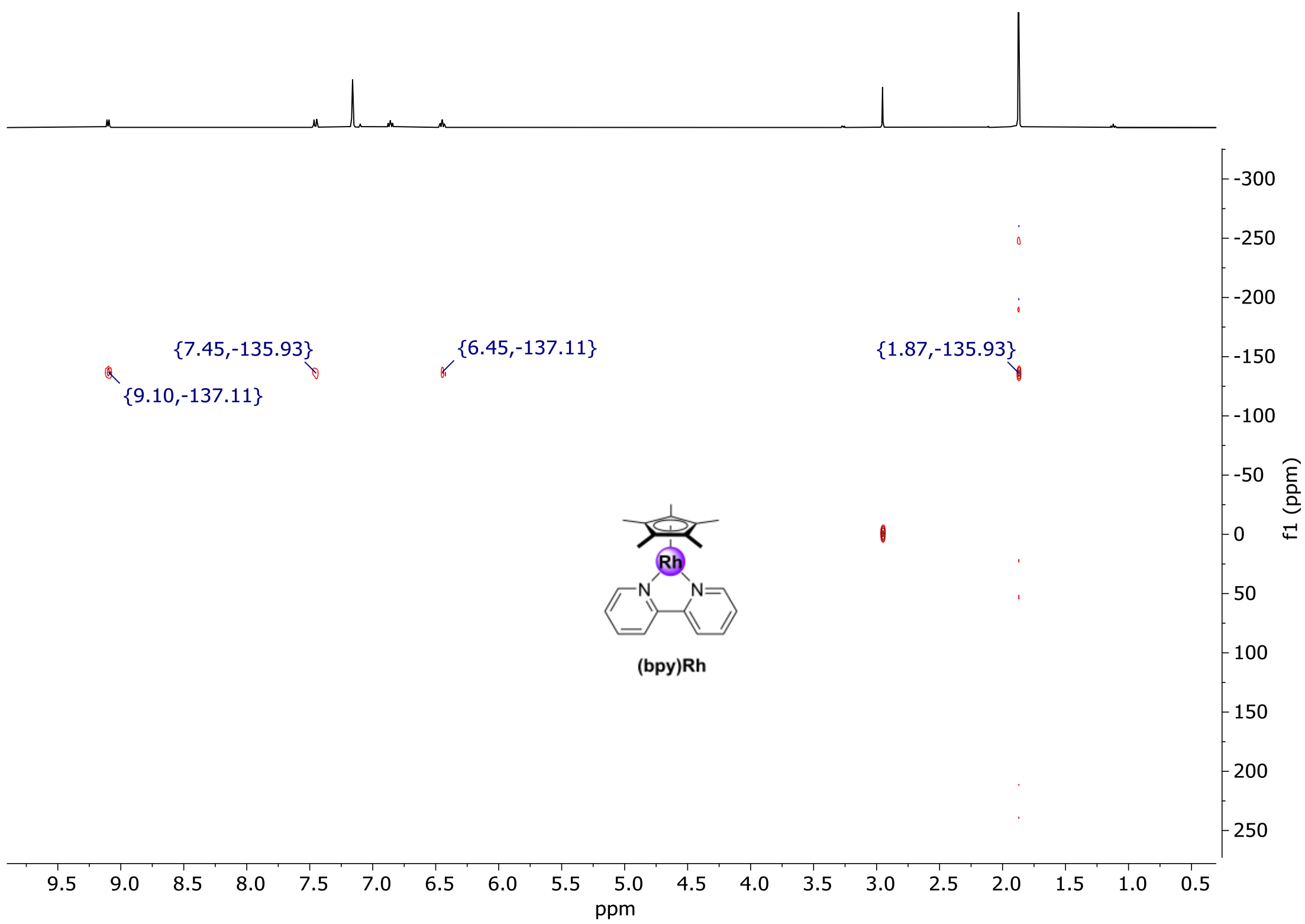

Figure S61. ^{1}H-^{15}N HMBC spectrum (^{1}H: 400 MHz, ^{15}N: 41 MHz; CD_3CN) for **(bpy)Rh**.

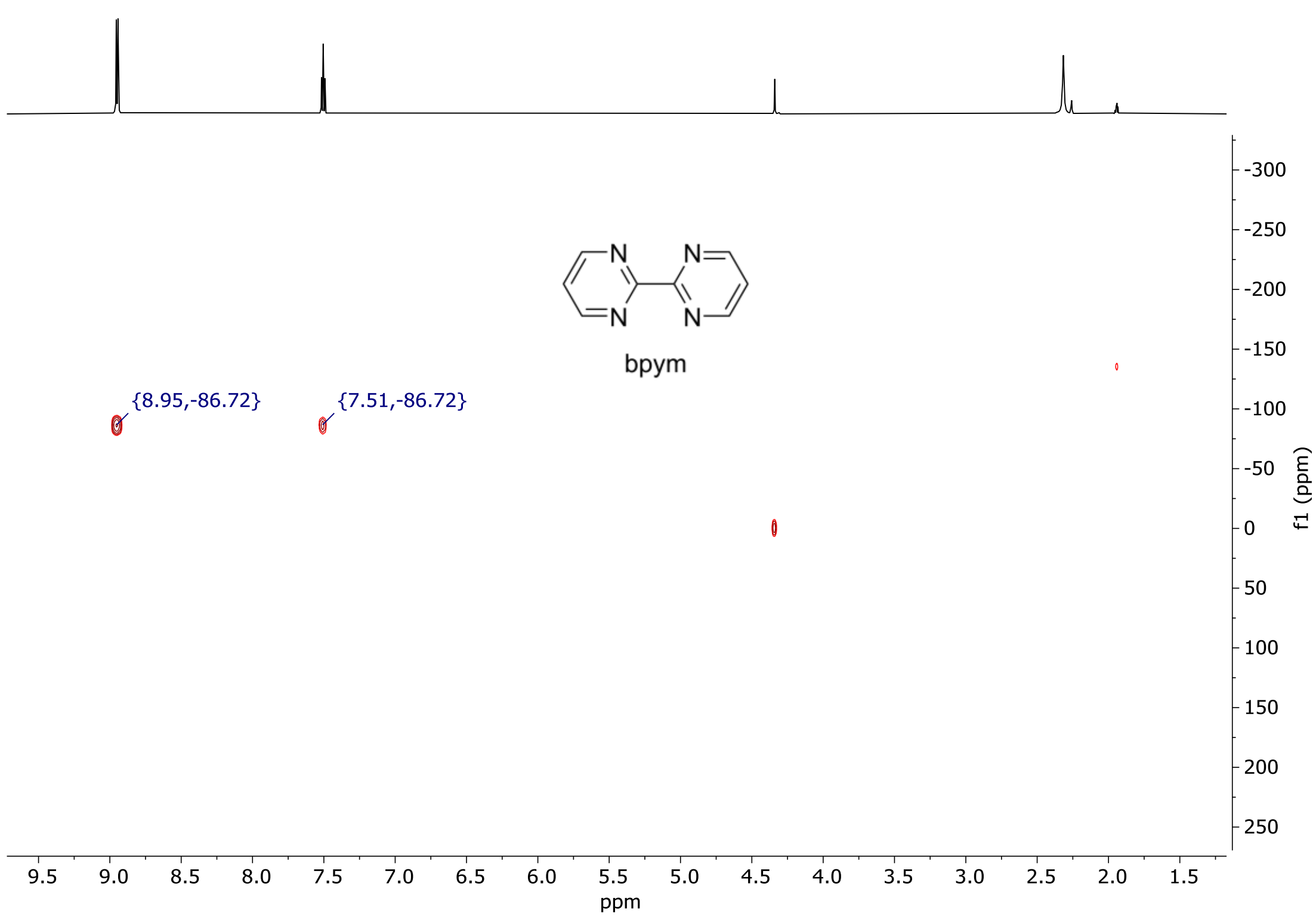

Figure S62. ^{1}H-^{15}N HMBC spectrum (^{1}H: 400 MHz, ^{15}N: 41 MHz; CD_3CN) for **bpym**.

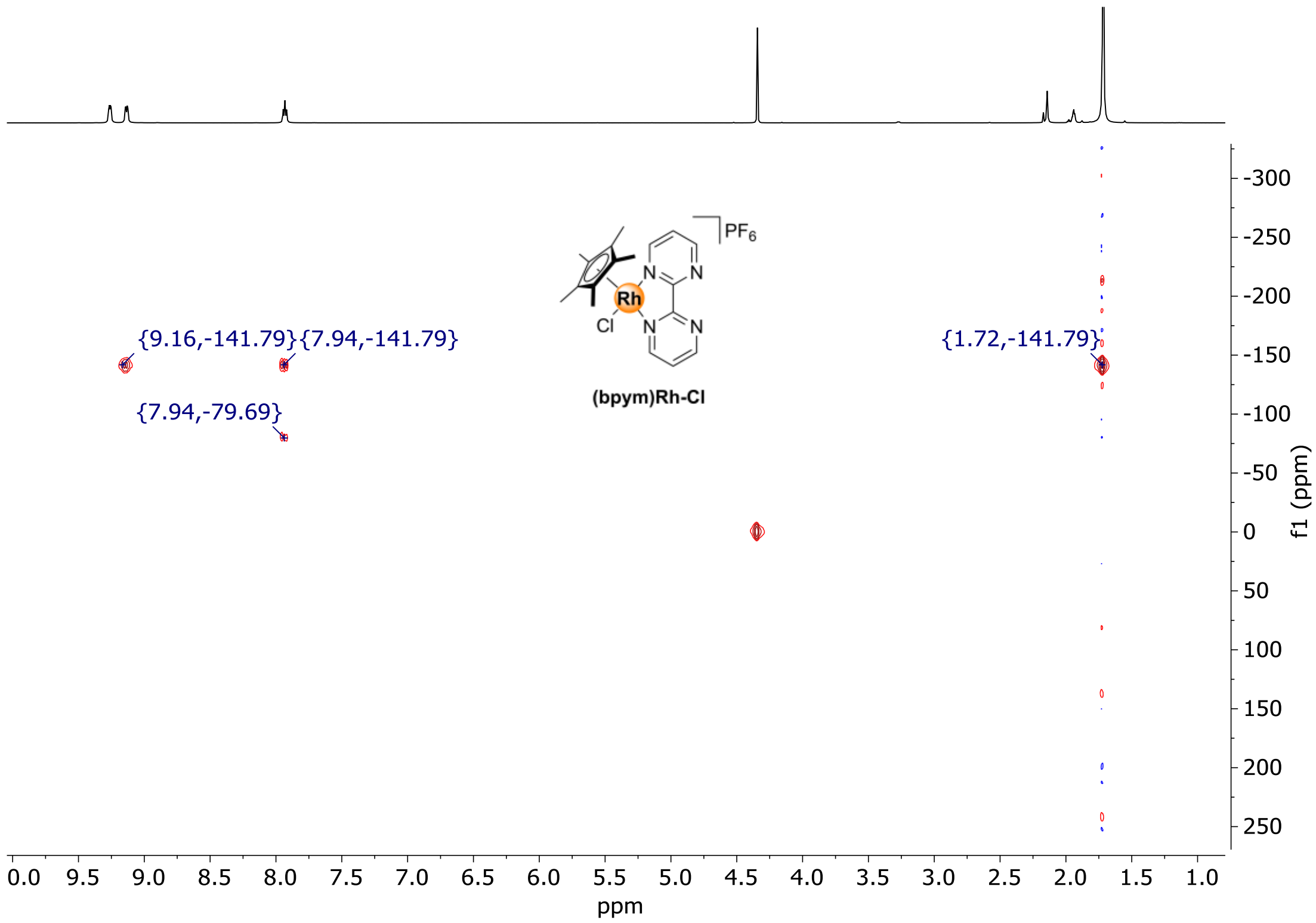

Figure S63. ^{1}H-^{15}N HMBC spectrum (^{1}H: 400 MHz, ^{15}N: 41 MHz; CD_3CN) for **(bpym)Rh-Cl**.

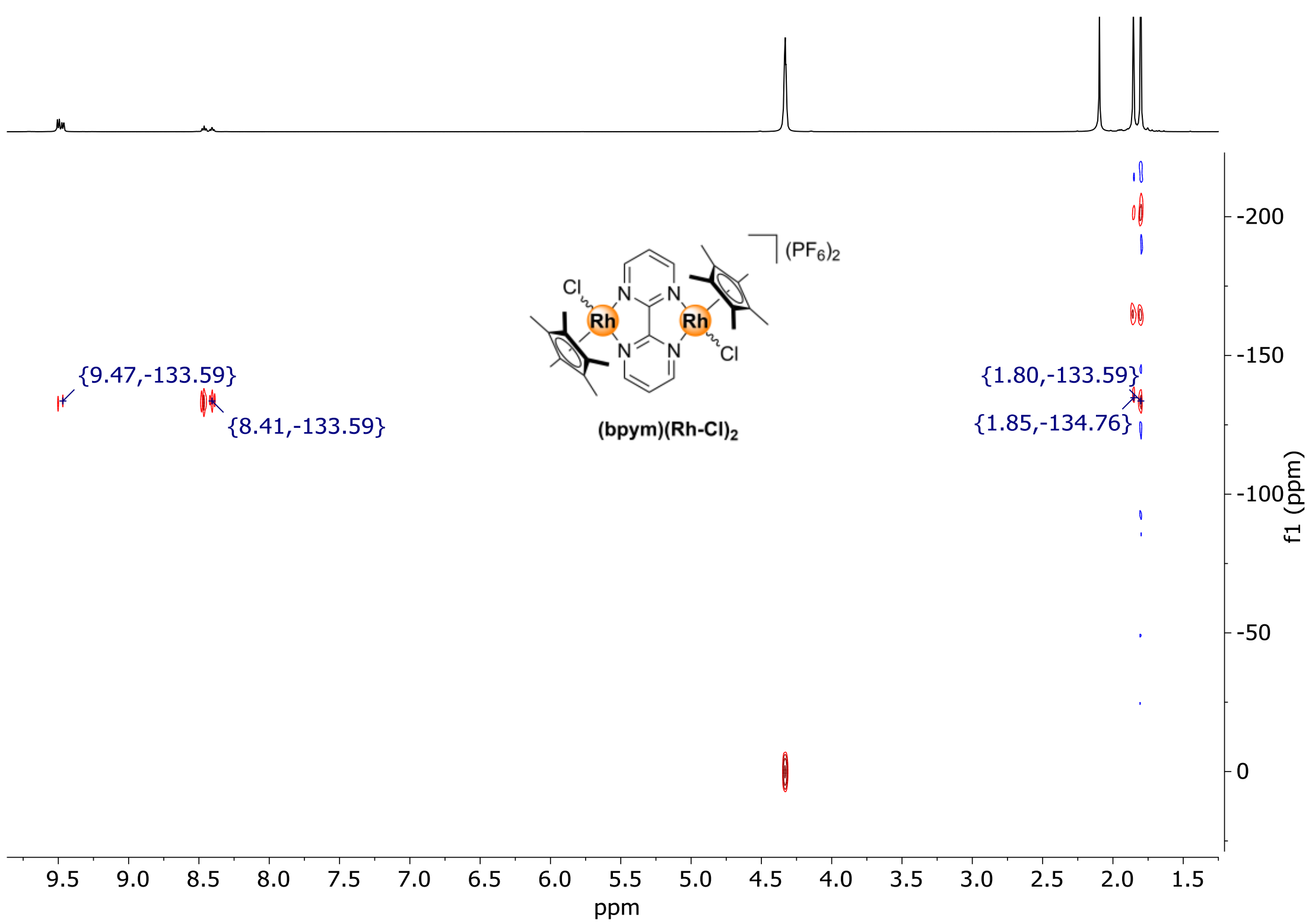

Figure S64. ^{1}H-^{15}N HMBC spectrum (^{1}H: 400 MHz, ^{15}N: 41 MHz; CD_3CN) for **(bpym)(Rh-Cl)$_2$**.

Table S1. Summary of ^{15}N data for compounds containing polyheterocyclic platforms in the present study.

Compound	^{15}N chemical shifts, ppm[a]				
	Rh–N_{py}	Rh–N_{pym}	$N_{pym,free}$	$N_{py,free}$	N_{NCCH3}
**bppm**	-	-	–97.26	–70.31	-
**(bppm)Rh-Cl**	–131.25	–147.65	–89.06	–69.14	-
**(bppm)Rh-NCCH$_3$**	–140.62	–157.03	–87.89	–69.14	–205.07
**(bppm)Rh**	–135.35	–152.63	[b]	[b]	-
**(bppm)(Rh-Cl)$_2$**	–130.07	–138.28	-	-	-
**(bppm)(Rh-NCCH$_3$)$_2$**	–139.45	–145.31	-	-	–207.90, –208.59[c]
**bpy**	-	-	-	–72.65	-
**(bpy)Rh-Cl**	–133.59	-	-	-	-
**(bpy)Rh-NCCH$_3$**	–142.96	-	-	-	–193.35
**(bpy)Rh**	–135.93	-	-	-	-
**bpym**	-	-	–86.72	-	-
**(bpym)Rh-Cl**	-	–141.79	–79.69	-	-
**(bpym)Rh-NCCH$_3$**	-	–151.17	–78.51	-	–206.24
**(bpym)(Rh-Cl)$_2$**	-	–133.59, –134.76[c]	-	-	-

[a] py = pyridine; pym = pyrimidine; [b] weak signals not observed; [c] shifts for major/minor diastereomers, respectively.

Discussion of ^{15}N NMR data

^{15}N NMR chemical shifts–determined via indirect detection in ^1H-^{15}N heteronuclear multiple bond correlation (HMBC) experiments and summarized in Table S1–provide insight into the chemical environment of the bppm and bpym ligands as a function of Rh coordination and oxidation state. In free bppm, the pyridine and pyrimidine N atoms appear at –70.31 and –97.26 ppm, respectively, consistent with the chemical shifts for the N atoms in the 2,2'-bipyridine (bpy, –72.65 ppm) and 2,2'-bipyrimidine (bpym, –86.72 ppm) ligands. Binding of [Cp*Rh] to one side of the bppm platform results in four different signals in the ^{15}N NMR data for **(bppm)Rh-Cl**. The signals corresponding to the Rh-bound N atoms were observed at –131.25 and –147.65 ppm; the 50-60 ppm upfield shifts upon complexation are consistent with previous reports for polypyridyl ligands.[1] Data collected for the literature complex **(bpy)Rh-Cl** (Chart 2) revealed a chemical shift for the N atoms in the bpy framework similar to the pyridine motifs in **(bppm)Rh-Cl** (–133.59 ppm). The signals for the non-coordinated N sites, which were often more difficult to detect in HMBC experiments, were observed at similar chemical shifts to free bppm (–89.06 and –70.31 ppm). In **(bppm)(Rh-Cl)$_2$**, only two ^{15}N signals are observed (–130.07 and –138.28 ppm), consistent with resymmetrization of the bppm scaffold.

Replacement of $Cl^–$ for $NCCH_3$ results in a ~10 ppm upfield shift in the resonances for the Rh-bound N atoms as determined from ^{15}N NMR data, while only minor effects were observed for the unbound N sites. Characterization of the literature complex **(bpy)Rh-NCCH$_3$** (Chart 2) revealed a similar shift in ^{15}N NMR (~10 ppm) from its chloride analogue. Exchange of the bound acetonitrile ligand appears to be facile in both **(bppm)Rh-NCCH$_3$** and **(bpy)Rh-NCCH$_3$**: no signals are observed for Rh-bound CH_3CN in ^1H NMR data collected in CD_3CN, though the ligated solvent can be detected in ^{15}N data (~ –200 ppm).

^{15}N NMR data revealed only minor effects of Rh oxidation state on the chemical shift of the bound N atoms, which appeared within ~5 ppm of those in the **(bppm)Rh-X** Rh(III) complexes. This observation

was corroborated by analysis of the corresponding bpy complex, **(bpy)Rh**, for which similarly minor changes are observed in the ^{15}N data upon reduction (Table S1). This is also consistent with the minimal effects of Rh oxidation state on the ^{31}P chemical shift for bis(phosphine) ligands.[2-4] In our hands, the signals corresponding to the non-Rh bound N atoms in bppm complexes are typically less intense than those for Rh-bound portions of the ligand; as these signals could not be detected for **(bppm)Rh**, internal comparison of chemical shifts between bound and unbound portions of the ligand could not be carried out.

^{15}N NMR analysis of **(bpym)Rh-Cl** and **(bpym)(Rh-Cl)$_2$** revealed similar profiles to the pyrimidine portion of the bppm framework and its complexes. The N atoms in free bpym appear at –86.72 ppm, more upfield than those in the pyridyl groups in bpy and similar to those for the pyrimidine fragment in bppm (Table S1). Binding of [Cp*Rh] to one of the bidentate sites results in substantial shielding of the N atoms bound to Rh (observed at –141.79 ppm); only a modest shift was observed for the unbound N atoms (–79.69 ppm). In **(bpym)(Rh-Cl)$_2$**, all four N atoms are chemically equivalent, appearing near –134 ppm in both diastereomers of this complex–a smaller upfield shift vs. free bpym than in **(bpym)Rh-Cl**, similarly to what is observed for **(bppm)(Rh-Cl)$_2$** vs. **(bppm)Rh-Cl**. As in the analogous complexes of the bppm ligand, exchange of Cl^- for CH_3CN in **(bpym)Rh-NCCH$_3$** resulted in only minor effects on ^{15}N chemical shifts. As in **(bppm)Rh-NCCH$_3$**, a signal corresponding to the solvent ligand N atom was observed at –206.24 ppm for **(bpym)Rh-NCCH$_3$**, further highlighting the similar nature of these two complexes.

Other NMR data

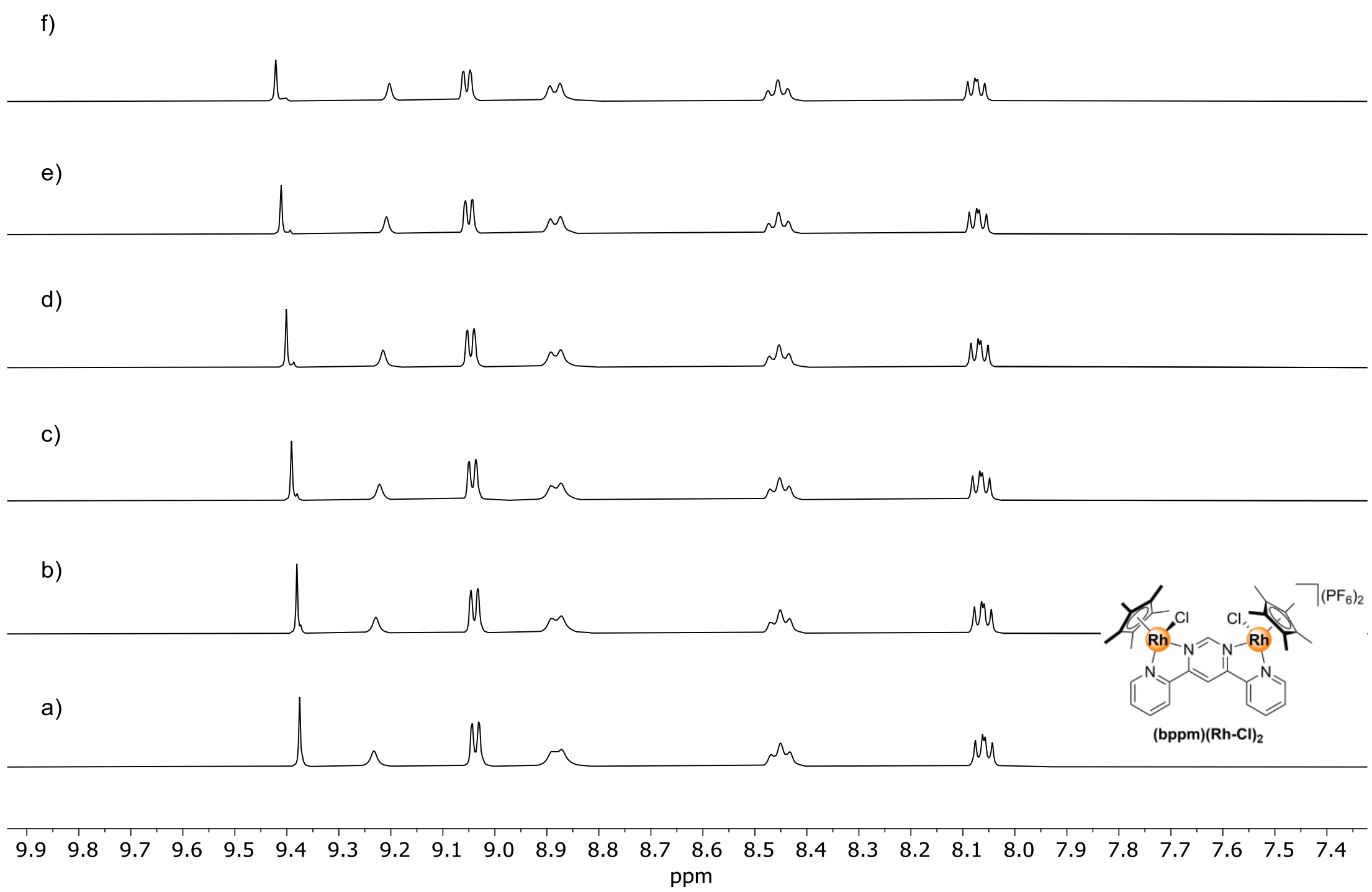

Figure S65. VT ^{1}H NMR spectra (400 MHz, CD_3CN) of **(bppm)(Rh-Cl)$_2$**; (a) 298 K; (b) 303 K; (c) 313 K; (d) 323 K; (e) 333 K; (f) 343 K.

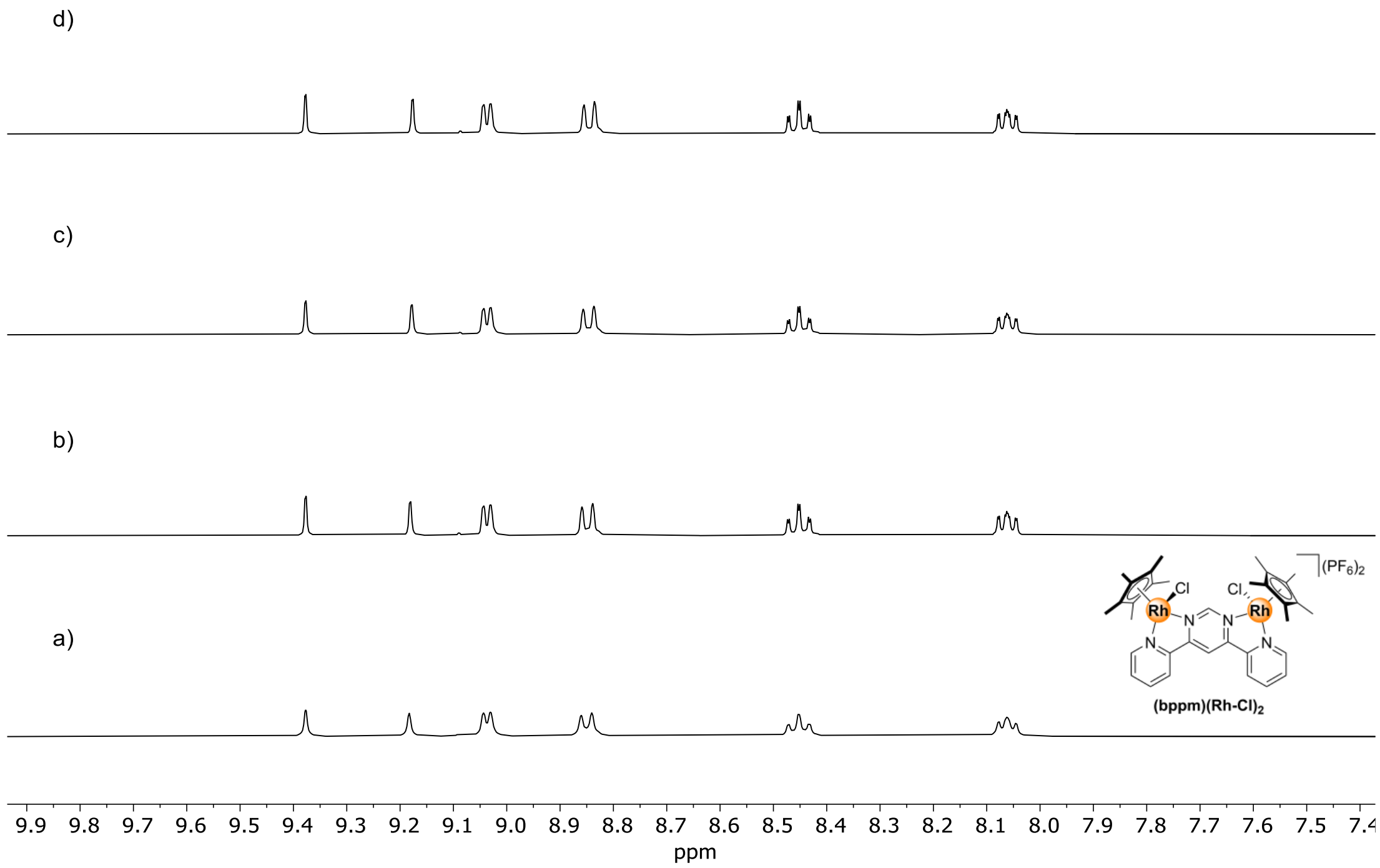

Figure S66. ^{1}H NMR spectra for **(bppm)(Rh-Cl)$_2$** after heating; (a) immediately after sample preparation; (b) after heating at 70 °C for 2 hours; (c) after heating at 70 °C for 21 hours; (d) after heating at 70 °C for 40 hours.

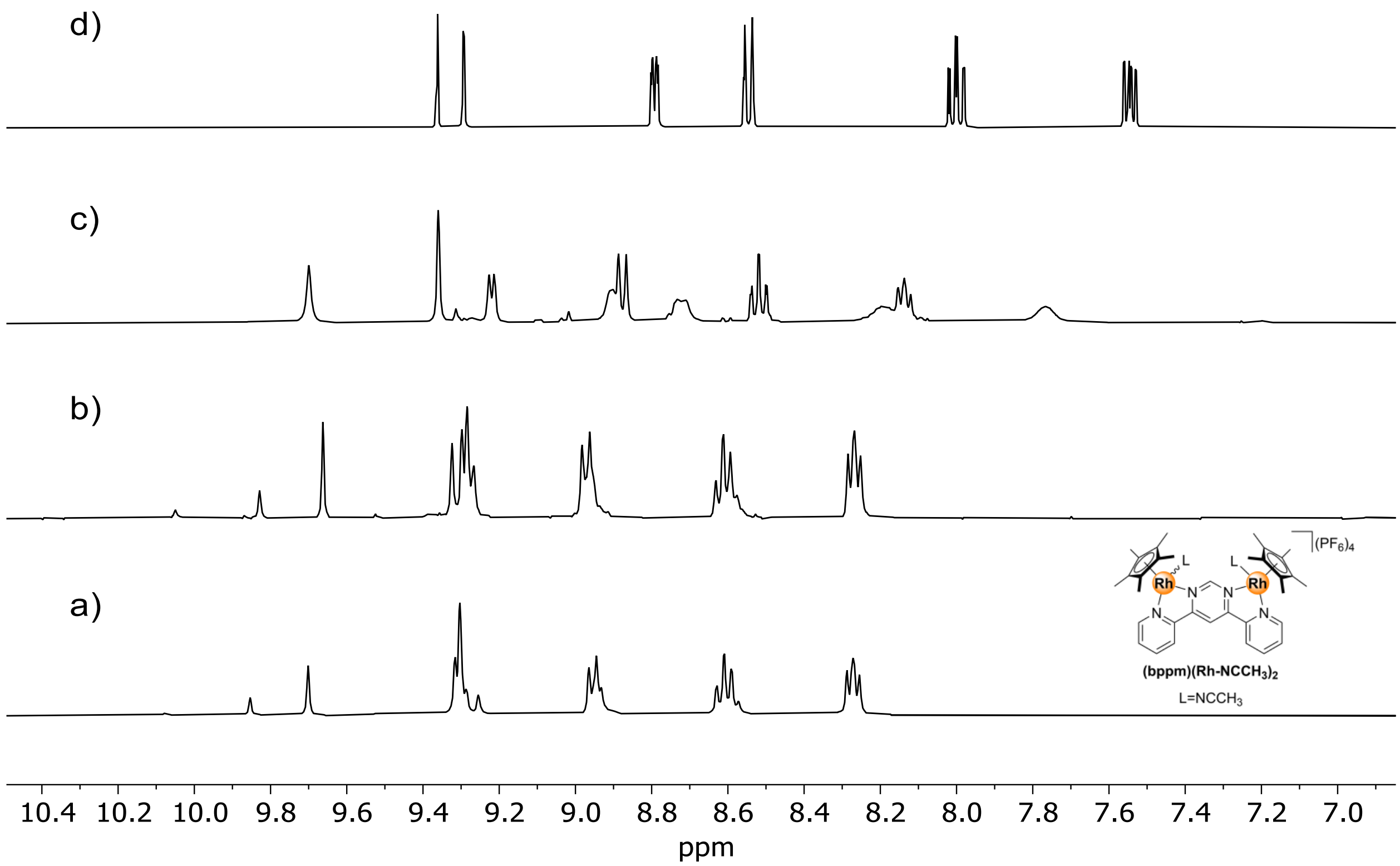

Figure S67. ^{1}H NMR (400 MHz, CD_3NO_2) monitoring of reaction of **(bppm)(Rh-NCCH$_3$)$_2$** with CD_3CN; (a) ^{1}H NMR spectrum of **(bppm)(Rh-NCCH$_3$)$_2$**; (b) ^{1}H NMR spectrum after addition of CD_3CN (~10 % v/v), 10 minutes; (c) ^{1}H NMR spectrum of **(bppm)Rh-NCCH$_3$**; (d) ^{1}H NMR spectrum of bppm.

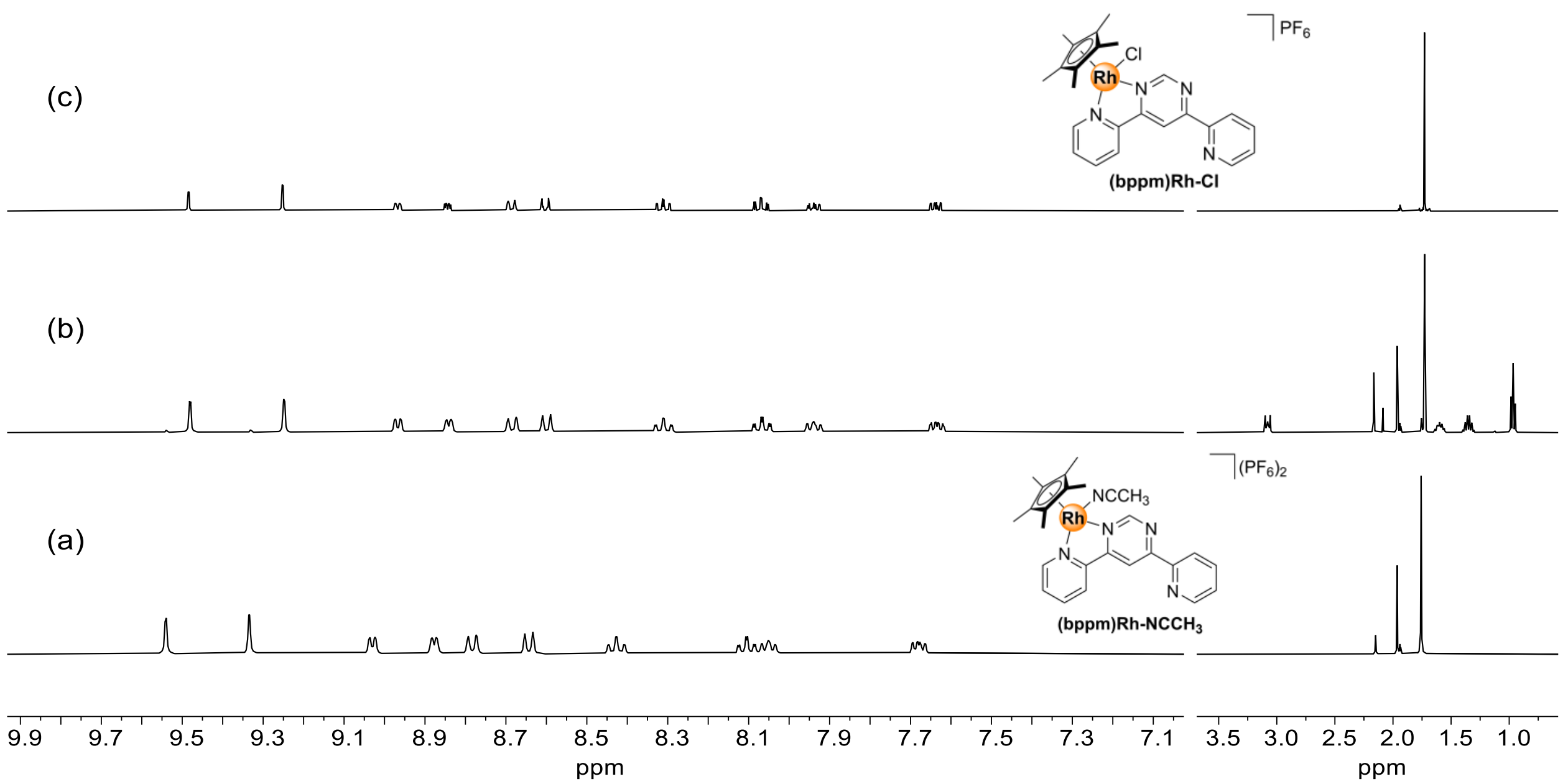

Figure S68. ^{1}H NMR (400 MHz, CD_3CN) monitoring of reaction of **(bppm)Rh-NCCH$_3$** with $^nBu_4NCl$; (a) ^{1}H NMR spectrum of **(bppm)Rh-NCCH$_3$**; (b) ^{1}H NMR spectrum after addition of 1 equiv. $^nBu_4NCl$; (c) ^{1}H NMR spectrum after addition of 2 equiv. $^nBu_4NCl$.

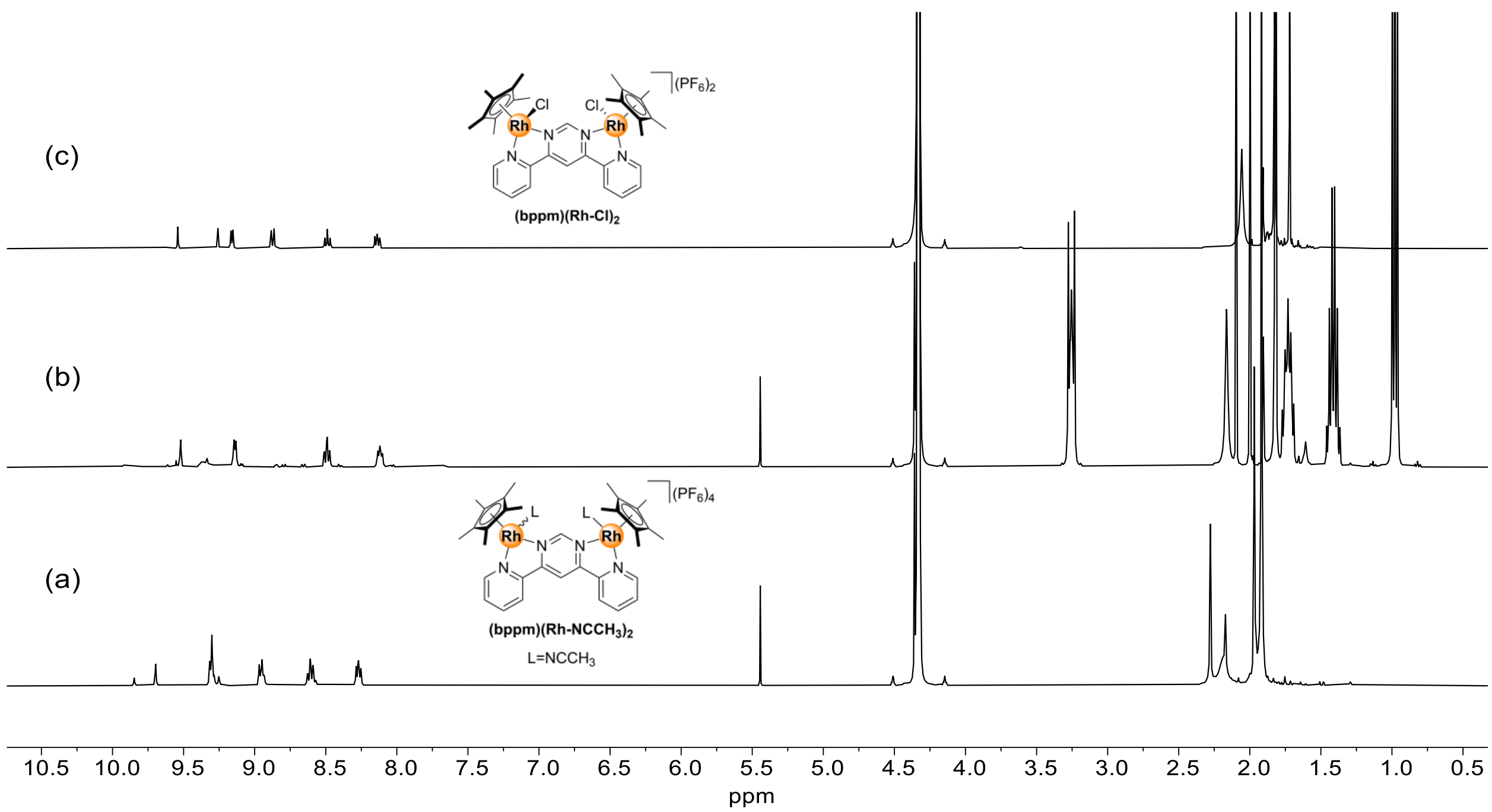

Figure S69. ^{1}H NMR (400 MHz, CD_3NO_2) monitoring of reaction of **(bppm)(Rh-NCCH$_3$)$_2$** with $^{n}Bu_4NCl$; (a) ^{1}H NMR spectrum of **(bppm)(Rh-NCCH$_3$)$_2$**; (b) ^{1}H NMR spectrum after addition of 1 equiv. $^{n}Bu_4NCl$; (c) ^{1}H NMR spectrum of **(bppm)(Rh-Cl)$_2$**.

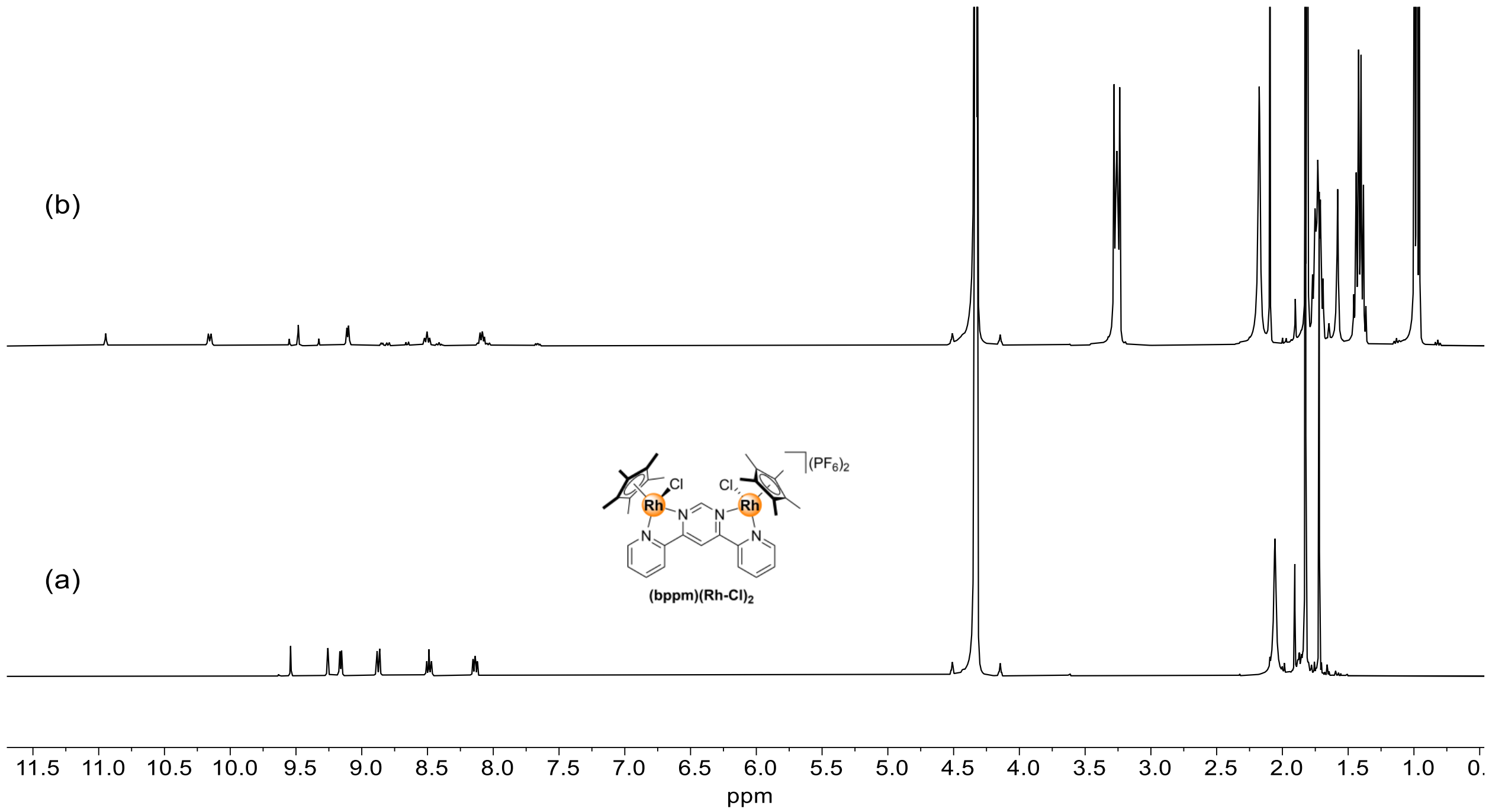

Figure S70. ^{1}H NMR (400 MHz, CD_3NO_2) monitoring of reaction of **(bppm)(Rh-Cl)₂** with $^{n}Bu_4NCl$; (a) ^{1}H NMR spectrum of **(bppm)(Rh-Cl)₂**; (b) ^{1}H NMR spectrum after addition of 1 equiv. $^{n}Bu_4NCl$.

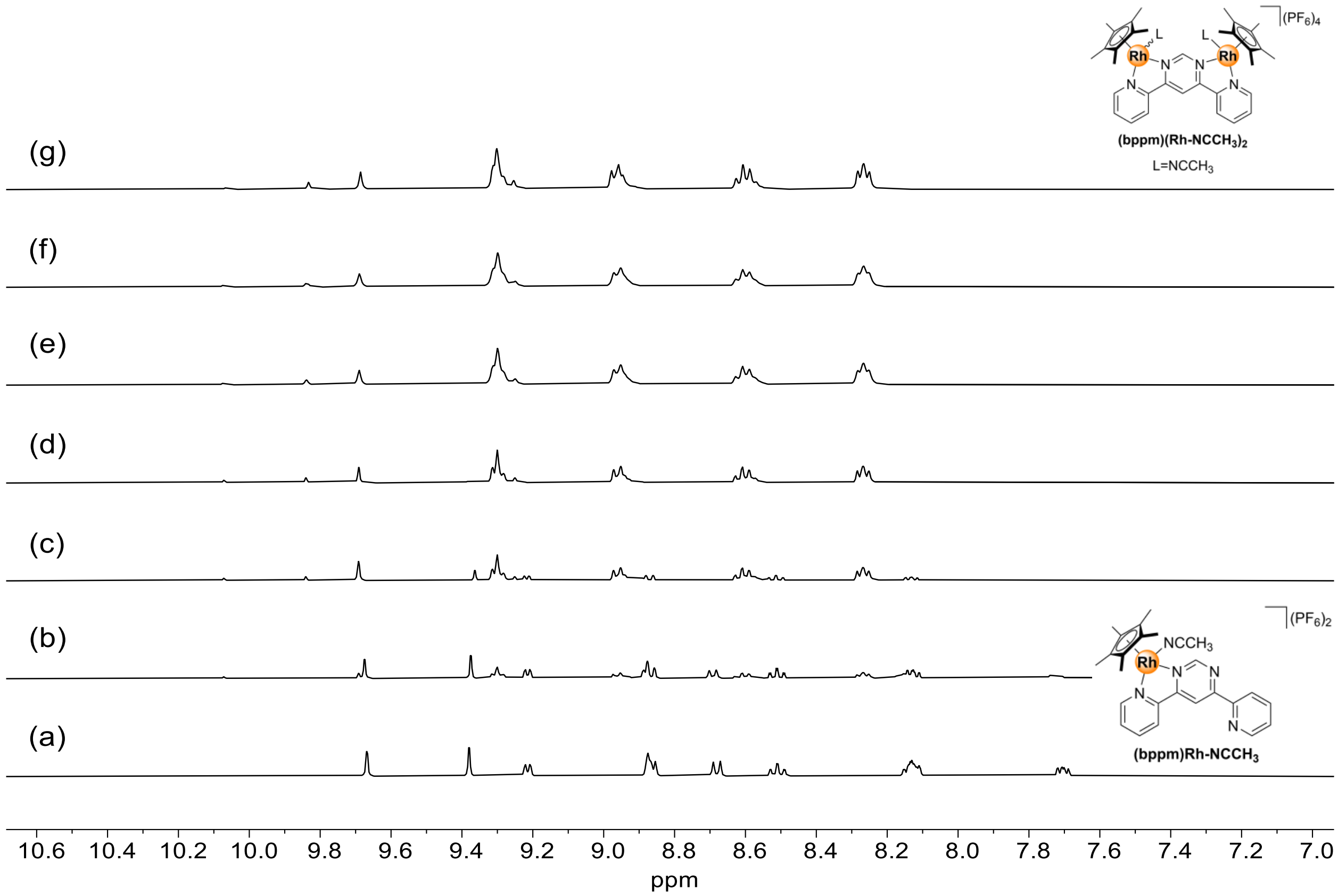

Figure S71. ^{1}H NMR (400 MHz, CD_3NO_2) monitoring of reaction of **(bppm)Rh-NCCH₃** with $[Cp^*Rh(NCCH_3)_3]^{2+}$; (a) **(bppm)Rh-NCCH₃**; (b) +0.33 equiv. $[Cp^*Rh(NCCH_3)_3]^{2+}$; (c). +0.67 equiv. $[Cp^*Rh(NCCH_3)_3]^{2+}$; (d) +1.0 equiv. $[Cp^*Rh(NCCH_3)_3]^{2+}$; (e) +1.33 equiv. $[Cp^*Rh(NCCH_3)_3]^{2+}$; (f) +1.67 equiv. $[Cp^*Rh(NCCH_3)_3]^{2+}$; (g) ^{1}H NMR spectrum of **(bppm)(Rh-NCCH₃)₂**.

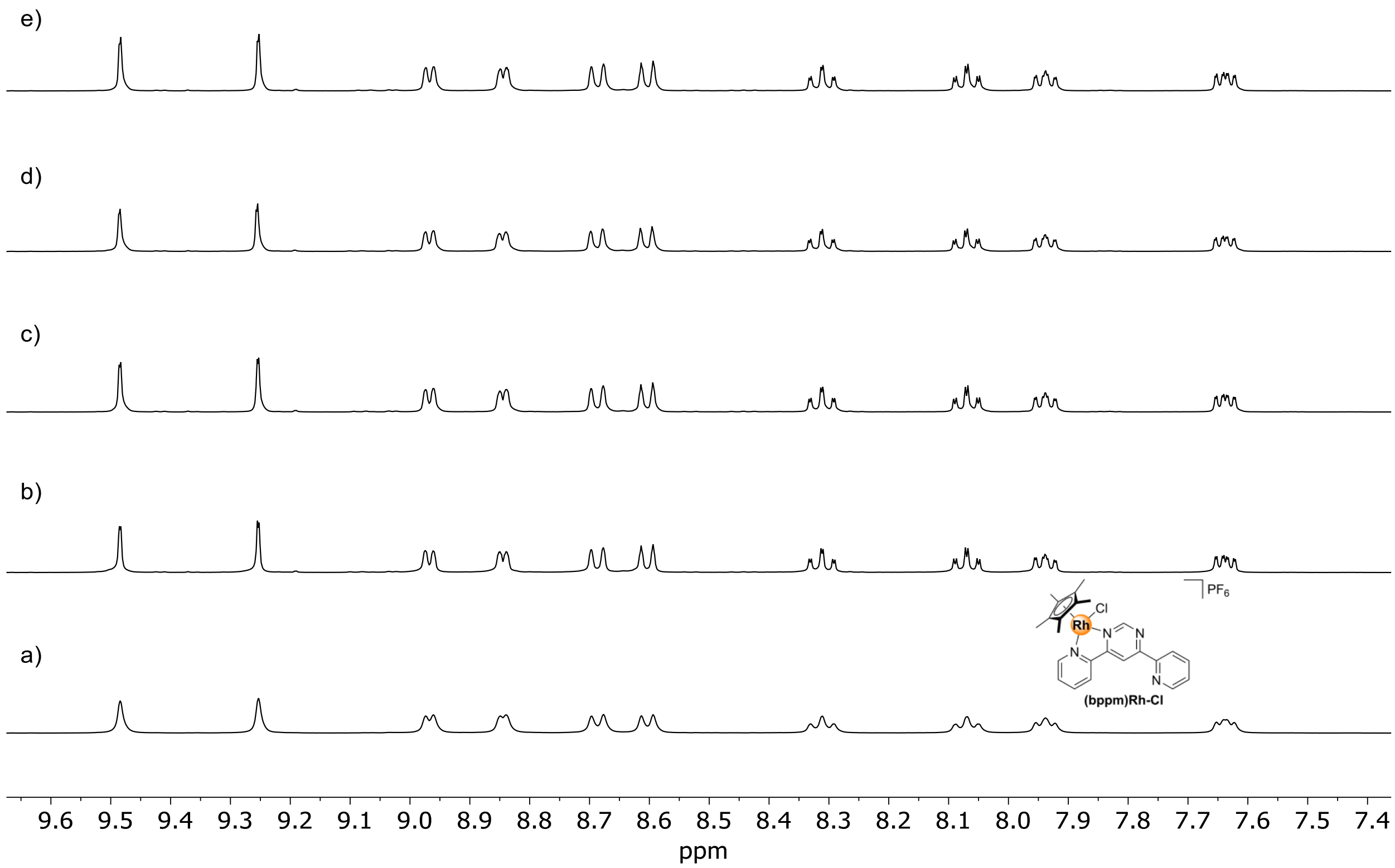

Figure S72. ^{1}H NMR (400 MHz, CD_3CN) data for **(bppm)Rh-Cl** under heating; (a) **(bppm)Rh-Cl**; (b) 70 °C, 1 h; (c) 70 °C, 3 h; (d) 70 °C, 20 h; (e) 70 °C, 44 h. No reactivity is observed.

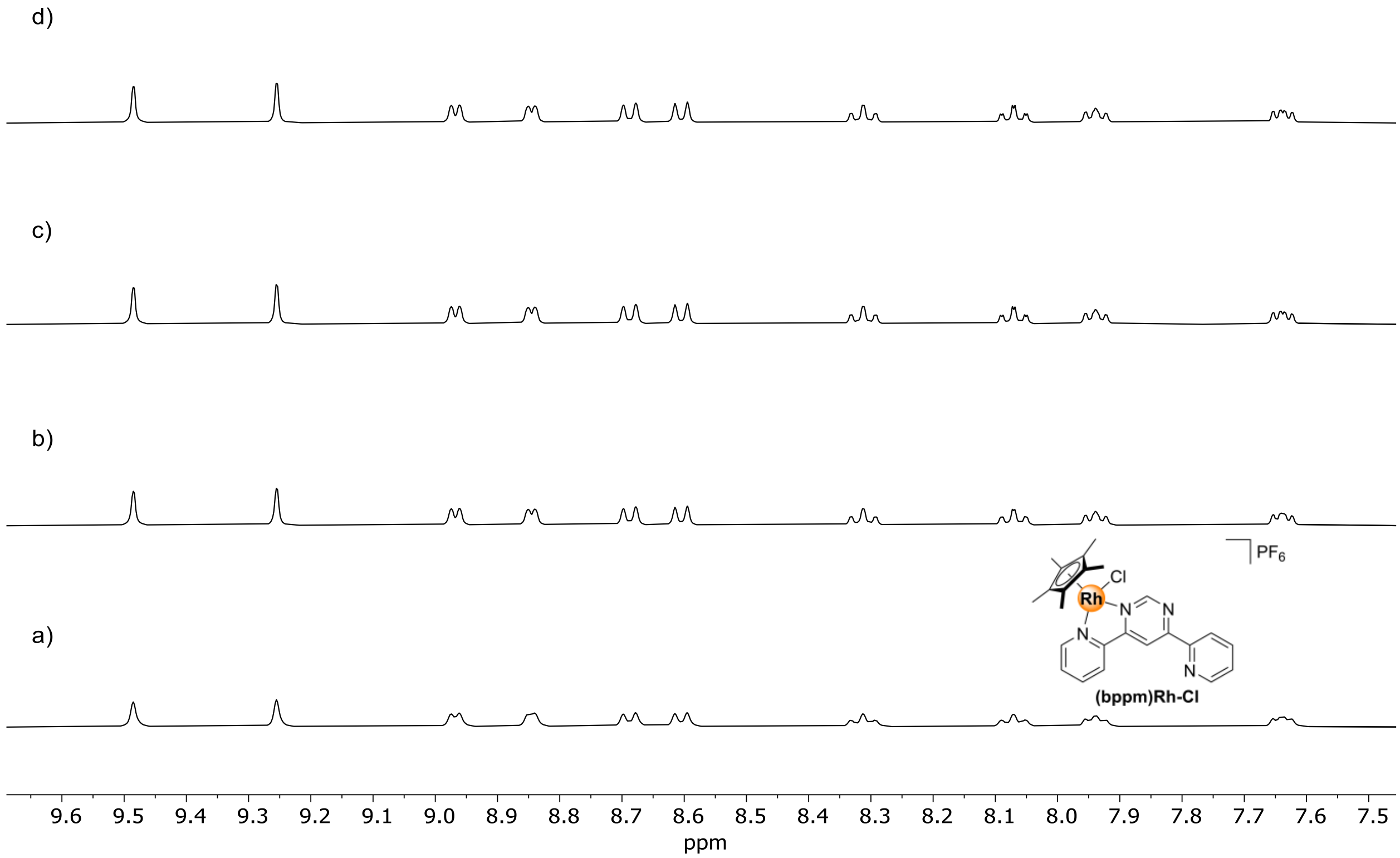

Figure S73. ^{1}H NMR (400 MHz, CD_3CN) data for **(bppm)Rh-Cl** under irradiation with light (CFL bulb); (a) **(bppm)Rh-Cl**; (b) hν, 1 h; (c) hν, 3 h; (d) hν, 20 h. No reactivity is observed.

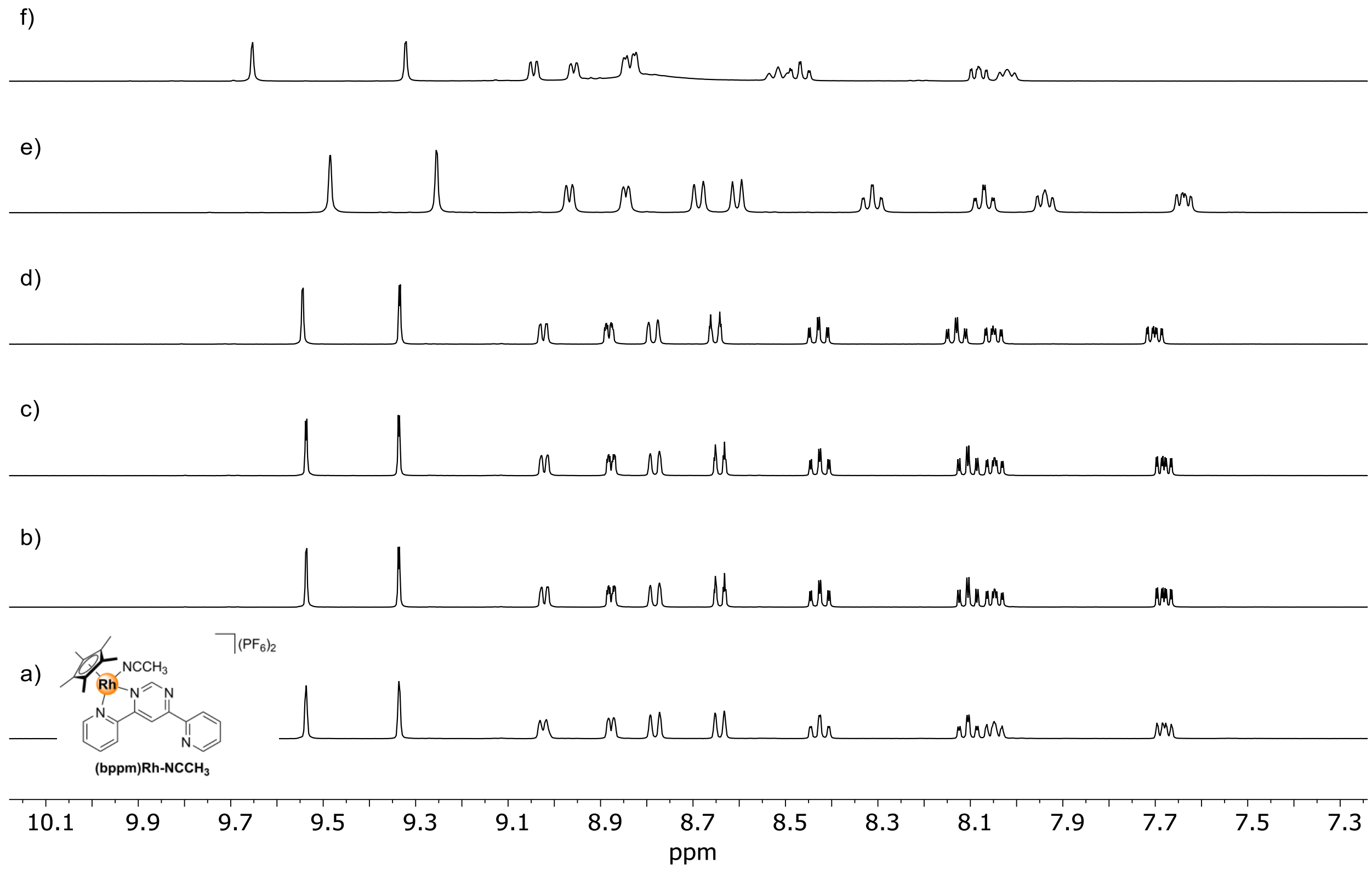

Figure S74. ^{1}H NMR (400 MHz, CD_3CN) data for **(bppm)Rh-NCCH$_3$** under heating; (a) **(bppm)Rh-NCCH$_3$**; (b) 70 °C, 1 h; (c) 70 °C, 3 h; (d) 70 °C, 20 h; (e) 70 °C, 44 h; (f) 70 °C, 51 h. The shifts observed for the resonances corresponding to **(bppm)Rh-NCCH$_3$** at longer times are accompanied by formation of a precipitate, suggest partial decomposition upon prolonged heating.

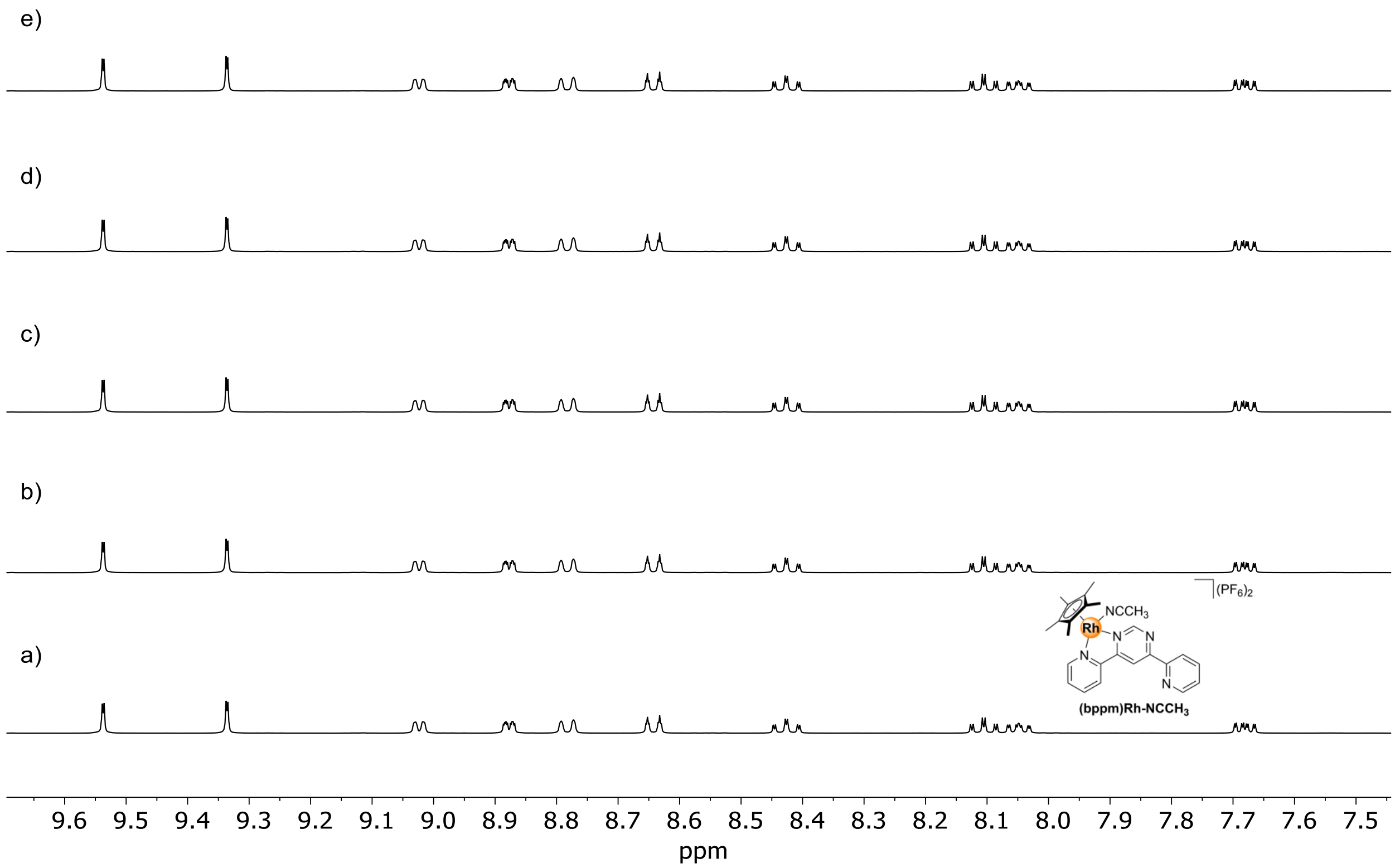

Figure S75. ^1H NMR (400 MHz, CD_3CN) data for **(bppm)Rh-NCCH$_3$** under irradiation with light (CFL bulb); (a) **(bppm)Rh-NCCH$_3$**; (b) hν, 1 h; (c) hν, 3 h; (d) hν, 20 h; (e) hν, 44 h. No reactivity is observed.

Discussion of studies of bidentate ligand exchange at the Rh(III) state.

To determine whether analogous ligand exchange reactivity to that observed upon reduction of bppm complexes could be engendered at the Rh(III) state, the stability of **(bppm)Rh-Cl**, **(bppm)Rh-NCCH$_3$**, and **(bppm)(Rh-Cl)$_2$** in solution was assessed. While Rh(III) species are expected to be significantly more substitutionally inert than their Rh(II) analogues, ligand exchange at Rh(III) has routinely been leveraged in the synthesis of literature complexes,[5-7] and was in fact critical to the synthesis of solvento complex **(bppm)Rh-NCCH$_3$** from the tris(acetonitrile) precursor The very instability of **(bppm)(Rh-NCCH$_3$)$_2$** in solution likely also results from facile ligand substitution at Rh(III), which would promote conversion to other species. However, heating of samples of **(bppm)Rh-Cl** or **(bppm)Rh-NCCH$_3$**, and **(bppm)(Rh-Cl)$_2$** in in CD_3CN at 70 °C or irradiation with visible light did not result in significant conversion to complexes with different nuclearity over a timeframe of hours to days (Figures S56-S59). Generally, therefore, interconversion between mono- and dirhodium(III) complexes does not appear to be readily operative even under more forcing conditions. These observations underscore the role of ET in promoting ligand exchange in **(bppm)Rh-NCCH$_3$**.

Discussion of synthesis and stability of mono- and dirhodium bpym complexes

In our hands, synthesis of **(bpym)Rh-Cl** and **(bpym)(Rh-Cl)$_2$** required modifications of the procedures in either earlier report. For **(bpym)Rh-Cl**, a higher excess of bpym ligand was needed for a more reliable synthesis than the original procedure.[8] Conversely, exclusion of [^{n}Bu$_4$N][PF$_6$] and use of a larger excess of Rh (2.8 equiv. [Rh]/bpym) provided **(bpym)(Rh-Cl)$_2$** in better yield than either reported procedure for this compound.[8-9]

While in the original reports, synthesis of **(bpym)(Rh-Cl)$_2$** provided the *syn* and *anti* diastereomers of this complex in nearly equal amounts, our procedure yielded a ~3:2 ratio of the two diastereomers. This ratio remained constant across multiple replicates (Figure S76); increasing the reagent concentrations ([Rh] = 30 m*M* vs. ~5 m*M* in the standard procedure) likewise only marginally affected the isomer ratio (Figure S77), though a substantial amount of **(bpym)Rh-Cl** was also formed under these conditions. Heating samples of **(bpym)(Rh-Cl)$_2$** in CD_3NO_2 (85 °C) had minimal effects on the isomeric ratio over a timeframe of several hours (Figure S63). While **(bpym)(Rh-Cl)$_2$** was described as stable in a variety of organic solvents by Štěpnička and Süss-Fink,[8] Kaim and coworkers had reported its instability in coordinating media.[9] Our observations broadly agree with the report by Kaim: complex **(bpym)(Rh-Cl)$_2$** is stable in CH_3NO_2 solutions at room temperature over a timeframe of several days; conversely, addition of CD_3CN (20-40% v/v) to a CD_3NO_2 solution of **(bpym)(Rh-Cl)$_2$** indicated partial conversion to **(bpym)Rh-Cl** within minutes based on ^{1}H NMR analysis (Figure S64).The ratio between the two **(bpym)(Rh-Cl)$_2$** diastereomers was also affected, with the peaks corresponding to major isomer disappearing more quickly than those for the minor isomer, though we have been unable to determine whether this observation results from preferential decomposition of the major isomer or interconversion between diastereomers. The instability of the **(bpym)(Rh-Cl)$_2$** complex in the presence of exogenous ligands confirms the lability of the bpym framework to the second [Cp*Rh] moiety even at the Rh(III) state, likely due to the sharing of both heterocycle donor groups.

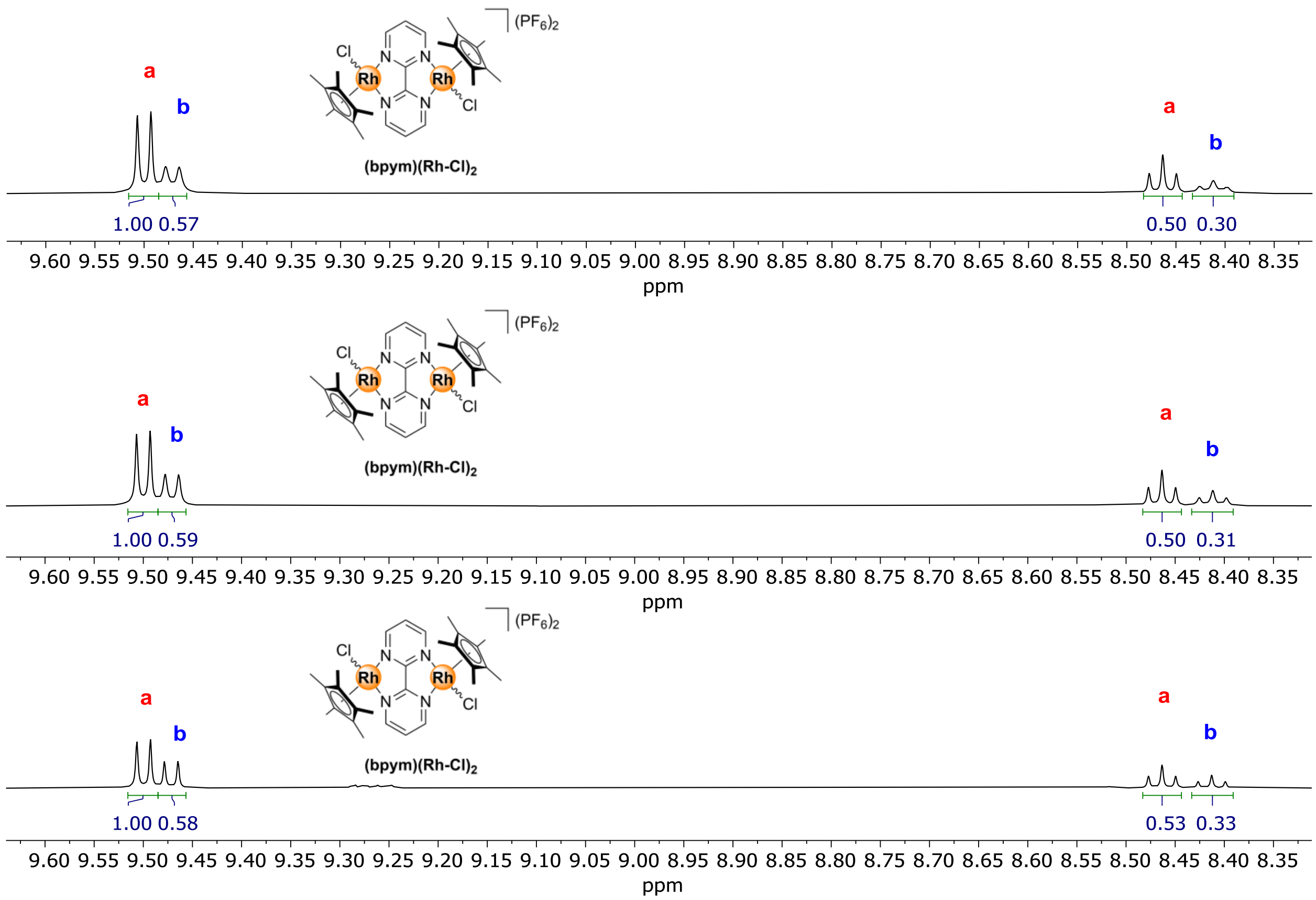

Figure S76. ^{1}H NMR spectra (400 MHz, CD_3NO_2) for **(bpym)(Rh-Cl)₂** (three synthesis replicates). The signals from the two diastereomers are labeled (a) and (b), respectively. The average diastereomeric ratio across the three replicates is 1.7:1.

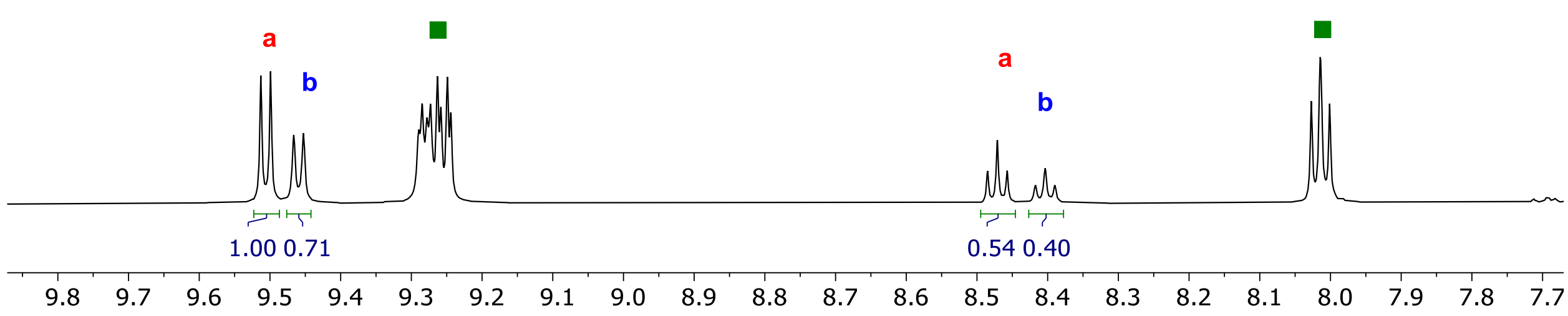

Figure S77. ^{1}H NMR spectrum (400 MHz, CD_3NO_2) for the material obtained from the synthesis of **(bpym)(Rh-Cl)₂** at higher concentration ([Rh] ≈ 30 m*M* vs. 5 m*M* in the typical procedure). Peaks marked with green squares correspond to **(bpym)Rh-Cl**, which also precipitates from the reaction solution under the more concentrated conditions. The ratio between the **(bpym)(Rh-Cl)₂** diastereomers (**a** and **b**) is 1.4.

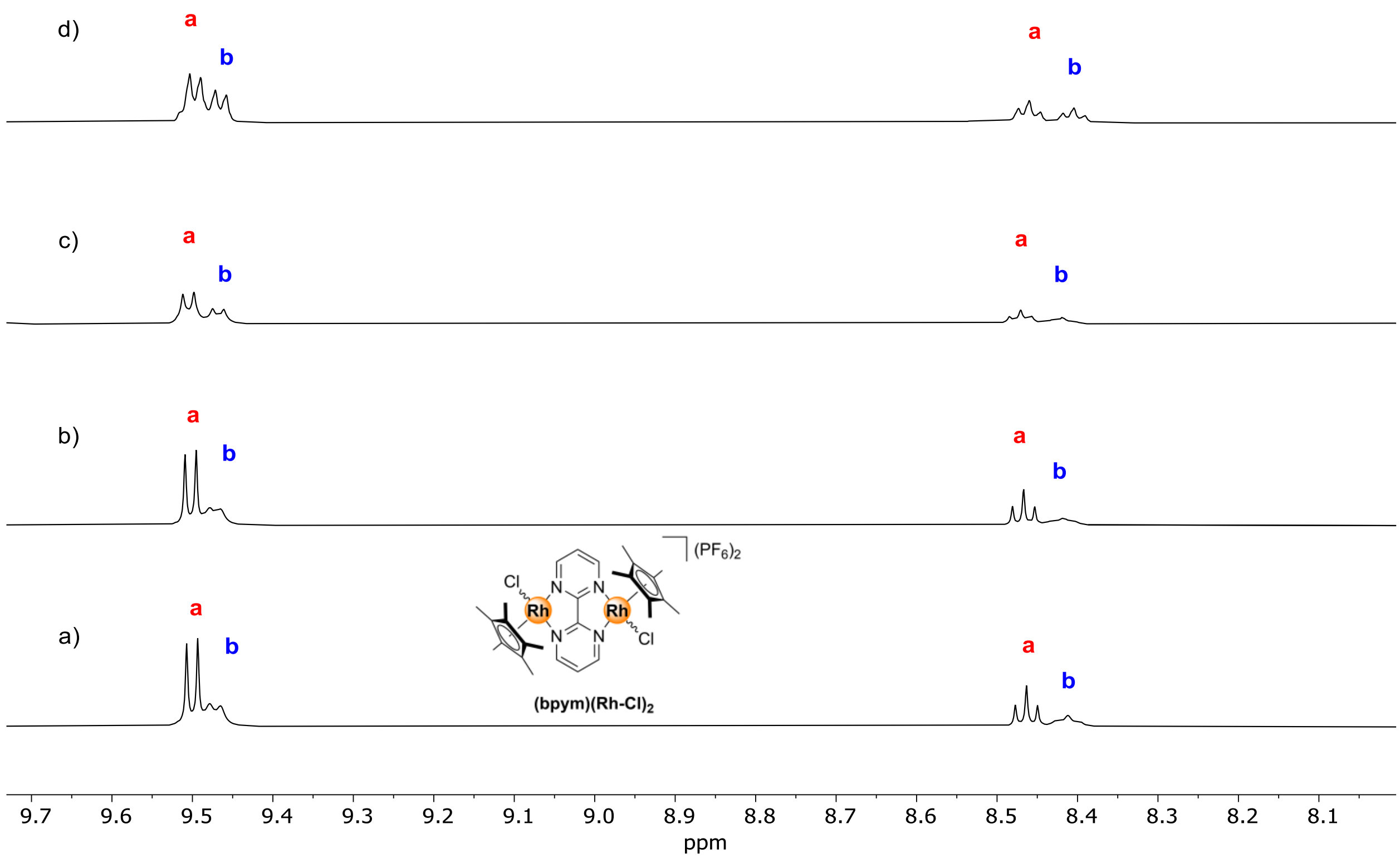

Figure S78. Variable temperature ^{1}H NMR spectra (400 MHz, CD_3NO_2) for **(bpym)(Rh-Cl)₂**. The signals from the two diastereomers are labeled **a** and **b**, respectively. (a) 298 K (**a**/**b** = 1.7); (b) 353 K (**a**/**b** = 1.7); (c) 383 K (**a**/**b** = 1.7); (d) 298 K after heating (**a**/**b** = 1.3)

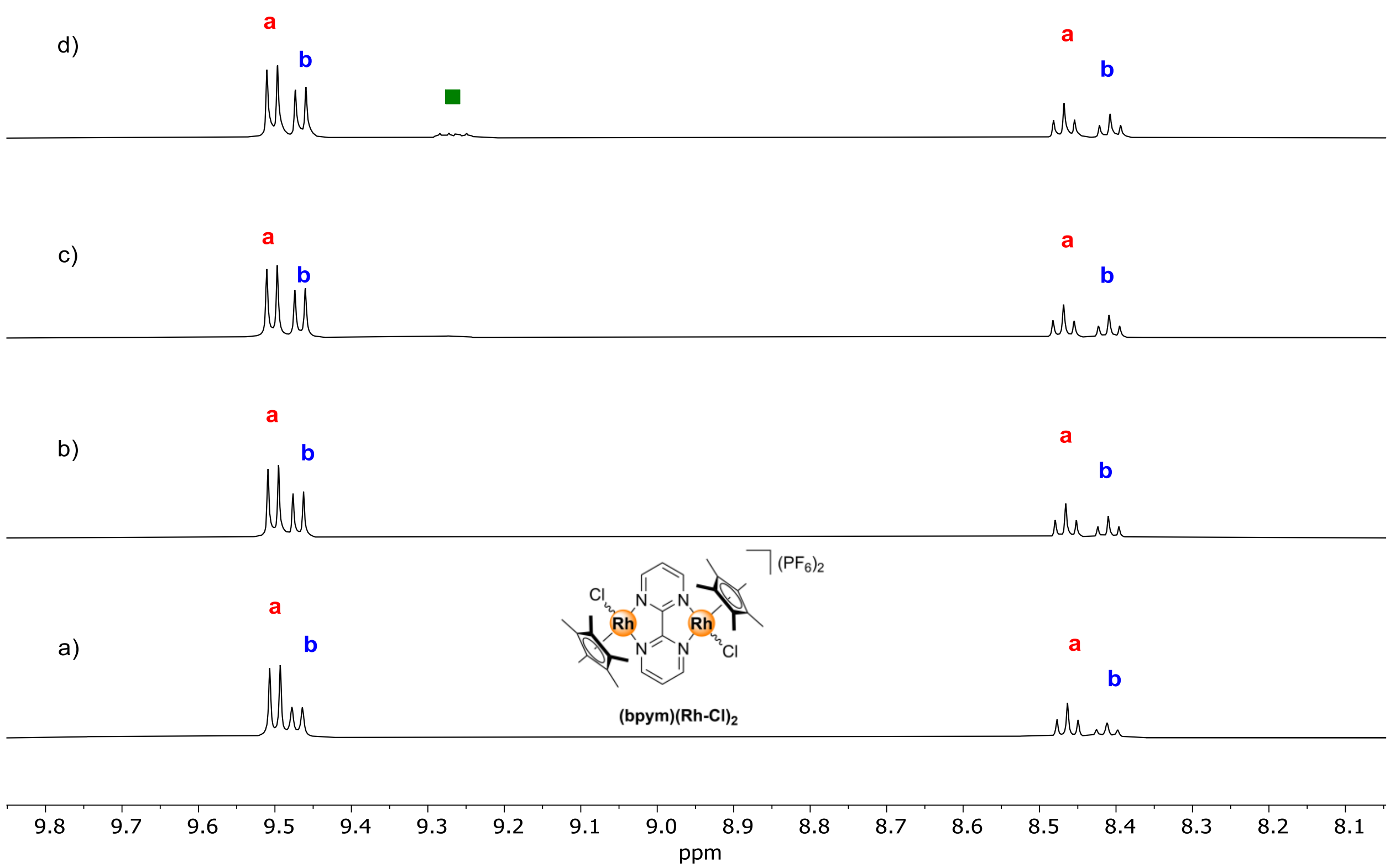

Figure S79. ^{1}H NMR spectra (400 MHz, CD_3NO_2) for **(bpym)(Rh-Cl)$_2$** after heating at 70 °C; the signals from the two diastereomers are labeled **a** and **b**, respectively. (a) prior to heating (**a**/**b** = 1.7); (b) 70 °C, 2 hours (**a**/**b** = 1.6); (c) 70 °C, 18 hours (**a**/**b** = 1.4); (d) 70 °C, 40 hours (**a**/**b** = 1.4). Peaks marked with green squares correspond to **(bpym)Rh-Cl**.

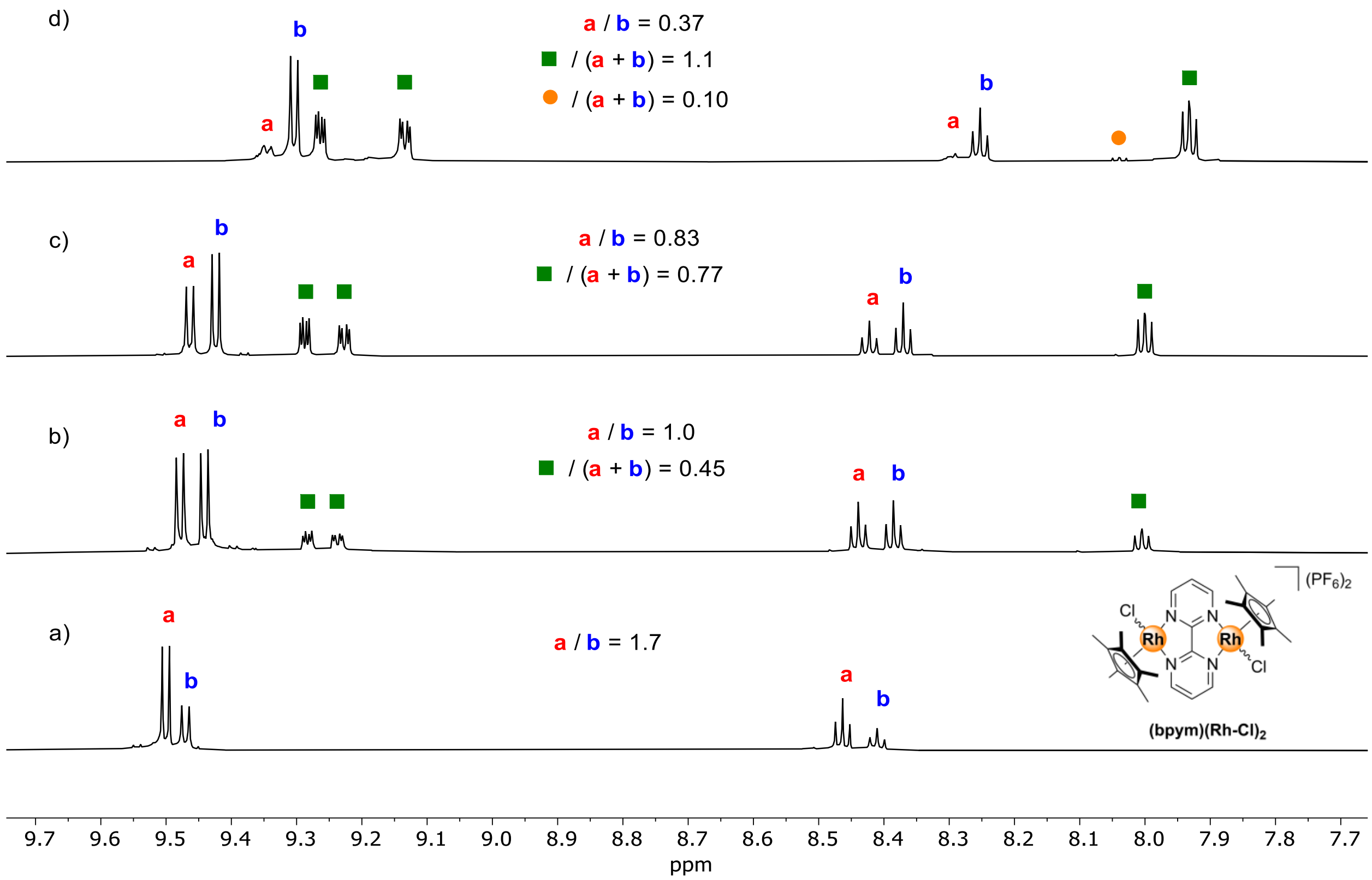

Figure S80. ^{1}H NMR spectra (500 MHz) for addition of CD_3CN to **(bpym)(Rh-Cl)₂**; (a) neat CD_3NO_2; (b) 5:1 CD_3NO_2/CD_3CN; (c) 4:1 CD_3NO_2/CD_3CN; (d) neat CD_3CN. Peaks marked as **a**/**b** correspond to the two diastereomers of **(bpym)(Rh-Cl)$_2$**; peaks marked with green squares correspond to **(bpym)Rh-Cl**; peaks marked with orange circles correspond to **(bpym)Rh-NCCH$_3$**. The listed ratios correspond to the ratio between **(bpym)(Rh-Cl)$_2$** diastereomers, the ratio between mono- and dirhodium chloride species, and the ratio of **(bpym)Rh-NCCH$_3$** vs. dirhodium species.

Electrochemistry

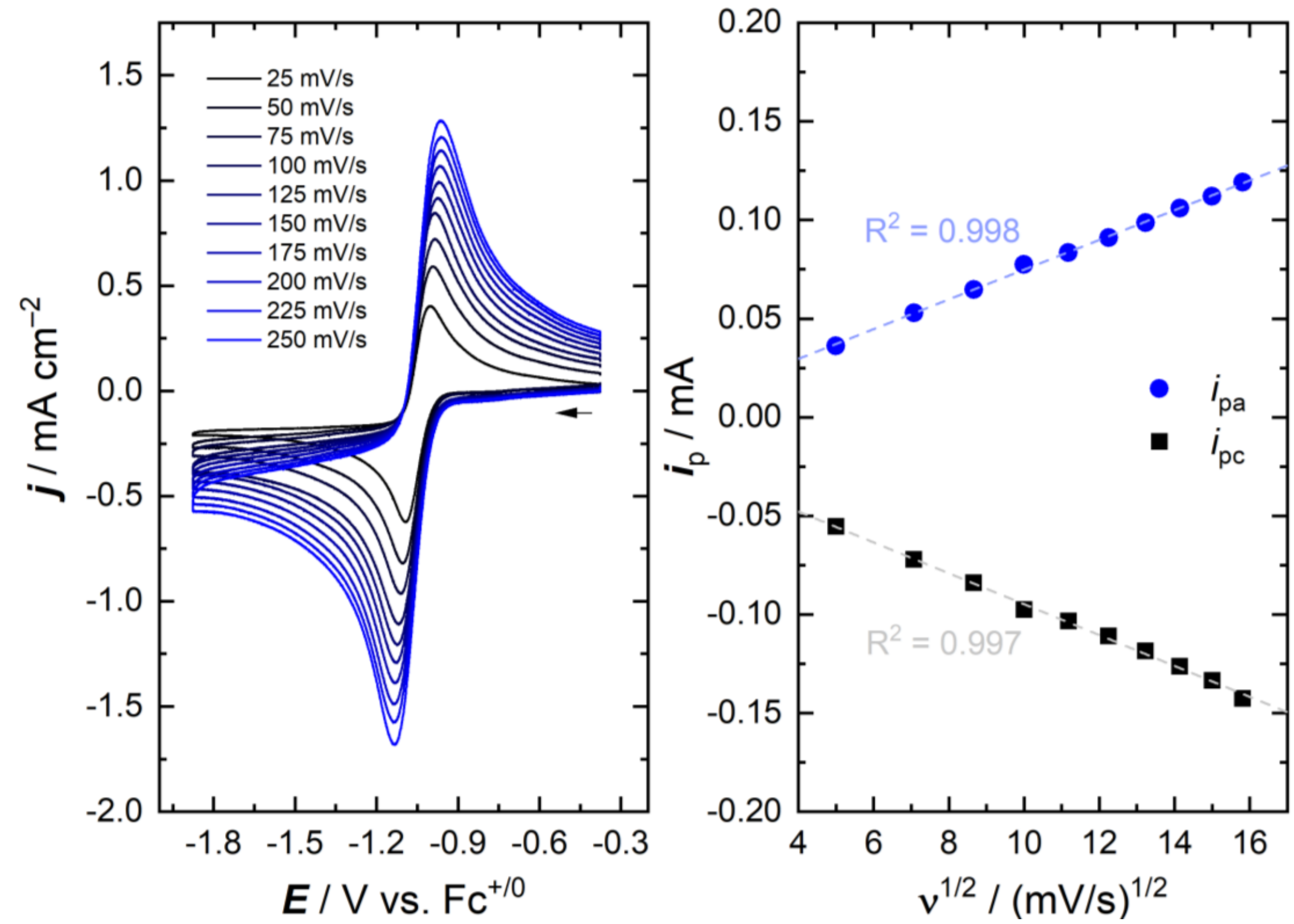

Figure S81. Left panel: CV data (0.1 *M* [$^{n}Bu_4N$][PF_6] in CH_3CN, [Rh] = 2 m*M*) for **(bppm)Rh-Cl** at varying scan rates. Right panel: plots of peak currents vs. the square root of the CV scan rate.

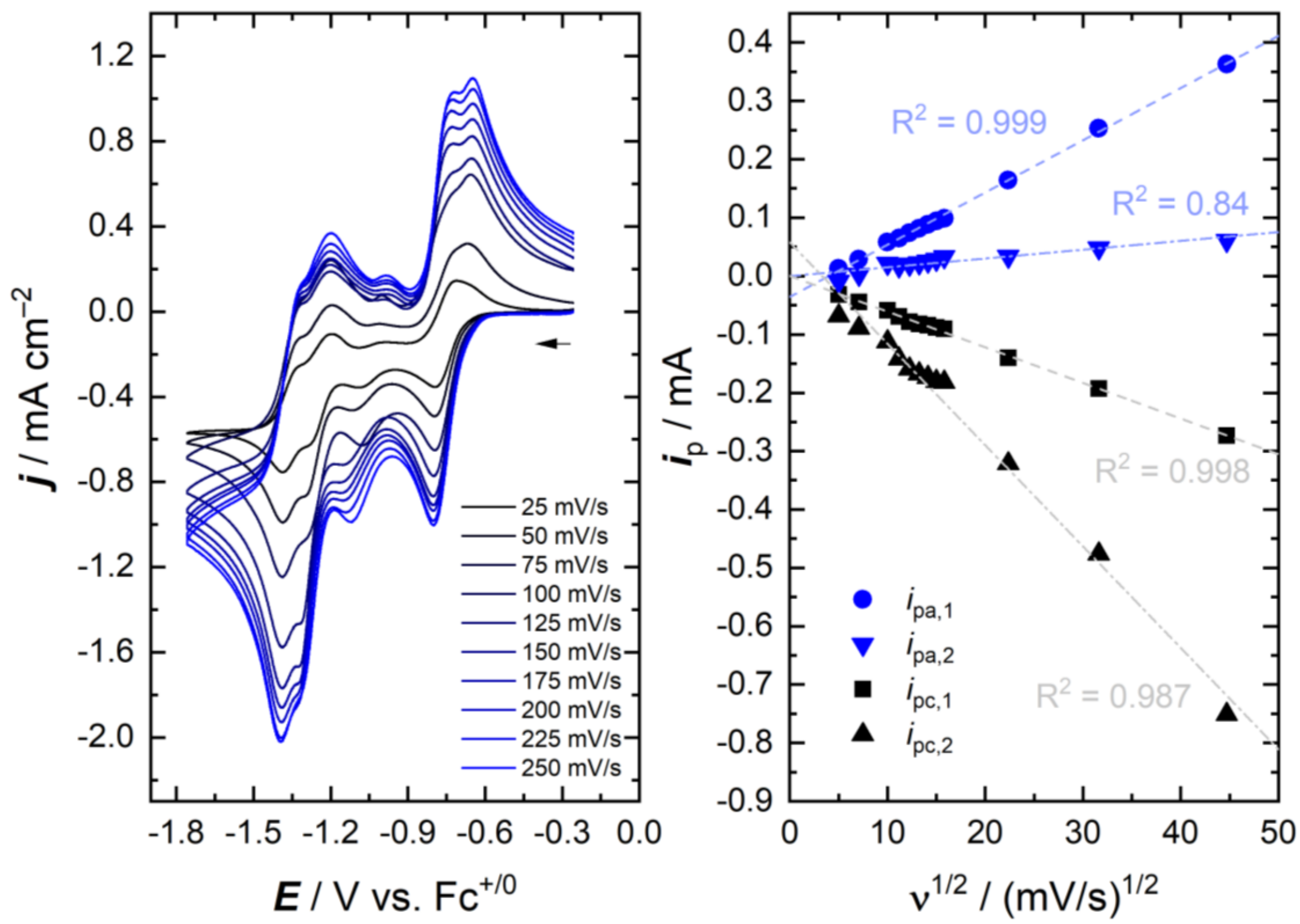

Figure S82. Left panel: CV data (0.1 *M* [^{n}Bu$_4$N][PF$_6$] in CH$_3$CN, [Rh complex] = 2 m*M*) for **(bppm)(Rh-Cl)$_2$** at varying scan rates. Right panel: plots of peak currents vs. the square root of the CV scan rate. The low linearity for the correlation between the anodic peak current for the second set of ET events and $\nu^{1/2}$ can be ascribed to the complex set of ET-induced chemical processes (dissociation of mono- and/or bidentate ligand). This correlation is however substantially more linear than that observed as a function of scan rate (ν; $R^2 = 0.64$).

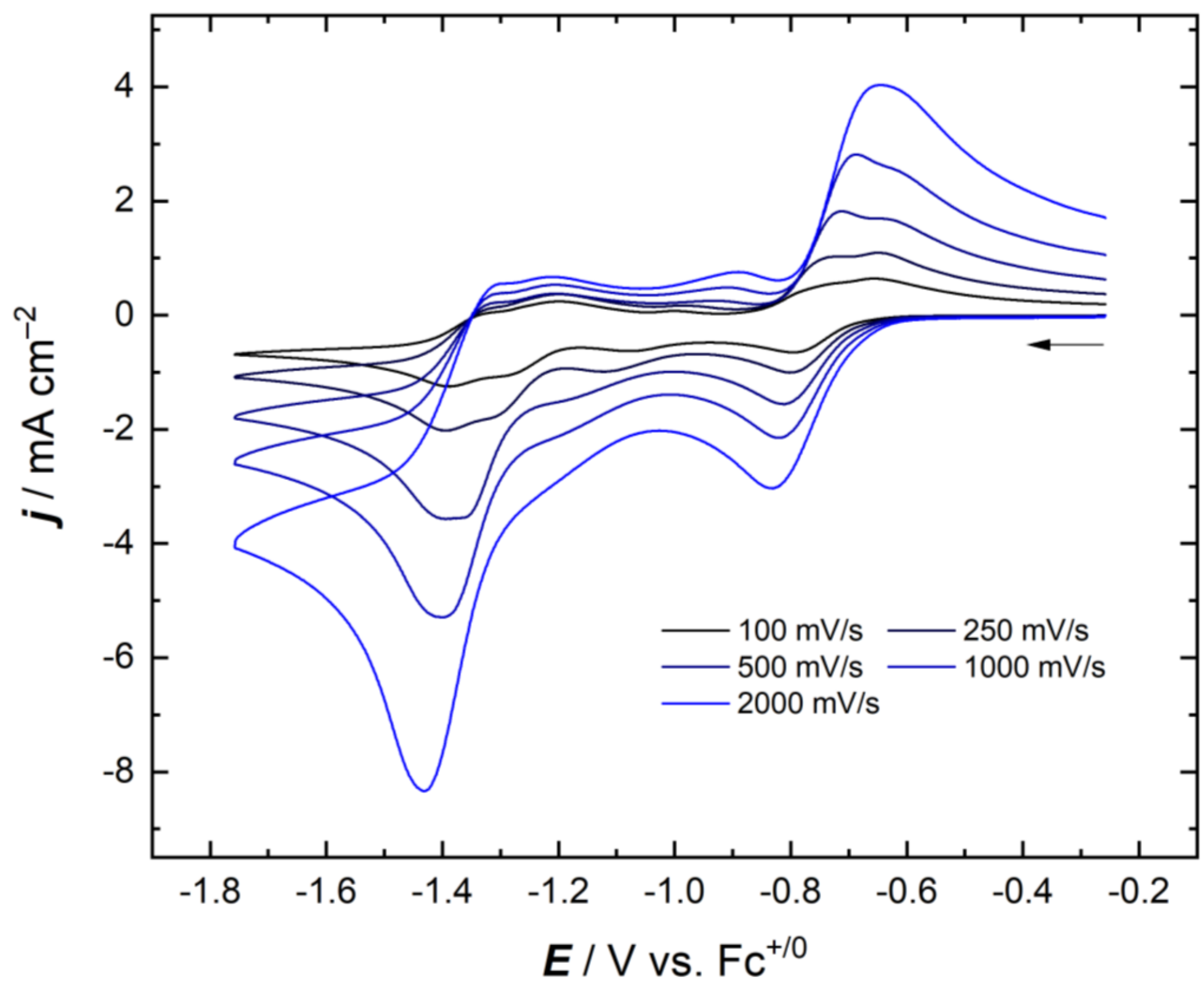

Figure S83. CV data (0.1 *M* [$^{n}Bu_4N$][PF_6] in CH_3CN, [Rh complex] = 2 m*M*) for **(bppm)(Rh-Cl)$_2$** at high scan rates.

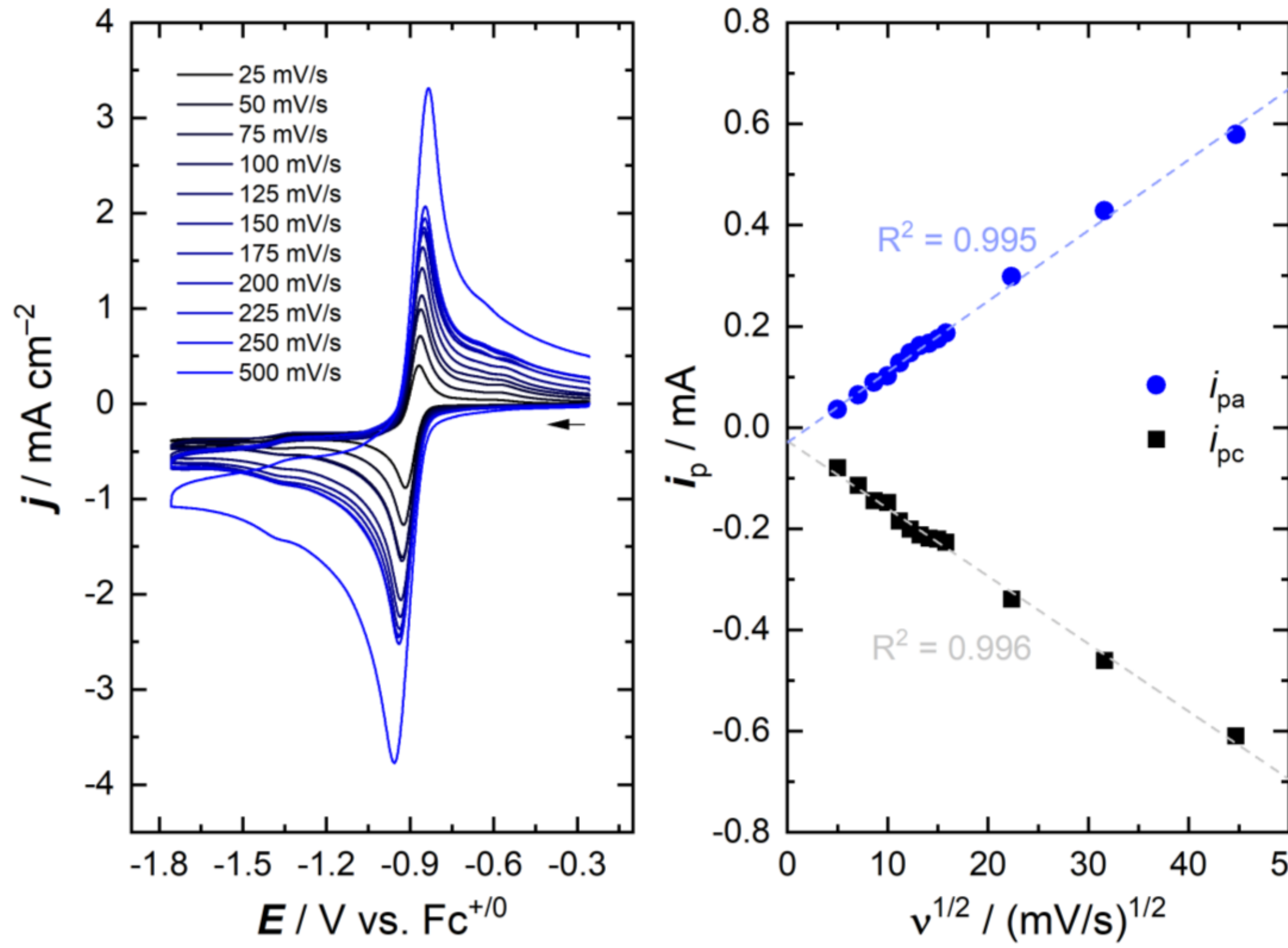

Figure S84. Left panel: CV data (0.1 *M* [^{n}Bu$_4$N][PF$_6$] in CH_3CN, [Rh] = 2 m*M*) for **(bppm)Rh-NCCH$_3$** at varying scan rates. Right panel: plots of peak currents vs. the square root of the CV scan rate.

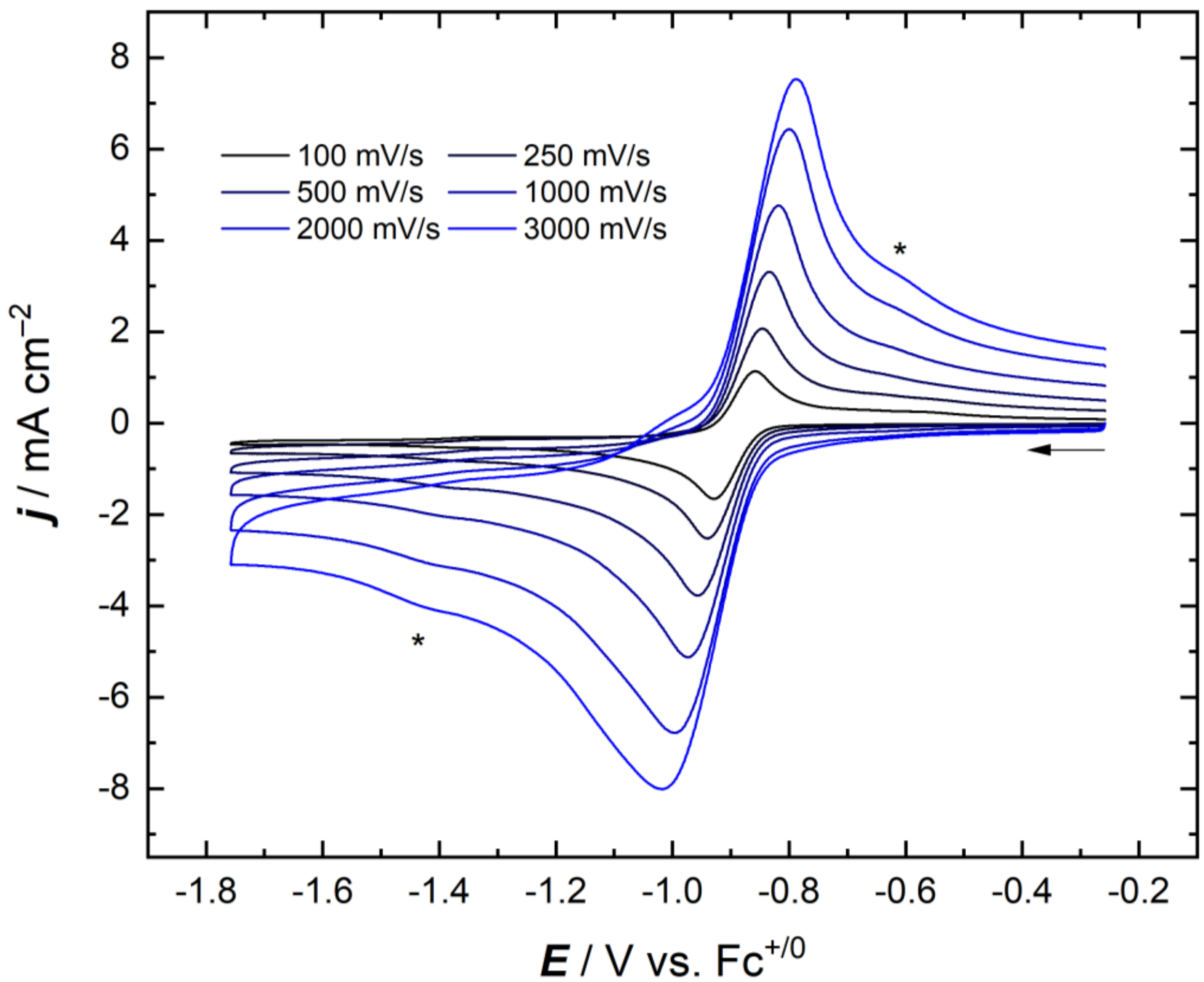

Figure S85. CV data (0.1 *M* [^{n}Bu$_4$N][PF$_6$] in CH_3CN, [Rh complex] = 2 m*M*) for **(bppm)Rh-NCCH$_3$** at high scan rates. The peaks marked with (*) correspond to events associated with the putative **(bppm)(Rh-NCCH$_3$)$_2$** byproduct, indicating that ET-induced reactivity is observed even at faster scan rates.

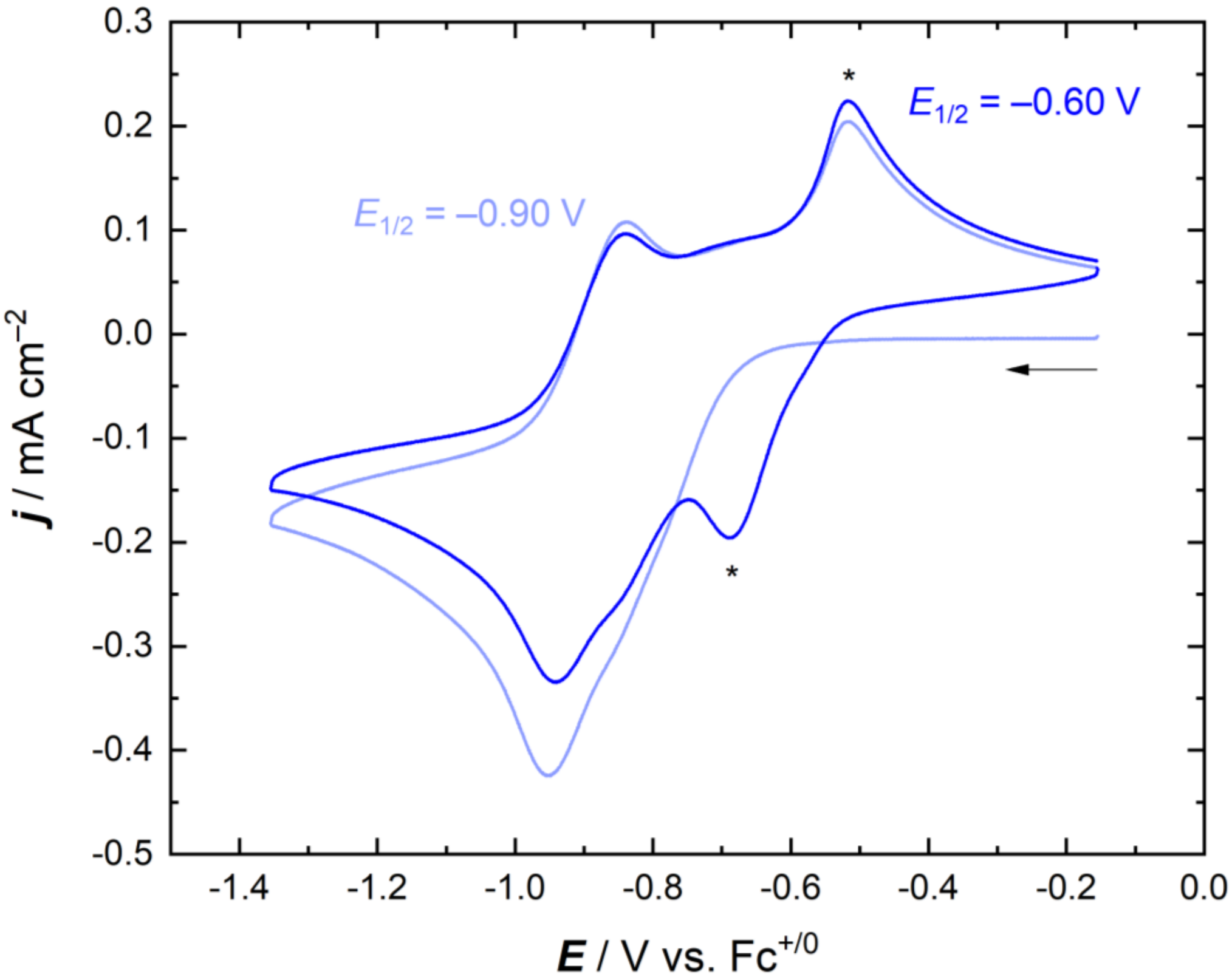

Figure S86. CV data (0.1 *M* [$^{n}Bu_4N$][PF_6] in 1,2-difluorobenzene (DFB), [Rh complex] = ~0.5 m*M*) for **(bppm)Rh-NCCH$_3$**. Peaks marked with (*) correspond to an electroactive byproduct generated following initial ET, possibly via reaction of a reduced Rh complex with DFB.

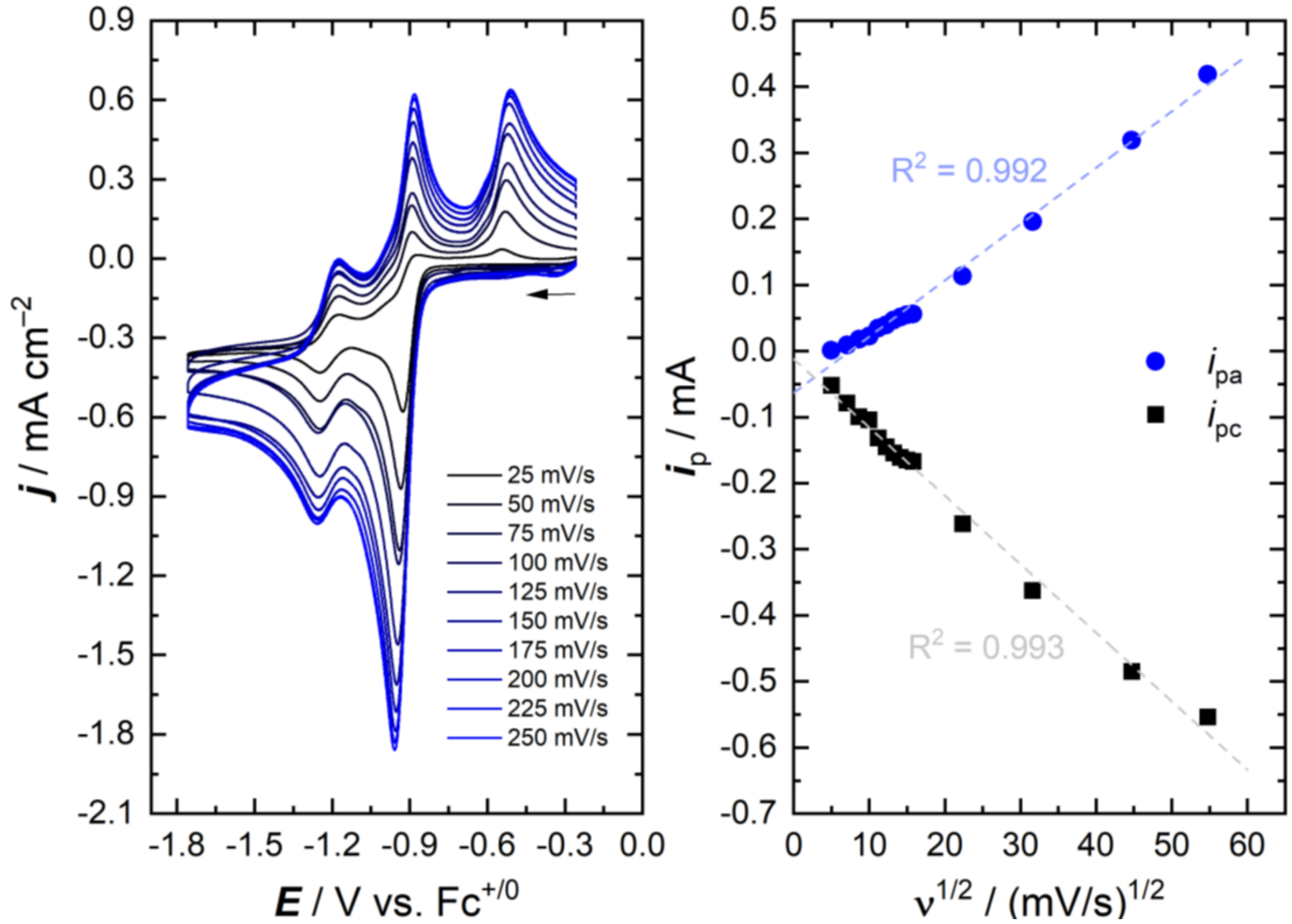

Figure S87. Left panel: CV data (0.1 *M* [^{n}Bu$_4$N][PF$_6$] in CH$_3$CN, [Rh] = 2 m*M*) for **(bpym)Rh-NCCH$_3$** at varying scan rates. Right panel: plots of peak currents vs. the square root of the CV scan rate.

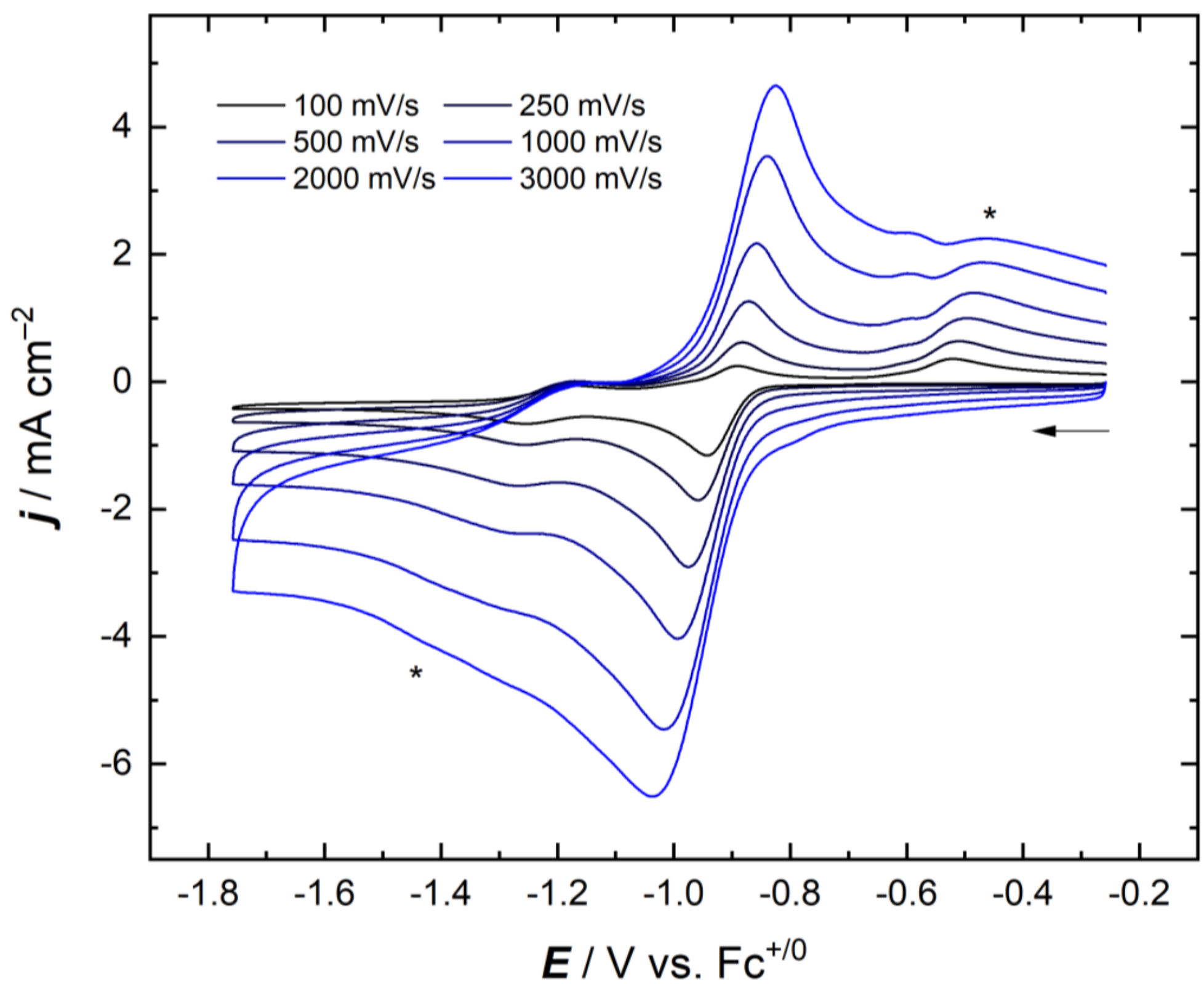

Figure S88. CV data (0.1 *M* [$^{n}Bu_4N$][PF_6] in CH_3CN, [Rh complex] = 2 m*M*) for **(bpym)Rh-Cl** at high scan rates. The peaks marked with (*) correspond to events associated with the putative **(bpym)(Rh-NCCH$_3$)$_2$** byproduct, indicating that ET-induced reactivity is observed even at faster scan rates.

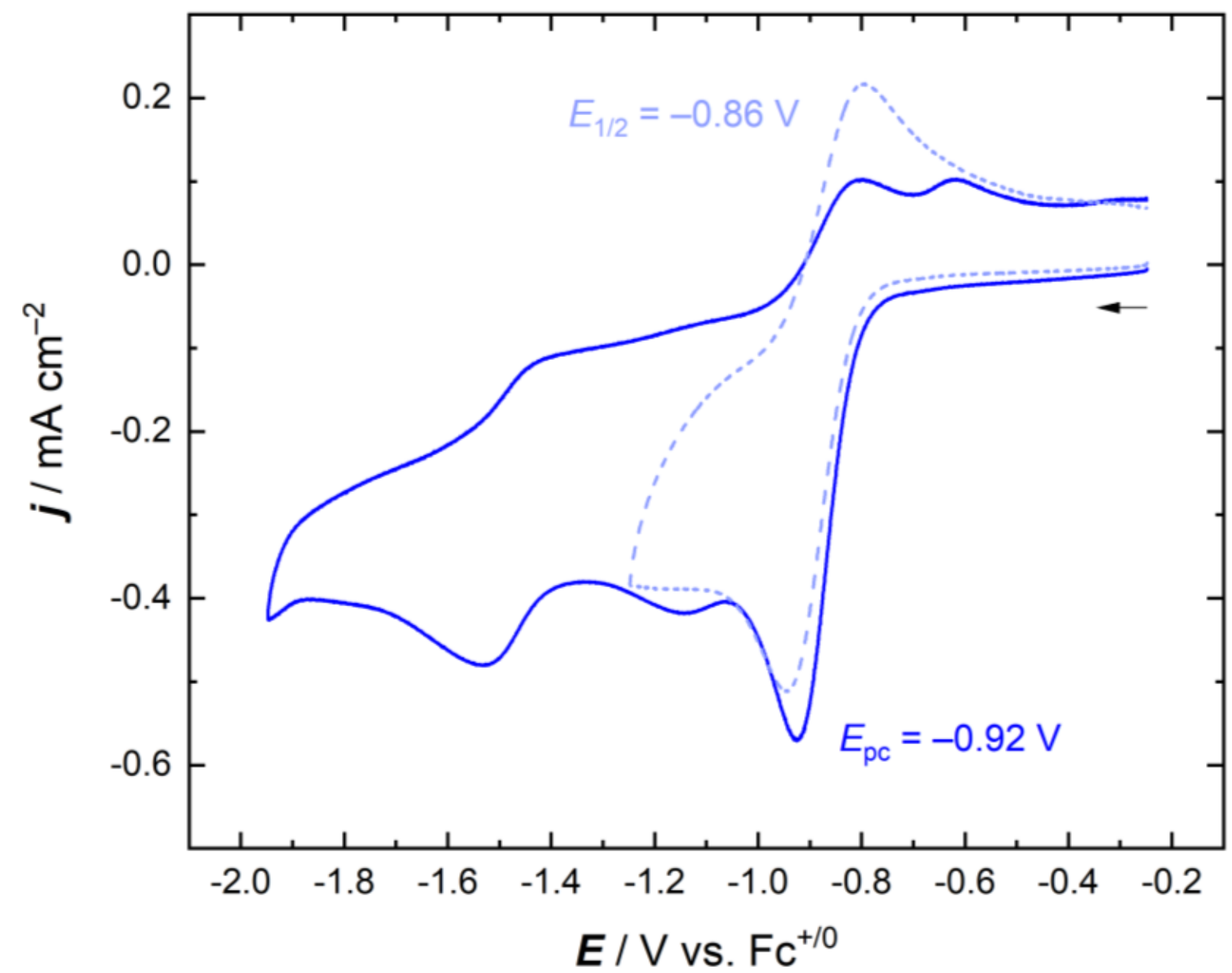

Figure S89. CV data (0.1 *M* [^{n}Bu$_4$N][PF$_6$] in CH_3CN, 100 mV/s, [Rh] = 2 m*M*) for [Cp*Rh(NCCH$_3$)$_3$](PF$_6$)$_2$. All scans starting at ~ –0.2 V vs Fc$^{+/0}$. Switching potential: –1.2 V (light blue dashed trace) or –1.95 V (dark blue solid trace).

X-ray crystallography

Table S2. Crystal and refinement data.

	**(bppm)Rh-Cl**	**(bppm)(Rh-Cl)$_2$**	**(bppm)Rh-NCCH$_3$**
CCDC number	2574181	2574175	2574180
empirical formula	$C_{24}H_{25}ClF_6N_4PRh$	$C_{38}H_{50}Cl_2F_{12}N_4OP_2Rh_2$	$C_{28}H_{31}F_{12}N_6P_2Rh$
formula wt	652.81	1145.48	844.44
T (K)	100(2)	100(2)	100(2)
a, Å	7.6587(7)	20.0350(16)	11.3640(10)
b, Å	11.4231(7)	14.4616(11)	11.8145(9)
c, Å	14.9692(10)	16.6848(13)	13.5682(11)
α, deg	75.805(3)	90	86.882(3)
β, deg	88.273(3)	116.383(2)	85.590(3)
γ, deg	87.799(2)	90	65.252(2)
V, Å^3	1268.38(16)	4330.7(6)	1649.0(2)
Z	2	4	2
cryst syst	triclinic	monoclinic	triclinic
space group	*P*-1	*C*2/c	*P*-1
ρ_{calcd}, g/cm^3	1.709	1.757	1.701
2θ range, deg	4.046 to 52.034	4.538 to 52.048	3.954 to 52.094
μ, mm^{-1}	0.908	1.049	0.714
abs corr	numerical	numerical	numerical
GOOF^c	1.108	1.072	1.045
R1,^{a} wR2^b (I > 2σ(I))	0.0196, 0.0490	0.0312, 0.0775	0.0272, 0.0647
Largest diff. peak/hole, e/Å^3	0.40/–0.27	0.53/–0.60	0.97/–0.57

^{a} $R1 = \Sigma||F_o|-|F_c||/\Sigma|F_o|$ ^{b} $wR2 = [\Sigma[w(F_o^2-F_c^2)^2]/\Sigma [w(F_o^2)^2]]^{1/2}$ ^{c} $GOOF = S = [\Sigma[w(F_o^2-F_c^2)^2]/(n-p)]^{1/2}$

Table S3. Crystal and refinement data (cont'd).

	**(bppm)Rh**	**(bpym)Rh-Cl**	**(bpym)Rh-Cl•CH_3CN**
CCDC number	2574177	2574174	2574179
empirical formula	$C_{24}H_{25}N_4Rh$	$C_{36}H_{42}Cl_2F_{12}N_8P_2Rh_2$	$C_{20}H_{24}ClF_6N_5PRh$
formula wt	472.39	1153.43	617.77
T (K)	100(2)	100(2)	100(2)
a, Å	16.8920(13)	23.259(3)	12.706(4)
b, Å	8.5138(6)	11.8163(13)	12.596(3)
c, Å	28.408(2)	7.5383(8)	15.319(4)
α, deg	90	90	90
β, deg	90	95.233(4)	102.824(10)
γ, deg	90	90	90
V, $Å^3$	4085.5(5)	2063.2(4)	2390.5(11)
Z	8	2	4
cryst syst	orthorhombic	monoclinic	monoclinic
space group	$Pna2_1$	$C2/m$	$P2_1/c$
ρ_{calcd}, g/cm^3	1.536	1.857	1.717
2θ range, deg	4.822 to 52.028	3.87 to 52.296	4.23 to 52.056
μ, mm^{-1}	0.854	1.102	0.959
abs corr	numerical	numerical	numerical
GOOF[c]	1.055	1.088	1.101
R1,[a] wR2[b]	0.0278,	0.0298,	0.0315,
(I > 2σ(I))	0.0651	0.0750	0.0765
Largest diff. peak/hole, e/$Å^3$	1.47/–0.68	0.44/–0.69	0.77/–0.52

[a] $R1 = \Sigma||F_o|-|F_c||/\Sigma|F_o|$ [b] $wR2 = [\Sigma[w(F_o^2-F_c^2)^2]/\Sigma [w(F_o^2)^2]]^{1/2}$ [c] $GOOF = S = [\Sigma[w(F_o^2-F_c^2)^2]/(n-p)]^{1/2}$

Table S4. Crystal and refinement data (cont'd).

	**(bpym)Rh-NCCH$_3$**	**(bpym)(Rh-Cl)$_2$**
CCDC number	2574178	2574176
empirical formula	$C_{24}H_{30}F_{12}N_7P_2Rh$	$C_{34}H_{49}Cl_2F_{12}N_5OP_2Rh_2$
formula wt	809.40	1110.44
T (K)	100(2)	100(2)
a, Å	9.3020(19)	16.850(3)
b, Å	12.336(3)	15.570(3)
c, Å	15.865(3)	17.384(3)
α, deg	110.281(7)	90
β, deg	94.163(7)	107.614(6)
γ, deg	104.361(7)	90
V, Å^3	1628.8(6)	4347.1(14)
Z	2	4
cryst syst	triclinic	monoclinic
space group	P-1	C2/m
ρ_{calcd}, g/cm^3	1.650	1.697
2θ range, deg	3.68 to 52.096	1.971 to 26.024
μ, mm^{-1}	0.720	1.042
abs corr	numerical	numerical
GOOF^c	1.061	1.143
R1,^{a} wR2^b	0.0384,	0.0415,
(I > 2σ(I))	0.1010	0.0951
Largest diff. peak/hole, e/Å^3	0.76/–0.53	0.73/–0.86

^{a} $R1 = \Sigma||F_o|-|F_c||/\Sigma|F_o|$ ^{b} $wR2 = [\Sigma[w(F_o^2-F_c^2)^2]/\Sigma [w(F_o^2)^2]]^{1/2}$ ^{c} $GOOF = S = [\Sigma[w(F_o^2-F_c^2)^2]/(n-p)]^{1/2}$

Table S5. Comparison of structural metrics for **(bppm)Rh-Cl** with additional monorhodium complexes of polyheterocyclic complexes (Chart S2).

	**(bppm)Rh-Cl**	**[CpRh(bpy)Br]$^+$**	**[CpRh(bpy)Br]$^+$**	**(bppd)Rh-Cl**	**(bppz)Rh-Cl**[a]		
CCDC #	2574181	2070587	1533255	739570	1414747	734453	604770[g]
				bond lengths (Å)			
Rh–N1	2.1164(14)	2.080(3)	2.084(4)	2.085(3)	2.098(3)	2.091(5)	2.141(15), 2.049(16)
Rh–N2	2.1167(14)	2.071(3)	2.077(4)	2.120(3)	2.092(3)	2.103(5)	2.083(16), 2.105(15)
Rh–Cl	2.4050(4)	2.4976(4)	2.6806(5)	2.3833(12)	2.3896(11)	2.403(5)	2.403(5), 2.379(5)
Rh–Cp(*)[b]	1.7802(7)	1.7839(15)	1.790(3)	1.790(1)	1.7891(15)	1.791(3)	1.759(9), 1.817(9)
$d_{C–C}$(Rh)[c]	1.475(2)	1.170(4)	1.471(7)	1.471(5)	1.485(5)	1.477(9)	1.47(3), 1.52(3)
$d_{C–C}$(non-Rh)[d]	1.480(2)	-	-	1.495(5)	1.486(5)	1.486(7)	1.43(3), 1.54(3)
				bond angles (°)			
N–Rh–N	76.46(5)	76.69(10)	77.61(15)	75.95(11)	75.75(13)	75.61(19)	74.9(6), 77.1(7)
∠(Cp*-[RhN_2])[e]	57.59(6)	56.12(12)	56.9(2)	58.21(14)	62.30(12)	61.9(2)	61.9(6), 62.5(7)
Reference	this work	[10]	[11]	[12]	[13]	[14]	[15]

[a] metrics for three different polymorphs; [b] distance between Rh and the calculated centroid of the Cp(*) ligand; [c] length of interring C–C bond between Rh-bound heterocycles; [d] length of interring bond between central heterocycle and non Rh-bound pyridyl fragment (absent in ^{H}bpy complexes); [e] angle between the plane of the Cp* ligand and the calculated mean plane for the 5-membered Rh metallocycle; [f] values for two independent molecules in the asymmetric unit cell shown.

Additional analysis of structural data: distortions in polyheterocyclic ligands

Chart S1. Interplane angles in polyheterocyclic ligand frameworks.

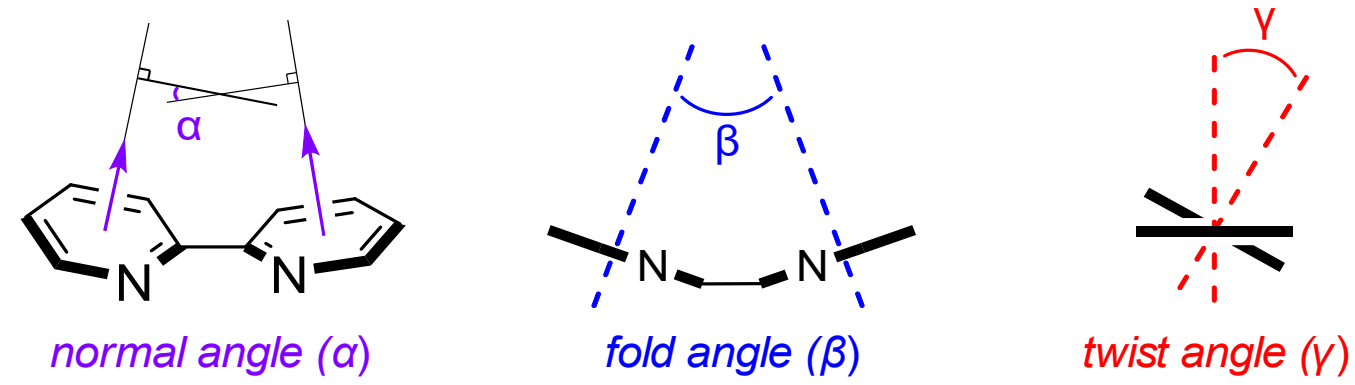

Chart S2. Mono- and dirhodium complexes for structural analysis.

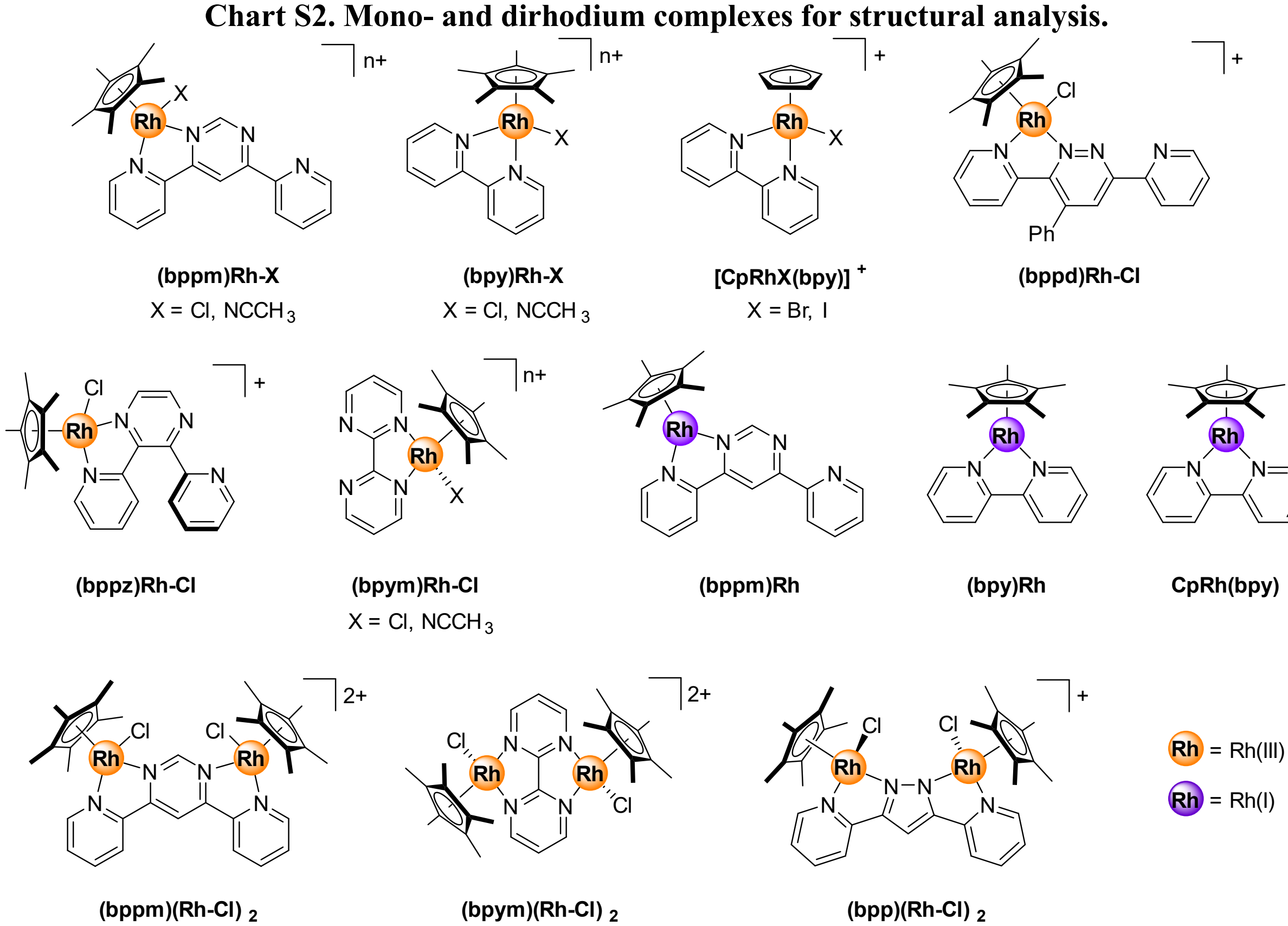

While the bond and angle metrics at the Rh site(s) in complexes of the bppm ligand are not significantly different from those in complexes supported by other mono- or dinucleating polyheterocyclic ligands, intriguing trends can be discerned in the extent of deformation of these aromatic frameworks. Three interplane angles can be defined for the heterocycles in polyheterocyclic platforms, which are visualized in Chart S1: the normal angle (α, defined as the angle between the vectors normal to the planes of the two heterocycles), the fold angle (β, the extent to which the ligand platform is bent along the interring axis) and the twist angle (γ, the dihedral angle between the two heterocycle planes). The latter two angles can be viewed as distinct components of the normal angle α. A survey of structures for complexes of the 2,2'-bipyridine (bpy) ligand analogous to **(bppm)Rh-Cl**, containing various Cp-type platforms and different monodentate ligands and outer-sphere anions, reveals that the bpy platforms in such compounds are typically not twisted ($\gamma \approx 0$), though they can be slightly folded ($\beta \sim$ 1-4°). Given the negligible twist angles, the normal angles are therefore usually indistinguishable from the fold angles in these systems (i.e., $\alpha \approx \beta$ when $\gamma \approx 0$). The **[CpRh(bpy)I]**$^+$ complex (Chart S2) represents an exception to this trend, as it is the only bpy complex of this type that displays a substantial twist ($\gamma \sim 10°$) on top of a typical fold angle ($\beta \sim 2°$), resulting in a larger normal angle than most such complexes.[11] Complex **(bppm)Rh-Cl** resembles this

exception more than the average bpy complex: the two Rh-bound heterocycles display a larger normal angle (α = 12.37(6)°) with both substantial fold (β = 8.60(6)°) and twist (γ = 8.93(6)°) components. Notably, **$[CpRh(bpy)I]^{+}$** and **(bppm)Rh-Cl** are the only two complexes in this group that display possible π-π interactions between heterocycle motifs on adjacent molecules in their solid-state structures (Table S8).[16] In **(bppm)Rh-Cl**, these interactions involve the central pyrimidine and the 'wingtip' pyridyl groups that are not bound to Rh (interplane angle: 3.12(6)°; centroid–centroid distance = 3.8645(10) Å; plane shift distances = 1.881(2), 1.735(3) Å), while in **$[CpRh(bpy)I]^{+}$** they involve the equivalent pyridyl group on adjacent complexes (interplane angles: 0.0(6)° and 0.0(7)°; centroid–centroid distances = 3.740(5) and 3.870(4) Å; plane shift distances = 1.577(9) and 1.808(10) Å). Based on these observations, it appears that the packing effects resulting from these π-π interactions are sufficient to deform the polypyridyl platforms, resulting in the greater twist angles (and overall α angles) measured in complexes for which such interactions are present.

Table S6. Ligand structural metrics for the complexes investigated in the present study.

Complex	CCDC#	normal angle (α)[a], °	fold angle (β)[a], °	twist angle (γ)[a], °	Ref.
**(bppm)Rh-Cl**	2574181	12.37(6)	8.60(6)	8.93(6)	this work
**(bppm)Rh-$NCCH_3$**	2574180	8.16(7)	6.94(7)	4.29(7)	this work
**(bpy)Rh-Cl**[b]	1420047	1.2(2)	1.2(2)	0.0(5)	[17]
	675527	2.5(5)	2.5(5)	0.0(5)	[18]
	1303106	1.4(3)	1.4(3)	0.0(11)	[19]
**(bpy)Rh-$NCCH_3$**	995532	3.06(8), 0.52(11)	2.80(8), 30(19)	1.24(8), 0.17(11)	[20]
**$[CpRh(bpy)Br]^{+}$**	2070587	4.13(12)	4.13(12)	0.03(11)	[10]
**$[CpRh(bpy)I]^{+}$**	1533255	10.01(19)	2.12(19)	9.8(2)	[11]
**(bppd)Rh-Cl**	739570	6.88(13)	6.62(13)	1.89(12)	[12]
**(bppz)Rh-Cl**[c]	1414747	18.87(11)	15.98(11)	10.18(11)	[13]
	734453	18.85(19)	15.91(18)	10.26(18)	[14]
	604770[d]	19.1(6), 19.8(6)	15.4(6), 17.6(6)	11.5(6), 9.2(6)	[15]
**(bppm)$(Rh\text{-}Cl)_2$**	2574175	10.09(10)	10.07(10)	0.57(7)	this work
**(bpym)$(Rh\text{-}Cl)_2$**	635001	17.7(3)	17.7(3)	0.0(6)	[8]
	2574176	16.4(2)	16.4(2)	0.0(7)	this work
**(bpp)$(Rh\text{-}Cl)_2$**	1401649	14.0(4), 17.1(4)	2.1(4), 8.6(4)	13.8(4), 14.8(4)	[21]
**(bppm)Rh**	2574177[d]	2.56(17), 2.69(17)	0.88(16), 0.52(16)	2.41(16), 2.63(17)	this work
**(bpy)Rh**	959935	2.54(11)	2.37(11)	0.92(11)	[22]
	995550	6.21(9)	5.94(9)	1.80(9)	[20]
**CpRh(bpy)**	1532782	2.62(6)	1.26(6)	2.29(6)	[11]
**(bpym)Rh-Cl•CH_3CN**	2574179	4.69(8)	4.56(9)	1.09(7)	this work
**(bpym)Rh-Cl**	2574174	4.15(12)	4.15(12)	0.0(2)	this work
**(bpym)Rh-$NCCH_3$**	2574178	3.09(10)	1.00(11)	2.92(10)	this work

[a] as defined in Chart S1; [b] metrics for three complexes containing different counteranions (PF_6^-, ^-OTf, and ClO_4^-, respectively); [c] metrics for three different polymorphs; [d] values for two independent molecules in the asymmetric unit cell shown.

Exchange of Cl^- for a $NCCH_3$ solvent molecule has minimal effects on the structural metrics at Rh; as a result, the relative extents of deformation of the polyheterocyclic frameworks remain the same between pairs of related complexes (**(bppm)Rh-Cl** vs. **(bppm)Rh-$NCCH_3$** and **(bpy)Rh-Cl** vs. **(bpy)Rh-$NCCH_3$**). Specifically, the bidentate binding site is more distorted in the bppm framework than in bpy, though the normal, fold, and twist angles are generally smaller in **(bppm)Rh-$NCCH_3$** than in **(bppm)Rh-Cl**. Similarly to what was observed for the chloride analogues, the magnitude of these distortions appears correlated with π-π interactions–while similar π-π interactions can be identified in **(bppm)Rh-$NCCH_3$** as for **(bppm)Rh-Cl**, no analogous interactions are observed in **(bpy)Rh-$NCCH_3$** (as seen in structures of the corresponding chloride complex).

Extension of these analyses to other $[Rh_1]$ complexes of polypyridyl frameworks with multiple binding sites reveals analogous trends. The monorhodium complex **(bppd)Rh-Cl** (Chart S2) displays a similar fold

angle to complex **(bppm)Rh-Cl** (β = 6.62(13)°) but a substantially smaller twist angle (1.89(2)°), more akin to complexes of the mononucleating bpy ligand. [12] Possible π-π interactions are observed in this complex, though not between two pyridyl groups–rather, between a pyridyl group and the Ph substituent on the pyrazine ring of an adjacent molecule; consequently, the metrics for these interactions are somewhat different than in **(bppm)Rh-Cl** and **[CpRh(bpy)I]$^+$**, especially with respect to the interplane angles, which are substantially larger in this case (10.67(14)°). Conversely, the *ortho* arrangement of the two pyridyl groups in the bppz ligand results in substantial distortions of this platform in the structures of its Rh complex (**(bppz)Rh-Cl**), which displays both large fold (β > 15°) and twist (γ > 9°) angles.[13-15] These observations underscore the steric constraints imposed by this ligand platform, particularly relevant to the reactivity of [Rh_2] complexes. While possible π-π interactions can be identified between pyridyl groups on adjacent fragments in these complexes, these have generally larger centroid-centroid distances (> 4.2 Å) and plane shifts (> 2.5 Å), suggesting weaker interactions in these cases, likely due to the more sterically demanding framework (Table S8).

Slightly different distortions are observed in the two structures for **(bpym)Rh-Cl** (with or without a co-crystallized CH_3CN molecule): in the structure of the solvated complex, the bipyrimidine framework has a negligible twist angle (γ 0.0(5)°), and a fold angle of ~4°, while in the structure containing no co-crystallized solvents the bpym ligand is both slightly twisted (γ = 1.09(7)°) as well as folded (β = 4.56(11)°). Exchange of the chloride ligand for CH_3CN appears to reverse the relative magnitude of these distortions, with **(bpym)Rh-NCCH$_3$** displaying a larger twist (2.92(10)°) than fold (1.00(11)°) angle. Possible π-π interactions are observed in all three complexes (Table S8); these interactions involve both heterocycles in the bpym framework in **(bpym)Rh-Cl•CH$_3$CN** and **(bpym)Rh-NCCH$_3$** (notably, both structures contain a co-crystallyzed CH_3CN molecule), while only one heterocycle appears to engage in these interactions in **(bpym)Rh-Cl**.

Reduction of Rh and loss of the $Cl^–$ ligand reduce the deformation of the bppm framework in **(bppm)Rh** vs. **(bppm)Rh-Cl**, with the reduced complex displaying smaller fold (~2.5°) and twist angles (< 1°) than its Rh(III) precursor. This observation is consistent with the change in geometry in the Rh coordination sphere relieving some of the constraints imposed on the bppm framework, resulting in an overall more planar scaffold. Intriguingly, while no π-π interactions are observed between heterocycles in this structure, one of the wingtip pyridyl groups on each complex is within interaction range of the Cp* ligand on a symmetry-related Rh center (Table S8). Conversely, **(bpy)Rh** displays slightly *larger* twist angles (γ ~ 2°) than its Rh(III) analogue **(bpy)Rh-Cl**, for which γ values are generally negligible. Since it may be expected that the loss of the monodentate ligands upon reduction to Rh(I) should result, as in the case of **(bppm)Rh**, in fewer pressures on the bpy framework in these complexes, the observed deformations may simply be due to packing effects. In fact, possible π-π interactions can be identified in each of these complexes–though, intriguingly, these involve the Cp*ligands on adjacent molecules rather than the bpy heterocycles (Table S8). As before, the corresponding cyclopentadienyl complexes represent exceptions to these trends–while the bpy scaffold was substantially twisted in **[CpRh(bpy)I]$^+$**, this strain is mostly relieved upon reduction to Rh(I). Still, **CpRh(bpy)** also retains a small twist angle (2.29(6)°), and in fact is the only complex in this family to display possible π-π interactions involving both of the pyridyl group on its bpy ligand.

The addition of a second [Cp*RhCl] fragment has a significant effect on the geometry of the bppm framework–while the normal angle α is similar for **(bppm)Rh-Cl** and **(bppm)(Rh-Cl)$_2$** (~12° and 10°, respectively), the bppm scaffold in **(bppm)(Rh-Cl)$_2$** has a negligible twist, so it is essentially only folded, whereas both β and γ angles were considerable in **(bppm)Rh-Cl**. The changes in the polyheterocyclic scaffolds are accompanied by a greater deformation of the Rh metallocycles, as indicated by the metallocycle RMSD values (larger in **(bppm)(Rh-Cl)$_2$**, see below). As in its monorhodium analogue, **(bppm)(Rh-Cl)$_2$** displays possible π-π interactions between wingtip pyridyl groups on adjacent complexes (Table S8). Two literature dirhodium complexes supported by similar frameworks have been structurally characterized, **(bpym)(Rh-Cl)$_2$** and **(bpp)(Rh-Cl)$_2$** (Chart S2); unfortunately, however, neither is an ideal point of comparison for **(bppm)(Rh-Cl)$_2$** due to the nature of the specific ligands, whose steric demands drive more extensive deformations. Both bpym and bpp complexes have substantially more distorted

bidentate platforms: in **(bpym)(Rh-Cl)$_2$**, the *syn* arrangement of the Cp* ligands causes the pyrimidine groups to deflect towards the opposite side of the scaffold, resulting in a large fold angle (β = 16.4(2)°), even though the corresponding twist is negligible ($\gamma \approx 0°$, presumably due to the identical nature of the {Cp*RhCl} fragment on either side of the scaffold groups). In **(bpp)(Rh-Cl)$_2$**, on the other hand, the ligand platform is both folded and twisted as a result of the nature of the smaller pyrazole central heterocycle.

Taken as a whole, these comparisons reveal that binding of Rh to polypyridyl ligands can result in substantial deformation of these conjugated frameworks. Several modes for distortion were analyzed, including different ways for the ligand platform to deviate from planarity (fold and twist angles) and the deformation of the 5-membered metallocycle. While the heterocycles in these ligands are usually close to coplanar (with the exception of compounds where substantial steric hindrances force more significant deviations from planarity), small deformations are quite ubiquitous, and deformations beyond 10° from planarity can be observed for specific frameworks, usually concurrently with intermolecular π-π interactions involving one or more heterocycles in these platforms.

Table S7. Structural metrics relevant to π-π interactions for the complexes investigated in the present study.

Complex	Fragment A[a]	Fragment B[a]	Interplane angle	Centroid-centroid distance	Plane shift distance	Ref.
**(bppm)Rh-Cl**	wingtip py	central pym	3.12(6)	3.8645(10)	1.881(2), 1.735(3)	this work
**(bppm)Rh-NCCH$_3$**	wingtip py	central pym	8.16(7)	3.8270(12)	1.694(3), 1.585(3)	this work
**(bpy)Rh-Cl**			*no evident π-π interactions identified*			17<br>18<br>19
**(bpy)Rh-NCCH$_3$**			*no evident π-π interactions identified*			20
**[CpRh(bpy)Br]$^+$**			*no evident π-π interactions identified*			10
**[CpRh(bpy)I]$^+$**	py	py	0.0(6)	3.740(5)	1.577(9)	11
**(bppd)Rh-Cl**	wingtip py	pendant Ph	10.67(14)	3.913	1.581(6), 2.216(6)	12
**(bppz)Rh-Cl**	wingtip py	wingtip py	0.0(3)	4.285(3)	2.586(5)	13
	wingtip py	wingtip py	0.0(4)	4.299(4)	2.592(9)	14
	wingtip py	wingtip py	0.4(5)	4.277(10)	2.58(2), 2.60(3)	15
**(bppm)(Rh-Cl)$_2$**	wingtip py	wingtip py	0.00(2)	3.855(2)	1.760(4)	this work
**(bpym)(Rh-Cl)$_2$**	pym	pym	0	3.949(6)	1.933(12)	8
	pym	pym	0.00(17)	3.955(5)	1.833(10)	this work
**(bpp)(Rh-Cl)$_2$**	wingtip py	wingtip py	0(2)	3.701(8)	1.533(17)	21
	wingtip py	wingtip py	0.0(4)	4.614(9)	2.799(19)	
**(bppm)Rh**	wingtip py	Cp*	3.1(2)	3.631(3)	0.489(9), 0.553(11)	this work
	wingtip py	Cp*	4.8(2)	3.677(3)	0.546(11), 0.568(9)	
**(bpy)Rh**	Cp*	Cp*	0.0(6)	3.619(3)	0.308(7)	22
	Cp*	Cp*	0.0(2)	3.633(2)	0.335(6)	20
**CpRh(bpy)**	py	py	2.62(6)	3.564(4)	1.064(7), 0.996(7)	11
**(bpym)Rh-Cl•CH$_3$CN**	pym	pym	4.19(8)	4.0931(15)	2.089(4), 2.308(4)	this work
**(bpym)Rh-Cl**	pym	pym	4.15(12)	3.6535(18)	3.452(2), 3.452(2)	this work
**(bpym)Rh-NCCH$_3$**	pym	pym	3.09(10)	3.9114(19)	2.217(5), 2.040(5)	this work

[a] aromatic groups involved in π-π interactions (py = pyridine; pym = pyrimidine; pz = pyrazine; Ph = phenyl; Cp* = pentamethylcyclopentadienyl ligand; central = middle fragment in platform with three heterocycles; wingtip = side fragment in platform with three heterocycles; pendant = substituent not part of polyheterocycle core);

Additional analysis of structural data: metallocycle planarity vs. polyheterocyclic ligand twist angle

Deformations are observed in the five-membered [RhN_2C_2] metallocycles in the mono- and dirhodium complexes analyzed in this work that correlate with the distortions observed in the polyheterocyclic ligand frameworks. Calculation of the mean planes for the [C_2N_2Rh] metallocycles gives root mean square deviation (RMSD) values between ~0.05 and 0.3 Å (Table S9); across all complexes investigated in the present study–independent of the nature of the polyheterocyclic ligand, the identity of the monodentate ligand at Rh, or the Rh oxidation state–in the absence of substantial twist angles between the heterocycles ($\gamma < 5°$), the RMSD values for the Rh metallocycles correlate linearly ($R^2 = 0.996$, n = 18; blue circles, Figure S61) with the normal distance between Rh and the mean plane defined by the other four atoms in the metallocycle ([C_2N_2]). On the other hand, twist angles greater than ~8.5° (e.g., in **(bppm)Rh-Cl**) do not obey the same correlation. These observations suggest that metallocycle distortions in low twist angle structures are entirely due to the position of the Rh atom; conversely, a greater twist between the heterocycles causes a more substantial deformation of the [C_2N_2] fragment, leading to greater distances between multiple atoms and the calculated mean plane (instead of just Rh). The structures of the anionic bpp ligand (Figure S61, green triangles) are outliers in this context, possibly due to the different constraints enforced by the anionic, 5-membered central heterocycle (pyrazole) vs. the neutral, 6-membered fragments (e.g., pyrimidine, pyrzine) in neutral ligands. **(bpp)(Rh-Cl)$_2$** displays substantial twist angles, which result in significant metallocycle deformation beyond the position of Rh (as observed in the large N–C–C–N torsion angles, >12°), yet are in closer agreement with the correlation observed for low twist angle structures.

Table S8. Metallocycle structural metrics for the complexes investigated in the present study in order of increasing twist angle (γ)[a].

Complex	twist angle (γ)[a], °	$d_{Rh-[C2N2]}$[b], Å	[RhC_2N_2] RMSD[c]	Ref.
**(bpy)Rh-Cl**[d]	0.0(5)	0.062(9)	0.013	[17]
**(bpy)Rh-Cl**[d]	0.0(5)	0.23(2)	0.047	[18]
**(bpy)Rh-Cl**[d]	0.0(11)	0.074(14)	0.016	[19]
**(bpym)Rh-Cl**	0.0(2)	0.071(5)	0.015	this work
**(bpym)(Rh-Cl)$_2$**	0.0(7)	0.179(9)	0.036	this work
**[CpRh(bpy)Br]$^+$**	0.03(11)	0.106(5)	0.022	[10]
**(bpy)Rh-NCCH$_3$**	0.17(11)[e]	0.122(5)[e]	0.025[e]	[20]
**(bppm)(Rh-Cl)$_2$**	0.57(7)	0.312(4)	0.065	this work
**(bpy)Rh**[f]	0.92(11)	0.052(5)	0.012	[22]
**(bpym)Rh-Cl•CH$_3$CN**	1.09(7)	0.157(4)	0.033	this work
**(bpy)Rh**[f]	1.80(9)	0.003(4)	0.005	[20]
**(bppd)Rh-Cl**	1.89(12)	0.210(6)	0.045	[12]
**CpRh(bpy)**	2.29(6)	0.089(3)	0.020	[11]
**(bppm)Rh**	2.41(16)	0.098(8)	0.021	this work
**(bppm)Rh**	2.63(17)[e]	0.095(8)[e]	0.021[e]	this work
**(bpy)Rh-NCCH$_3$**	1.24(8)	0.054(4)	0.012	[20]
**(bpym)Rh-NCCH$_3$**	2.92(10)	0.109(5)	0.024	this work
**(bppm)Rh-NCCH$_3$**	4.29(7)	0.311(3)	0.066	this work
**(bppm)Rh-Cl**	8.93(6)	0.056(3)	0.028	this work
**(bppz)Rh-Cl**	9.2(6)	0.03(3)	0.032	[15]
**[CpRh(bpy)I]$^+$**	9.8(2)	0.055(8)	0.031	[11]
**(bppz)Rh-Cl**	10.18(11)	0.069(5)	0.035	[13]
**(bppz)Rh-Cl**	10.26(18)	0.070(8)	0.035	[14]
**(bppz)Rh-Cl**	11.5(6)	0.07(3)	0.037	[15]
**(bpp)(Rh-Cl)$_2$**	13.8(4)	0.482(15)	0.106	[21]
**(bpp)(Rh-Cl)$_2$**	14.8(4)	0.432(15)	0.100	[21]

[a] as defined in Chart S1; [b] normal distance between Rh and the mean plane calculated for the [C_2N_2] fragment in the metallocycle; [c] Root Mean Square Deviation (RMSD) for calculated mean plane for Rh metallocycle.

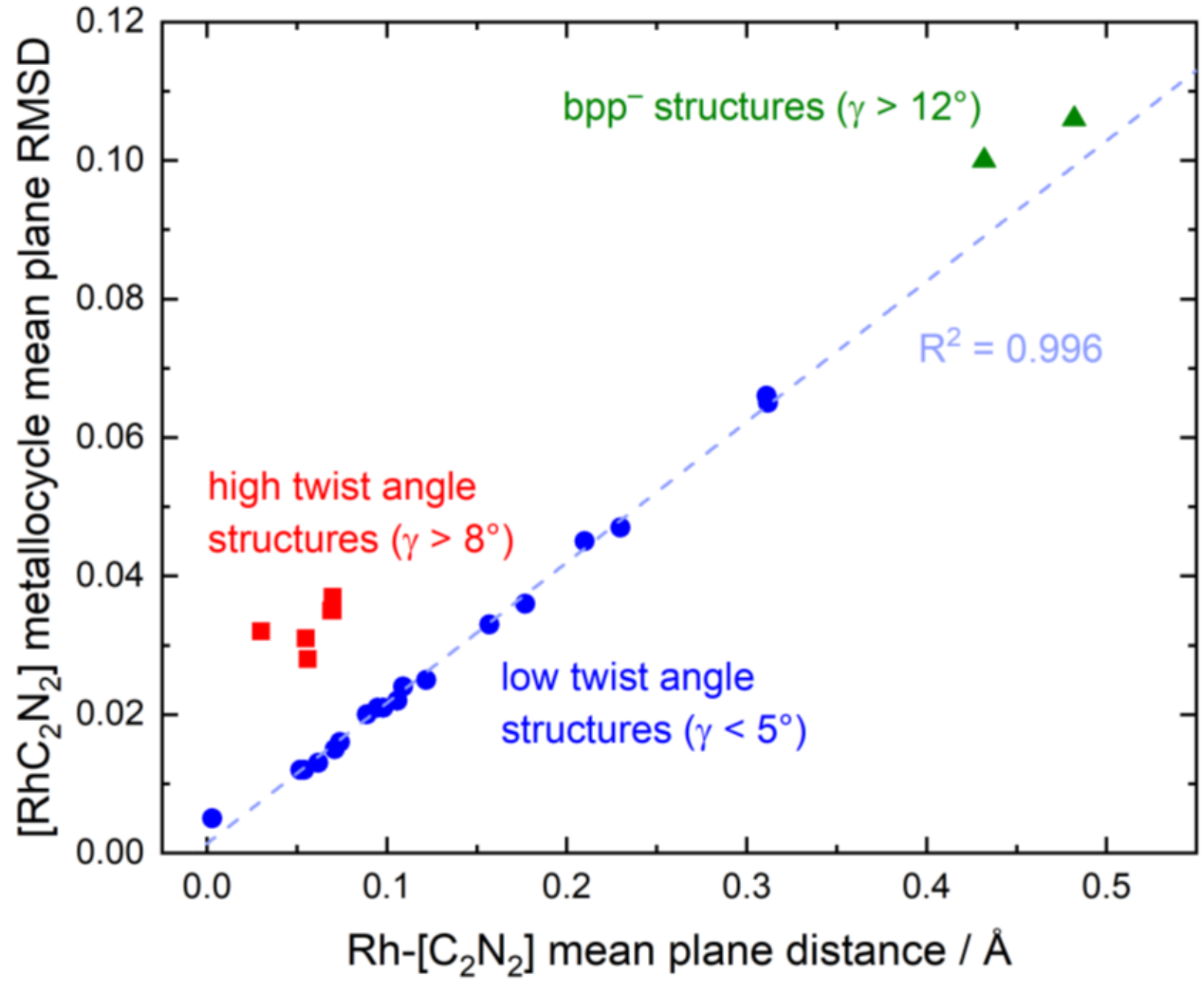

Figure S90. Plot of the RMSD for the best-fit plane for the 5-membered metallocycle vs. the normal distance between Rh and the mean plane defined by the non-Rh atoms in the metallocycle ([C_2N_2]). Blue circles: structures with small twist angles ($\gamma < 5°$, n = 18); red squares: structures with large twist angles ($\gamma > 8°$, n = 6); green triangles: bpp^- structures ($\gamma > 12°$, n = 2). Twist angle as defined in Chart S1.

Refinement details for (bppm)Rh-Cl
No special refinement necessary.

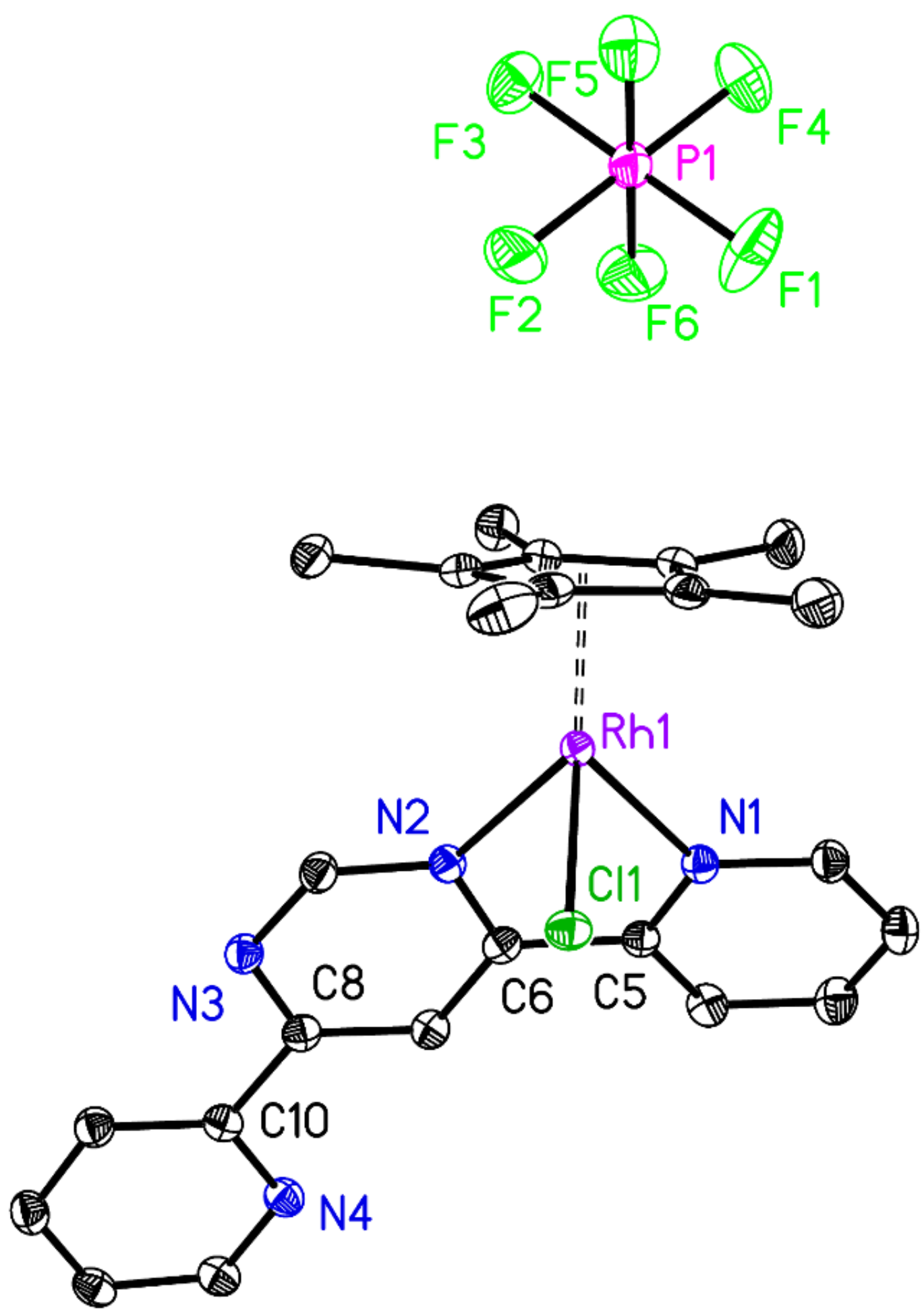

Figure S91. Full solid-state structure of **(bppm)Rh-Cl**. Hydrogen atoms omitted for clarity. Displacement ellipsoids shown at the 50% probability level. Data collected at 100 K.

Refinement details for (bppm)(Rh-Cl)$_2$

(bppm)(Rh-Cl)$_2$ crystallizes as a solvate with a single co-crystallized Et_2O solvent molecule.

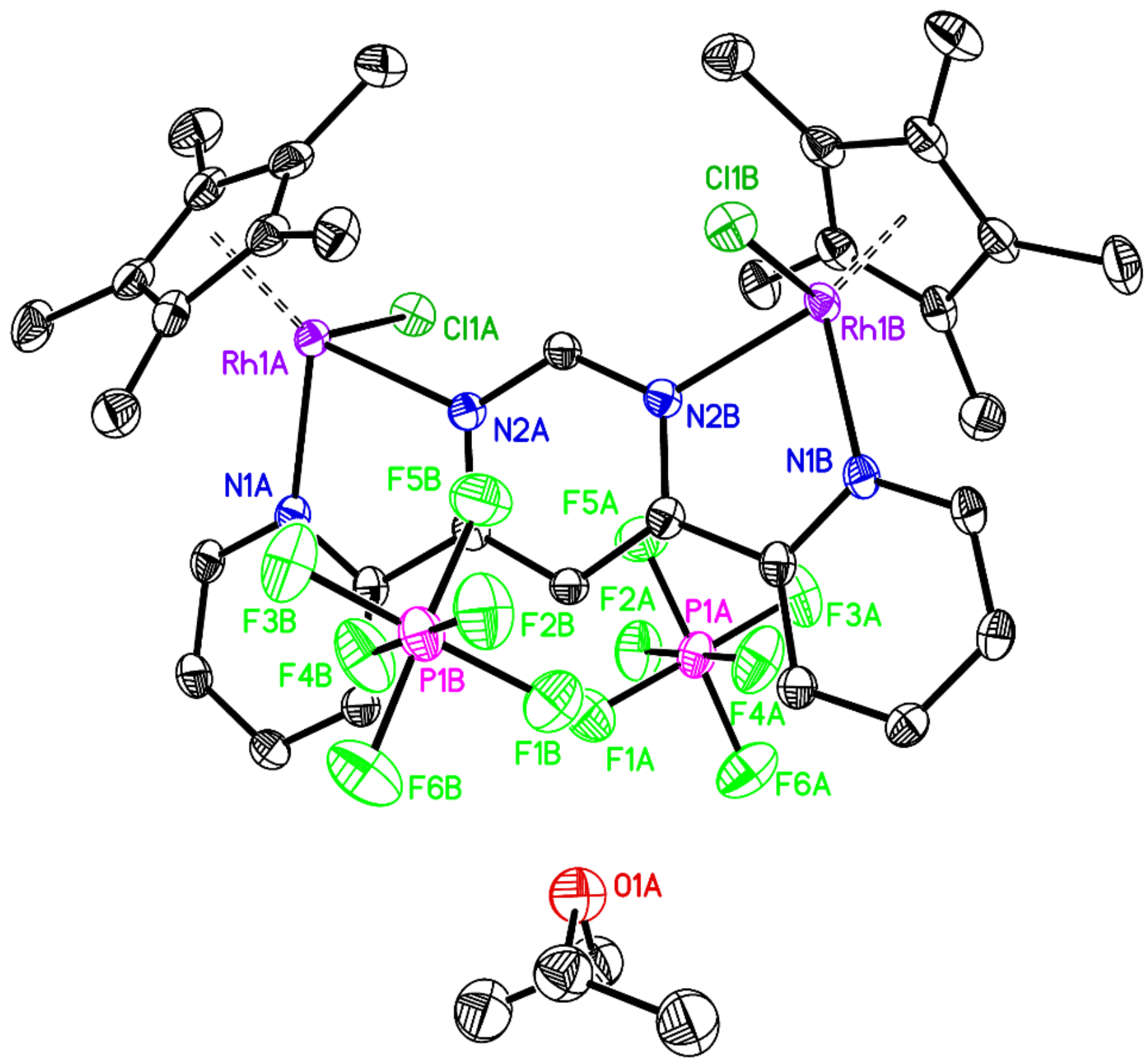

Figure S92. Full solid-state structure of **(bppm)(Rh-Cl)$_2$**. Hydrogen atoms omitted for clarity. Displacement ellipsoids shown at the 50% probability level. Data collected at 100 K.

Refinement details for (bppm)Rh-NCCH$_3$

(bppm)Rh-NCCH$_3$ crystallizes as a solvate with a single co-crystallized CH_3CN solvent molecule. One of the two outer-sphere PF_6^- counteranions was disordered over two positions, which were modeled as two distinct orientations with 75:25 occupancy.

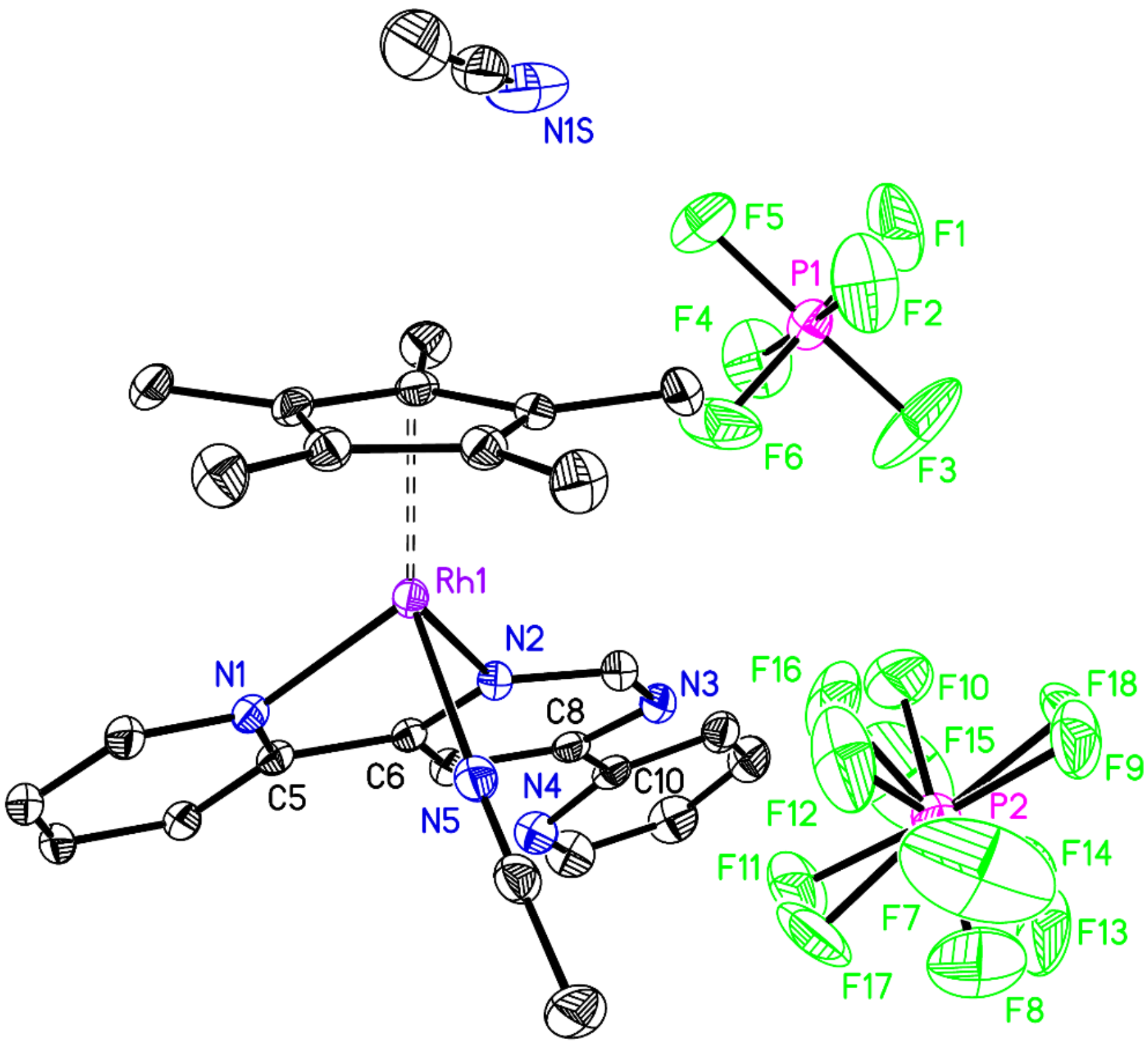

Figure S93. Full solid-state structure of **(bppm)Rh-NCCH$_3$**. Hydrogen atoms omitted for clarity. Displacement ellipsoids shown at the 50% probability level. Data collected at 100 K.

Refinement details for (bppm)Rh

(bppm)Rh crystallizes as a twinned structure (twin law= [–1 0 0, 0 –1 0, 0 0 –1]).

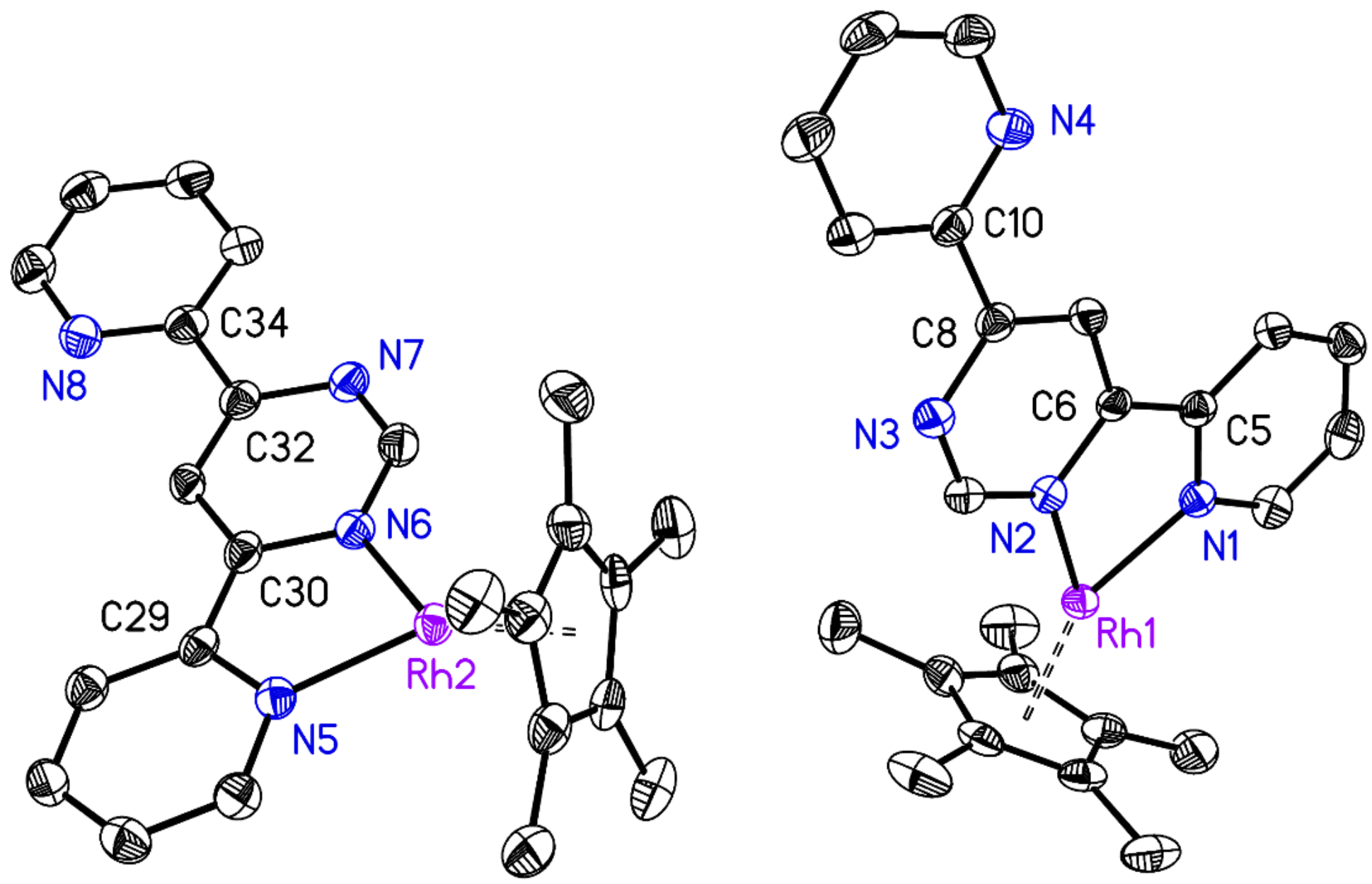

Figure S94. Full solid-state structure of **(bppm)Rh**. Hydrogen atoms omitted for clarity. Displacement ellipsoids shown at the 50% probability level. Data collected at 100 K.

Refinement details for (bpym)Rh-Cl

No special refinement necessary.

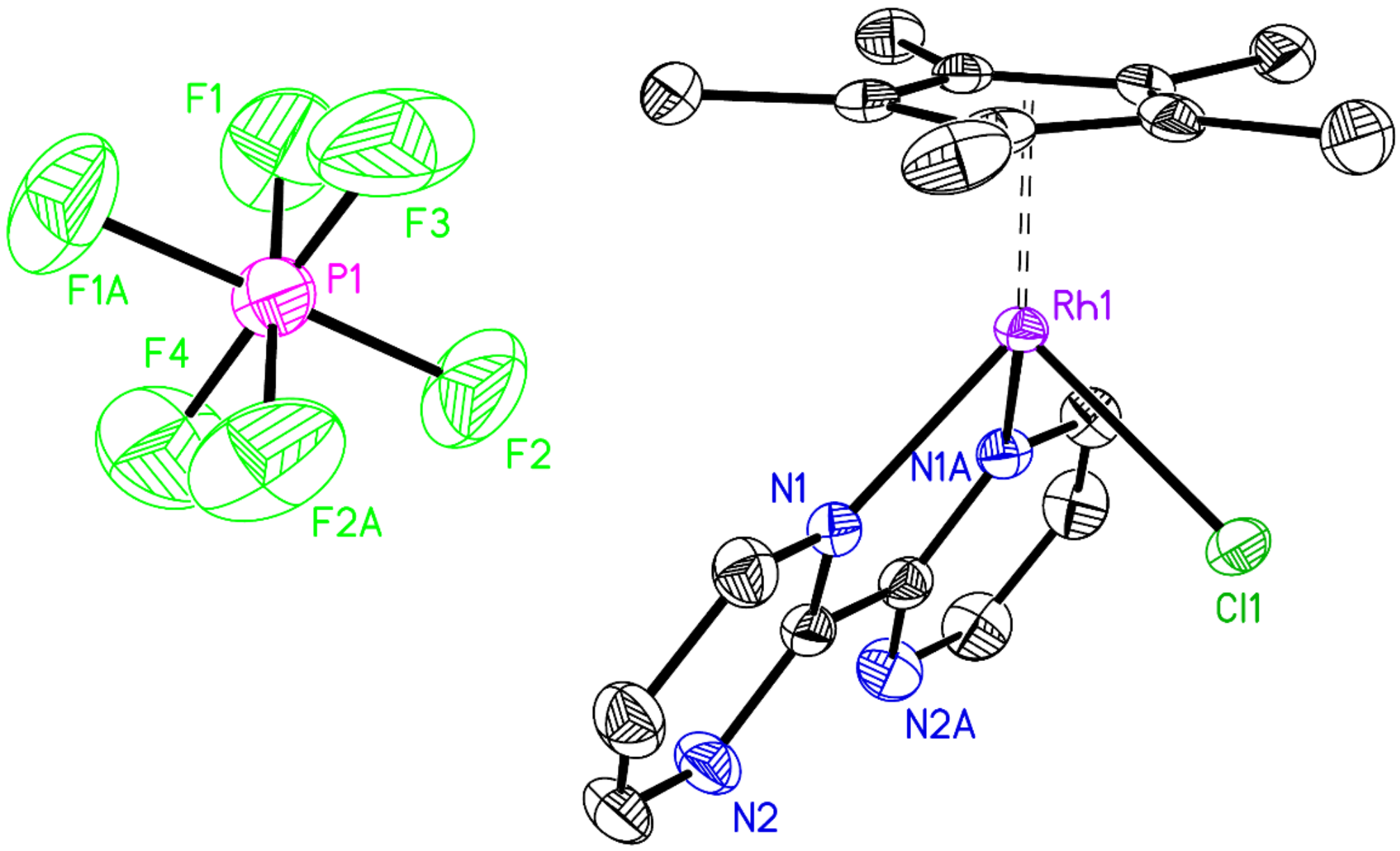

Figure S95. Full solid-state structure of **(bpym)Rh-Cl**. Hydrogen atoms omitted for clarity. Displacement ellipsoids shown at the 50% probability level. Data collected at 100 K.

Refinement details for (bpym)Rh-Cl•CH_3CN

(bpym)Rh-Cl•CH_3CN crystallizes as a solvate with a single co-crystallized CH_3CN solvent molecule.

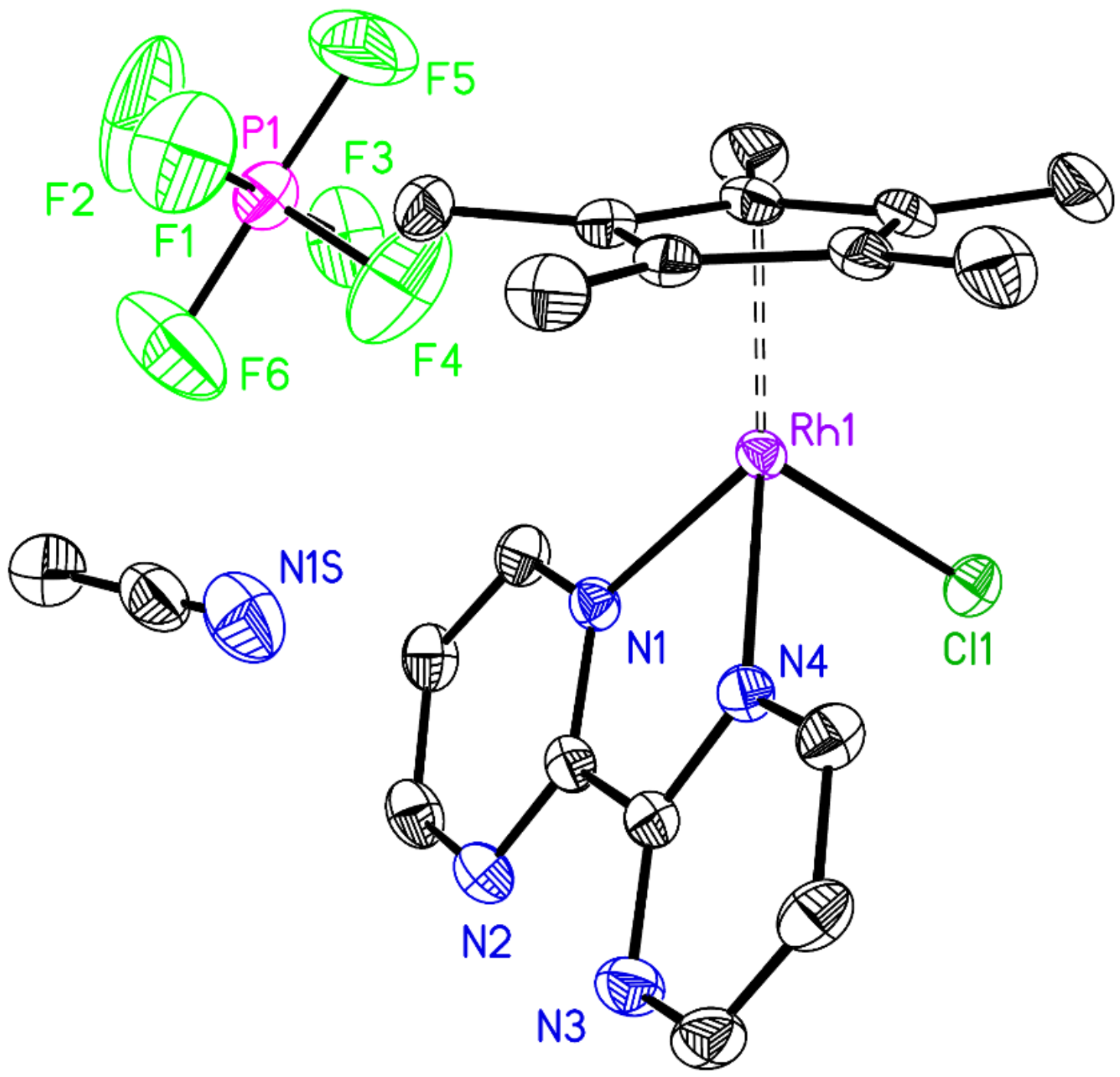

Figure S96. Full solid-state structure of **(bpym)Rh-Cl•CH_3CN**. Hydrogen atoms omitted for clarity. Displacement ellipsoids shown at the 50% probability level. Data collected at 100 K.

Refinement details for (bpym)(Rh-Cl)$_2$

(bpym)(Rh-Cl)$_2$ crystallizes as a solvate with co-crystallized CH_3CN and Et_2O molecules. The disordered Et_2O molecule was modeled using the Disordered Structure Refinement (DSR) plugin[23] and SADI, SIMU, and RIGU restraints. One of the two outer-sphere PF_6^- counteranions was disordered over two positions, which were modeled as two distinct orientations with 58:42 occupancy using an EADP constraint for two *trans* F atoms (F10 and F11).

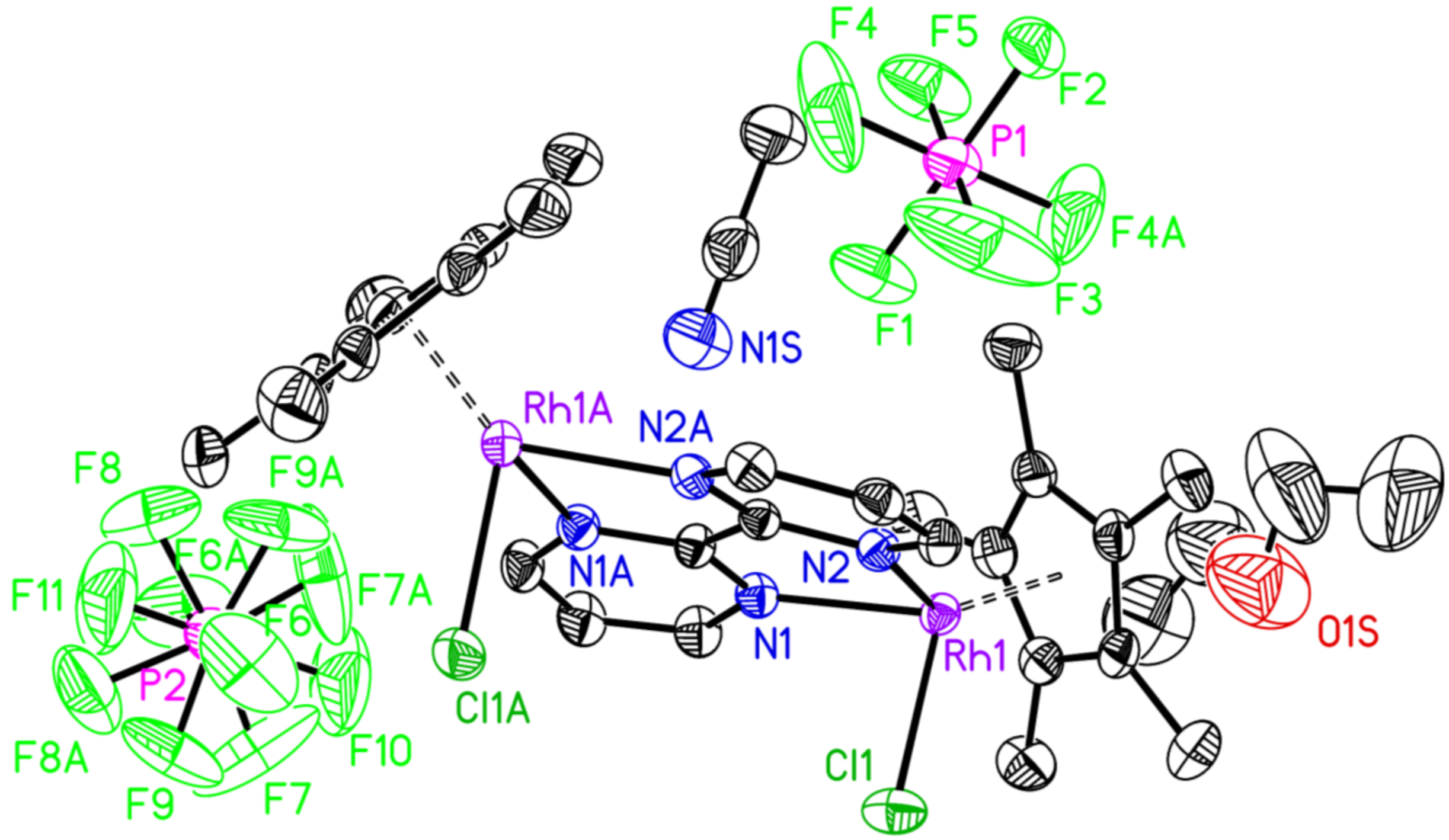

Figure S97. Full solid-state structure of **(bpym)(Rh-Cl)$_2$**. Hydrogen atoms omitted for clarity. Displacement ellipsoids shown at the 50% probability level. Data collected at 100 K.

Refinement details for (bpym)Rh-NCCH$_3$

(bpym)Rh-NCCH$_3$ crystallizes as a solvate with two co-crystallized CH_3CN molecules. One of the two outer-sphere PF_6^- counteranions was disordered over two positions, which were modeled as two distinct orientations with 83:17 occupancy.

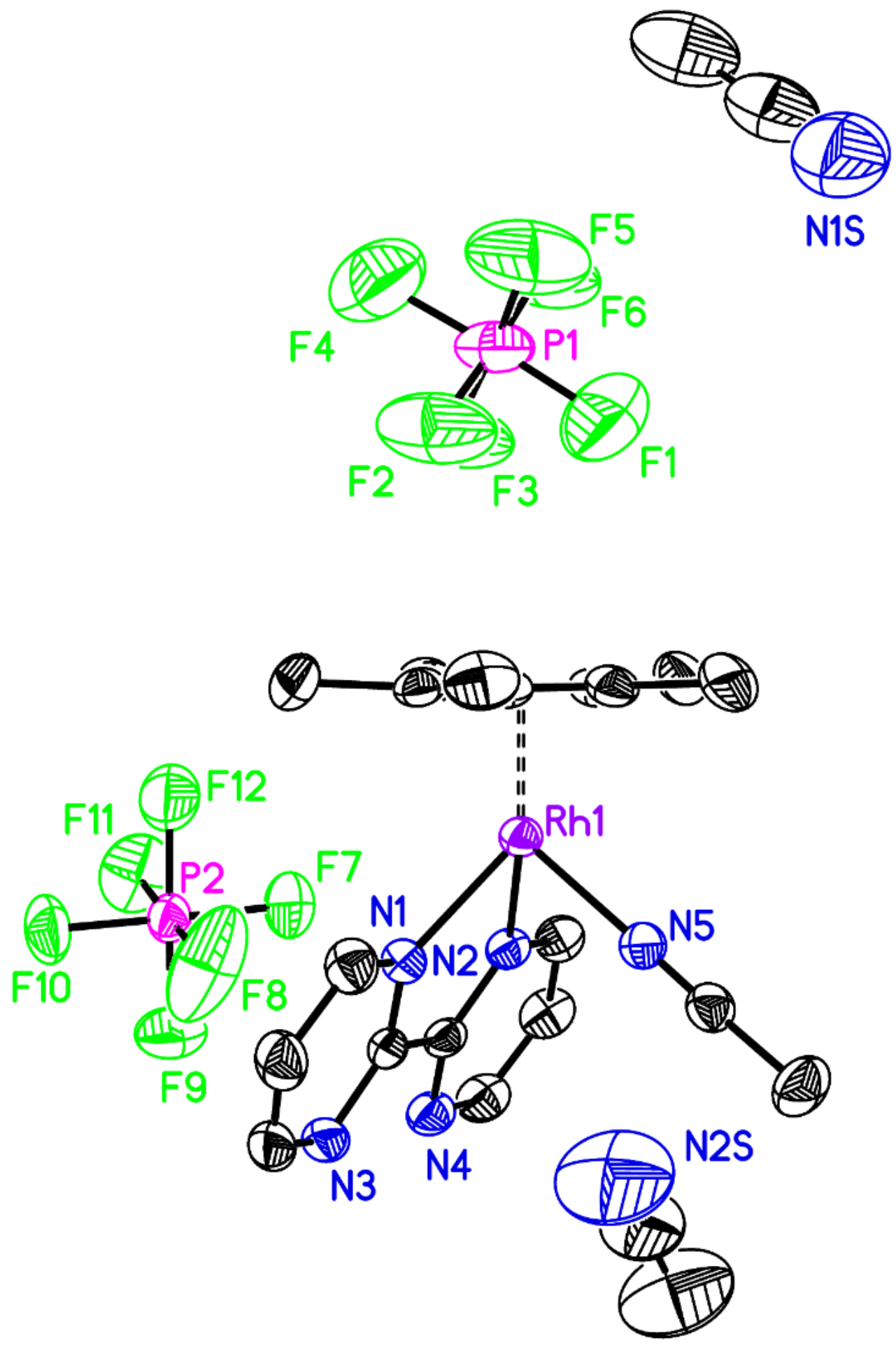

Figure S98. Full solid-state structure of **(bpym)Rh-NCCH$_3$**. Hydrogen atoms omitted for clarity. Only one of two orientation of disordered PF_6^- counteranion (P2) shown for clarity. Displacement ellipsoids shown at the 50% probability level. Data collected at 100 K.

References

(1) Pazderski, L., ^{15}N NMR coordination shifts in Pd(II), Pt(II), Au(III), Co(III), Rh(III), Ir(III), Pd(IV), and Pt(IV) complexes with pyridine, 2,2′-bipyridine, 1,10-phenanthroline, quinoline, isoquinoline, 2,2′-biquinoline, 2,2′: 6′, 2′-terpyridine and their alkyl or aryl derivatives. *Magn. Reson. Chem.* **2008**, *46*, S3-S15.

(2) Boyd, E. A.; Lionetti, D.; Henke, W. C.; Day, V. W.; Blakemore, J. D., Preparation, Characterization, and Electrochemical Activation of a Model [Cp*Rh] Hydride. *Inorg. Chem.* **2019**, *58*, 3606-3615.

(3) Hopkins, J. A.; Lionetti, D.; Day, V. W.; Blakemore, J. D., Chemical and Electrochemical Properties of [Cp*Rh] Complexes Supported by a Hybrid Phosphine-Imine Ligand. *Organometallics* **2019**, *38*, 1300-1310.

(4) Comadoll, C. G.; Henke, W. C.; Hopkins Leseberg, J. A.; Douglas, J. T.; Oliver, A. G.; Day, V. W.; Blakemore, J. D., Examining the Modular Synthesis of [Cp*Rh] Monohydrides Supported by Chelating Diphosphine Ligands. *Organometallics* **2021**, *40*, 3808-3818.

(5) Vicente, J.; Chicote, M. T.; Vicente-Hernández, I.; Bautista, D., On the Reactivity Toward Ketones of New Methyl Amino Complexes of Rh(III) and Ag(I). Synthesis of Ortho-Rhodiated Acetophenone Methyl Imine Complexes. *Inorg. Chem.* **2007**, *46*, 8939-8949.

(6) Chugh, V.; Wu, J.; Leutzsch, M.; Randel, H.; Weyhermüller, T.; Auer, A. A.; Farès, C.; Werlé, C., Controlling hydrogen transfer dynamics in adaptive semihydrogenation of alkynes: Unveiling and directing outer- vs. inner-sphere mechanisms. *Chem Catal.* **2024**, *4*, 101078.

(7) Ferrer-Bru, C.; Ferrer, J.; Passarelli, V.; Lahoz, F. J.; García-Orduña, P.; Carmona, D., Molecular Dihydrogen Activation by (C_5Me_5)M/N (M=Rh, Ir) Transition Metal Frustrated Lewis Pairs: Reversible Proton Migration to, and Proton Abstraction from, the C_5Me_5 Ligand. *Chem. – Eur. J.* **2024**, *30*, e202304140.

(8) Govindaswamy, P.; Canivet, J.; Therrien, B.; Suss-Fink, G.; Stepnicka, P.; Ludvik, J., Mono and dinuclear rhodium, iridium and ruthenium complexes containing chelating 2,2'-bipyrimidine ligands: Synthesis, molecular structure, electrochemistry and catalytic properties. *J. Organomet. Chem.* **2007**, *692*, 3664-3675.

(9) Kaim, W.; Reinhardt, R.; Greulich, S.; Sieger, M.; Klein, A.; Fielder, J., Coupling of electron transfer and bond dissociation processes in dinuclear complexes with rhodium and iridium reaction centres bridged by 2,2′-bipyrimidine. *Collect. Czech. Chem. Commun.* **2001**, *66*, 291-306.

(10) Kuvshinova, S. S.; Nelyubina, Y. V.; Larionov, V. A.; Vorobyeva, D. V.; Osipov, S. N.; Loginov, D. A., Half-sandwich complexes of group 9 metals with N,N′-ligands for CF_3-carbenoid alkylation of N-(pyrimidin-2-yl)indole. *J. Organomet. Chem.* **2021**, *946-947*, 121899.

(11) Lionetti, D.; Day, V. W.; Blakemore, J. D., Synthesis and Electrochemical Properties of Half-Sandwich Rhodium and Iridium Methyl Complexes. *Organometallics* **2017**, *36*, 1897-1905.

(12) Prasad, K. T.; Gupta, G.; Chandra, A. K.; Pavan, M. P.; Rao, K. M., Spectral, structural and DFT studies of platinum group metal 3,6-bis(2-pyridyl)-4-phenylpyridazine complexes and their ligand bonding modes. *J. Organomet. Chem.* **2010**, *695*, 707-716.

(13) Pandey, D. S.; Puerta, M. C.; Valerga, P., *CSD Communication* **2015**

(14) Pandey, D. S.; Valerga, P.; Puerta, M. C., *CSD Communication* **2009**

(15) Singh, S. K.; Chandra, M.; Dubey, S. K.; Pandey, D. S., Tuned helical array of Rh^{III}/Ir^{III} Cp* complexes with polypyridyl ligands. *Eur. J. Inorg. Chem.* **2006**, 3954-3961.

(16) Janiak, C., A critical account on π–π stacking in metal complexes with aromatic nitrogen-containing ligands. *J. Chem. Soc. Dalton Trans.* **2000**, 3885-3896.

(17) Banerjee, S.; Soldevila-Barreda, J. J.; Wolny, J. A.; Wootton, C. A.; Habtemariam, A.; Romero-Canelón, I.; Chen, F.; Clarkson, G. J.; Prokes, I.; Song, L.; O'Connor, P. B.; Schünemann, V.; Sadler, P. J., New activation mechanism for half-sandwich organometallic anticancer complexes. *Chem. Sci.* **2018**, *9*, 3177-3185.

(18) Scharwitz, M. A.; Ott, I.; Geldmacher, Y.; Gust, R.; Sheldrick, W. S., Cytotoxic half-sandwich rhodium(III) complexes: Polypyridyl ligand influence on their DNA binding properties and cellular uptake. *J. Organomet. Chem.* **2008**, *693*, 2299-2309.
(19) Dadci, L.; Elias, H.; Frey, U.; Hornig, A.; Koelle, U.; Merbach, A. E.; Paulus, H.; Schneider, J. S., Pi-Arene Aqua Complexes of Cobalt, Rhodium, Iridium, and Ruthenium - Preparation, Structure, and Kinetics of Water Exchange and Water Substitution. *Inorg. Chem.* **1995**, *34*, 306-315.
(20) Blakemore, J. D.; Hernandez, E. S.; Sattler, W.; Hunter, B. M.; Henling, L. M.; Brunschwig, B. S.; Gray, H. B., Pentamethylcyclopentadienyl rhodium complexes. *Polyhedron* **2014**, *84*, 14-18.
(21) Koizumi, T.; Ohkura, Y.; Tsuda, E., Synthesis, structure, and electrochemical behavior of a new dinuclear Rh(III) complex bridged by a bpp^- ligand (bpp^-=3,5-bis(2-pyridyl)pyrazolate). *Inorg. Chem. Commun.* **2016**, *67*, 25-28.
(22) Nakai, H.; Jeong, K.; Matsumoto, T.; Ogo, S., Catalytic C-F Bond Hydrogenolysis of Fluoroaromatics by [(η^5-C_5Me_5)Rh^I(2,2'-bipyridine)]. *Organometallics* **2014**, *33*, 4349-4352.
(23) Kratzert, D.; Krossing, I., Recent improvements in DSR. *J. Appl. Crystallogr.* **2018**, *51*, 928-934.
